# Synthetic circular RNA switches and circuits that control protein expression in mammalian cells

**DOI:** 10.1093/nar/gkac1252

**Published:** 2023-01-16

**Authors:** Shigetoshi Kameda, Hirohisa Ohno, Hirohide Saito

**Affiliations:** Department of Life Science Frontiers, Center for iPS Cell Research and Application (CiRA), Kyoto University, 53 Kawahara-cho, Shogoin, Sakyoku, Kyoto, 606-8507, Japan; Graduate School of Medicine, Kyoto University,Yoshida-Konoe-cho, Sakyo-ku, Kyoto, 606-8501, Japan; Department of Life Science Frontiers, Center for iPS Cell Research and Application (CiRA), Kyoto University, 53 Kawahara-cho, Shogoin, Sakyoku, Kyoto, 606-8507, Japan; Department of Life Science Frontiers, Center for iPS Cell Research and Application (CiRA), Kyoto University, 53 Kawahara-cho, Shogoin, Sakyoku, Kyoto, 606-8507, Japan

## Abstract

Synthetic messenger RNA (mRNA) has been focused on as an emerging application for mRNA-based therapies and vaccinations. Recently, synthetic circular RNAs (circRNAs) have shown promise as a new class of synthetic mRNA that enables superior stability and persistent gene expression in cells. However, translational control of circRNA remained challenging. Here, we develop ‘circRNA switches’ capable of controlling protein expression from circRNA by sensing intracellular RNA or proteins. We designed microRNA (miRNA) and protein-responsive circRNA switches by inserting miRNA-binding or protein-binding sequences into untranslated regions (UTRs), or Coxsackievirus B3 Internal Ribosome Entry Site (CVB3 IRES), respectively. Engineered circRNAs efficiently expressed reporter proteins without inducing severe cell cytotoxicity and immunogenicity, and responded to target miRNAs or proteins, controlling translation levels from circRNA in a cell type-specific manner. Moreover, we constructed circRNA-based gene circuits that selectively activated translation by detecting endogenous miRNA, by connecting miRNA and protein-responsive circRNAs. The designed circRNA circuits performed better than the linear mRNA-based circuits in terms of persistent expression levels. Synthetic circRNA devices provide new insights into RNA engineering and have a potential for RNA synthetic biology and therapies.

## INTRODUCTION

Gene delivery using synthetic messenger RNA (mRNA) is an effective method for transient gene expression, with a reduced risk of genomic integration in the cell (1). One of the limitations hindering its broader application for medical research is lower gene expression persistence caused by its instability. To improve the stability and performance of synthetic mRNA, substantial efforts have been made to engineer new RNA structures and, in recent years, synthetic circular RNAs (circRNAs) have shown promise as a new class of synthetic mRNA with superior stability and persistent gene expression (2–4).

Endogenous circRNAs are generated through back-splicing (5). When first discovered, they were thought to be products of splicing errors (6). However, they have since been reported to be involved in various biological functions as either protein-coding or non-coding RNA (7). Additionally, these circRNAs resist exonuclease-mediated degradation as their covalent-closed loop structure lacks free 5′ and 3′ ends. This common structural feature allows circRNAs to exhibit a longer half-life than linear mRNAs in cells (7,8).

To expand the potential of mRNA therapeutics, it is important to produce desired outputs depending on the cell state and reduce off-target effects in non-target cells and tissues (9,10). Such ‘smart mRNA’ could be a useful tool for cell type-specific gene regulation and future therapeutic applications (11,12). Even before the function of endogenous circRNAs was determined, there had been efforts to adapt the circular structure to improve the stability of synthetic RNAs (13,14). However, research in the field of circRNA is relatively early, and studies have mainly focused on the methods of cyclization (3), the immunogenicity (15–18), and the performance of translation (3,19). Design principles of circRNAs that sense intracellular conditions and autonomously control their translation level remain unknown. Defining these principles would be useful in the development of therapies with suppressed off-target expression in non-target cells, maximizing the therapeutic effect.

MicroRNAs (miRNAs) are small non-coding RNAs that regulate protein expression from mRNAs via translational repression and mRNA degradation (20). As the activity of miRNAs differs among cell types, they can be used as an indicator to distinguish between various cell types (21). We have previously developed miRNA-responsive linear mRNA (miRNA-responsive switch), which is composed of a protein-coding sequence and an antisense sequence to the target of the miRNA (anti-miR) at the untranslated region (UTR), enabling the distinguishment of cell types based on endogenous miRNA activity. We have succeeded in regulating gene expression using miRNA-responsive switches in a cell type-specific manner for various applications, including cell purification derived from human pluripotent stem cells, selective killing of cancer cells, and genome editing of target cells (22–25). Notably, these miRNA-responsive switches did not change the expression level of endogenous target miRNA and global gene expression patterns regulated by miRNAs, indicating that perfect base-pairing between miRNA and its antisense target induces mRNA cleavage through siRNA-like pathways with the RNA-induced silencing complex (RISC) (i.e. miRNA-responsive switches do not function as a miRNA ‘sponge’) (22,25). Studies on endogenous circRNAs suggested that circRNAs with partially matched miRNA target sites have been reported as potential miRNA sponges or inhibitors (26–28), whereas circRNAs with a near-perfect miRNA target site undergo degradation by sensing miRNA, much like linear mRNAs (29). Therefore, to perform cell-type-specific protein expression from circRNA, we expected that miRNA-responsive switch technology could be applied.

RNA-binding protein (RBP)-responsive mRNA switches also allow the control of protein expression from linear mRNAs in a cell-type-specific manner. In previous studies, protein-responsive switches have been designed to repress cap-dependent translation of linear mRNAs by inserting the protein-binding aptamer sequence at the 5′-UTR (23,30,31). The virus-derived Internal Ribosome Entry Site (IRES) can be used for designing translatable circRNA (2,3), and its underlying translation mechanism and the composition of its initiation factors are similar to those of cap-dependent translation (32). We expected that IRES-dependent translation could be controlled by RBPs, similar to the protein-responsive switches reported previously (23,30,31). So far, endogenous circRNAs have been reported to function as sponges or scaffolds for RBPs (33–36), and synthetic circRNA devices have mimicked these functions, only as RBP regulators or inhibitors (18,37,38). In translatable circRNAs, no devices have been reported in which RBPs regulate the translation of circRNA. Thus, we aimed to investigate the design principles of protein-responsive switches for cap-independent translation of circRNA.

**Figure 1.**
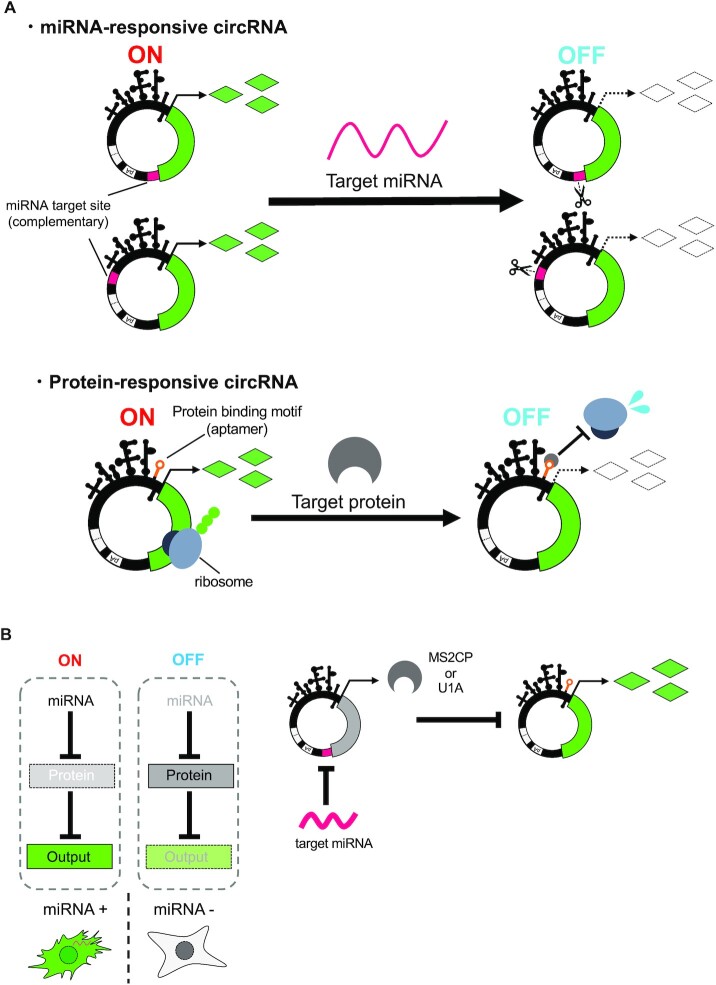
Schematic illustrations of circRNA switches and circuits. (**A**) Design of miRNA or protein-responsive circRNA switch. miRNA-responsive circRNA has the antisense of target miRNA sequence at the UTR. Protein-responsive circRNA has a protein-binding motif in IRES region. In both systems, gene expression from circRNA is repressed if the target miRNA or protein is present. (**B**) Scheme of circRNA circuit composed of miRNA- and protein-responsive circRNA switches. The first output (MS2CP or U1A protein) is encoded on a miRNA-responsive circRNA switch, and the second output (reporter protein) is encoded on a protein-responsive circRNA switch. In the OFF state (absence of input miRNAs), MS2CP or U1A protein represses translation of the second output gene-coding circRNA. In ON state (presence of input miRNAs), the MS2CP or U1A translation is repressed by the miRNAs, which leads to output translation.

Here we present a design strategy for developing synthetic circRNA devices that control circRNA translation in a cell type-specific manner by detecting target miRNAs and RBPs (Figure 1). We designed miRNA- and RBP-responsive circRNA switches by engineering the UTRs and Coxsackievirus B3 (CVB3) IRES and constructed synthetic circRNA circuits for RNA-only delivery. The designed circRNA circuits showed better expression level persistence than linear mRNA-based circuits.

## MATERIALS AND METHODS

### Template plasmid construction for circRNA and linear mRNA

All PCRs were performed by PrimeSTAR MAX DNA polymerase (TaKaRa). After the template was digested by DpnI (TOYOBO) at 37°C for 30 min, PCR products were purified by QIAquick PCR Purification Kit (QIAGEN) or Monarch DNA Gel Extraction Kit (New England Biolabs). To prepare template plasmids for RNA, PCR products were cloned into PCR-linearized pUC19 vector by Gibson assembly using NEBuilder HiFi DNA Assembly Master Mix (New England Biolabs) and transformed to DynaCompetent Cells JetGiga *Escherichia coli* DH5α (BioDynamics Laboratory). After culturing *Escherichia coli* at 37°C for 8–16 h with 3 ml LB/Ampicillin (Amp) media, plasmids were purified by NucleoSpin Plasmid EasyPure (TaKaRa). For template plasmids for internal polyA-containing circRNAs, the internal polyA part was inserted and cloned by using synthetic ssDNA has A120 mer with 20 nt overhang for assembling (5′-ttGAATAAAGCCTGAGTAGGAAAAAAAAAAAAAAAAAAAAAAAAAAAAAAAAAAAAAAAAAAAAAAAAAAAAAAAAAAAAAAAAAAAAAAAAAAAAAAAAAAAAAAAAAAAAAAAAAAAAAAAAAAAAAAAAAAAAAAAAGGCTATTATGCGTTACCGGC-3′). Then, transformed *E. coli* was cultured with 50 ml LB/Amp media, and plasmids were purified by PureYield Plasmid Midiprep System (Promega). All plasmid sequences were checked by the Sanger sequencing method using BigDye Terminator v3.1 Cycle Sequencing Kit (Thermo Fisher Scientific) and 3500xL Genetic Analyzer (Thermo Fisher Scientific).

### Synthesis and purification of circRNA and linear mRNA

DNA templates for in vitro transcription (IVT) were amplified from template plasmids using PrimeSTAR MAX DNA polymerase (TaKaRa). After the template plasmid was digested by DpnI (TOYOBO) at 37°C for 30 min, PCR products were purified by QIAquick PCR Purification Kit (QIAGEN). For IVT templates for internal polyA-containing circRNAs, template plasmids were linearized by EcoRI-HF (New England Biolabs) or BamHI-HF (New England Biolabs) and purified by Monarch PCR & DNA Cleanup Kit (New England Biolabs).

Synthesis of mRNAs used the MEGAscript T7 Kit (Thermo Fisher Scientific). For capped linear mRNA synthesis, transcription was performed with 75 mM Anti Reverse Cap Analog (TriLink BioTechnologies) or G(5′)ppp(5′)A RNA Cap Structure Analog (A-cap) (New England Biolabs Japan) in a GTP 4:1 solution. For modified base-containing circRNA and linear mRNA synthesis, pseudouridine-5′-triphosphate (Ψ) and 5-methylcytidine-5′-triphosphate (m5C) or N1-methylpseudouridine-5′-triphosphate (m1Ψ) (TriLink BioTechnologies) were used instead of uridine triphosphate (U) and cytidine triphosphate (C). IVT reaction mixtures were incubated at 37°C for up to 6 h and then mixed with TURBO DNase (Thermo Fisher Scientific), and additionally incubated at 37°C for 30 min to remove the template DNA. The resulting RNAs were purified by the Monarch RNA Cleanup Kit (New England Biolabs). For circRNAs, they were incubated at 55°C with splicing buffer (50 mM Tris–HCl, 10 mM MgCl_2_, 1 mM DTT, pH 7.5, 2 mM GTP) for 30 min and purified again. Purified RNAs were subjected to 4% denaturing polyacrylamide gel electrophoresis (PAGE) (8.3 M urea) and subsequent elution from the gel by overnight incubation at 37°C with 200 rpm shaking in elution buffer (0.3 M sodium acetate pH 5.2, 0.1% SDS). The eluted RNAs were purified by phenol-chloroform extraction and precipitated with isopropanol. After dissolving the RNA pellet in nuclease- free water, RNAs were desalted using Amicon Ultra 0.5 ml Centrifugal Filters Urtracel-50K (Millipore), then incubated with Antarctic Phosphatase (New England Biolabs) at 37°C for 30 min. Phosphatase-treated RNAs were re-purified by phenol-chloroform extraction and isopropanol-precipitation. In this study, all circRNAs and linear mRNAs were purified from polyacrylamide gel, except for linear mRNAs used in Figure 5C to screen the appropriate variant. Concentrations of purified RNAs were measured by NanoDrop2000 (Thermo Fisher Scientific) and used in cellular experiments. All RNA sequences used in this research are described in Supplementary Sequences.

### shRNA preparation for U1A knockdown

For transcribing shRNAs, a single-strand DNA templates (U1A-shRNA 5′- CTGATCAAGAAGGATGAGCTAAAAAAGCTATGCTCTTTTTTAGCTCATCCTTCTTGATCTATAGTGAGTCGTATTAGC-3′, Control-shRNA 5′ -CTGCCTAAGGTTAAGTCGCCCTCGCCTATGCTGCGAGGGCGACTTAACCTTAGGCTATAGTGAGTCGTATTAGC-3′) were annealed to T7 forward primer (5′ -GCTAATACGACTCACTATAG-3′). Generated partial double-stranded templates were transcribed by using MEGAshortscript T7 Kit (Thermo Fisher Scientific). IVT reaction mixtures were incubated at 37°C for 16 h and then mixed with TURBO DNase (Thermo Fisher Scientific), and additionally incubated at 37°C for 30 min to remove the template DNA. After IVT and template removal, shRNAs were purified by the same procedures described in ‘Synthesis and purification of circRNA and linear mRNA’ with 12% denaturing PAGE and isopropanol-precipitation using Gene-Packman Coprecipitant (Nacalai Tesque).

The shRNA sequences (30) were as follows:

U1A-shRNA 5′-GAUCAAGAAGGAUGAGCUAAAAAAGAGCAUAGCUUUUUUAGCUCAUCCUUCUUGAUCAG-3′
Control-shRNA 5′- GCCUAAGGUUAAGUCGCCCUCGCAGCAUAGGCGAGGGCGACUUAACCUUAGGCAG-3′

### Cell culture and RNA transfection

HEK293FT (Invitrogen), HeLa CCL2 (ATCC) and A549 (RCB3677) cells were cultured in Dulbecco's modified Eagle's medium (DMEM) 4.5 g/l glucose (Nacalai Tesque) supplemented with 10% fetal bovine serum (FBS) (Biocera, Ireland Origin), 0.1 mM MEM non-essential amino acids (Life Technologies), 2 mM l-glutamine (Life Technologies) and 1 mM sodium pyruvate (Nacalai Tesque). All cell lines were cultured at 37°C with 5% CO_2_. All transfections were performed using Lipofectamine MessengerMAX (Thermo Fisher Scientific) according to the manufacturer's protocol. RNAs were co-transfected with synthetic miRNA mimics or inhibitors (Thermo Fisher Scientific) in the case of miRNA-responsive switch experiments. In the case of the RBP-responsive switch experiments, the target RBP was expressed in cells by co-transfecting with RBP-coding mRNAs. The transfection condition details of each experiment are shown in Supplementary Table S1.

### RNase R digestion assay

2.5 μg of *in vitro*-transcribed RNAs (Circular EGFP ΔpAΔIRES) were incubated at 37°C with 10 U of RNase R (Cosmo Bio) in a 10 μl mixture. After a 45 min incubation, the mixture was subjected to 4 and 8% denaturing PAGE (8.3 M urea), and then stained by SYBR Green II Nucleic Acid Gel Stain (TaKaRa). Stained RNA was detected by Typhoon FLA-7000 (GE Healthcare).

### Splice junction sequencing

Circular EGFP ΔpAΔIRES purified from denaturing polyacrylamide gel was reverse transcribed using reverse transcription primer (5′-CCTACTCAGGCTTTATTCAAAGACCAAG-3′) and SuperScript IV Reverse Transcriptase (Thermo Fisher Scientific). Reverse-transcribed cDNA was used as a template for PCR using PrimeSTAR Max DNA Polymerase (TaKaRa) with primer set for splice junction amplification (Fwd: 5′-agctcgccgaccactaccagcag-3′, Rev: 5′-gtagcggctgaagcactgcacg-3′). Amplified product was purified by Monarch DNA Gel Extraction Kit (New England Biolabs), and then sequenced by the same method described in ‘Template plasmid construction for mRNA’.

### Flow cytometry and data analysis

HEK293FT, A549 (1.0 × 10^5^ cells), and HeLa (0.5 × 10^5^ cells) cells were seeded onto 24-well plates 24 h before transfection. All flow cytometry measurements were performed 24 h after the transfection using BD Accuri C6 (BD Biosciences). Cells were washed with phosphate buffered saline (PBS, Nacalai Tesque), trypsinized with 100 μl of 0.25% Trypsin–EDTA (Thermo Fisher Scientific), and incubated at 37°C for 5 min. After incubation, 150 μl of fresh medium was added. Cells were transferred to a fresh microcentrifuge tube passing through a nylon mesh. EGFP was detected by FL1 (533/30 nm, 99% attenuated), and iRFP670 was detected by FL4 (675/25 nm) filters, respectively. Collected data were analyzed using FlowJo 10.5.3 software. For data analysis, gates were generated by using mock samples. Data from the debris were removed when preparing forward versus side dot plots (FSC-A versus SSC-A). Then, events on the chart edges in the dot plots of FL-1 versus FL-4 were removed. In the histogram where iRFP670-intensity is displayed on the X-axis, the iRFP670-positive (reference-positive) gate was defined by a mock sample with 99.9% cells outside the gate. In the following analysis, the mean of EGFP+/iRFP670+ was used for calculation.

### RT-qPCR analysis

For the RT-qPCR targeting immune response-related genes, A549 (1.0 × 10^5^ cells) cells were seeded onto 24-well plates 24 h before transfection. Then, cells were washed with 1 ml of PBS and total RNA was extracted 24 h after transfection. To induce immune response-related genes, 200 ng of Polyinosinic-polycytidylic acid [Poly(I:C)] (Enzo Life Sciences, Inc.) was transfected as a positive control. Total RNA extraction was performed using TRIzol Reagent (Thermo Fisher Scientific) and Monarch RNA Cleanup Kit (New England Biolabs) according to the manufacturer's protocol. 400 ng total RNA was used as a template for reverse transcription performed using ReverTra Ace qPCR RT Master Mix with gDNA Remover (TOYOBO) in a 10 μl reaction mixture. All optional steps described in the kit manual were applied.

For the RT-qPCR targeting synthetic linear mRNAs and circRNAs (Supplementary Figures S2B and S4B), HEK293FT (1.0 × 10^5^ cells) cells were seeded onto 24-well plates 24 h before transfection. Then, total RNA was extracted at each time point after transfection. Extracted total RNA was treated with TURBO DNase (Thermo Fisher Scientific) and re-purified by Monarch RNA Cleanup Kit (New England Biolabs) according to the manufacturer's protocol. Reverse transcription was performed using a High-Capacity cDNA Reverse Transcription Kit (Thermo Fisher Scientific) with 100 ng total RNA template and 20 μl reaction volume. In both cases, the synthesized cDNA solution was diluted by nuclease-free water with 5-fold dilution. One μl of the diluted-cDNA solution was analyzed by qPCR. The qPCR analysis was performed using THUNDERBIRD Next SYBR qPCR Mix (TOYOBO) with 20 μl reaction mixture and QuantStudio 3 Real-time PCR Systems (Thermo Fisher Scientific) following the manufacturer's protocol, with three steps reaction. Target mRNA quantities were normalized by ATP5B mRNA. All qPCRs were performed in technical duplicates and the averages of Ct were processed to calculate relative expression levels using the ΔCt or ΔΔCt method. The primers for qPCR are listed in Supplementary Table S2.

### Western blot analysis

HEK293FT (1.0 × 10^5^ cells) cells were seeded onto 24-well plates 24 h before transfection. 24 h after transfection, cells were washed with 1 ml of PBS and lysed in 50 μl of RIPA buffer (Nacalai Tesque). The cell lysates were analyzed with subsequent western blotting as previously performed (30). The transferred membranes were incubated with specific primary antibodies, Anti-SNRPA (Santa Cruz Biotechnology, 200-fold dilution) and Anti-Enterobacterio Phage MS2 Coat Protein (Sigma-Aldrich, 5000-fold dilution), respectively. Anti-GAPDH antibody (Santa Cruz Biotechnology) was used at 500-fold dilution. Then, the blot was incubated with secondary antibodies. Goat Anti-Mouse IgG (H + L)-HRP conjugate (Bio-Rad) or Goat anti-Rabbit IgG (H + L)-HRP conjugate (BIO-RAD) was used at 400-fold dilution. All incubation steps were performed using iBind Flex Western Device (Thermo Fisher Scientific). Detection of the blot was performed with ECL Prime Western Blotting Detection Reagent (GE Healthcare) and Amersham ImageQuant 800 (Cytiva). The protein expression level was calculated from band intensities with ImageJ (NIH).

### WST-1 assay

HEK293FT, A549 (2.0 × 10^4^ cells) and HeLa (1.0 × 10^4^ cells) were seeded onto 96-well plates 24 h before transfection. 24 h after transfection, 10 μl/well of WST-1 reagent (Sigma-Aldrich) was added to the medium of each well, and the plates were incubated for 1 h at 37°C. After the incubation, the absorbance of 440 and 620 nm was measured by PE Envision 2104 Multilabel Reader (PerkinElmer).

### Secreted luciferase assay

HEK293FT, A549 (1.5 × 10^4^ cells) and HeLa (0.5 × 10^4^ cells) cells were seeded onto 24-well plates 24 h before transfection. After transfection, culture media was harvested and replaced every 24 h up to 5 days (120 h) at each time point. Culture media was harvested and replaced for 24–120 h after transfection at each time point. Media was harvested into Protein LoBind tubes (Eppendorf) and stored at −30°C. To detect bioluminescence from Metridia Luciferase (MetLuc2), 50 μl of harvested media was transferred into a Greiner LUMITRA 200 microplate (Greiner), and then 10 μl of 0.5× substrate/reaction buffer from Ready-To-Glow Secreted Luciferase Reporter Assay (TaKaRa) was added by injector attached on a plate reader. After 30s double orbit shaking with 3.0 mm diameter and 30s incubation delay, luminescence was detected by Centro LB 960 (Berthold Technologies) with an integration time of 1 s. Normalized MetLuc activity was calculated by normalizing the MetLuc activity 24 h after transfection.

### Statistical analysis

Statistical values including the exact n and statistical test are reported in the figure legends. The levels of significance are denoted as *P < 0.05, **P < 0.01 and ***P < 0.001. N.S. means non-significant (0.05 < *P*). All statistical tests were performed by Dunnett's test, and two-tailed unpaired Student's or Welch's *t*-test using R or Excel (Microsoft). The type of *t*-test was determined by *F*-test.

## RESULTS

### Design and evaluation of synthetic circRNAs

**Figure 2.**
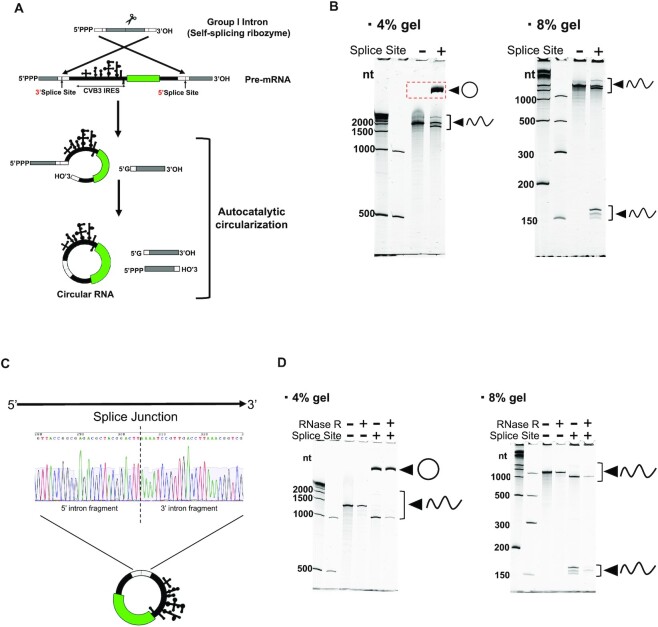
circRNA construction by permuted intron-exon (PIE) splicing. (**A**) Schematic illustration of PIE splicing. (**B**) Denaturing PAGE gel images after *in vitro* transcribed circRNA (ΔpA). DynaMarker RNA High (BioDynamics Laboratory) and Low Range ssRNA Ladder (NEB) were used as molecular weight markers. In the 4% gel, 4 bands (Splice Site +) represented as linear products are predicted to be 3805, 2060, 1910 and 1745 nucleotides (nt) in length from top to bottom. Linear product (Splice Site −) is predicted to be 2041 nt. In the 8% gel, bands at the bottom (Splice Site +) are predicted to contain linear products after splicing (the predicted length is 164 and 151 nt). (**C**) Sanger sequencing result of RT-PCR amplifying splice junction. (**D**) Denaturing PAGE gel images after RNase R treatment. In the 4% gel, four bands (RNase R −, Splice Site +) represented as linear products are predicted to be 2323, 1319, 1168 and 1004 nt in length from top to bottom. Linear product (Splice Site −) is predicted to be 1300 nt. Gel imaging experiments were repeated independently, at least twice.

Several strategies for *in vitro* mRNA cyclization have been reported (39). We chose an engineered Permuted Intron-Exon (PIE) Splicing system, which is an efficient circularization method with no enzymatic treatment for long circRNA construction (3). We designed a circRNA construct that had permuted split fragments of group I catalytic introns corresponding to *Anabaena* pre-tRNA with homology arms at both ends, and a CVB3 IRES upstream of the open reading frame (ORF) (Figure 2A). The circRNA construct with splice sites resulted in a migrated band expected as a circular RNA in denaturing PAGE (Figure 2B), which was confirmed to be circRNA through splice junction identification with ligated 5′ and 3′ splice sites (Figure 2C). Resistance to RNase R was confirmed by observing a band of the migrated product of circRNA even after RNase R treatment whereas other linear products were degraded by the treatment (Figure 2D). RNA purification from polyacrylamide gels removed most of the impurities as reported previously (3,16) (Supplementary Figure S1). These data indicate that our construct generated circRNA products as expected. Previous studies reported that such features are unique in PIE Splicing-derived circRNA (3,18).

**Figure 3.**
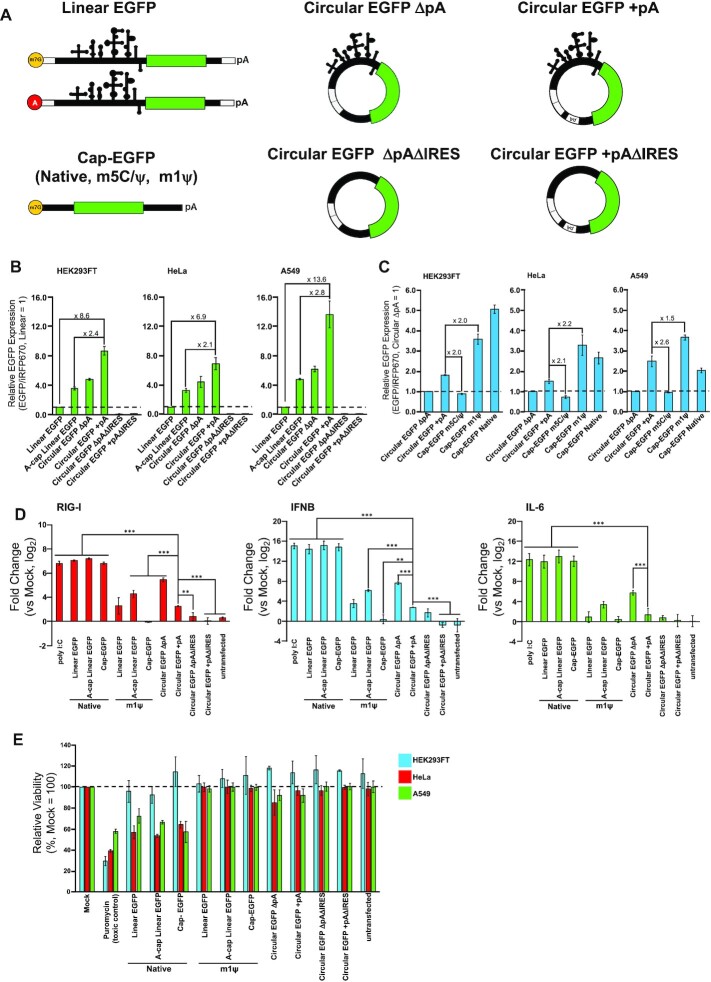
Comparison of reporter expression and immunogenicity. (**A**) Structure illustration of linear mRNAs and circRNAs used in these evaluations. All mRNAs code *EGFP* as a reporter gene. (**B**) Comparison of reporter expression between circRNAs and linear mRNAs with the same sequence component. 0.3 pmol of each reporter mRNA and transfection control, *iRFP670*-coding mRNA were transfected. (**C**) Comparison of circRNAs and linear mRNAs (Cap-EGFPs) without any other structural features corresponding to CVB3 IRES and self-splicing ribozyme. 0.3 pmol of each reporter *EGFP*-coding mRNA and transfection control, *iRFP670*-coding mRNA were transfected. (**D**) Evaluation of immune response-related genes by RT-qPCR after 24 h of transfection in A549 cells. 0.6 pmol of each mRNA was transfected. 200 ng poly I:C was transfected as a positive control. Levels of significance are denoted as **P < 0.01, ***P < 0.001 (Dunnett's test). N.S. (non-significant, *P >* 0.05) pairs were not denoted on the graph. (**E**) Evaluation of cytotoxicity by WST-1 assay was performed with a 96-well format. 0.15 pmol of each mRNA was transfected. For toxic control, the cells were cultured in 1 μg/ml (HEK293FT, HeLa) or 2 μg/ml (A549) puromycin for 1 day before the measurement. All data in this figure are presented as mean ± SD, *n* = 3.

Next, we examined the translatability of synthesized circRNAs and the effect of circularization. We compared the levels of protein production from circRNAs with those of linear mRNAs (linRNAs). Overall protein expression levels from circRNAs and linRNAs were evaluated using reporter expression (EGFP) normalized by transfection control (iRFP670). We prepared different types of RNA, including four types of circRNAs with or without 120 nucleotides of polyA (pA) sequences (circRNA + pA, circRNA ΔpA), without IRES (circRNA + pAΔIRES, circRNA ΔpAΔIRES), and three types of linRNAs (Linear EGFP, Cap-EGFP) (Figure 3A).

First, we compared EGFP expression of circRNA and linRNA with the same sequence components (UTR, CVB3 IRES, ribozyme-derived sequence, *EGFP*-coding region, pA). circRNAs with IRES showed higher expression levels than linRNAs in the three tested human cell lines (HEK293FT, HeLa, A549) whereas circRNAs ΔIRES did not activate translation (Figure 3B). Notably, circRNA + pA showed higher EGFP expression than IRES-dependent linRNAs or circRNA ΔpA (Figure 3B, Supplementary Figure S2A), confirming that the pA sequence enhances protein expression from circRNA. It has been reported that pA or pAC spacer sequences inserted to the vicinity of 5′ and 3′, or only that of 5′ intron fragments in circRNA promote translation by interaction with eukaryotic translation initiation factor (eIF4G) and polyA-binding protein (PABP), similar to the translation mechanism of linRNA (3,40,41). We assume that a similar effect was also observed when the 120-mer polyA sequence used in the tail of our linRNA was inserted internally into the circRNA (between the 3′ UTR and the 3′ intron fragment). To investigate the stabilization of RNA by cyclization, we quantified the amount of residual circRNA and linRNA by RT-qPCR. The relative levels of circRNA (Circular EGFP + pA) are higher than linRNA (Linear EGFP) at 8 and 24 h after transfection (Supplementary Figure S2B), indicating that enhanced stability by cyclization is likely to contribute to higher reporter expression.

We next compared the performance of circRNAs with conventional cap-dependent linRNAs (Cap-EGFP) with modified bases (m5C/ψ, m1ψ; modRNAs). These base modifications have been applied to synthetic mRNAs to reduce immunogenicity and improve protein expression (42–44). Although the protein expression level from circRNA + pA was lower than m1ψ or native linRNA, it showed a higher expression level than that from Cap-EGFP with m5C/ψ (Figure 3C, Supplementary Figure S2C). The results indicate that the expression level from circRNA is superior to that from linRNAs with the same sequence context and cap-dependent modRNA with m5C/ψ.

We next investigated the effect of immunogenicity of linRNAs or circRNA transfection by RT-qPCR (Figure 3D). As expected, expression levels of immune response-related genes (RIG-1, IFNB and IL-6) were upregulated after transfecting native mRNA, and the degree of upregulation was decreased when the modRNA (m1ψ) equivalent was transfected in place of its native mRNA. We observed that circRNA constructs with native bases also reduced the expression of these genes, confirming the reduced immunogenicity of circRNAs (16). CircRNA ΔpA showed similar levels of the expression of these genes compared with Acap-linRNA (m1ψ). Interestingly, circRNA + pA decreased the expression of these genes compared with circRNA ΔpA, indicating that the addition of the internal polyA sequence further reduced the immunogenicity of circRNA.

Additionally, we investigated the cytotoxic effect of the circRNAs in the three cell lines (Figure 3E). We confirmed that transfection of linRNA with native base decreased HeLa or A549 cell viability whereas that of modRNA (m1ψ) maintained normal cell viability, confirming previous observations (42–44). Our circRNAs (circRNA + pA and circRNA ΔpA, with or without IRES sequences) also maintained cell viability without cytotoxic effect. Thus, we conclude that our circRNA constructs efficiently expressed target proteins without inducing severe cell cytotoxicity and immunogenicity, which can be used for further circRNA engineering.

### Construction and evaluation of miRNA-responsive circRNA switches

**Figure 4.**
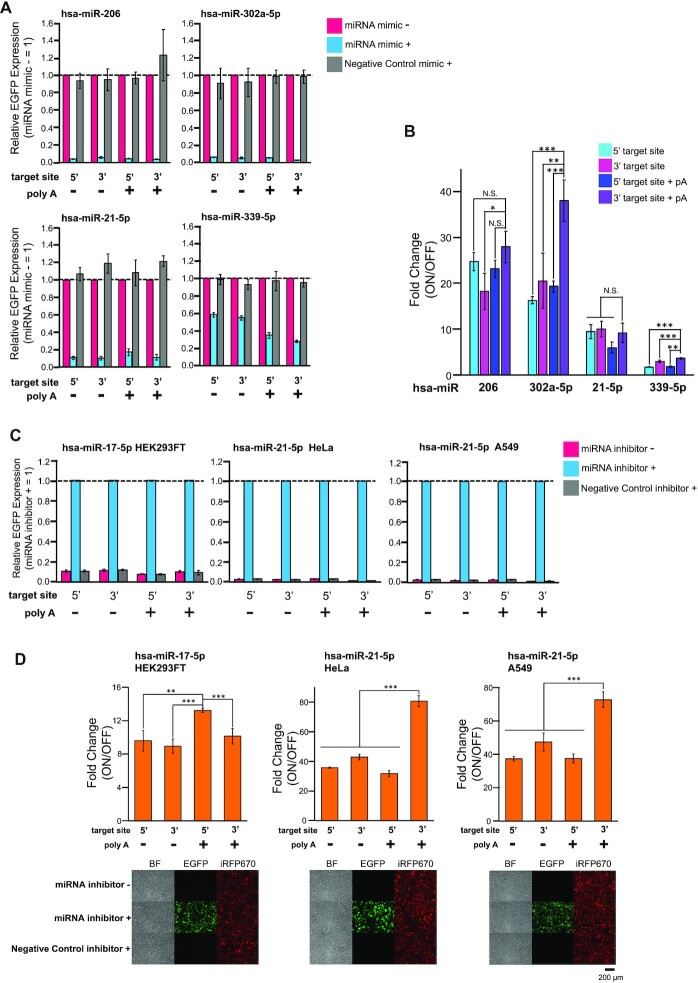
Design and evaluation of miRNA-responsive circRNA switches. (**A**) Evaluation by co-transfecting miRNA mimics in HEK293FT cells. Relative EGFP Expression was calculated by normalizing the sample without a miRNA mimic (magenta). 0.3 pmol of each reporter mRNA and transfection control mRNA were transfected. 0.25 pmol of specific miRNA mimic or Negative Control mimic was co-transfected for evaluation. (**B**) The fold-change of each miRNA-responsive circRNAs was evaluated in (A). The ON state was determined as the sample without miRNA mimic. (**C**) Endogenous miRNA detection by miRNA-responsive circRNAs in HEK293FT, HeLa and A549. Relative EGFP Expression was calculated by normalizing the sample with a specific miRNA inhibitor (cyan). 0.3 pmol of each reporter mRNA and transfection control mRNA were transfected. 1 pmol (hsa-miR-17-5p, HEK293FT), 2 pmol (hsa-miR-21–5p, HeLa) or 4 pmol (hsa-miR-21-5p, A549) of specific miRNA inhibitor or Negative Control inhibitor was co-transfected for evaluation. (**D**) The fold-change of each endogenous miRNA-responsive circRNA was evaluated in Figure 4C and fluorescent images of miRNA-responsive circRNA switches showing the best fold change. The ON state was determined as the sample with a specific miRNA inhibitor. The scale bar at the fluorescent images indicates 200 μm. Levels of significance are denoted as *P < 0.05, **P < 0.01, ***P < 0.001 (Dunnett's test). N.S. means non-significant (*P >*0.05). All data in this figure are presented as mean ± SD, *n* = 3.

Next, we designed circRNAs that contained a fully complementary anti-miR sequence at the UTRs and evaluated them by co-transfecting various miRNA mimics. We prepared four different *Homo sapiens* microRNA (hsa-miR-206, hsa-miR-302a-5p, hsa-miR-21-5p and hsa-miR-339-5p)-responsive circRNA switches by inserting anti-miR either before the CVB3 IRES (5′-insertion) or after the *EGFP*-coding sequence (3′-insertion). Twenty-four h after transfection with circRNA switches and the corresponding miRNA mimics, we analyzed EGFP expression from the circRNAs by flow cytometer and fluorescent microscopy. Notably, all designed miRNA-responsive switches repressed EGFP expression by sensing the target miRNA mimic (Figure 4A, Supplementary Figure S3A). The observed fold changes (approximately 2- to 38-fold ranges) between the ON state and OFF state depended on the target miRNA, the insertion position of anti-miR, and the presence or absence of an internal polyA sequence (Figure 4B).

We speculated that the relatively low-fold change of miR-21-5p- and miR-339-5p-responsive switches might be due to endogenous miRNA activity expressed in the HEK293FT cell (45). Thus, we added target miRNA inhibitors into the cells to block the activity of endogenous miRNA. Co-transfection with the miR-21-5p inhibitor resulted in the rescue of circRNA translation; however, this was not seen for the miR-339-5p inhibitor (Supplementary Figure S3B). This result suggests that the observed low fold change in the case of the miR-21-5p-responsive switch is due to a decrease in the ON state caused by intrinsic miR-21-5p activity. In fact, our previous study showed that the HEK293FT cell expresses endogenous miR-21-5p, although its activity is lower compared with other cancer cell lines (e.g. HeLa cells) (44,45). For the miR-339-5p-responsive switch, however, it may be caused by other factors like accessibility to the target site caused by RNA secondary structures, as predicted with CentroidFold (46) (Supplementary Figure S3C).

To investigate whether target miRNA could enhance the cleavage and degradation of miRNA-sensing circRNAs, we analyzed circRNA levels in the presence or absence of miRNA mimic by RT-qPCR. We used miR-206- and miR-302a-5p-responsive switches and the corresponding primer pairs that amplify the remaining sequence at three different regions of the circRNAs (Supplementary Figure S4A). The presence of target miRNA mimic enhanced the degradation of both circRNA switches, confirming the miRNA-mediated circRNA degradation. The surrounding region of the miRNA target site in the 3′ UTR (ORF end∼3UTR) is more susceptible to degradation than the top and middle region of ORF (ORF top, ORF middle) (Supplementary Figure S4B), suggesting that miRNA-responsive circRNA switch may undergo endonucleolytic cleavage at the fully matched miRNA target site portion and be degraded from the vicinity of the target site after the miRNA-mediated cleavage (20,29). The results also indicates that the low leakage expression observed in the OFF state of the circRNA switch (Supplementary Figure S3A, mimic+) may be due to the translation from partially degraded circRNAs.

In addition, we investigated whether the designed circRNA switches could detect endogenous miRNAs and modulate translation in the target cell. We focused on hsa-miR-17-5p in HEK293FT and hsa-miR-21-5p in HeLa and A549 because these miRNAs are efficiently expressed in each cell type (45,47). The reporter expression from the transfected switches was rescued only when it was co-transfected with a target miRNA-specific inhibitor, showing ON/OFF fold changes were approximately 12-fold (hsa-miR-17-5p) and 80-fold (hsa-miR-21-5p), with clear separation of each cell with or without the inhibitor by flow cytometry (Figure 4C, D, and Supplementary Figure S5), which was comparable to the results obtained with the miRNA mimics (Figure 4A, B, and Supplementary Figure S3A). These results indicate that miRNA-responsive circRNA switches efficiently detected target miRNAs, controlling their translation level in a cell type-specific manner.

### CVB3 IRES engineering for RBP-responsive circRNA switches

**Figure 5.**
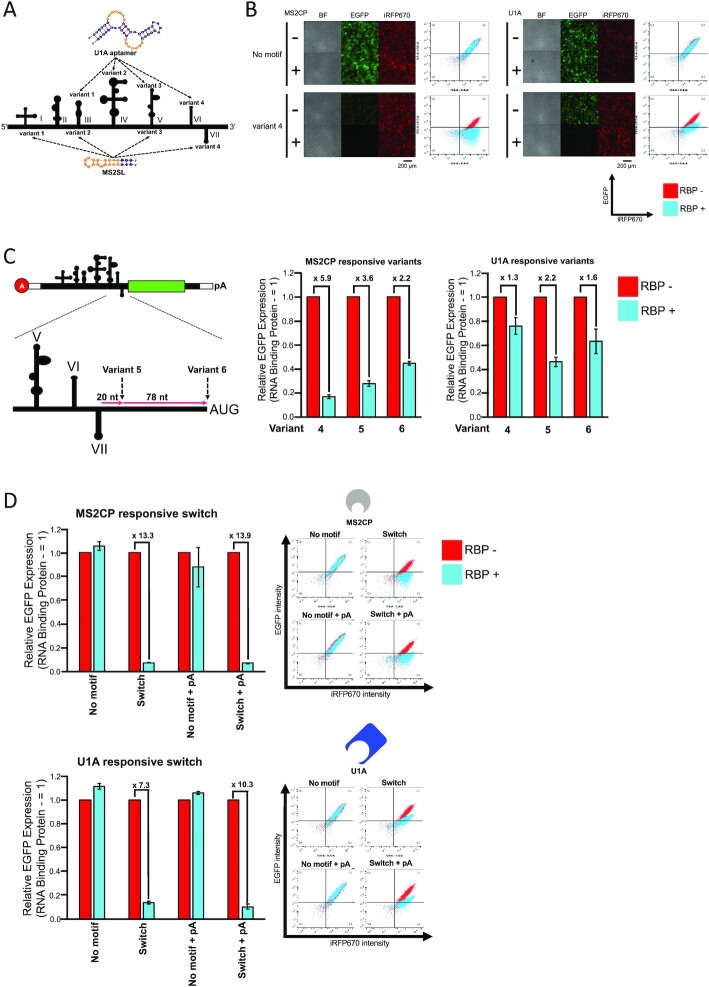
Design and evaluation of protein-responsive circRNA switches. (**A**) Investigation of effective position for the protein-binding motif (aptamer) insertion. Four different variants (variants 1–4) were designed by inserting MS2SL or U1A aptamer. Orange bases in MS2SL and U1A aptamer indicate that protein-binding region. (**B**) Fluorescent microscope images and scatter plots of flow cytometry evaluated by co-transfecting MS2CP or U1A-coding mRNA in HEK293FT. The scale bar at the fluorescent images indicates 200 μm. 0.3 pmol of each reporter mRNA and transfection control mRNA were transfected. 0.05 pmol of RBP (MS2CP or U1A)-coding mRNA was co-transfected for evaluation. (**C**) Optimization of the position for the motif-insertion. Additional variants were designed by inserting the motif at 20 nt (variant 5) or 98 nt (variant 6) downstream of the domain VII stem-loop. A-cap Linear EGFP with the variants was used for evaluation. 0.3 pmol of each reporter mRNA and transfection control mRNA were transfected. 0.05 pmol of RBP (MS2CP or U1A)-coding mRNA was co-transfected for evaluation. (**D**) Evaluation of U1A and MS2CP-responsive circRNA switches by using circRNA contexts in HEK293FT cells. 0.3 pmol of each reporter mRNA and transfection control mRNA were transfected. 0.05 pmol of MS2CP-coding mRNA or 0.15 pmol of U1A-coding mRNA was co-transfected for evaluation. All data in this figure are presented as mean ± SD, *n* = 3. Relative EGFP Expression was calculated by normalizing the sample without target protein-coding mRNA. The plots shown are representative data from three biological replicates. The vertical axis of the scatter plot shows the fluorescence intensity of EGFP, and the horizontal axis shows the fluorescence intensity of iRFP670.

We next designed protein-responsive circRNA by using two RBPs: MS2 bacteriophage coat protein (MS2CP) and spliceosome-related SNRPA (U1A) protein. To investigate the positions of protein-binding that enable IRES-dependent translation repression, we tested four CVB3 IRES variants (variants 1–4) designed by inserting protein-binding motifs (MS2SL and U1A aptamer) while referring to the secondary structure model and structural features necessary for translation initiation (32,48,49) (Figure 5A). Inserting the MS2CP or U1A-binding motif showed that only variant 4, in which the motif was inserted in domains VI (MS2CP) or VII (U1A), was able to repress translation in the presence of the target RBP, whereas other variants did not efficiently express the reporter or did not respond to target RBP (Figure 5B and Supplementary Figure S6).

From these results, we expected that motif insertion to CVB3 IRES near or downstream of the binding site for translation initiation factors, eIF4G and eIF4A (which bind to domains V–VII) (49), or the antisense region against 18S rRNA (the linker region between domains V and VI) (48), may be effective for generating the RBP-responsive circRNA switch. To optimize the insertion position, we further designed variants 5 and 6, with the inserted-motif at 20 nt or 98 nt (just upstream of the ORF) downstream of the domain VII stem-loop and compared their repression efficiencies with that of variant 4. We evaluated the protein production of CVB3 IRES variants 4–6 using A-cap linear *EGFP* mRNA which also functioned as a miRNA-responsive switch (Supplementary Figure S7). The results showed that variant 4 for MS2SL, and variant 5 for U1A aptamer were the best for translation repression in the presence of MS2CP or U1A (Figure 5C and Supplementary Figure S8). We applied these variants to evaluate their performance in circRNA to confirm the effect of the presence or absence of the internal polyA sequence on translational suppression and fold change. The translational repression observed in circRNA switch constructs was all efficient, as seen in the fold changes (7.3- to 13.9-fold ranges) between ON and OFF states in the presence or absence of MS2CP or U1A (Figure 5D). Together, these results indicates that RBP-responsive circRNA switches can be designed by engineering the regions of domains VI and VII of CVB3 IRES.

In addition, we analyzed the expression level of MS2CP and U1A by western blotting and confirmed the effective expression from these *RBP*-coding mRNAs (Supplementary Figure S9A and B). Although we detected the expression of endogenous U1A, an apparent increase in expression was observed in cells transfected with *U1A*-coding mRNA. We next investigated whether endogenous U1A protein may affect the ON state of U1A-responsive circRNA, even in the absence of *U1A*-coding mRNA. We performed a knockdown assay of endogenous U1A by shRNAs used in the previous study (30). As expected, the endogenous U1A expression was repressed by U1A-targeted shRNA (Supplementary Figure S9C, left), but the knockdown of endogenous U1A did not increase the reporter EGFP expression from the U1A-responsive circRNA (Supplementary Figure S9C, right), suggesting that endogenous U1A does not affect the performance of the circRNA switch under the condition.

### Construction of synthetic circRNA circuits and their extended driving

**Figure 6.**
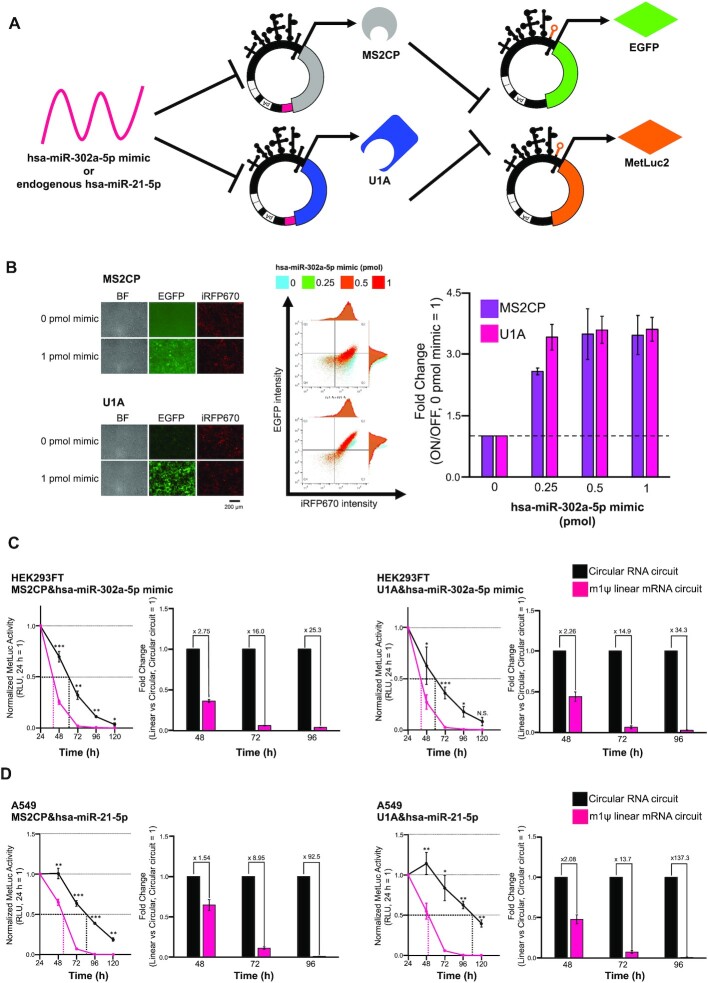
circRNA-based gene circuits that activate translation by detecting miRNA. (**A**) Schematic illustration of miRNA-responsive ON circuit. MS2CP or U1A is encoded on the first miRNA-responsive circRNA switch. The reporter gene (*EGFP* or *MetLuc2*) is encoded on the second protein-responsive circRNA switch. (**B**) Fluorescent images, scatter plots of a flow cytometer, and titration results of ON circuits with miR-302a-5p mimic in HEK293FT cells. The scale bar at the fluorescent images indicates 200 μm. 0.3 pmol of RBP (MS2CP or U1A)-coding mRNA, reporter mRNA and transfection control mRNA were transfected. 0.25, 0.5 or 1 pmol of hsa-miR-302a-5p mimic was co-transfected for evaluation. The plots shown are representative data from three biological replicates. The vertical axis of the scatter plot shows the fluorescence intensity of EGFP, and the horizontal axis shows the fluorescence intensity of iRFP670. (**C**) Evaluation of circRNA circuit persistence with a miR-302a-5p mimic in HEK293FT cells. 45 fmol of RBP (MS2CP or U1A)-coding mRNA and reporter mRNA were transfected. 1 pmol of hsa-miR-302a-5p mimic was co-transfected to activate the circuits. (**D**) Evaluation of circRNA circuit persistence with endogenous miR-21–5p in A549 cells. 45 fmol of RBP (MS2CP or U1A)-coding mRNA and reporter mRNA were transfected. Levels of significance are denoted as *P < 0.05, **P < 0.01, ***P < 0.001 (two-tailed unpaired Student's or Welch's *t*-test determined by *F*-test). N.S. means non-significant (*P >*0.05). All data in this figure are presented as mean ± SD, *n* = 3.

Finally, we investigated whether circRNAs could improve the driving time of synthetic RNA circuits composed of both the miRNA-responsive and RBP-responsive switches. When multiple switches are available, it is possible to construct synthetic genetic circuits by designing the output from one switch to be the input of another switch (23) (Figures 1B and 6A).

We first confirmed the enhanced stability and gene expression persistence of the designed circRNA. We compared gene expressions of Metridia luciferase (*MetLuc2*)-coding circRNA (Circular MetLuc2 + pA), with conventional cap-dependent linRNAs with modified bases (m5C/ψ, m1ψ) using the three human cell lines (Supplementary Figure S10). The circRNA constructs showed better expression persistence than the linRNAs with enhanced expression duration by modified bases (42), consistent with the previous reports (3,16).

We next designed a miRNA-responsive ON system by using two circRNA switches, miRNA (miR-302a-5p)-responsive circRNA that produced RBP (MS2CP or U1A) and the RBP-responsive circRNA that produced EGFP. The designed circRNA circuits using either MS2CP or U1A produced EGFP only in the presence of miR-302a-5p mimic, functioning as an ON switch by detecting target miRNA (Figure 6B, left). The fold changes between the ON and OFF states of these miRNA-responsive circuits were approximately 3.5-folds in the presence of 1 pmol of miR-302a-5p mimic (Figure 6B, right).

We also compared the performance of the persistence of circRNA circuits with linear mRNA circuits used in previous studies (31,44,50), by using the MetLuc2 reporter. We tested them using two cell lines, HEK293FT and A549, to detect exogenous miR-302a-5p mimic or endogenous miR-21-5p, respectively. In both cases, the circRNA circuit showed better performance of duration than the linear RNA circuit with m1ψ (1.5- to 137-fold duration) after approximately 96 h of RNA transfection (Figure 6C and D), indicating that our circRNA circuits improved the expression persistence of synthetic mRNA circuits.

## DISCUSSION

In this study, we provided the principle for designing miRNA and protein-responsive circRNA switches with controlled expression by detecting intracellular conditions in mammalian cells. No previous studies have shown synthetic circRNAs with regulatory functions. In particular, RBP-mediated translational repression of circRNA was not observed in either the synthetic or endogenous system. Among RBP-responsive CVB3 IRES variants, only those in which the protein-binding motif was inserted into domain VI or VII, downstream of the binding site for translation initiation factors and antisense region against 18S rRNA, were functional (variant4, Figure 5A, B and Supplementary Figure S6). A previous report with a cap-dependent system suggested that inhibition of the translation initiation step by RBP binding plays a central role in repression (51,52). Our results indicate that the protein-responsive switch can also be designed for cap-independent translation by inserting motifs at a position that efficiently inhibits the assembly of the translation initiation factors or subsequent ribosome scanning in the IRES. The suitable position for the motif insertion seems to be located at the downstream region of domain VI in the CVB3 IRES, which was confirmed by experiments using variants 1–6 (Figure 5B, C and Supplementary Figure S6). Further investigations will be required to study whether similar results could be obtained in other IRES-inserted circRNA switches in the future. For miRNA-responsive circRNA switches, it was noted that they were able to detect endogenous miRNAs in the target cell (hsa-miR-17-5p in HEK 293FT, hsa-miR-21-5p in HeLa and A549 cells), making it possible to regulate circRNA translation in a cell-type-specific manner (Figures 4D and 6D). Such cell-type-specific regulation would reduce potential side effects for future mRNA therapeutics.

Due to the superior stability and expression persistence of circRNAs, RNA-based gene circuits constructed from circRNA switches were more durable than those composed of linear modRNAs (Figure 6 and Supplementary Figure S10). Thus, our circRNA switches may solve the previously reported issue of a shorter half-life for modRNA-based circuits (23). Durable RNA-based gene circuits have also been realized using replicon vectors (23). Our circRNA-based circuits may have several advantages over such replicon-based circuits, such as a more compact size, no unexpected self-replication, easier handling, and higher transfection efficiency with lipid nanoparticle-based systems (53). Direct comparison of the performances between circRNA- and replicon-based circuits will be important in future studies.

Improving the performance of circRNA switches should also be addressed in future studies. In the comparison using our linear mRNA switch systems (31,44,50) (linear switches) with HEK293FT, the performance of miRNA-responsive circRNA switches was comparable (m1ψ) or superior (m5C/ψ) to that of base-substituted linear switches, except for the one affected by endogenous miR-21-5p activity (Supplementary Figure S11A), whereas protein-responsive circRNA switches performed less efficiently than m1ψ-substituted linear mRNA switches (Supplementary Figure S11B). In the linear switches, it has been shown that incorporation of m1ψ to mRNA could enhance its sensitivity to several miRNAs and RBPs, contributing to better performance (44,54). However, CVB3 IRES with the base modification (e.g. m5C/ψ or m1ψ) disrupted protein expression (16) (Supplementary Figure S12). Therefore, to develop chemically modified IRES-dependent circRNA switches, we need to engineer functional IRES with modified bases through rational engineering or directed evolution approaches.

RNA sequence and structure engineering is an alternative method to achieve a superior ON and OFF state without modified-base substitution for circRNA switches. Previous research on linear mRNA engineering has achieved improvements through codon optimization of the ORF (55), more stable UTRs (56,57), and engineering for protein or miRNA binding sequences (30,31,58). These approaches may be adaptable to circRNA-based systems, although optimization would be required in some cases. For example, in miRNA-responsive circRNA switches, they likely preferred 3′ insertion, rather than 5′, with internal polyA-containing constructs (Figure 4B), contrary to our previous observation with the linear mRNA system (22). We expected this tendency was caused by the rigid structure derived from CVB3 IRES on the 5′ side, which may affect miRNA-mediated circRNA degradation, although the miR-17-5p-responsive circRNA switch showed the best fold-change in 5′ target site insertion (Figure 4D). In addition, contrary to the previous report of increased sensitivity with increasing copy number of miR-302a-5p target sites in the linear mRNA switch (58), a single copy insertion in the 3′ UTR showed the best fold-change between ON and OFF states for miR-302a-5p responsive circRNA switch (Supplementary Figure S13, right). In the case of the miR-206-responsive circRNA switch, 2-copy insertion into both the 5′ and 3′ UTRs (2 × 2 insertion, 4 copies total) showed the best fold-change (Supplementary Figure S13, left). These observations suggest that sequence dependency in circRNA systems is more pronounced than in linRNA systems, and that target miRNA-specific optimization steps will be required to maximize performance for circRNA switches (59). Recently, Chen et al. reported the sequence elements that enhance circRNA performance (41). The identified various accessory parts and engineered IRES with enhanced translation would improve the performance of circRNA switch (e.g. enhanced ON state) (44).

Recently, it has been reported that exogenous circRNAs cause an immune response, while contrasting reports suggested that they are less immunogenic (15–17). RT-qPCR analysis for immune response-related genes (RIG-I, IFNB, IL-6) showed that our circRNA is less immunogenic than linear mRNAs with native bases, whereas a more intense immune response was observed compared with chemically modified linear mRNAs, especially linear mRNAs with the commonly used Cap-EGFP structure (Figure 3D). Notably, lower immunogenicity was observed in circRNAs with internal polyA sequences compared to those without an internal polyA sequence. A previous report by Liu *et al.* suggested that the RNA duplex constructed by ribozymes and CVB3 IRES causes circRNA immunogenicity (18). Our observations indicate that immunogenicity caused by such structures in circRNAs may be reduced by internal polyA-120 sequence longer than constructs in previous reports (3,16,41). Thus, the stronger expression levels observed with long polyA insertion may be a synergistic effect of the recruitment of eIF4G by PABP (3,40,41) and the reduction of immunogenicity. We expect that further investigation is needed to determine the composition and length of the polyA sequence to achieve enhanced translation and reduced immunogenicity. In terms of cytotoxicity, our circRNAs showed better cell viability than native linear mRNAs, which was comparable to those of chemically modified ones (Figure 3E), indicating that the induction level of these immune response-related genes by our circRNA does not affect cells. However, future *in vivo* evaluation is required to determine how these immunogenicity differences will affect actual therapeutic applications.

In conclusion, our circRNA switches and circuits provide new insights into the engineering of circRNA, which is still underdeveloped compared to linear synthetic mRNA. We believe that synthetic circRNA devices with translation regulations process the broad potential for synthetic biology, mRNA-based therapies, and cellular engineering.

## DATA AVAILABILITY

The authors declare that all data supporting the findings of this study are available within the paper and its supplementary information files. All raw data for each graph are shown in the Supplementary Table S3. Requests for materials should be made to the corresponding author. All plasmids and mRNAs generated in this study are available upon request.

## Supplementary Material

gkac1252_Supplemental_Files
