# Supplementary material for: Synthetic circular RNA switches and circuits that control protein expression in mammalian cells
Source: Nucleic Acids Res. 2023 Jan 16;51(4):e24. doi: 10.1093/nar/gkac1252 (PMC9976894; doi:10.1093/nar/gkac1252)
Supplement: gkac1252_Supplemental_Files [file gkac1252_supplemental_files.zip › SK_Supplemental data_circRNA switch_1101_5.pdf]

Synthetic circular RNA switches and circuits that control protein expression in mammalian cells

Shigetoshi Kameda[1,2], Hirohisa Ohno[1], Hirohide Saito[1] *

[1] Department of Life Science Frontiers, Center for iPS Cell Research and Application (CiRA), Kyoto 606-8507, Japan.
[2] Graduate School of Medicine, Kyoto University, Kyoto 606-8501, Japan.

*Correspondence and requests for materials should be addressed to H.S. (email: hirohide.saito@cira.kyoto-u.ac.jp)

This file includes the following:

Supplementary Tables (S1, S2)
Supplementary Figures (S1-S13)
Supplementary Sequences
Supplementary References

Supplementary Tables

Supplementary Table S1. Transfection overview

Figure	Transfected-mRNA(s) (/well)	miRNA mimic/inhibitor (/well)	Cell line and number of seeded cells (cells/well)	Lipofectamine MessengerMAX (µl/well)
3B	0.3 pmol Cap-iRFP670 (m5C/Ψ)<br>0.3 pmol Linear EGFP (m7G cap, A-cap), Circular EGFP (ΔpA, +pA) or Ciruclar EGFP ΔIRES (ΔpA, +pA)	-	HEK293FT, A549 : 1.0 x 10^5<br>HeLa : 0.5 x 10^5	1
3C	0.3 pmol Cap-iRFP670 (m5C/Ψ)<br>0.3 pmol Circular EGFP ΔpA, Circular EGFP +pA or Cap-EGFP (Native, m5C/Ψ, m1Ψ)	-	HEK293FT, A549 : 1.0 x 10^5<br>HeLa : 0.5 x 10^5	1
3D	0.6 pmol Linear EGFP (m7G-cap, A-cap, Native, m1Ψ), Cirular EGFP (ΔpA, +pA) Circular EGFP ΔIRES (ΔpA, +pA) or Cap-EGFP (Native, m1Ψ)	-	A549 : 1.0 x 10^5	1
3E	0.15 pmol Linear EGFP (m7G-cap, A-cap, Native, m1Ψ), Cirular EGFP (ΔpA, pA+) Circular EGFP ΔIRES (ΔpA, pA+) or Cap-EGFP (Native, m1Ψ)	-	HEK293FT, A549 : 2.0 x 10^4<br>HeLa : 1.0 x 10^4	0.2
4A・B	0.3 pmol Cap-iRFP670 (m5C/Ψ)<br>0.3 pmol 5' or 3'T miR Circular EGFP ΔpA or 5' or 3'T miR Cirular EGFP +pA<br>5' or 3'T miR Linear EGFP	0.25 pmol miRNA mimic or Negative Control mimic	HEK293FT : 1.0 x 10^5	1
4C・D	0.3 pmol Cap-iRFP670 (m5C/Ψ)<br>0.3 pmol 5' or 3'T miR Circular EGFP ΔpA or 5' or 3'T miR Cirular EGFP +pA	HEK293FT : 1 pmol miR-17-5p inhibitor or Negative Control inhibitor<br>HeLa : 2 pmol miR-21-5p inhibitor or Negative Control inhibitor<br>A549 : 4 pmol miR-21-5p inhibitor or Negative Control inhibitor	HEK293FT, A549 : 1.0 x 10^5<br>HeLa : 0.5 x 10^5	1
5B	0.3 pmol Cap-iRFP670 (m5C/Ψ)<br>MS2CP : 0.3 pmol Cirular EGFP or MS2CP responsive Circular EGFP variant1～4, 0.05 pmol Cap-MS2CP (m1Ψ)<br>U1A : 0.3 pmol Cirular EGFP or U1A responsive Circular EGFP variant1～4, 0.05 pmol Cap-U1A (m1Ψ)	-	HEK293FT : 1.0 x 10^5	1
5C	0.3 pmol Cap-iRFP670 (m5C/Ψ)<br>MS2CP : 0.3 pmol MS2CP responsive linear EGFP variant4～6 (A-cap), 0.05 pmol Cap-MS2CP (m1Ψ)<br>U1A : 0.3 pmol U1A responsive linear EGFP variant4～6 (A-cap), 0.05 pmol Cap-U1A (m1Ψ)	-	HEK293FT : 1.0 x 10^5	1
5D	0.3 pmol Cap-iRFP670 (m5C/Ψ)<br>MS2CP : 0.3 pmol Cirular EGFP (ΔpA or pA+) or MS2CP responsive Circular EGFP variant 4 (ΔpA or pA+), 0.05 pmol Cap-MS2CP (m1Ψ)<br>U1A : 0.3 pmol Cirular EGFP (ΔpA or pA+) or U1A responsive Circular EGFP variant 5 (ΔpA or pA+), 0.15 pmol Cap-U1A (m1Ψ)	-	HEK293FT : 1.0 x 10^5	1
6B	0.3 pmol Cap-iRFP670 (m5C/Ψ)<br>MS2CP : 0.3 pmol MS2CP responsive Circular EGFP variant4 +pA, 0.3 pmol 3'T302a-5p Circular MS2CP +pA<br>U1A : 0.3 pmol U1A responsive Circular EGFP variant5 +pA, 0.3 pmol 3'T302a-5p Circular U1A +pA	0, 0.25, 0.5 1 pmol miR-302a-5p mimic	HEK293FT : 1.0 x 10^5	1
6C	MS2CP : 45 fmol MS2CP responsive Circular MetLuc2 variant4 + pA, 45 fmol 3'T302a-5p Circular MS2CP +pA<br>U1A : 45 fmol U1A responsive Circular EGFP variant5 +pA, 45 fmol 3'T302a-5p Circular U1A + pA	1 pmol miR-302a-5p mimic	HEK293FT : 1.5 x 10^4	1
6D	MS2CP : 45 fmol MS2CP responsive Circular MetLuc2 variant4 +pA, 45 fmol 3'T21-5p Circular MS2CP +pA<br>U1A : 45 fmol U1A responsive Circular EGFP variant5 +pA, 45 fmol 3'T21-5p Circular U1A +pA	-	A549 : 1.5 x 10^4	1

Figure	Transfected-mRNA(s) (/well)	miRNA mimic/inhibitor (/well)	Cell line and number of seeded cells (cells/well)	Lipofectamine MessengerMAX (μl/well)
S2B	0.3 pmol Cap-iRFP670 (m5C/Ψ)<br>0.3 pmol Linear EGFP (m7G cap) or Circular EGFP (+pA)	-	HEK293FT : 1.0 x 10^5	1
S3B	0.3 pmol Cap-iRFP670 (m5C/Ψ)<br>0.3 pmol 5' or 3'T miR Circular EGFP or 5' or 3'T miR Cirular EGFP +pA	0.25 pmol miRNA mimic, Negative control mimic, miRNA inhibitor or Negative Control inhibitor	HEK293FT : 1.0 x 10^5	1
S4B	0.3 pmol Cap-iRFP670 (m5C/Ψ)<br>0.3 pmol 5' or 3'T miR Cirular EGFP +pA	0.25 pmol miRNA mimic or Negative Control mimic	HEK293FT : 1.0 x 10^5	1
S7	0.3 pmol Cap-iRFP670 (m5C/Ψ)<br>0.3 pmol 5' or 3'T miR Linear EGFP or 5' or 3'T miR Circular EGFP ΔpA or 5' or 3'T miR Cirular EGFP +pA	0.25 pmol miRNA mimic	HEK293FT : 1.0 x 10^5	1
S9A・B	0.3 pmol Cap-iRFP670 (m5C/Ψ)<br>0.3 pmol Circular EGFP +pA<br>0.05 pmol Cap-MS2CP (m1Ψ) or 0.15 pmol Cap-U1A (m1Ψ)	-	HEK293FT : 1.0 x 10^5	1
S9C	0.3 pmol Cap-iRFP670 (m5C/Ψ)<br>0.3 pmol U1A responsive Circular EGFP variant5 +pA<br>1, 2, 4, pmol shRNA (U1A or control)	-	HEK293FT : 1.0 x 10^5	1
S10B	45 fmol Circular MetLuc2 +pA or Cap-MetLuc2 (m5C/Ψ, m1Ψ)	-	HEK293FT, A549 : 1.5 x 10^4<br>HeLa : 0.5 x 10^4	1
S11A	0.3 pmol Cap-iRFP670 (m5C/Ψ)<br>0.3 pmol 3'T miR Cirular EGFP +pA or linear miRNA-responsive switch (m5C/Ψ, m1Ψ)	0.25 pmol miRNA mimic	HEK293FT : 1.0 x 10^5	1
S11B	0.3 pmol Cap-iRFP670 (m5C/Ψ)<br>MS2CP : 0.3 pmol MS2CP responsive Circular EGFP variant4 +pA or 0.3 pmol EGFP linear switch (m5C/Ψ,m1Ψ), 0.05 pmol Cap-MS2CP (m1Ψ)<br>U1A : 0.3 pmol U1A responsive Circular EGFP variant5 + pA or 0.3 pmol EGFP linear switch mRNA (Native, m5C/Ψ or m1Ψ), 0.15 pmol Cap-U1A (m1Ψ)	-	HEK293FT : 1.0 x 10^5	1
S12	0.3 pmol Cap-iRFP670 (m5C/Ψ)<br>0.3 pmol Linear EGFP (Native, m5C/Ψ, m1Ψ)	-	HEK293FT : 1.0 x 10^5	1
S13	0.3 pmol Cap-iRFP670 (m5C/Ψ)<br>0.3 pmol 3'T, 4x 5'T, 4x3'T or 2x2 5&3'T miR Circular EGFP +pA	0.125, 0.25. 0.5 pmol miRNA mimic	HEK293FT : 1.0 x 10^5	1

All experiments were performed in 24-well format, except for Figure 3E (WST-1 assay), which was performed in 96-well format.

Supplementary Table S2. Primers used in RT-qPCR analysis.

Target gene	Primers (5'-3')	Amplicon length (bp)	Supplementary Reference
ATP5B	fwd : CAGCATTTGGGTGAGAGCAC<br>rev : TCTGCCCAAAGTCTCAGGAC	129	(S1)
RIG-I	fwd : GTTGTCCCCATGCTGTTCTT<br>rev : GCAAGTCTTACATGGCAGCA	124	(S2)
IFN-β	fwd : CTCTCCTGTTGTGCTTCTCC<br>rev : GTCAAAGTTCATCCTGTCCTTG	152	(S3)
IL-6	fwd : AGCCACTCACCTCTTCAGAAC<br>rev : GCCTCTTTGCTGCTTTCACAC	119	(S4)
EGFP ORF top	fwd : CCTGAAGTTCATCTGCACCAC<br>rev : GCATGGCGGACTTGAAGAAG	137	this paper
EGFP ORF middle	fwd : GACGACGGCAACTACAAGAC<br>rev : GTTGTACTCCAGCTTGTGCCC	129	this paper
EGFP ORF～3'UTR (miR-206)	fwd : TGCATCTCGAGTGATAGCCAC<br>rev : CAAAGACCAAGAGGTACAGGTG	114	this paper
EGFP ORF～3'UTR (miR-302a-5p)	fwd : GCATCTCGAGTGATAGAGCAAG<br>rev : CAAAGACCAAGAGGTACAGGTG	114	this paper

Supplementary Figures

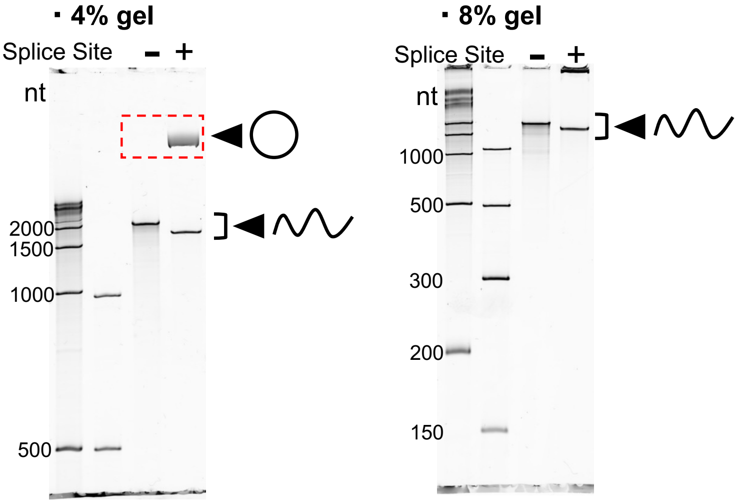

Figure S1. Denaturing polyacrylamide gel images for RNAs after the gel purification. DynaMarker RNA High (BioDynamics Laboratory) and Low Range ssRNA Ladder (NEB) were used as molecular weight markers. circRNA band is shown by red dotted rectangle. While only a minor contamination of nicked circRNA product was seen, which was also observed in the previous reports (3, 16). The gel images are representative data from at least two independent experiments.

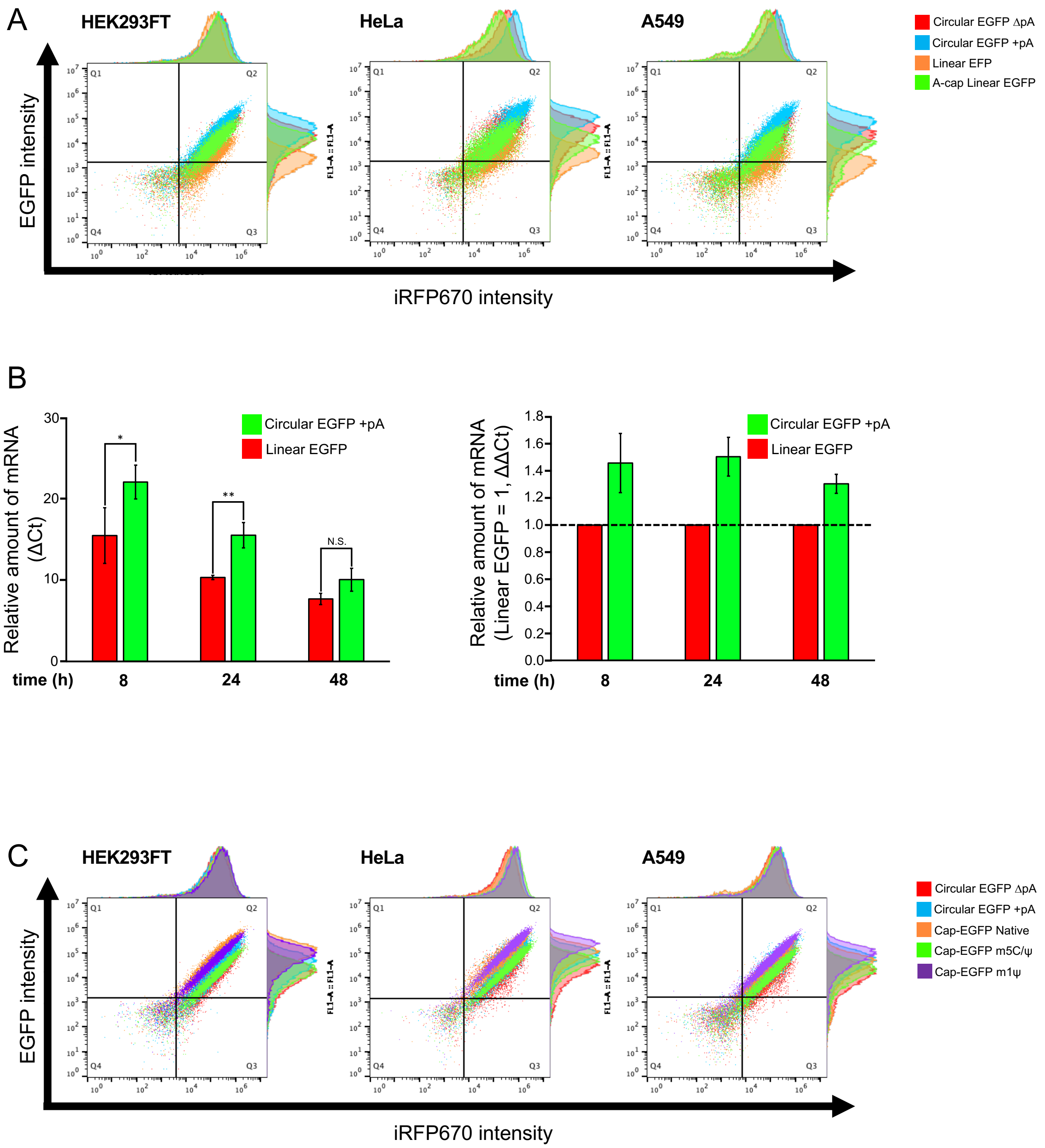

Figure S2. (A) Scatter plots generated from flow cytometry analysis in Figure 3B. (B) Analysis of the relative amount of circRNA and linRNA by RT-qPCR. The residual RNA level was compared between Linear EGFP and Circular EGFP +pA transfection after 8, 24 and 48 h in HEK293FT cells. Target mRNA quantities were normalized by ATP5B mRNA. Levels of significance are denoted as *P < 0.05, **P < 0.01 (two-tailed unpaired Student's or Welch's t-test determined by F-test). N.S. means non-significant (P > 0.05).

(C) Scatter plots generated from flow cytometry analysis in Figure 3C. All data in this figure are presented as mean ± SD, n = 3. The plots shown are representative data from three biological replicates. The vertical axis of the scatter plot shows the fluorescence intensity of EGFP, and the horizontal axis shows the fluorescence intensity of iRFP670.

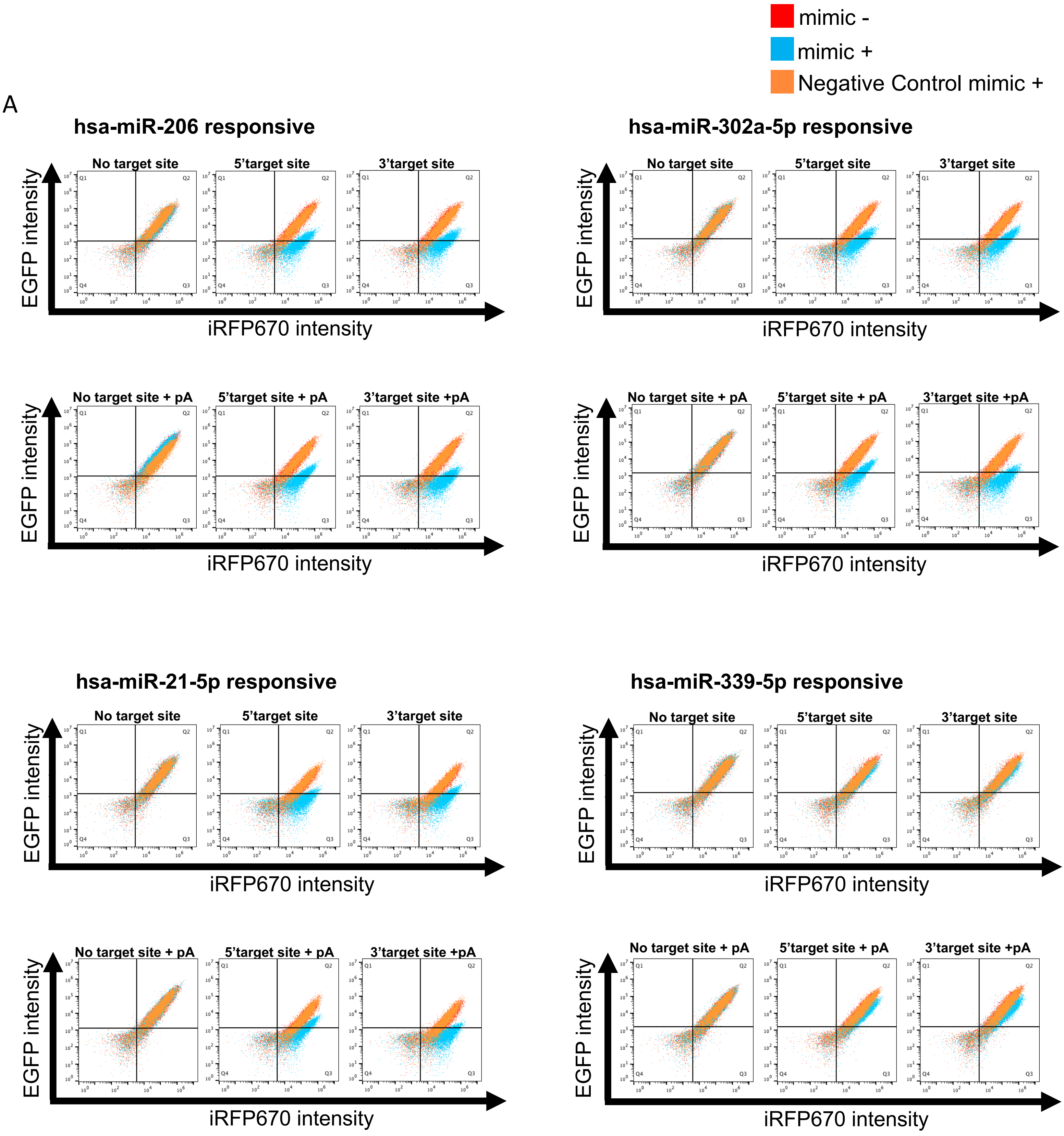
mimic -
mimic +
Negative Control mimic +
A
hsa-miR-206 responsive
No target site
5’target site
3’target site
No target site + pA
5’target site + pA
3’target site +pA
hsa-miR-302a-5p responsive
No target site
5’target site
3’target site
No target site + pA
5’target site + pA
3’target site +pA
hsa-miR-21-5p responsive
No target site
5’target site
3’target site
No target site + pA
5’target site + pA
3’target site +pA
hsa-miR-339-5p responsive
No target site
5’target site
3’target site
No target site + pA
5’target site + pA
3’target site +pA
EGFP intensity
iRFP670 intensity

B

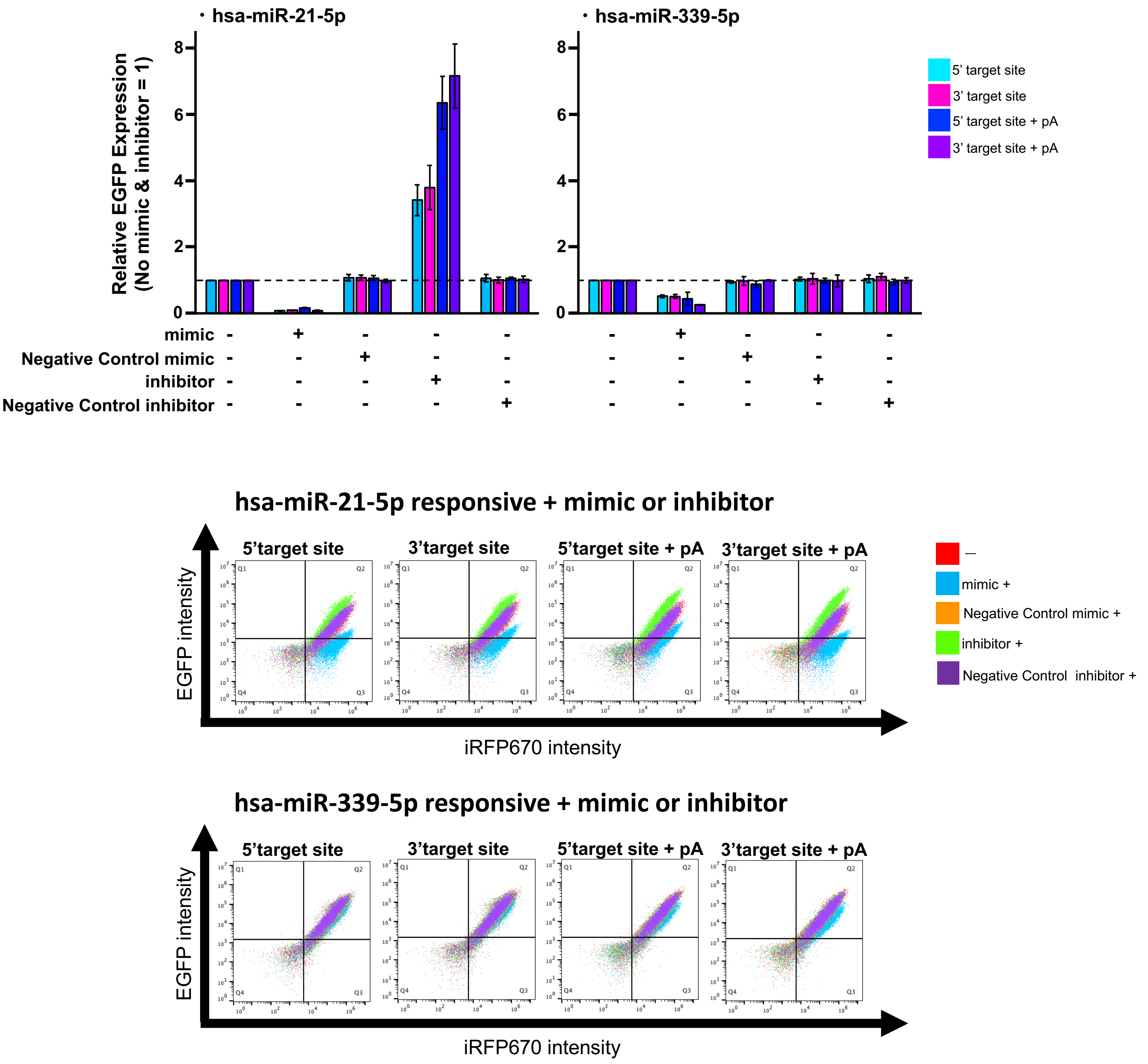

· hsa-miR-21-5p
· hsa-miR-339-5p
Relative EGFP Expression
(No mimic & inhibitor = 1)
0
2
4
6
8
mimic
Negative Control mimic
inhibitor
Negative Control inhibitor
5' target site
3' target site
5' target site + pA
3' target site + pA
hsa-miR-21-5p responsive + mimic or inhibitor
5'target site
3'target site
5'target site + pA
3'target site + pA
EGFP intensity
iRFP670 intensity
–
mimic +
Negative Control mimic +
inhibitor +
Negative Control inhibitor +
hsa-miR-339-5p responsive + mimic or inhibitor

C

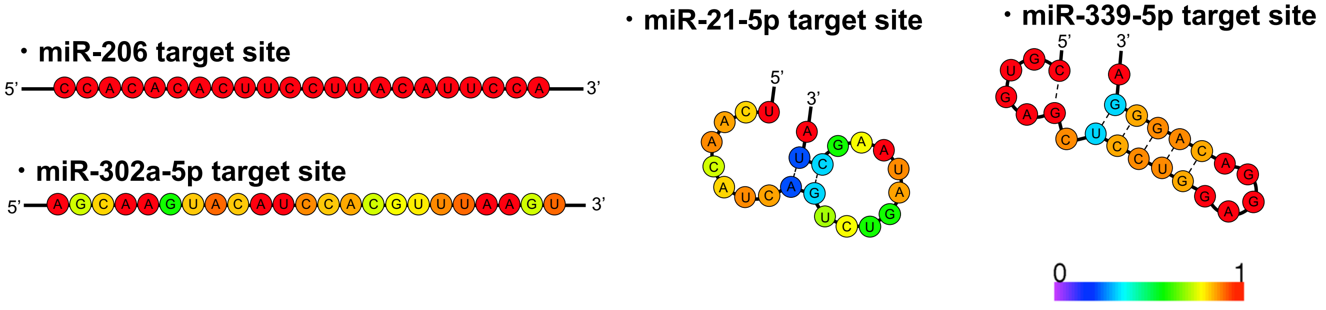

· miR-206 target site
5' CCACACACUUCCUUACAUUCCA 3'
· miR-302a-5p target site
5' AGCAAGUACAUCCACGUUUAAGU 3'
· miR-21-5p target site
· miR-339-5p target site
5'
3'
0
1

Figure S3. (A) Scatter plots generated from flow cytometry analysis in Figure 4A and 4B. (B) Evaluation of miR-21-5p- or miR-339-5p-responsive circRNA switches with co-transfecting miRNA mimic or inhibitor. (C) Secondary structure predictions of miRNA target sites used in Figure 4A and 4B with the base-pairing probabilities shown in color. Base-pairing probabilities were calculated by CentroidFold software (S5). The red pairs have a high probability of forming, green pairs have a medium probability of forming, and blue pairs have a low probability of forming. The normalized scale showing zero to one probability is shown on the lower right. All data in this figure are presented as mean ± SD, n = 3. The plots shown are representative data from three biological replicates. The vertical axis of the scatter plot shows the fluorescence intensity of EGFP, and the horizontal axis shows the fluorescence intensity of iRFP670.

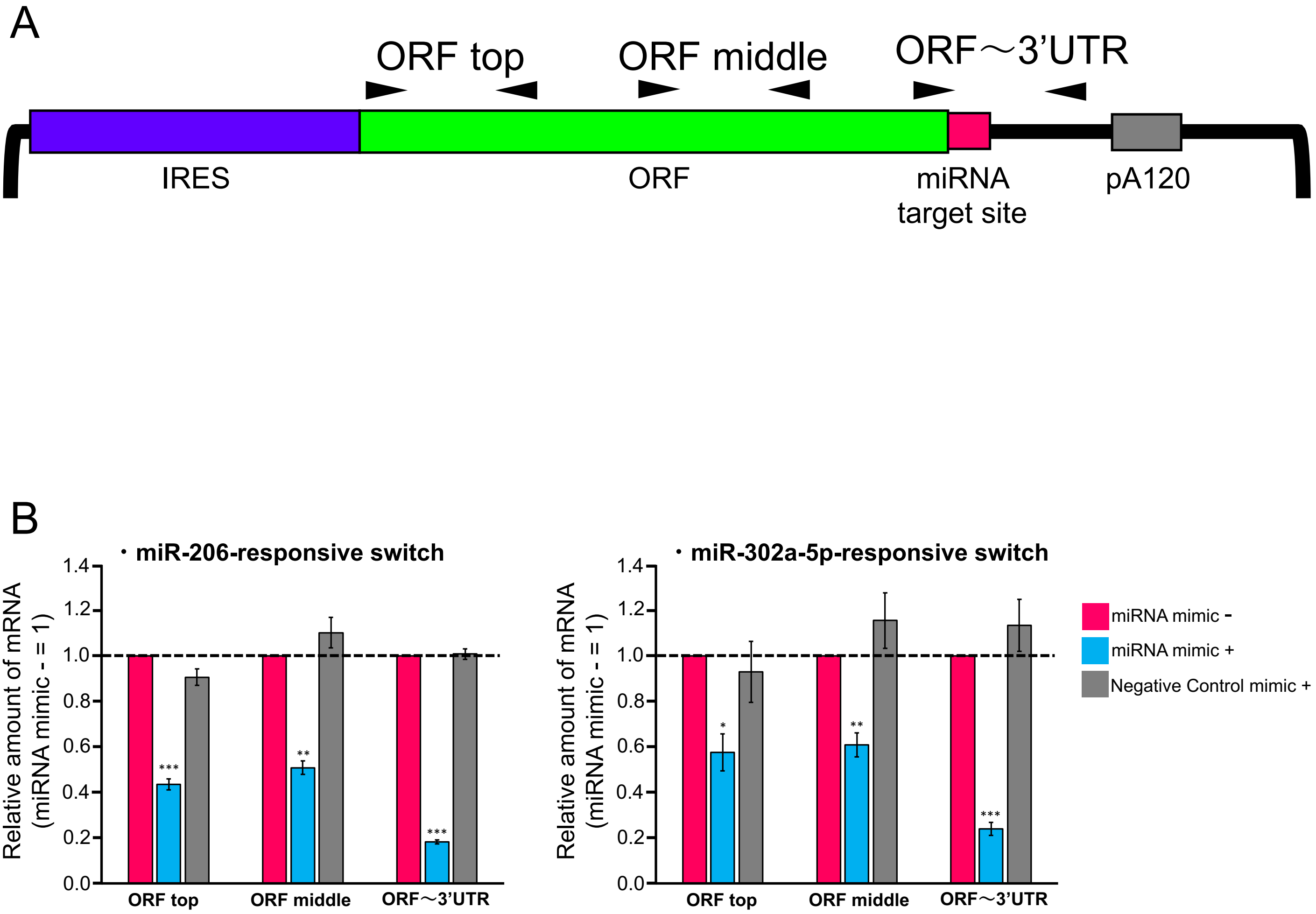

Figure S4. (A) Schematic illustrations of primer pairs on miRNA-responsive circRNA switches. Three different regions were amplified by each primer pair. (B) Evaluation of mRNA amount by RT-qPCR after 24 h of transfection in HEK293FT cells. The relative mRNA amount was calculated by normalizing the sample without a miRNA mimic (magenta). Target mRNA quantities were normalized by ATP5B mRNA. Levels of significance are denoted as *P < 0.05, **P < 0.01, ***P < 0.001 (two-tailed unpaired Student's or Welch's t-test determined by F-test, mimic + vs Negative Control mimic +). N.S. means non-significant (P > 0.05). All data in this figure are presented as mean ± SD, n = 3.

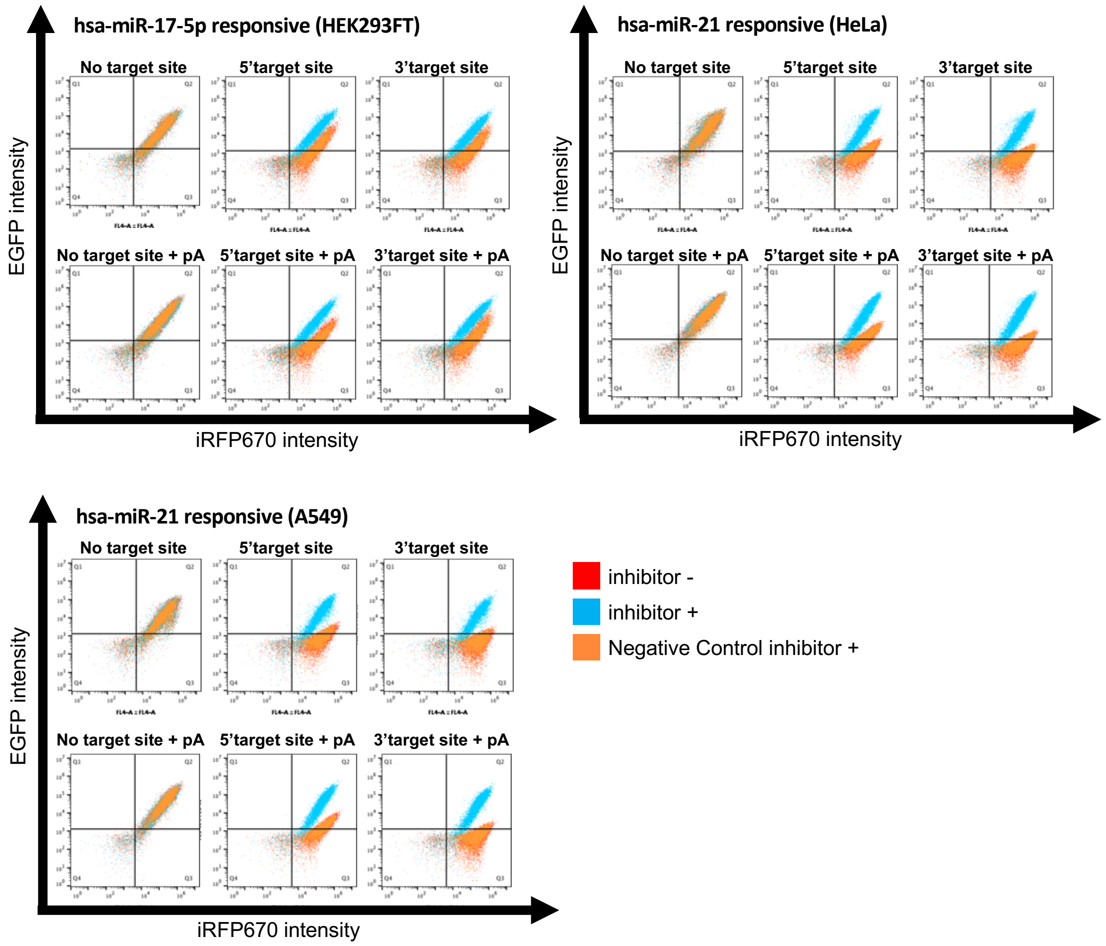

Figure S5. Scatter plots generated from flow cytometry analysis in Figure 4C, 4D. The plots shown are representative data from three biological replicates. The vertical axis of the scatter plot shows the fluorescence intensity of EGFP, and the horizontal axis shows the fluorescence intensity of iRFP670.

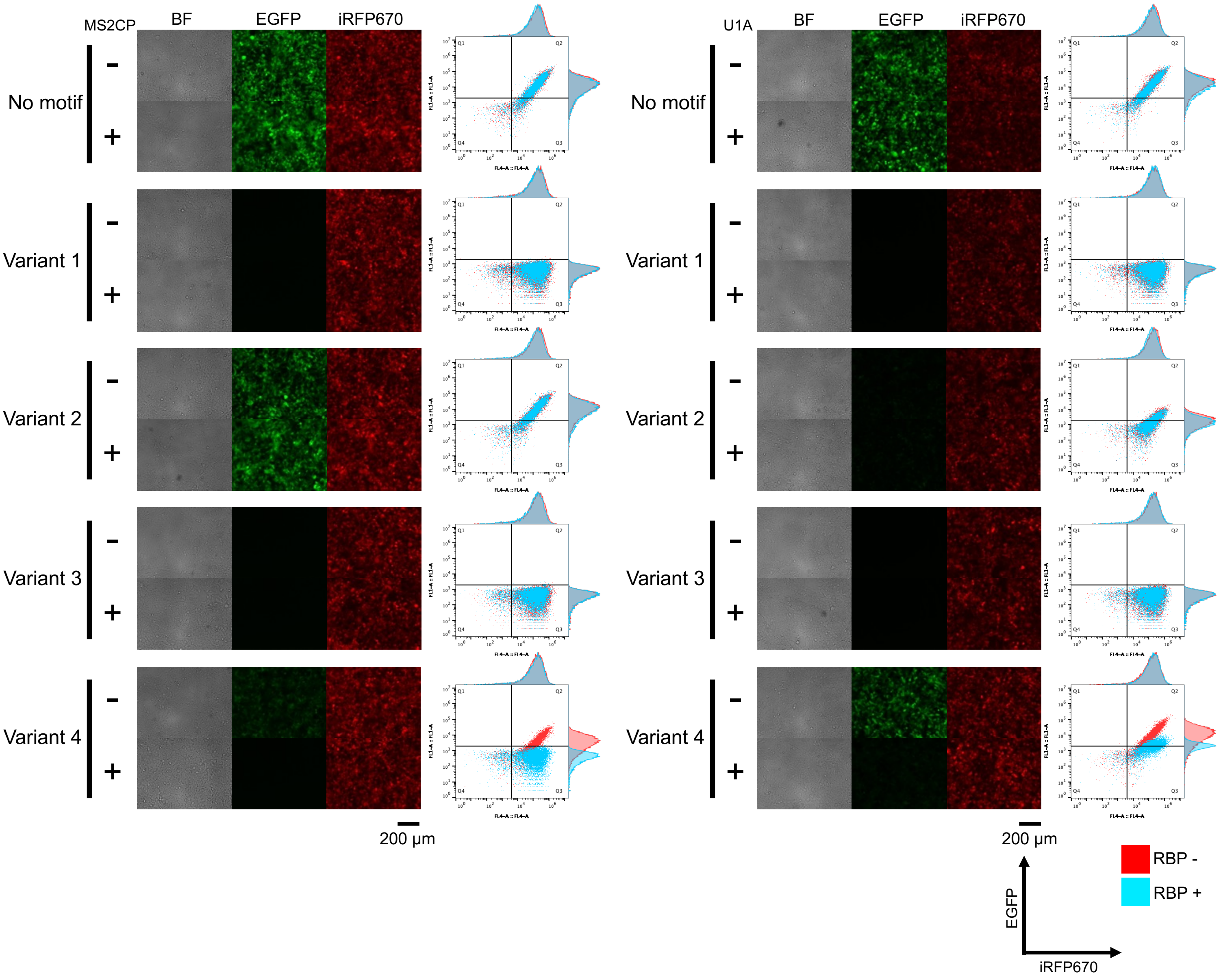

Figure S6. Fluorescent microscopy images and scatter plots from flow cytometry analysis in Figure 5B (including variants 1-4). The scale bar in fluorescent images indicates 200 µm. The plots shown are representative data from three biological replicates. The vertical axis of the scatter plot shows the fluorescence intensity of EGFP, and the horizontal axis shows the fluorescence intensity of iRFP670.

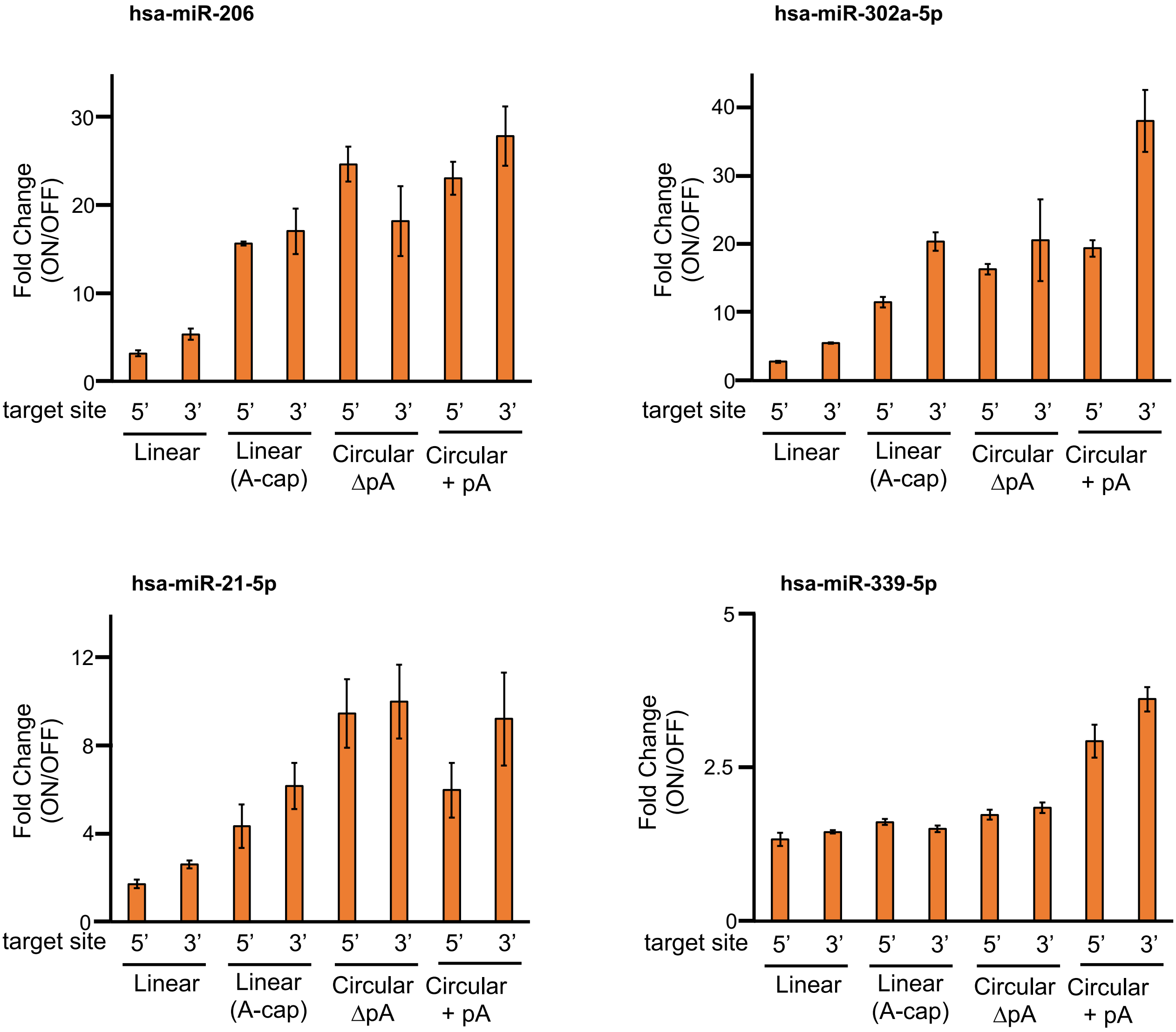

Figure S7. Comparison of miRNA-responsive circRNAs and linear mRNAs with the same sequence components of circRNAs in HEK293FT cells. For circRNA constructs, the results from a single comparison performed simultaneously were presented in two separate graphs, Figure 4B and S7. All data in this figure are presented as mean ± SD, n = 3.

MS2CP responsive variants

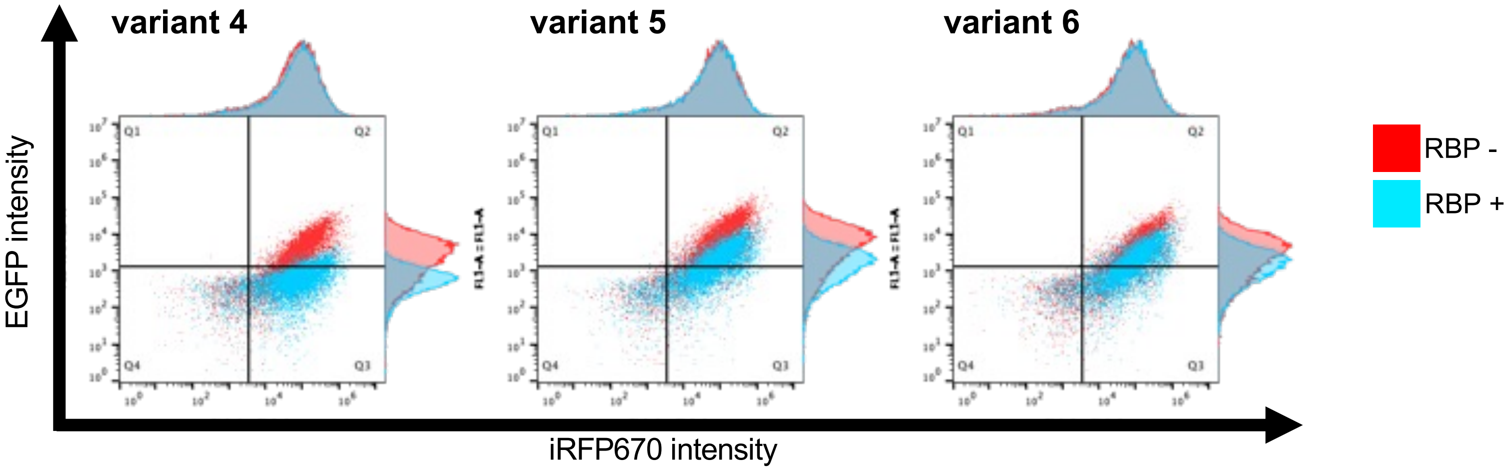

U1A responsive variants

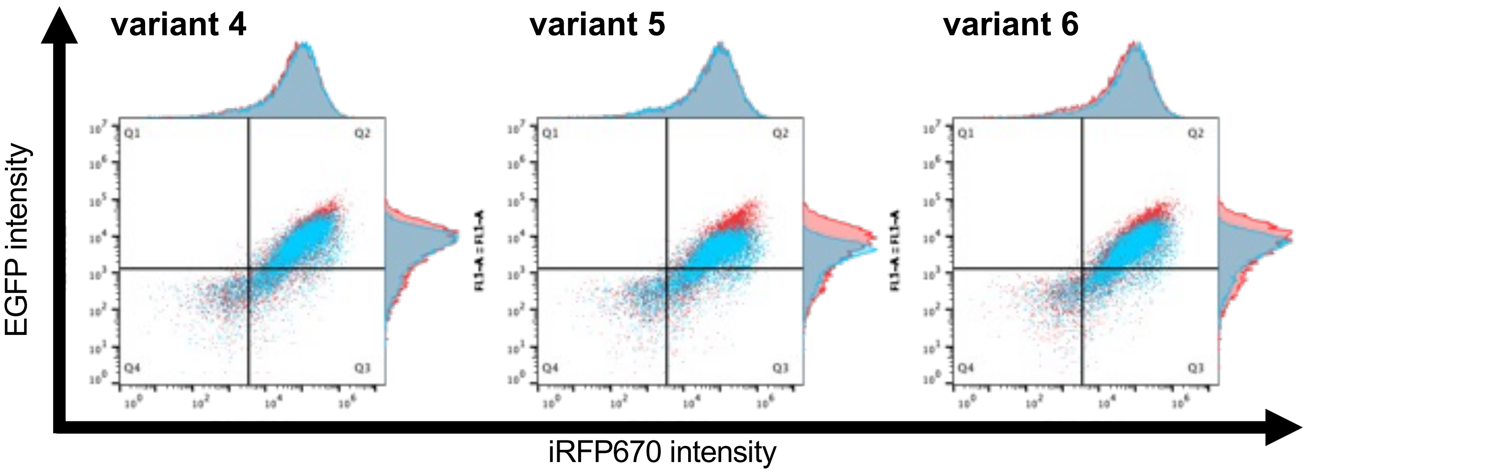

Figure S8. Scatter plots generated from flow cytometry analysis in Figure 5C. The plots shown are representative data from three biological replicates. The vertical axis of the scatter plot shows the fluorescence intensity of EGFP, and the horizontal axis shows the fluorescence intensity of iRFP670.

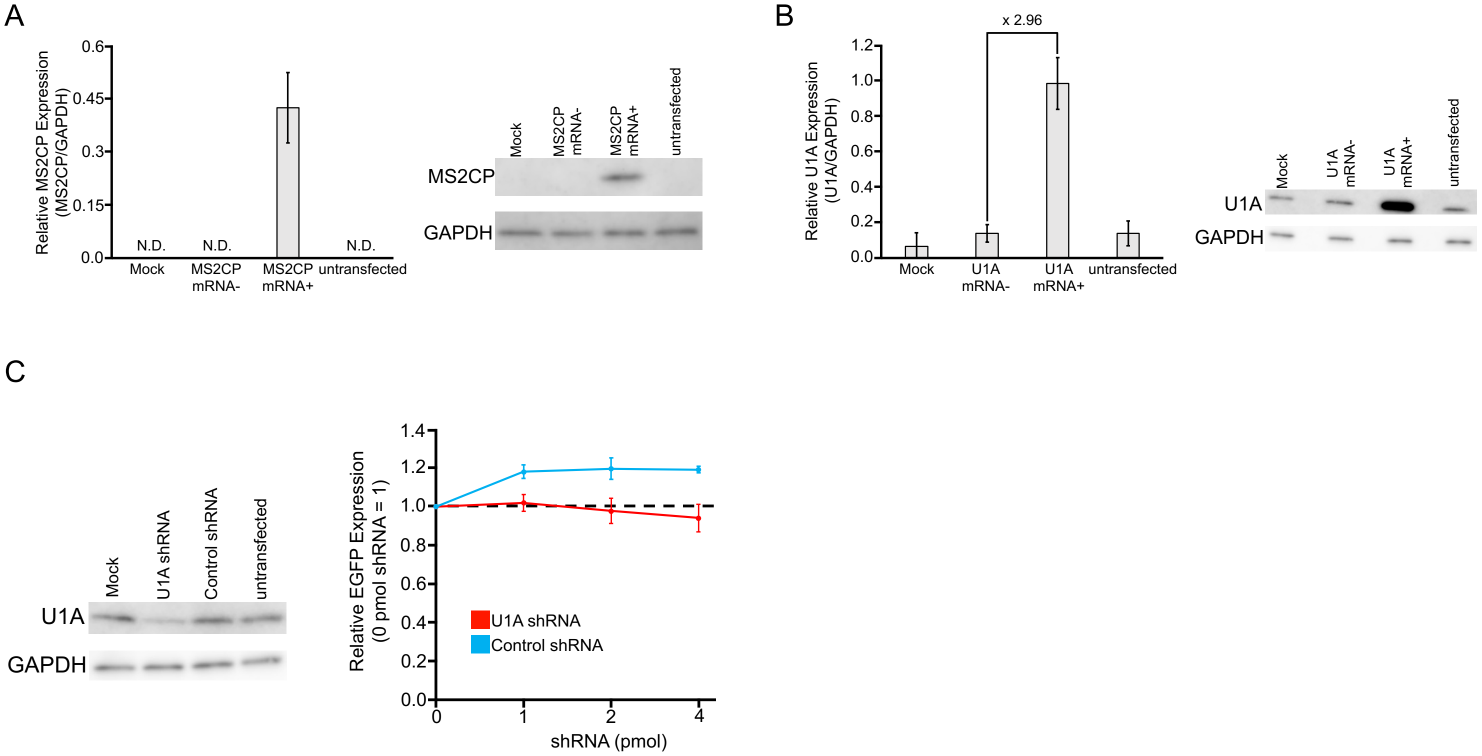

Figure S9. (A) Western blot analysis of MS2CP. 0.05 pmol of *MS2CP*-coding mRNA, 0.3 pmol of each reporter mRNA and transfection control mRNA were co-transfected. Cell culture was performed in 24-well format. (B) Western blot analysis of U1A. 0.15 pmol of *U1A*-coding mRNA, 0.3 pmol of each reporter mRNA and transfection control mRNA were co-transfected. Cell culture was performed in 24-well format. (C) Western blot analysis of U1A (left) and EGFP reporter expression from U1A-responsive circRNA switch (right) with shRNAs. 4 pmol of shRNAs were co-transfected for knockdown evaluation in western blotting. 0.3 pmol of each reporter mRNA and transfection control mRNA were transfected and 1, 2 or 4 pmol of shRNA was co-transfected. The slight increase in reporter expression upon co-transfection of control shRNA has also been observed in previous studies (S6). All data in this figure are presented as mean ± SD, n = 3. The experiments were performed in HEK293FT. GAPDH was used as a loading control for all western blot analyses. The band images of western blotting shown are representative of data from three biological replicates. The contrast of each western blot band shown in the figures was adjusted (Figure S9A・B right, S9C left). Quantification of band intensity was performed using image data before contrast adjustment (Figure S9A・B left graph).

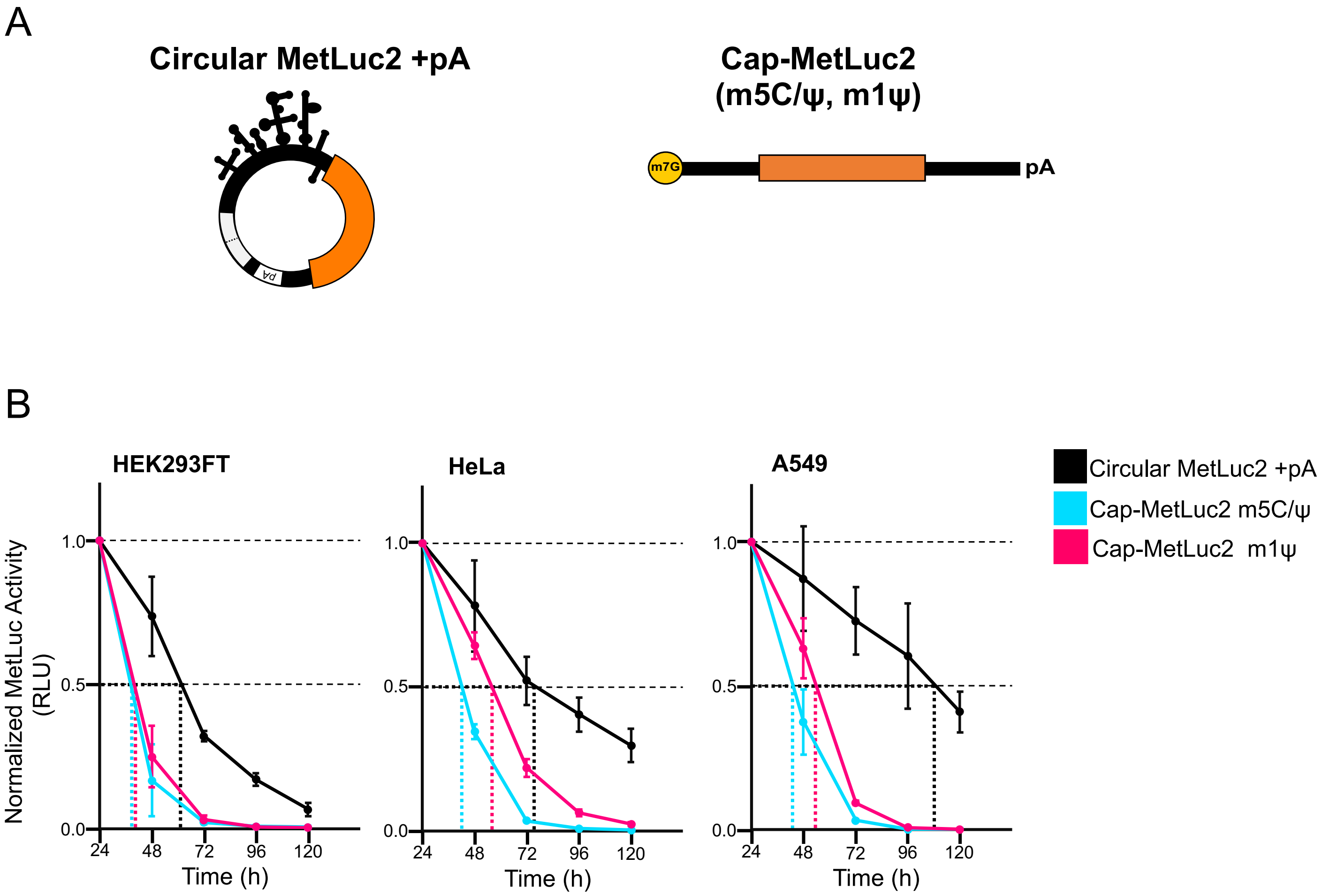

Figure S10. (A) Structure illustration of mRNAs used in the secreted luciferase assay. All mRNAs code Metridia Luciferase (*MetLuc2*) as a reporter gene. (B) Comparison of circRNAs and conventional linear mRNA with base substitutions (m5C/ψ, m1ψ). 45 fmol of reporter mRNA were transfected, and cell culture was performed in 24-well format. All data in this figure are presented as mean ± SD, n = 3.

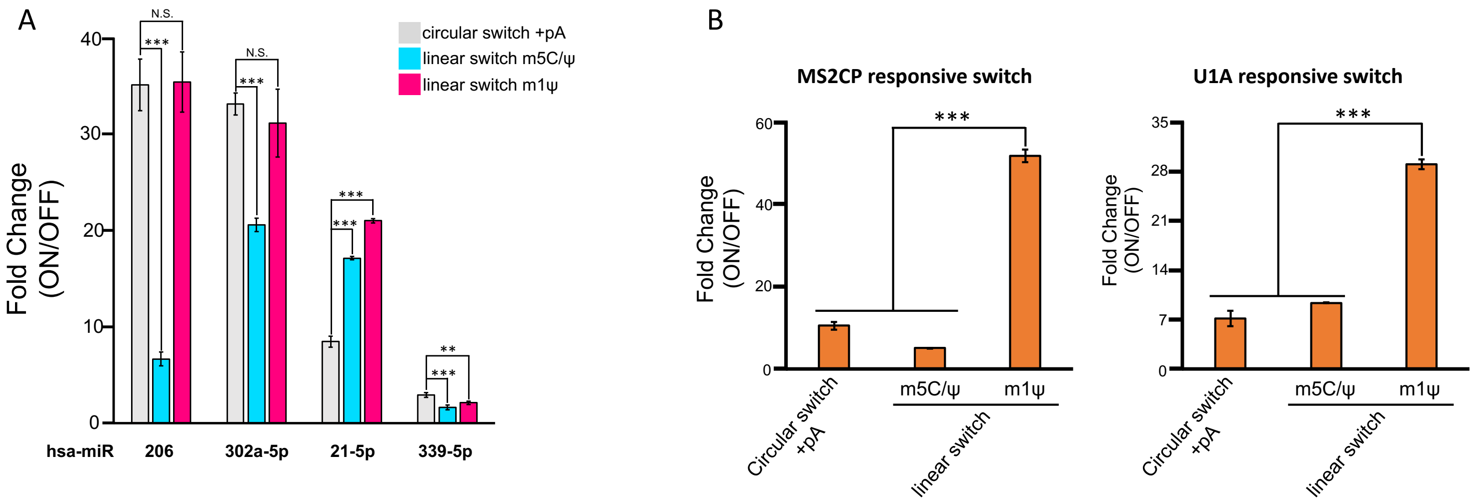

Figure S11. (A) Comparison of the designed miRNA-responsive circRNAs and linear mRNAs with base substitution (m5C/ψ, m1ψ) in HEK293FT cells. 0.3 pmol of each reporter mRNA and transfection control mRNA were transfected, and cell culture was performed in 24-well format. 0.25 pmol of specific miRNA mimic or Negative Control mimic was co-transfected for evaluation. (B) Comparison of MS2CP- or U1A-responsive circRNAs and linear mRNAs with base substitution (m5C/ψ, m1ψ) in HEK293FT cells. 0.3 pmol of each reporter mRNA and transfection control mRNA were transfected and cell culture was performed in 24-well format. 0.05 pmol of *MS2CP*-coding mRNA or 0.15 pmol of *U1A*-coding mRNA was co-transfected for evaluation. Levels of significance are denoted as **P* < 0.05, ****P* < 0.001 (Dunnett's test). N.S. means non-significant (*P* > 0.05). All data in this figure are presented as mean ± SD, n = 3.

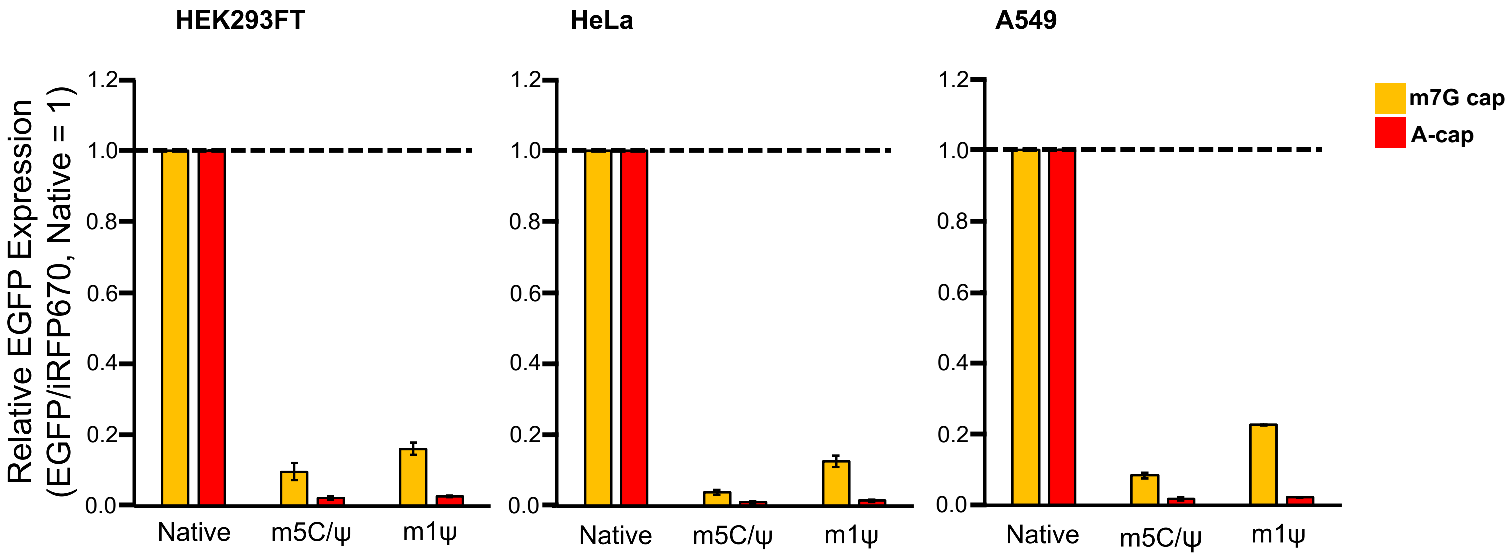

Figure S12. Translatability of linear mRNAs (m7Gcap-EGFP and A-cap-EGFP) with IRES transcribed with non-modified bases (Native) or modified bases (m5C/ψ, m1ψ). 0.3 pmol of each reporter mRNA and transfection control mRNA were transfected, and cell culture was performed in 24-well format. All data in this figure are presented as mean ± SD, n = 3.

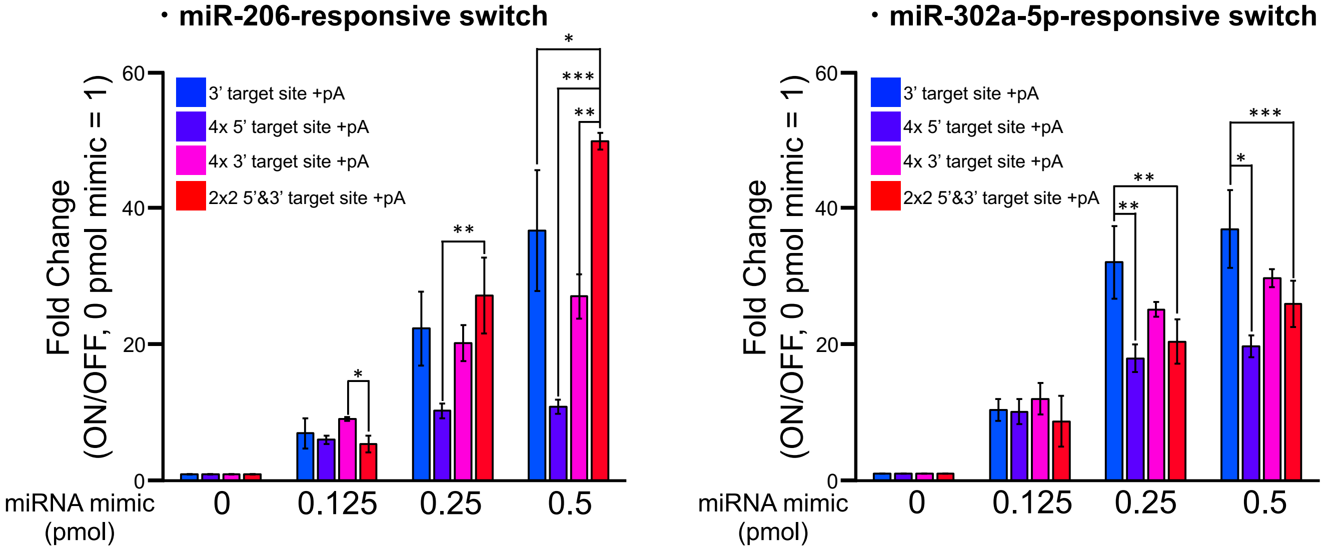

Figure S13. Evaluation of miR-206 or miR-302a-5p-responsive circRNA switches with multiple copies and its patterns of the miRNA target site. 0.3 pmol of each reporter mRNA and transfection control mRNA were transfected and cell culture was performed in 24-well format. 0.125, 0.25, 0.5 pmol of specific miRNA mimic was co-transfected for evaluation. Levels of significance are denoted as *$P < 0.05$, ***$P < 0.001$ (Dunnett’s test). N.S. (non-significant, $P > 0.05$) pairs were not denoted on the graph. All data in this figure are presented as mean ± SD, n = 3.

Supplementary Sequences

Supplementary Sequences. mRNA sequences used in this report

Circular EGFP ΔpA (**bold: circularized**)

Group I intron, CVB3 IRES, EGFP

GGGAGACCCUCGACCGUCGAUUGUCCACUGGUCAACAAUAGAUGACUUACAACUAAUCG
GAAGGUGCAGAGACUCGACGGGAGCUACCCUAACGUCAAGACGAGGGUAAAGAGAG
UCCAAUUCUCAAAGCCAAUAGGCAGUAGCGAAAGCUGCAAGAGAAUG**AAAAUCCGUUGA**
CCUUAAACGGUCGUGUGGGUUCAAGUCCCUCCACCCCCACGCCGGAAACGCAAUAGC
CGGCGAAUUAAGAGAGAAAAGAAGAGUAAGAAGAAAUAUAAGACACCGGUCGCCACC
UUAAAACAGCCUGUGGGUUGAUCCCACCCACAGGCCCAUUGGGCGCUAGCACUCUGG
UAUCACGGUACCUUUGUGCGCCUGUUUUAUACCCCCUCCCCCAACUGUAACUUAGAAG
UAACACACACCGAUCAACAGUCAGCGUGGCACACCAGCCACGUUUUGAUCAAGCACUU
CUGUUACCCCGGACUGAGUAUCAAUAGACUGCUCACGCGGUUGAAGGAGAAAGCGUU
CGUUAUCCGGCCAACUACUUCGAAAAACCUAGUAACACCGUGGAAGUUGCAGAGUGU
UUCGCUCAGCACUACCCCAGUGUAGAUCAGGUCGAUGAGUCACCGCAUUCCCCACGG
GCGACCGUGGCGGUGGCUGCGUUGGCGGCCUGCCCAUGGGGAAACCCAUGGGACGCU
CUAAUACAGACAUGGUGCGAAGAGUCUAUUGAGCUAGUUGGUAGUCCUCCGGCCCCU
GAAUGCGGCUAAUCCUAACUGCGGAGCACACACCCUCAAGCCAGAGGGCAGUGUGUC
GUAACGGGCAACUCUGCAGCGGAACCGACUACUUUGGGUGUCCGUGUUUCAUUUUAU
UCCUAUACUGGCUGCUUAUGGUGACAAUUGAGAGAUCGUUACCAUAUAGCUAUUGGA
UUGGCCAUCCGGUGACUAAUAGAGCUAUUAUAUCCCUUUGUUGGGUUUAUACCACU
UAGCUUGAAAGAGGUUAAAACAUUACAAUUCAUUGUUAAGUUGAAUACAGCAAAauggg
auccgugagcaagggcgaggagcuguucaccgggguggugcccauccuggucgagcuggacggcgacguaaa
cggccacaaguucagcguguccggcgagggcgagggcgaugccaccuacggcaagcugacccugaaguucauc
ugcaccaccggcaagcugcccgugcccuggcccacccucgugaccacccugaccuacggcgugcagugcuuca
gccgcuaccccgaccacaugaagcagcacgacuucuucaaguccgccaugcccgaaggcuacguccaggagcg
caccaucuucuucaaggacgacggcaacuacaagacccgcgccgaggugaaguucgagggcgacacccuggug
aaccgcaucgagcugaagggcaucgacuucaaggaggacggcaacauccuggggcacaagcuggaguacaacu
acaacagccacaacgucuauaucauggccgacaagcagaagaacggcaucaaggugaacuucaagauccgcca
caacaucgaggacggcagcgugcagcucgccgaccacuaccagcagaacacccccaucggcgacggccccgug
cugcugcccgacaaccacuaccugagcacccaguccgcccugagcaaagaccccaacgagaagcgcgaucacau
gguccugcuggaguucgugaccgccgccgggaucacucucggcauggacgagcuguacaagagaucucauau
gcaucucgagugauagucuagaccuucugcggggcuugccuucuggccaugcccuucuucucucccuugcac
cuguaccucuuggucuuuGAAUAAAGCCUGAGUAGGGGCUAUUAUGCGUUACCGGCGAGAC

GCUACGGACUUAAAUAAUUGAGCCUUAAAGAAGAAAUUCUUUAAGUGGAUGCUCUCAAA
CUCAGGGAAACCUAAAUCUAGUUAUAGACAAGGCAAUCCUGAGCCAAGCCGAAGUAGUA
AUUAGUAAGACCAGUGGACAAUCGACGGAUAACAGCAUAUCUAG

Circular EGFP +pA (**bold: circularized**)

Group I intron, CVB3 IRES, EGFP

GGGAGACCCUCGACCGUCGAUUGUCCACUGGUCAACAAUAGAUGACUUACAACUAAUCG
GAAGGUGCAGAGACUCGACGGGAGCUACCCUAACGUCAAGACGAGGGUAAAGAGAGAG
UCCAAUUCUCAAAGCCAAUAGGCAGUAGCGAAAGCUGCAAGAGAAUG**AAAAUCCGUUGA**
CCUUAAACGGUCGUGUGGGUUCAAGUCCCUCCACCCCCACGCCGGAAACGCAAUAGC
CGGCGAAUUAAGAGAGAAAAGAAGAGUAAGAAGAAAUAUAAGACACCGGUCGCCACC
UUAAAACAGCCUGUGGGUUGAUCCCACCCACAGGCCCAUUGGGCGCUAGCACUCUGG
UAUCACGGUACCUUUGUGCGCCUGUUUUAUACCCCCUCCCCCAACUGUAACUUAGAAG
UAACACACACCGAUCAACAGUCAGCGUGGCACACCAGCCACGUUUUGAUCAAGCACUU
CUGUUACCCCGGACUGAGUAUCAAUAGACUGCUCACGCGGUUGAAGGAGAAAGCGUU
CGUUAUCCGGCCAACUACUUCGAAAAACCUAGUAACACCGUGGAAGUUGCAGAGUGU
UUCGCUCAGCACUACCCCAGUGUAGAUCAGGUCGAUGAGUCACCGCAUUCCCCACGG
GCGACCGUGGCGGUGGCUGCGUUGGCGGCCUGCCCAUGGGGAAACCCAUGGGACGCU
CUAAUACAGACAUGGUGCGAAGAGUCUAUUGAGCUAGUUGGUAGUCCUCCGGCCCCU
GAAUGCGGCUAAUCCUAACUGCGGAGCACACACCCUCAAGCCAGAGGGCAGUGUGUC
GUAACGGGCAACUCUGCAGCGGAACCGACUACUUUGGGUGUCCGUGUUUCAUUUUAU
UCCUAUACUGGCUGCUUAUGGUGACAAUUGAGAGAUCGUUACCAUAUAGCUAUUGGA
UUGGCCAUCCGGUGACUAAUAGAGCUAUUAUAUCCCUUUGUUGGGUUUAUACCACU
UAGCUUGAAAGAGGUUAAAACAUUACAAUUCAUUGUUAAGUUGAAUACAGCAAAauggg
auccgugagcaagggcgaggagcuguucaccgggguggugcccauccuggucgagcuggacggcgacguaaa
cggccacaaguucagcguguccggcgagggcgagggcgaugccaccuacggcaagcugacccugaaguucauc
ugcaccaccggcaagcugcccgugcccuggcccacccucgugaccacccugaccuacggcgugcagugcuuca
gccgcuaccccgaccacaugaagcagcacgacuucuucaaguccgccaugcccgaaggcuacguccaggagcg
caccaucuucuucaaggacgacggcaacuacaagacccgcgccgaggugaaguucgagggcgacacccuggug
aaccgcaucgagcugaagggcaucgacuucaaggaggacggcaacauccuggggcacaagcuggaguacaacu
acaacagccacaacgucuauaucauggccgacaagcagaagaacggcaucaaggugaacuucaagauccgcca
caacaucgaggacggcagcgugcagcucgccgaccacuaccagcagaacacccccaucggcgacggccccgug
cugcugcccgacaaccacuaccugagcacccaguccgcccugagcaaagaccccaacgagaagcgcgaucacau
gguccugcuggaguucgugaccgccgccgggaucacucucggcauggacgagcuguacaagagaucucauau
gcaucucgagugauagucuagaccuucugcggggcuugccuucuggccaugcccuucuucucucccuugcac
cuguaccucuuggucuuuGAAUAAAGCCUGAGUAGGAAAAAAAAAAAAAAAAAAAAAAAAA

AAAAAAAAAAAAAAAAAAAAAAAAAAAAAAAAAAAAAAAAAAAAAAAAAAAAAAAAAA
AAAAAAAAAAAAAAAAAAAAAAAAAAAAAAAAAAAAAGGCUAUUAUGCGUUACCGGCG
AGACGCUACGGACUUAAAUAAUUGAGCCUUAAAGAAGAAAUUCUUUAAGUGGAUGCUCU
CAAACUCAGGGAAACCUAAAUCUAGUUAUAGACAAGGCAAUCCUGAGCCAAGCCGAAGU
AGUAAUUAGUAAGACCAGUGGACAAUCGACGGAUAACAGCAUAUCUAGACACAGGAAAC
AGCUAUGACCAUGAUUACGCCAAGCUUGCAUGCCUGCAGGUCGACUCUAGAGGAUCCC
CGGGUACCGAGCUCGAAUU

Circular EGFP ΔpAΔIRES (**bold: circularized**)

Group I intron, CVB3 IRES, EGFP

GGGAGACCCUCGACCGUCGAUUGUCCACUGGUCAACAAUAGAUGACUUACAACUAAUCG
GAAGGUGCAGAGACUCGACGGGAGCUACCCUAACGUCAAGACGAGGGUAAAGAGAG
UCCAAUUCUCAAAGCCAAUAGGCAGUAGCGAAAGCUGCAAGAGAAUG**AAAAUCCGUUGA**
CCUUAAACGGUCGUGUGGGUUCAAGUCCCUCCACCCCCACGCCGGAAACGCAAUAGC
CGGCGAAUUAAGAGAGAAAAGAAGAGUAAGAAGAAAUAUAAGACACCGGUCGCCACC
augggauccgugagcaagggcgaggagcuguucaccgggguggugcccauccuggucgagcuggacggcgac
guaaacggccacaaguucagcguguccggcgagggcgagggcgaugccaccuacggcaagcugacccugaagu
ucaucugcaccaccggcaagcugcccgugcccuggcccacccucgugaccacccugaccuacggcgugcagug
cuucagccgcuaccccgaccacaugaagcagcacgacuucuucaaguccgccaugcccgaaggcuacguccag
gagcgcaccaucuucuucaaggacgacggcaacuacaagacccgcgccgaggugaaguucgagggcgacaccc
uggugaaccgcaucgagcugaagggcaucgacuucaaggaggacggcaacauccuggggcacaagcuggagu
acaacuacaacagccacaacgucuauaucauggccgacaagcagaagaacggcaucaaggugaacuucaagau
ccgccacaacaucgaggacggcagcgugcagcucgccgaccacuaccagcagaacacccccaucggcgacggcc
ccgugcugcugcccgacaaccacuaccugagcacccaguccgcccugagcaaagaccccaacgagaagcgcga
ucacaugguccugcuggaguucgugaccgccgccgggaucacucucggcauggacgagcuguacaagagauc
ucauaugcaucucgagugauagucuagaccuucugcggggcuugccuucuggccaugcccuucuucucuccc
uugcaccuguaccucuuggucuuuGAAUAAAGCCUGAGUAGGGGCUAUUAUGCGUUACCGGC
GAGACGCUACGGACUUAAAUAAUUGAGCCUUAAAGAAGAAAUUCUUUAAGUGGAUGCUC
UCAAACUCAGGGAAACCUAAAUCUAGUUAUAGACAAGGCAAUCCUGAGCCAAGCCGAAG
UAGUAAUUAGUAAGACCAGUGGACAAUCGACGGAUAACAGCAUAUCUAG

Circular EGFP +pAΔIRES (**bold: circularized**)

Group I intron, CVB3 IRES, EGFP

GGGAGACCCUCGACCGUCGAUUGUCCACUGGUCAACAAUAGAUGACUUACAACUAAUCG
GAAGGUGCAGAGACUCGACGGGAGCUACCCUAACGUCAAGACGAGGGUAAAGAGAG
UCCAAUUCUCAAAGCCAAUAGGCAGUAGCGAAAGCUGCAAGAGAAUG**AAAAUCCGUUGA**

CCUUAAACGGUCGUGUGGGUUCAAGUCCCUCCACCCCCACGCCGGAAACGCAAUAGC
CGGCGAAUUAAGAGAGAAAAGAAGAGUAAGAAGAAAUAUAAGACACCGGUCGCCACC
augggauccgugagcaagggcgaggagcuguucaccgggguggugcccauccuggucgagcuggacggcgac
guaaacggccacaaguucagcguguccggcgagggcgagggcgaugccaccuacggcaagcugacccugaagu
ucaucugcaccaccggcaagcugcccgugcccuggcccacccucgugaccacccugaccuacggcgugcagug
cuucagccgcuaccccgaccacaugaagcagcacgacuucuucaaguccgccaugcccgaaggcuacguccag
gagcgcaccaucuucuucaaggacgacggcaacuacaagacccgcgccgaggugaaguucgagggcgacaccc
uggugaaccgcaucgagcugaagggcaucgacuucaaggaggacggcaacauccuggggcacaagcuggagu
acaacuacaacagccacaacgucuauaucauggccgacaagcagaagaacggcaucaaggugaacuucaagau
ccgccacaacaucgaggacggcagcgugcagcucgccgaccacuaccagcagaacacccccaucggcgacggcc
ccgugcugcugcccgacaaccacuaccugagcacccaguccgcccugagcaaagaccccaacgagaagcgcga
ucacaugguccugcuggaguucgugaccgccgccgggaucacucucggcauggacgagcuguacaagagauc
ucauaugcaucucgagugauagucuagaccuucugcggggcuugccuucuggccaugcccuucuucucuccc
uugcaccuguaccucuuggucuuuGAAUAAAGCCUGAGUAGGAAAAAAAAAAAAAAAAAAAA
AAAAAAAAAAAAAAAAAAAAAAAAAAAAAAAAAAAAAAAAAAAAAAAAAAAAAAAAA
AAAAAAAAAAAAAAAAAAAAAAAAAAAAAAAAAAAAAAAAAGGCUAUUAUGCGUUAC
CGGCGAGACGCUACGGACUUAAAUAAUUGAGCCUUAAAGAAGAAAUUCUUUAAGUGGAU
GCUCUCAAACUCAGGGAAACCUAAAUCUAGUUAUAGACAAGGCAAUCCUGAGCCAAGCC
GAAGUAGUAAUUAGUAAGACCAGUGGACAAUCGACGGAUAACAGCAUAUCUAGACACAG
GAAACAGCUAUGACCAUGAUUACGCCAAGCUUGCAUGCCUGCAGGUCGACUCUAGAGGA
UCCCCGGGUACCGAGCUCGAAUU

Linear EGFP

Group I intron, CVB3 IRES, EGFP

GGGAAAAUCCGUUGACCUUAAACGGUCGUGUGGGUUCAAGUCCCUCCACCCCCACGCC
GGAAACGCAAUAGCCGGCGAAUUAAGAGAGAAAAGAAGAGUAAGAAGAAAUAUAAGACAC
CGGUCGCCACCUUAAAACAGCCUGUGGGUUGAUCCCACCCACAGGCCCAUUGGGCGCU
AGCACUCUGGUAUCACGGUACCUUUGUGCGCCUGUUUUAUACCCCCUCCCCCAACUGU
AACUUAGAAGUAACACACACCGAUCAACAGUCAGCGUGGCACACCAGCCACGUUUUGAU
CAAGCACUUCUGUUACCCCGGACUGAGUAUCAAUAGACUGCUCACGCGGUUGAAGGAG
AAAGCGUUCGUUAUCCGGCCAACUACUUCGAAAAACCUAGUAACACCGUGGAAGUUGCA
GAGUGUUUCGCUCAGCACUACCCCAGUGUAGAUCAGGUCGAUGAGUCACCGCAUUCCC
CACGGGCGACCGUGGCGGUGGCUGCGUUGGCGGCCUGCCCAUGGGGAAACCCAUGGG
ACGCUCUAAUACAGACAUGGUGCGAAGAGUCUAUUGAGCUAGUUGGUAGUCCUCCGGC
CCCUGAAUGCGGCUAAUCCUAACUGCGGAGCACACACCCUCAAGCCAGAGGGCAGUGU
GUCGUAACGGGCAACUCUGCAGCGGAACCGACUACUUUGGGUGUCCGUGUUUCAUUUU

AUUCCUAUACUGGCUGCUUAUGGUGACAAUUGAGAGAUCGUUACCAUAUAGCUAUUGGA
UUGGCCAUCCGGUGACUAAUAGAGCUAUUAUAUCCCUUUGUUGGGUUUAUACCACUU
AGCUUGAAAGAGGUUAAAACAUUACAAUUCAUUGUUAAGUUGAAUACAGCAAAaugggaucc
gugagcaagggcgaggagcuguucaccgggguggugcccauccuggucgagcuggacggcgacguaaacggccacaag
uucagcguguccggcgagggcgagggcgaugccaccuacggcaagcugacccugaaguucaucugcaccaccggcaagc
ugcccgugcccuggcccacccucgugaccacccugaccuacggcgugcagugcuucagccgcuaccccgaccacaugaa
gcagcacgacuucuucaaguccgccaugcccgaaggcuacguccaggagcgcaccaucuucuucaaggacgacggcaac
uacaagacccgcgccgaggugaaguucgagggcgacacccuggugaaccgcaucgagcugaagggcaucgacuucaag
gaggacggcaacauccuggggcacaagcuggaguacaacuacaacagccacaacgucuauaucauggccgacaagcag
aagaacggcaucaaggugaacuucaagauccgccacaacaucgaggacggcagcgugcagcucgccgaccacuaccagc
agaacacccccaucggcgacggccccgugcugcugcccgacaaccacuaccugagcacccaguccgcccugagcaaaga
ccccaacgagaagcgcgaucacaugguccugcuggaguucgugaccgccgccgggaucacucucggcauggacgagcug
uacaagagaucucauaugcaucucgagugauagucuagaccuucugcggggcuugccuucuggccaugcccuucuucu
cucccuugcaccuguaccucuuggucuuuGAAUAAAGCCUGAGUAGGGGCUAUUAUGCGUUACCGG
CGAGACGCUACGGACUUAAAAAAAAAAAAAAAAAAAAAAAAAAAAAAAAAAAAAAAAAAAAA
AAAAAAAAAAAAAAAAAAAAAAAAAAAAAAAAAAAAAAAAAAAAAAAAAAAAAAAAAAAAAAA
AAAAAAAAAAAA

Cap-EGFP

EGFP

GGGCGAAUUAAGAGAGAAAAGAAGAGUAAGAAGAAAUAUAAGACACCGGUcgccaccauggga
uccgugagcaagggcgaggagcuguucaccgggguggugcccauccuggucgagcuggacggcgacguaaacggccac
aaguucagcguguccggcgagggcgagggcgaugccaccuacggcaagcugacccugaaguucaucugcaccaccggc
aagcugcccgugcccuggcccacccucgugaccacccugaccuacggcgugcagugcuucagccgcuaccccgaccacau
gaagcagcacgacuucuucaaguccgccaugcccgaaggcuacguccaggagcgcaccaucuucuucaaggacgacggc
aacuacaagacccgcgccgaggugaaguucgagggcgacacccuggugaaccgcaucgagcugaagggcaucgacuuc
aaggaggacggcaacauccuggggcacaagcuggaguacaacuacaacagccacaacgucuauaucauggccgacaag
cagaagaacggcaucaaggugaacuucaagauccgccacaacaucgaggacggcagcgugcagcucgccgaccacuacc
agcagaacacccccaucggcgacggccccgugcugcugcccgacaaccacuaccugagcacccaguccgcccugagcaa
agaccccaacgagaagcgcgaucacaugguccugcuggaguucgugaccgccgccgggaucacucucggcauggacga
gcuguacaagagaucucauaugcaucucgagugauagucuagaccuucugcggggcuugccuucuggccaugcccuuc
uucucucccuugcaccuguaccucuuggucuuuGAAUAAAGCCUGAGUAGGAAAAAAAAAAAAAAAAA
AAAAAAAAAAAAAAAAAAAAAAAAAAAAAAAAAAAAAAAAAAAAAAAAAAAAAAAAAAAAAAA
AAAAAAAAAAAAAAAAAAAAAAAAAAAAAAAAAAAAAAAA

Cap-iRFP670

iRFP670

GGGCGAAUUAAGAGAGAAAAGAAGAGUAAGAAGAAAUAUAAGACACCGGUcgccaccATGgc
gcguaaggucgaucucaccuccugcgaucgcgagccgauccacaucccggcagcauucagccgugcggcugccugcua
gccugcgacgcgcaggcggugcggaucacgcgcauuacggaaaaugccggcgcguucuuuggacgcgaaacuccgcgg
gucggugagcuacucgccgauuacuucggcgagaccgaagcccaugcgcugcgcaacgcacuggcgcaguccuccgauc
caaagcgaccggcgcugaucuucgguuggcgcgacggccugaccggccgcaccuucgacaucucacugcaucgccauga
cgguacaucgaucaucgaguucgagccugcggcggccgaacaggccgacaauccgcugcggcugacgcggcagaucauc
gcgcgcaccaaagaacugaagucgcucgaagagauggccgcacgggugccgcgcuaucugcaggcgaugcucggcuau
caccgcgugauguuguaccgcuucgcggacgacggcuccgggauggugaucggcgaggcgaagcgcagcgaccucgag
agcuuucucggucagcacuuuccggcgucgcugguccgcagcaggcgcggcuacuguacuugaagaacgcgauccgcg
uggucucggauucgcgcggcaucagcagccggaucgugcccgagcacgacgccuccggcgccgcgcucgaucugucguu
cgcgcaccugcgcagcaucucgcccugccaucucgaauuucugcggaacaugggcgucagcgccucgaugucgcugucg
aucaucauugacggcacgcuaugggauugaucaucugucaucauuacgagccgcgugccgugccgauggcgcagcgc
gucgcggccgaaauguucgccgacuucuuaucgcugcacuucaccgccgcccaccaccaacgcagaucucauauGCAU
CUCGAGUGAUAGucuagaccuucugcggggcuugccuucuggccaugcccuucuucucucccuugcaccuguacc
ucuuggucuuuGAAUAAAGCCUGAGUAGGAAAAAAAAAAAAAAAAAAAAAAAAAAAAAAAAA
AAAAAAAAAAAAAAAAAAAAAAAAAAAAAAAAAAAAAAAAAAAAAAAAAAAAAAAAAAAAAAA
AAAAAAAAAAAAAAAAAAAAAA

Circular MetLuc2 +pA (**bold: circularized**)

Group I intron, CVB3 IRES, MetLuc2

GGGAGACCCUCGACCGUCGAUUGUCCACUGGUCAACAAUAGAUGACUUACAACUAAUCG
GAAGGUGCAGAGACUCGACGGGAGCUACCCUAACGUCAAGACGAGGGUAAAGAGAG
UCCAAUUCUCAAAGCCAAUAGGCAGUAGCGAAAGCUGCAAGAGAAUG**AAAAUCCGUUGA**
CCUUAAACGGUCGUGUGGGUUCAAGUCCCUCCACCCCCACGCCGGAAACGCAAUAGC
CGGCGAAUUAAGAGAGAAAAGAAGAGUAAGAAGAAAUAUAAGACACCGGUCGCCACC
UUAAAACAGCCUGUGGGUUGAUCCCACCCACAGGCCCAUUGGGCGCUAGCACUCUGG
UAUCACGGUACCUUUGUGCGCCUGUUUUAUACCCCCUCCCCCAACUGUAACUUAGAAG
UAACACACACCGAUCAACAGUCAGCGUGGCACACCAGCCACGUUUUGAUCAAGCACUU
CUGUUACCCCGGACUGAGUAUCAAUAGACUGCUCACGCGGUUGAAGGAGAAAGCGUU
CGUUAUCCGGCCAACUACUUCGAAAAACCUAGUAACACCGUGGAAGUUGCAGAGUGU
UUCGCUCAGCACUACCCCAGUGUAGAUCAGGUCGAUGAGUCACCGCAUUCCCCACGG
GCGACCGUGGCGGUGGCUGCGUUGGCGGCCUGCCCAUGGGGAAACCCAUGGGACGCU
CUAAUACAGACAUGGUGCGAAGAGUCUAUUGAGCUAGUUGGUAGUCCUCCGGCCCCU
GAAUGCGGCUAAUCCUAACUGCGGAGCACACACCCUCAAGCCAGAGGGCAGUGUGUC
GUAACGGGCAACUCUGCAGCGGAACCGACUACUUUGGGUGUCCGUGUUUCAUUUUAU

UCCUAUACUGGCUGCUUAUGGUGACAAUUGAGAGAUCGUUACCAUAUAGCUAUUGGA UUGGCCAUCCGGUGACUAAUAGAGCUAUUAUAUCCCUUUGUUGGGUUUAUACCACU UAGCUUGAAAGAGGUUAAAACAUUACAAUUCAUUGUUAAGUUGAAUACAGCAAAaugga caucaagguggguguucacccugguguucagcgcccuggugcaggccaagagcaccgaguucgaccccaacauc gacaucgugggccuggaaggcaaguucggcaucaccaaccuggaaaccgaccuguucaccaucugggagacca uggaagugaugaucaaggccgacaucgccgacaccgaccgggccagcaacuucguggccaccgagaccgacgc caaccggggcaagaugcccggcaagaagcugccccuggccgucaucauggaaauggaagccaacgccuucaag gccggcugcacccggggcugccugaucugccugagcaagaucaagugcaccgccaagaugaagguguacaucc ccggcaggugccacgacuacggcggcgacaagaaaaccggccaggccggcaucgugggcgccaucguggacau ccccgagaucagcggcuucaaagaaauggcccccauggaacaguucaucgcccagguggacagaugcgccagc ugcaccaccggcugccugaagggccuggccaacgugaagugcagcgagcugcugaagaaguggcugcccgacc gcugcgccagcuucgccgacaagauccagaaagaggugcacaacaucaagggcauggccggcgacaggugauc uagaccuucugcggggcuugccuucuggccaugcccuucuucucucccuugcaccuguaccucuuggucuuu GAAUAAAGCCUGAGUAGGAAAAAAAAAAAAAAAAAAAAAAAAAAAAAAAAAAAAAAAAAA AAAAAAAAAAAAAAAAAAAAAAAAAAAAAAAAAAAAAAAAAAAAAAAAAAAAAAAAAAAA AAAAAAAAAAAAAAAAAAAAAAGGCUAUUAUGCGUUACCGGCGAGACGCUACGGACUU
AAAUAAUUGAGCCUUAAAGAAGAAAUUCUUUAAGUGGAUGCUCUCAAACUCAGGGAAAC CUAAAUCUAGUUAUAGACAAGGCAAUCCUGAGCCAAGCCGAAGUAGUAAUUAGUAAGAC CAGUGGACAAUCGACGGAUAACAGCAUAUCUAGACACAGGAAACAGCUAUGACCAUGAU UACGCCAAGCUUGCAUGCCUGCAGGUCGACUCUAGAGGAUCCCCGGGUACCGAGCUCG AAUU

Cap-MetLuc2

MetLuc2

GGGCGAAUUAAGAGAGAAAAGAAGAGUAAGAAGAAAUAUAAGACACCGGUcgccaccauggac aucaagguggguguucacccugguguucagcgcccuggugcaggccaagagcaccgaguucgaccccaacaucgacauc gugggccuggaaggcaaguucggcaucaccaaccuggaaaccgaccuguucaccaucugggagaccauggaagugaug aucaaggccgacaucgccgacaccgaccgggccagcaacuucguggccaccgagaccgacgccaaccggggcaagaugc ccggcaagaagcugccccuggccgucaucauggaaauggaagccaacgccuucaaggccggcugcacccggggcugccu gaucugccugagcaagaucaagugcaccgccaagaugaagguguacauccccggcaggugccacgacuacggcggcga caagaaaaccggccaggccggcaucgugggcgccaucguggacauccccgagaucagcggcuucaaagaaauggccccc auggaacaguucaucgcccagguggacagaugcgccagcugcaccaccggcugccugaagggccuggccaacgugaag ugcagcgagcugcugaagaaguggcugcccgaccgcugcgccagcuucgccgacaagauccagaaagaggugcacaac aucaagggcauggccggcgacaggugaucuagaccuucugcggggcuugccuucuggccaugcccuucuucucucccuu gcaccuguaccucuuggucuuuGAAUAAAGCCUGAGUAGGAAAAAAAAAAAAAAAAAAAAAAAAAA AAAAAAAAAAAAAAAAAAAAAAAAAAAAAAAAAAAAAAAAAAAAAAAAAAAAAAAAAAAAAAA

AAAAAAAAAAAAAAAAAAAAAAAAAAAAAA

5’T206 Circular EGFP ΔpA (**bold: circularized**)

Group I intron, CVB3 IRES, EGFP, miR-206 target site

GGGAGACCCUCGACCGUCGAUUGUCCACUGGUCAACAAUAGAUGACUUACAACUAAUCG
GAAGGUGCAGAGACUCGACGGGAGCUACCCUAACGUCAAGACGAGGGUAAAGAGAGAG
UCCAAUUCUCAAAGCCAAUAGGCAGUAGCGAAAGCUGCAAGAGAAUG**AAAAUCCGUUGA**
CCUUAAACGGUCGUGUGGGUUCAAGUCCCUCCACCCCCACGCCGGAAACGCAAUAGC
CGGCGAAUUAAGAGAGAAAAGAAGAGUAAGAAGAAAUAUAAGACACCGGUCCCACAC
ACUUCCUUACAUUCCAGCCACCUUAAAACAGCCUGUGGGUUGAUCCCACCCACAGGCC
CAUUGGGCGCUAGCACUCUGGUAUCACGGUACCUUUGUGCGCCUGUUUUAUACCCCC
UCCCCCAACUGUAACUUAGAAGUAACACACACCGAUCAACAGUCAGCGUGGCACACCA
GCCACGUUUUGAUCAAGCACUUCUGUUACCCCGGACUGAGUAUCAAUAGACUGCUCAC
GCGGUUGAAGGAGAAAGCGUUCGUUAUCCGGCCAACUACUUCGAAAAACCUAGUAAC
ACCGUGGAAGUUGCAGAGUGUUUCGCUCAGCACUACCCCAGUGUAGAUCAGGUCGAU
GAGUCACCGCAUUCCCCACGGGCGACCGUGGCGGUGGCUGCGUUGGCGGCCUGCCCA
UGGGGAAACCCAUGGGACGCUCUAAUACAGACAUGGUGCGAAGAGUCUAUUGAGCUA
GUUGGUAGUCCUCCGGCCCCUGAAUGCGGCUAAUCCUAACUGCGGAGCACACACCCU
CAAGCCAGAGGGCAGUGUGUCGUAACGGGCAACUCUGCAGCGGAACCGACUACUUUG
GGUGUCCGUGUUUCAUUUUAUUCCUAUACUGGCUGCUUAUGGUGACAAUUGAGAGAU
CGUUACCAUAUAGCUAUUGGAUUGGCCAUCCGGUGACUAAUAGAGCUAUUAUAUCC
CUUUGUUGGGUUUAUACCACUUAGCUUGAAAGAGGUUAAAACAUUACAAUUCAUUGUU
AAGUUGAAUACAGCAAAaugggauccgugagcaagggcgaggagcuguucaccgggguggugcccauc
cuggucgagcuggacggcgacguaaacggccacaaguucagcguguccggcgagggcgagggcgaugccacc
uacggcaagcugacccugaaguucaucugcaccaccggcaagcugcccgugcccuggcccacccucgugacca
cccugaccuacggcgugcagugcuucagccgcuaccccgaccacaugaagcagcacgacuucuucaaguccgc
caugcccgaaggcuacguccaggagcgcaccaucuucuucaaggacgacggcaacuacaagacccgcgccgag
gugaaguucgagggcgacacccuggugaaccgcaucgagcugaagggcaucgacuucaaggaggacggcaaca
uccuggggcacaagcuggaguacaacuacaacagccacaacgucuauaucauggccgacaagcagaagaacgg
caucaaggugaacuucaagauccgccacaacaucgaggacggcagcgugcagcucgccgaccacuaccagcag
aacacccccaucggcgacggccccgugcugcugcccgacaaccacuaccugagcacccaguccgcccugagcaa
agaccccaacgagaagcgcgaucacaugguccugcuggaguucgugaccgccgccgggaucacucucggcaug
gacgagcuguacaagagaucucauaugcaucucgagugauagucuagaccuucugcggggcuugccuucugg
ccaugcccuucuucucucccuugcaccuguaccucuuggucuuuGAAUAAAGCCUGAGUAGGGGCU
AUUAUGCGUUACCGGCGAGACGCUACGGACUUAAAUAAUUGAGCCUUAAAGAAGAAAU
UCUUUAAGUGGAUGCUCUCAAACUCAGGGAAACCUAAAUCUAGUUAUAGACAAGGCAAU

CCUGAGCCAAGCCGAAGUAGUAAUUAGUAAGACCAGUGGACAAUCGACGGAUAACAGCA
UAUCUAG

5'T206 Circular EGFP +pA (**bold: circularized**)

Group I intron, CVB3 IRES, EGFP, miR-206 target site

GGGAGACCCUCGACCGUCGAUUGUCCACUGGUCAACAAUAGAUGACUUACAACUAAUCG
GAAGGUGCAGAGACUCGACGGGAGCUACCCUAACGUCAAGACGAGGGUAAAGAGAG
UCCAAUUCUCAAAGCCAAUAGGCAGUAGCGAAAGCUGCAAGAGAAUG**AAAAUCCGUUGA
CCUUAAACGGUCGUGUGGGUUCAAGUCCCUCCACCCCCACGCCGGAAACGCAAUAGC
CGGCGAAUUAAGAGAGAAAAGAAGAGUAAGAAGAAAUAUAAGACACCGGUCCCACAC
ACUUCCUUACAUUCCAGCCACCUUAAAACAGCCUGUGGGUUGAUCCCACCCACAGGCC
CAUUGGGCGCUAGCACUCUGGUAUCACGGUACCUUUGUGCGCCUGUUUUAUACCCCC
UCCCCCAACUGUAACUUAGAAGUAACACACACCGAUCAACAGUCAGCGUGGCACACCA
GCCACGUUUUGAUCAAGCACUUCUGUUACCCCGGACUGAGUAUCAAUAGACUGCUCAC
GCGGUUGAAGGAGAAAGCGUUCGUUAUCCGGCCAACUACUUCGAAAAACCUAGUAAC
ACCGUGGAAGUUGCAGAGUGUUUCGCUCAGCACUACCCCAGUGUAGAUCAGGUCGAU
GAGUCACCGCAUUCCCCACGGGCGACCGUGGCGGUGGCUGCGUUGGCGGCCUGCCCA
UGGGGAAACCCAUGGGACGCUCUAAUACAGACAUGGUGCGAAGAGUCUAUUGAGCUA
GUUGGUAGUCCUCCGGCCCCUGAAUGCGGCUAAUCCUAACUGCGGAGCACACACCCU
CAAGCCAGAGGGCAGUGUGUCGUAACGGGCAACUCUGCAGCGGAACCGACUACUUUG
GGUGUCCGUGUUUCAUUUUAUUCCUAUACUGGCUGCUUAUGGUGACAAUUGAGAGAU
CGUUACCAUAUAGCUAUUGGAUUGGCCAUCCGGUGACUAAUAGAGCUAUUAUAUCC
CUUUGUUGGGUUUAUACCACUUAGCUUGAAAGAGGUUAAAACAUUACAAUUCAUUGUU
AAGUUGAAUACAGCAAAaugggauccgugagcaagggcgaggagcuguucaccgggguggugcccauc
cuggucgagcuggacggcgacguaaacggccacaaguucagcguguccggcgagggcgagggcgaugccacc
uacggcaagcugacccugaaguucaucugcaccaccggcaagcugcccgugcccuggcccacccucgugacca
cccugaccuacggcgugcagugcuucagccgcuaccccgaccacaugaagcagcacgacuucuucaaguccgc
caugcccgaaggcuacguccaggagcgcaccaucuucuucaaggacgacggcaacuacaagacccgcgccgag
gugaaguucgagggcgacacccuggugaaccgcaucgagcugaagggcaucgacuucaaggaggacggcaaca
uccuggggcacaagcuggaguacaacuacaacagccacaacgucuauaucauggccgacaagcagaagaacgg
caucaaggugaacuucaagauccgccacaacaucgaggacggcagcgugcagcucgccgaccacuaccagcag
aacacccccaucggcgacggccccgugcugcugcccgacaaccacuaccugagcacccaguccgcccugagcaa
agaccccaacgagaagcgcgaucacaugguccugcuggaguucgugaccgccgccgggaucacucucggcaug
gacgagcuguacaagagaucucauaugcaucucgagugauagucuagaccuucugcggggcuugccuucugg
ccaugcccuucuucucucccuugcaccuguaccucuuggucuuuGAAUAAAGCCUGAGUAGGAAAA
AAAAAAAAAAAAAAAAAAAAAAAAAAAAAAAAAAAAAAAAAAAAAAAAAAAAAAAAAAAAAAAA**

AAAAAAAAAAAAAAAAAAAAAAAAAAAAAAAAAAAAAAAAAAAAAAAAAAAAAAAAAAAAAAAA
GGCUAUUAUGCGUUACCGGCGAGACGCUACGGACUUAAAUAAUUGAGCCUUAAAGAAG
AAAUUCUUUAAGUGGAUGCUCUCAAACUCAGGGAAACCUAAAUCUAGUUAUAGACAAGG
CAAUCCUGAGCCAAGCCGAAGUAGUAAUUAGUAAGACCAGUGGACAAUCGACGGAUAAC
AGCAUAUCUAGACACAGGAAACAGCUAUGACCAUGAUUACGCCAAGCUUGCAUGCCUGC
AGGUCGACUCUAGAGGAUCCCCGGGUACCGAGCUCGAAUU

3’T206 Circular EGFP ΔpA (**bold: circularized**)
Group I intron, CVB3 IRES, EGFP, miR-206 target site
GGGAGACCCUCGACCGUCGAUUGUCCACUGGUCAACAAUAGAUGACUUACAACUAAUCG
GAAGGUGCAGAGACUCGACGGGAGCUACCCUAACGUCAAGACGAGGGUAAAGAGAG
UCCAAUUCUCAAAGCCAAUAGGCAGUAGCGAAAGCUGCAAGAGAAUG**AAAAUCCGUUGA**
CCUUAAACGGUCGUGUGGGUUCAAGUCCCUCCACCCCCACGCCGGAAACGCAAUAGC
CGGCGAAUUAAGAGAGAAAAGAAGAGUAAGAAGAAAUAUAAGACACCGGUCGCCACC
UUAAAACAGCCUGUGGGUUGAUCCCACCCACAGGCCCAUUGGGCGCUAGCACUCUGG
UAUCACGGUACCUUUGUGCGCCUGUUUUAUACCCCCUCCCCCAACUGUAACUUAGAAG
UAACACACACCGAUCAACAGUCAGCGUGGCACACCAGCCACGUUUUGAUCAAGCACUU
CUGUUACCCCGGACUGAGUAUCAAUAGACUGCUCACGCGGUUGAAGGAGAAAGCGUU
CGUUAUCCGGCCAACUACUUCGAAAAACCUAGUAACACCGUGGAAGUUGCAGAGUGU
UUCGCUCAGCACUACCCCAGUGUAGAUCAGGUCGAUGAGUCACCGCAUUCCCCACGG
GCGACCGUGGCGGUGGCUGCGUUGGCGGCCUGCCCAUGGGGAAACCCAUGGGACGCU
CUAAUACAGACAUGGUGCGAAGAGUCUAUUGAGCUAGUUGGUAGUCCUCCGGCCCCU
GAAUGCGGCUAAUCCUAACUGCGGAGCACACACCCUCAAGCCAGAGGGCAGUGUGUC
GUAACGGGCAACUCUGCAGCGGAACCGACUACUUUGGGUGUCCGUGUUUCAUUUUAU
UCCUAUACUGGCUGCUUAUGGUGACAAUUGAGAGAUCGUUACCAUAUAGCUAUUGGA
UUGGCCAUCCGGUGACUAAUAGAGCUAUUAUAUCCCUUUGUUGGGUUUAUACCACU
UAGCUUGAAAGAGGUUAAAACAUUACAAUUCAUUGUUAAGUUGAAUACAGCAAAauggg
auccgugagcaagggcgaggagcuguucaccgggguggugcccauccuggucgagcuggacggcgacguaaa
cggccacaaguucagcguguccggcgagggcgagggcgaugccaccuacggcaagcugacccugaaguucauc
ugcaccaccggcaagcugcccgugcccuggcccacccucgugaccacccugaccuacggcgugcagugcuuca
gccgcuaccccgaccacaugaagcagcacgacuucuucaaguccgccaugcccgaaggcuacguccaggagcg
caccaucuucuucaaggacgacggcaacuacaagacccgcgccgaggugaaguucgagggcgacacccuggug
aaccgcaucgagcugaagggcaucgacuucaaggaggacggcaacauccuggggcacaagcuggaguacaacu
acaacagccacaacgucuauaucauggccgacaagcagaagaacggcaucaaggugaacuucaagauccgcca
caacaucgaggacggcagcgugcagcucgccgaccacuaccagcagaacacccccaucggcgacggccccgug
cugcugcccgacaaccacuaccugagcacccaguccgcccugagcaaagaccccaacgagaagcgcgaucacau

gguccugcuggaguucgugaccgccgccgggaucacucucggcauggacgagcuguacaagagaucucauau
gcaucucgagugauagCCACACACUUCCUUACAUUCCAucuagaccuucugcggggcuugccuucug
gccaugcccuucuucucucccuugcaccuguaccucuuggucuuuGAAUAAAGCCUGAGUAGGGGC
UAUUAUGCGUUACCGGCGAGACGCUACGGACUUAAAUAAUUGAGCCUUAAAGAAGAAA
UUCUUUAAGUGGAUGCUCUCAAACUCAGGGAAACCUAAAUCUAGUUAUAGACAAGGCAA
UCCUGAGCCAAGCCGAAGUAGUAAUUAGUAAGACCAGUGGACAAUCGACGGAUAACAGC
AUAUCUAG

3’T206 Circular EGFP +pA (**bold: circularized**)

Group I intron, CVB3 IRES, EGFP, miR-206 target site

GGGAGACCCUCGACCGUCGAUUGUCCACUGGUCAACAAUAGAUGACUUACAACUAAUCG
GAAGGUGCAGAGACUCGACGGGAGCUACCCUAACGUCAAGACGAGGGUAAAGAGAG
UCCAAUUCUCAAAGCCAAUAGGCAGUAGCGAAAGCUGCAAGAGAAUG**AAAAUCCGUUGA**
CCUUAAACGGUCGUGUGGGUUCAAGUCCCUCCACCCCCACGCCGGAAACGCAAUAGC
CGGCGAAUUAAGAGAGAAAAGAAGAGUAAGAAGAAAUAUAAGACACCGGUCGCCACC
UUAAAACAGCCUGUGGGUUGAUCCCACCCACAGGCCCAUUGGGCGCUAGCACUCUGG
UAUCACGGUACCUUUGUGCGCCUGUUUUAUACCCCCUCCCCCAACUGUAACUUAGAAG
UAACACACACCGAUCAACAGUCAGCGUGGCACACCAGCCACGUUUUGAUCAAGCACUU
CUGUUACCCCGGACUGAGUAUCAAUAGACUGCUCACGCGGUUGAAGGAGAAAGCGUU
CGUUAUCCGGCCAACUACUUCGAAAAACCUAGUAACACCGUGGAAGUUGCAGAGUGU
UUCGCUCAGCACUACCCCAGUGUAGAUCAGGUCGAUGAGUCACCGCAUUCCCCACGG
GCGACCGUGGCGGUGGCUGCGUUGGCGGCCUGCCCAUGGGGAAACCCAUGGGACGCU
CUAAUACAGACAUGGUGCGAAGAGUCUAUUGAGCUAGUUGGUAGUCCUCCGGCCCCU
GAAUGCGGCUAAUCCUAACUGCGGAGCACACACCCUCAAGCCAGAGGGCAGUGUGUC
GUAACGGGCAACUCUGCAGCGGAACCGACUACUUUGGGUGUCCGUGUUUCAUUUUAU
UCCUAUACUGGCUGCUUAUGGUGACAAUUGAGAGAUCGUUACCAUAUAGCUAUUGGA
UUGGCCAUCCGGUGACUAAUAGAGCUAUUAUAUCCCUUUGUUGGGUUUAUACCACU
UAGCUUGAAAGAGGUUAAAACAUUACAAUUCAUUGUUAAGUUGAAUACAGCAAAauggg
auccgugagcaagggcgaggagcuguucaccgggguggugcccauccuggucgagcuggacggcgacguaaa
cggccacaaguucagcguguccggcgagggcgagggcgaugccaccuacggcaagcugacccugaaguucauc
ugcaccaccggcaagcugcccgugcccuggcccacccucgugaccacccugaccuacggcgugcagugcuuca
gccgcuaccccgaccacaugaagcagcacgacuucuucaaguccgccaugcccgaaggcuacguccaggagcg
caccaucuucuucaaggacgacggcaacuacaagacccgcgccgaggugaaguucgagggcgacacccuggug
aaccgcaucgagcugaagggcaucgacuucaaggaggacggcaacauccuggggcacaagcuggaguacaacu
acaacagccacaacgucuauaucauggccgacaagcagaagaacggcaucaaggugaacuucaagauccgcca
caacaucgaggacggcagcgugcagcucgccgaccacuaccagcagaacacccccaucggcgacggccccgug

cugcugcccgacaaccacuaccugagcacccaguccgcccugagcaaagaccccaacgagaagcgcgaucacau
gguccugcuggaguucgugaccgccgccgggaucacucucggcauggacgagcuguacaagagaucucauau
gcaucucgagugauagCCACACACUUCCUUACAUUCCAucuagaccuucugcggggcuugccuucug
gccaugcccuucuucucucccuugcaccuguaccucuuggucuuuGAAUAAAGCCUGAGUAGGAAA
AAAAAAAAAAAAAAAAAAAAAAAAAAAAAAAAAAAAAAAAAAAAAAAAAAAAAAAA
AAAAAAAAAAAAAAAAAAAAAAAAAAAAAAAAAAAAAAAAAAAAAAAAAAAAAAAA
AGGCUAUUAUGCGUUACCGGCGAGACGCUACGGACUUAAAUAAUUGAGCCUUAAAGAA
GAAAUUCUUUAAGUGGAUGCUCUCAAACUCAGGGAAACCUAAAUCUAGUUAUAGACAAG
GCAAUCCUGAGCCAAGCCGAAGUAGUAAUUAGUAAGACCAGUGGACAAUCGACGGAUAA
CAGCAUAUCUAGACACAGGAAACAGCUAUGACCAUGAUUACGCCAAGCUUGCAUGCCUG
CAGGUCGACUCUAGAGGAUCCCCGGGUACCGAGCUCGAAUU

4x 5'T206 Circular EGFP +pA (**bold: circularized**)

Group I intron, CVB3 IRES, EGFP, miR-206 target site

GGGAGACCCUCGACCGUCGAUUGUCCACUGGUCAACAAUAGAUGACUUACAACUAAUCG
GAAGGUGCAGAGACUCGACGGGAGCUACCCUAACGUCAAGACGAGGGUAAAGAGAG
UCCAAUUCUCAAAGCCAAUAGGCAGUAGCGAAAGCUGCAAGAGAAUG**AAAAUCCGUUGA**
CCUUAAACGGUCGUGUGGGUUCAAGUCCCUCCACCCCCACGCCGGAAACGCAAUAGC
CGGCGAAUUAAGAGAGAAAAGAAGAGUAAGAAGAAAUAUAAGACACCGGUCCCACAC
ACUUCCUUACAUUCCACCACACACUUCCUUACAUUCCACCACACACUUCCUUACAUUC
CACCACACACUUCCUUACAUUCCAGCCACCUUAAAACAGCCUGUGGGUUGAUCCCACC
CACAGGCCCAUUGGGCGCUAGCACUCUGGUAUCACGGUACCUUUGUGCGCCUGUUUU
AUACCCCCUCCCCCAACUGUAACUUAGAAGUAACACACCGAUCAACAGUCAGCGUG
GCACACCAGCCACGUUUUGAUCAAGCACUUCUGUUACCCCGGACUGAGUAUCAAUAGA
CUGCUCACGCGGUUGAAGGAGAAAGCGUUCGUUAUCCGGCCAACUACUUCGAAAAAC
CUAGUAACACCGUGGAAGUUGCAGAGUGUUUCGCUCAGCACUACCCCAGUGUAGAUC
AGGUCGAUGAGUCACCGCAUUCCCCACGGGCGACCGUGGCGGUGGCUGCGUUGGCGG
CCUGCCCAUGGGGAAACCCAUGGGACGCUCUAAUACAGACAUGGUGCGAAGAGUCUA
UUGAGCUAGUUGGUAGUCCUCCGGCCCCUGAAUGCGGCUAAUCCUAACUGCGGAGCA
CACACCCUCAAGCCAGAGGGCAGUGUGUCGUAACGGGCAACUCUGCAGCGGAACCGA
CUACUUUGGGUGUCCGUGUUUCAUUUUAUUCCUAUACUGGCUGCUUAUGGUGACAAU
UGAGAGAUCGUUACCAUAUAGCUAUUGGAUUGGCCAUCCGGUGACUAAUAGAGCUAU
UAUAUAUCCCUUUGUUGGGUUUAUACCACUUAGCUUGAAAGAGGUUAAAACAUUACAA
UUCAUUGUUAAGUUGAAUACAGCAAAaugggauccgugagcaagggcgaggagcuguucaccggg
guggugcccauccuggucgagcuggacggcgacguaaacggccacaaguucagcguguccggcgagggcgag
ggcgaugccaccuacggcaagcugacccugaaguucaucugcaccaccggcaagcugcccgugcccuggccca

cccucgugaccacccugaccuacggcgugcagugcuucagccgcuaccccgaccacaugaagcagcacgacuu
cuucaaguccgccaugcccgaaggcuacguccaggagcgcaccaucuucuucaaggacgacggcaacuacaag
acccgcgccgaggugaaguucgagggcgacacccuggugaaccgcaucgagcugaagggcaucgacuucaagg
aggacggcaacauccuggggcacaagcuggaguacaacuacaacagccacaacgucuauaucauggccgacaa
gcagaagaacggcaucaaggugaacuucaagauccgccacaacaucgaggacggcagcgugcagcucgccgac
cacuaccagcagaacacccccaucggcgacggccccgugcugcugcccgacaaccacuaccugagcacccaguc
cgcccugagcaaagaccccaacgagaagcgcgaucacauggucc ugcuggaguucgugaccgccgccgggauc
acucucggcauggacgagcuguacaagagaucucauaugcaucucgagugauagucuagaccuucugcgggg
cuugccuucuggccaugcccuucuucucucccuugcaccuguaccucuuggucuuuGAAUAAAGCCUGA
GUAGGAAAAAAAAAAAAAAAAAAAAAAAAAAAAAAAAAAAAAAAAAAAAAAAAAAAAA
AAAAAAAAAAAAAAAAAAAAAAAAAAAAAAAAAAAAAAAAAAAAAAAAAAAAAAAAAA
AAAAAAAAAGGCUAUUAUGCGUUACCGGCGAGACGCUACGGACUUAAAUAAUUGAGCC
UUAAAGAAGAAAUUCUUUAAGUGGAUGCUCUCAAACUCAGGGAAACCUAAAUCUAGUUA
UAGACAAGGCAAUCCUGAGCCAAGCCGAAGUAGUAAUUAGUAAGACCAGUGGACAAUCG
ACGGAUAACAGCAUAUCUAGACACAGGAAACAGCUAUGACCAUGAUUACGCCAAGCUUG
CAUGCCUGCAGGUCGACUCUAGAGGAUCCCCGGGUACCGAGCUCGAAUU

4x 3’T206 Circular EGFP +pA (**bold: circularized**)

Group I intron, CVB3 IRES, EGFP, miR-206 target site

GGGAGACCCUCGACCGUCGAUUGUCCACUGGUCAACAAUAGAUGACUUACAACUAAUCG
GAAGGUGCAGAGACUCGACGGGAGCUACCCUAACGUCAAGACGAGGGUAAAGAGAG
UCCAAUUCUCAAAGCCAAUAGGCAGUAGCGAAAGCUGCAAGAGAAUG**AAAAUCCGUUGA**
CCUUAAACGGUCGUGUGGGUUCAAGUCCCUCCACCCCCACGCCGGAAACGCAAUAGC
CGGCGAAUUAAGAGAGAAAAGAAGAGUAAGAAGAAAUAUAAGACACCGGUCGCCACC
UUAAAACAGCCUGUGGGUUGAUCCCACCCACAGGCCCAUUGGGCGCUAGCACUCUGG
UAUCACGGUACCUUUGUGCGCCUGUUUUAUACCCCCUCCCCCAACUGUAACUUAGAAG
UAACACACACCGAUCAACAGUCAGCGUGGCACACCAGCCACGUUUUGAUCAAGCACUU
CUGUUACCCCGGACUGAGUAUCAAUAGACUGCUCACGCGGUUGAAGGAGAAAGCGUU
CGUUAUCCGGCCAACUACUUCGAAAAACCUAGUAACACCGUGGAAGUUGCAGAGUGU
UUCGCUCAGCACUACCCCAGUGUAGAUCAGGUCGAUGAGUCACCGCAUUCCCCACGG
GCGACCGUGGCGGUGGCUGCGUUGGCGGCCUGCCCAUGGGGAAACCCAUGGGACGCU
CUAAUACAGACAUGGUGCGAAGAGUCUAUUGAGCUAGUUGGUAGUCCUCCGGCCCCU
GAAUGCGGCUAAUCCUAACUGCGGAGCACACACCCUCAAGCCAGAGGGCAGUGUGUC
GUAACGGGCAACUCUGCAGCGGAACCGACUACUUUGGGUGUCCGUGUUUCAUUUUAU
UCCUAUACUGGCUGCUUAUGGUGACAAUUGAGAGAUCGUUACCAUAUAGCUAUUGGA
UUGGCCAUCCGGUGACUAAUAGAGCUAUUAUAUCCCUUUGUUGGGUUUAUACCACU

UAGCUUGAAAGAGGUUAAAACAUUACAAUUCAUUGUUAAGUUGAAUACAGCAAAauggg
auccgugagcaagggcgaggagcuguucaccgggguggugcccauccuggucgagcuggacggcgacguaaa
cggccacaaguucagcguguccggcgagggcgagggcgaugccaccuacggcaagcugacccugaaguucauc
ugcaccaccggcaagcugcccgugcccuggcccacccucgugaccacccugaccuacggcgugcagugcuuca
gccgcuaccccgaccacaugaagcagcacgacuucuucaaguccgccaugcccgaaggcuacguccaggagcg
caccaucuucuucaaggacgacggcaacuacaagacccgcgccgaggugaaguucgagggcgacacccuggug
aaccgcaucgagcugaagggcaucgacuucaaggaggacggcaacauccuggggcacaagcuggaguacaacu
acaacagccacaacgucuauaucauggccgacaagcagaagaacggcaucaaggugaacuucaagauccgcca
caacaucgaggacggcagcgugcagcucgccgaccacuaccagcagaacacccccaucggcgacggccccgug
cugcugcccgacaaccacuaccugagcacccaguccgcccugagcaaagaccccaacgagaagcgcgaucacau
gguccugcuggaguucgugaccgccgccgggaucacucucggcauggacgagcuguacaagagaucucauau
gcaucucgagugauagCCACACACUUCCUUACAUUCCACCACACACUUCCUUACAUUCCAC
CACACACUUCCUUACAUUCCACCACACACUUCCUUACAUUCCAucuagaccuucugcggggc
uugccuucuggccaugcccuucuucucucccuugcaccuguaccucuuggucuuuGAAUAAAGCCUGA
GUAGGAAAAAAAAAAAAAAAAAAAAAAAAAAAAAAAAAAAAAAAAAAAAAAAAAAAA
AAAAAAAAAAAAAAAAAAAAAAAAAAAAAAAAAAAAAAAAAAAAAAAAAAAAAAAAAA
AAAAAAAAAGGCUAUUAUGCGUUACCGGCGAGACGCUACGGACUUAAAUAAUUGAGCC
UUAAAGAAGAAAUUCUUUAAGUGGAUGCUCUCAAACUCAGGGAAACCUAAAUCUAGUUA
UAGACAAGGCAAUCCUGAGCCAAGCCGAAGUAGUAAUUAGUAAGACCAGUGGACAAUCG
ACGGAUAACAGCAUAUCUAGACACAGGAAACAGCUAUGACCAUGAUUACGCCAAGCUUG
CAUGCCUGCAGGUCGACUCUAGAGGAUCCCCGGGUACCGAGCUCGAAUU

2x2 5’&3’T206 Circular EGFP +pA (**bold: circularized**)

Group I intron, CVB3 IRES, EGFP, miR-206 target site

GGGAGACCCUCGACCGUCGAUUGUCCACUGGUCAACAAUAGAUGACUUACAACUAAUCG
GAAGGUGCAGAGACUCGACGGGAGCUACCCUAACGUCAAGACGAGGGUAAAGAGAGAG
UCCAAUUCUCAAAGCCAAUAGGCAGUAGCGAAAGCUGCAAGAGAAUGAAAAUCCGUUGA
CCUUAAACGGUCGUGUGGGUUCAAGUCCCUCCACCCCCACGCCGGAAACGCAAUAGC
CGGCGAAUUAAGAGAGAAAAGAAGAGUAAGAAGAAAUAUAAGACACCGGUCCCACAC
ACUUCCUUACAUUCCACCACACACUUCCUUACAUUCCAGCCACCUUAAAACAGCCUGU
GGGUUGAUCCCACCCACAGGCCCAUUGGGCGCUAGCACUCUGGUAUCACGGUACCUU
UGUGCGCCUGUUUUAUACCCCCUCCCCCAACUGUAACUUAGAAGUAACACACACCGAU
CAACAGUCAGCGUGGCACACCAGCCACGUUUUGAUCAAGCACUUCUGUUACCCCGGA
CUGAGUAUCAAUAGACUGCUCACGCGGUUGAAGGAGAAAGCGUUCGUUAUCCGGCCA
ACUACUUCGAAAAACCUAGUAACACCGUGGAAGUUGCAGAGUGUUUCGCUCAGCACU
ACCCCAGUGUAGAUCAGGUCGAUGAGUCACCGCAUUCCCACGGGCGACCGUGGCGG

UGGCUGCGUUGGCGGCCUGCCCAUGGGGAAACCCAUGGGACGCUCUAAUACAGACAU
GGUGCGAAGAGUCUAUUGAGCUAGUUGGUAGUCCUCCGGCCCCUGAAUGCGGCUAAU
CCUAACUGCGGAGCACACACCCUCAAGCCAGAGGGCAGUGUGUCGUAACGGGCAACU
CUGCAGCGGAACCGACUACUUUGGGUGUCCGUGUUUCAUUUUAUUCCUAUACUGGCU
GCUUAUGGUGACAAUUGAGAGAUCGUUACCAUAUAGCUAUUGGAUUGGCCAUCCGGU
GACUAAUAGAGCUAUUAUAUAUCCCUUUGUUGGGUUUAUACCACUUAGCUUGAAAGAG
GUUAAAACAUUACAAUUCAUUGUUAAGUUGAAUACAGCAAAaugggauccgugagcaagggc
gaggagcuguucaccgggguggugcccauccuggucgagcuggacggcgacguaaacggccacaaguucagc
guguccggcgagggcgagggcgaugccaccuacggcaagcugacccugaaguucaucugcaccaccggcaagc
ugcccgugcccuggcccacccucgugaccacccugaccuacggcgugcagugcuucagccgcuaccccgacca
caugaagcagcacgacuucuucaaguccgccaugcccgaaggcuacguccaggagcgcaccaucuucuucaag
gacgacggcaacuacaagacccgcgccgaggugaaguucgagggcgacacccuggugaaccgcaucgagcuga
agggcaucgacuucaaggaggacggcaacauccuggggcacaagcuggaguacaacuacaacagccacaacgu
cuauaucauggccgacaagcagaagaacggcaucaaggugaacuucaagauccgccacaacaucgaggacggc
agcgugcagcucgccgaccacuaccagcagaacacccccaucggcgacggccccgugcugcugcccgacaacc
acuaccugagcacccaguccgcccugagcaaagaccccaacgagaagcgcgaucacauggucctgcuggaguu
cgugaccgccgccgggaucacucucggcauggacgagcuguacaagagaucucauaugcaucucgagugaua
gCCACACACUUCCUUACAUUCCACCACACACUUCCUUACAUUCCAucuagaccuucugcggg
gcuugccuucuggccaugcccuucuucucucccuugcaccuguaccucuuggucuuuGAAUAAAGCCUG
AGUAGGAAAAAAAAAAAAAAAAAAAAAAAAAAAAAAAAAAAAAAAAAAAAAAAAA
AAAAAAAAAAAAAAAAAAAAAAAAAAAAAAAAAAAAAAAAAAAAAAAAAAAAAAAAAA
AAAAAAAAAAGGCUAUUAUGCGUUACCGGCGAGACGCUACGGACUUAAAUAAUUGAGC
CUUAAAGAAGAAAUUCUUUAAGUGGAUGCUCUCAAACUCAGGGAAACCUAAAUCUAGUU
AUAGACAAGGCAAUCCUGAGCCAAGCCGAAGUAGUAAUUAGUAAGACCAGUGGACAAUC
GACGGAUAACAGCAUAUCUAGACACAGGAAACAGCUAUGACCAUGAUUACGCCAAGCUU
GCAUGCCUGCAGGUCGACUCUAGAGGAUCCCCGGGUACCGAGCUCGAAUU

5’T302a-5p Circular EGFP ΔpA (**bold: circularized**)

Group I intron, CVB3 IRES, EGFP, miR-302a-5p target site

GGGAGACCCUCGACCGUCGAUUGUCCACUGGUCAACAAUAGAUGACUUACAACUAAUCG
GAAGGUGCAGAGACUCGACGGGAGCUACCCUAACGUCAAGACGAGGGUAAAGAGAGAG
UCCAAUUCUCAAAGCCAAUAGGCAGUAGCGAAAGCUGCAAGAGAAUG**AAAAUCCGUUGA**
CCUUAAACGGUCGUGUGGGUUCAAGUCCCUCCACCCCCACGCCGGAAACGCAAUAGC
CGGCGAAUUAAGAGAGAAAAGAAGAGUAAGAAGAAAUAUAAGACACCGGUCAGCAAG
UACAUCCACGUUUAAGUGCCACCUUAAAACAGCCUGUGGGUUGAUCCCACCCACAGGC

CCAUUGGGCGCUAGCACUCUGGUAUCACGGUACCUUUGUGCGCCUGUUUUAUACCCC
CUCCCCCAACUGUAACUUAGAAGUAACACACACCGAUCAACAGUCAGCGUGGCACACC
AGCCACGUUUUGAUCAAGCACUUCUGUUACCCCGGACUGAGUAUCAAUAGACUGCUCA
CGCGGUUGAAGGAGAAAGCGUUCGUUAUCCGGCCAACUACUUCGAAAAACCUAGUAA
CACCGUGGAAGUUGCAGAGUGUUUCGCUCAGCACUACCCCAGUGUAGAUCAGGUCGA
UGAGUCACCGCAUUCCCCACGGGCGACCGUGGCGGUGGCUGCGUUGGCGGCCUGCCC
AUGGGGAAACCCAUGGGACGCUCUAAUACAGACAUGGUGCGAAGAGUCUAUUGAGCU
AGUUGGUAGUCCUCCGGCCCCUGAAUGCGGCUAAUCCUAACUGCGGAGCACACACCC
UCAAGCCAGAGGGCAGUGUGUCGUAACGGGCAACUCUGCAGCGGAACCGACUACUUU
GGGUGUCCGUGUUUCAUUUUAUUCCUAUACUGGCUGCUUAUGGUGACAAUUGAGAGA
UCGUUACCAUAUAGCUAUUGGAUUGGCCAUCCGGUGACUAAUAGAGCUAUUAUAUC
CCUUUGUUGGGUUUAUACCACUUAGCUUGAAAGAGGUUAAAACAUUACAAUUCAUUGU
UAAGUUGAAUACAGCAAAaugggauccgugagcaagggcgaggagcuguucaccgggguggugccca
uccuggucgagcuggacggcgacguaaacggccacaaguucagcguguccggcgagggcgagggcgaugcca
ccuacggcaagcugacccugaaguucaucugcaccaccggcaagcugcccgugcccuggcccacccucgugac
cacccugaccuacggcgugcagugcuucagccgcuaccccgaccacaugaagcagcacgacuucuucaagucc
gccaugcccgaaggcuacguccaggagcgcaccaucuucuucaaggacgacggcaacuacaagacccgcgccg
aggugaaguucgagggcgacacccuggugaaccgcaucgagcugaagggcaucgacuucaaggaggacggca
acauccuggggcacaagcuggaguacaacuacaacagccacaacgucuauaucauggccgacaagcagaagaa
cggcaucaaggugaacuucaagauccgccacaacaucgaggacggcagcgugcagcucgccgaccacuaccag
cagaacacccccaucggcgacggccccgugcugcugcccgacaaccacuaccugagcacccaguccgcccuga
gcaaagaccccaacgagaagcgcgaucacauggucgugcuggaguucgugaccgccgccgggaucacucucgg
cauggacgagcuguacaagagaucucauaugcaucucgagugauagucuagaccuucugcggggcuugccuu
cuggccaugcccuucuucucucccuugcaccuguaccucuuggucuuuGAAUAAAGCCUGAGUAGGG
GCUAUUAUGCGUUACCGGCGAGACGCUACGGACUUAAAUAAUUGAGCCUUAAAGAAGA
AAUUCUUUAAGUGGAUGCUCUCAAACUCAGGGAAACCUAAAUCUAGUUAUAGACAAGGC
AAUCCUGAGCCAAGCCGAAGUAGUAAUUAGUAAGACCAGUGGACAAUCGACGGAUAACA
GCAUAUCUAG

5'T302a-5p Circular EGFP +pA (**bold: circularized**)

Group I intron, CVB3 IRES, EGFP, miR-302a-5p target site

GGGAGACCCUCGACCGUCGAUUGUCCACUGGUCAACAAUAGAUGACUUACAACUAAUCG
GAAGGUGCAGAGACUCGACGGGAGCUACCCUAACGUCAAGACGAGGGUAAAGAGAGAG
UCCAAUUCUCAAAGCCAAUAGGCAGUAGCGAAAGCUGCAAGAGAAUG**AAAAUCCGUUGA**
CCUUAAACGGUCGUGUGGGUUCAAGUCCCUCCACCCCCACGCCGGAAACGCAAUAGC
CGGCGAAUUAAGAGAGAAAAGAAGAGUAAGAAGAAAUAUAAGACACCGGUCAGCAAG

UACAUCCACGUUUAAGUGCCACCUUAAAACAGCCUGUGGGUUGAUCCCACCCACAGGC
CCAUUGGGCGCUAGCACUCUGGUAUCACGGUACCUUUGUGCGCCUGUUUUAUACCCC
CUCCCCCAACUGUAACUUAGAAGUAACACACCGAUCAACAGUCAGCGUGGCACACC
AGCCACGUUUUGAUCAAGCACUUCUGUUACCCCGGACUGAGUAUCAAUAGACUGCUCA
CGCGGUUGAAGGAGAAAGCGUUCGUUAUCCGGCCAACUACUUCGAAAAACCUAGUAA
CACCGUGGAAGUUGCAGAGUGUUUCGCUCAGCACUACCCCAGUGUAGAUCAGGUCGA
UGAGUCACCGCAUUCCCCACGGGCGACCGUGGCGGUGGCUGCGUUGGCGGCCUGCCC
AUGGGGAAACCCAUGGGACGCUCUAAUACAGACAUGGUGCGAAGAGUCUAUUGAGCU
AGUUGGUAGUCCUCCGGCCCCUGAAUGCGGCUAAUCCUAACUGCGGAGCACACACCC
UCAAGCCAGAGGGCAGUGUGUCGUAACGGGCAACUCUGCAGCGGAACCGACUACUUU
GGGUGUCCGUGUUUCAUUUUAUUCCUAUACUGGCUGCUUAUGGUGACAAUUGAGA
UCGUUACCAUAUAGCUAUUGGAUUGGCCAUCCGGUGACUAAUAGAGCUAUUAUAUC
CCUUUGUUGGGUUUAUACCACUUAGCUUGAAAGAGGUUAAAACAUUACAAUUCAUUGU
UAAGUUGAAUACAGCAAAaugggauccgugagcaagggcgaggagcuguucaccgggguggugccca
uccuggucgagcuggacggcgacguaaacggccacaaguucagcguguccggcgagggcgagggcgaugcca
ccuacggcaagcugacccugaaguucaucugcaccaccggcaagcugcccgugcccuggcccacccucgugac
cacccugaccuacggcgugcagugcuucagccgcuaccccgaccacaugaagcagcacgacuucuucaagucc
gccaugcccgaaggcuacguccaggagcgcaccaucuucuucaaggacgacggcaacuacaagacccgcgccg
aggugaaguucgagggcgacacccuggugaaccgcaucgagcugaagggcaucgacuucaaggaggacggca
acauccuggggcacaagcuggaguacaacuacaacagccacaacgucuauaucauggccgacaagcagaagaa
cggcaucaaggugaacuucaagauccgccacaacaucgaggacggcagcgugcagcucgccgaccacuaccag
cagaacacccccaucggcgacggccccgugcugcugcccgacaaccacuaccugagcacccaguccgcccuga
gcaaagaccccaacgagaagcgcgaucacauggucc ugcuggaguucgugaccgccgccgggaucacucucgg
cauggacgagcuguacaagagaucucauaugcaucucgagugauagucuagaccuucugcggggcuugccuu
cuggccaugcccuucuucucucccuugcaccuguaccucuuggucuuuGAAUAAAGCCUGAGUAGGA
AAAAAAAAAAAAAAAAAAAAAAAAAAAAAAAAAAAAAAAAAAAAAAAAAAAAAAAAAAAAAAAA
AAAAAAAAAAAAAAAAAAAAAAAAAAAAAAAAAAAAAAAAAAAAAAAAAAAAAAAAAAAAAAAA
AAAGGCUAUUAUGCGUUACCGGCGAGACGCUACGGACUUAAAUAAUUGAGCCUUAAAG
AAGAAAUUCUUUAAGUGGAUGCUCUCAAACUCAGGGAAACCUAAAUCUAGUUAUAGACA
AGGCAAUCCUGAGCCAAGCCGAAGUAGUAAUUAGUAAGACCAGUGGACAAUCGACGGAU
AACAGCAUAUCUAGACACAGGAAACAGCUAUGACCAUGAUUACGCCAAGCUUGCAUGCC
UGCAGGUCGACUCUAGAGGAUCCCCGGGUACCGAGCUCGAAUU

3’T302*a*-5p Circular EGFP ΔpA (**bold: circularized**)

Group I intron, CVB3 IRES, EGFP, miR-302a-5p target site

GGGAGACCCUCGACCGUCGAUUGUCCACUGGUCAACAAUAGAUGACUUACAACUAAUCG

GAAGGUGCAGAGACUCGACGGGAGCUACCCUAACGUCAAGACGAGGGUAAAGAGAG
UCCAAUUCUCAAAGCCAAUAGGCAGUAGCGAAAGCUGCAAGAGAAUG**AAAAUCCGUUGA**
CCUUAAACGGUCGUGUGGGUUCAAGUCCCUCCACCCCCACGCCGGAAACGCAAUAGC
CGGCGAAUUAAGAGAGAAAAGAAGAGUAAGAAGAAAUAUAAGACACCGGUCGCCACC
UUAAAACAGCCUGUGGGUUGAUCCCACCCACAGGCCCAUUGGGCGCUAGCACUCUGG
UAUCACGGUACCUUUGUGCGCCUGUUUUAUACCCCCUCCCCCAACUGUAACUUAGAAG
UAACACACACCGAUCAACAGUCAGCGUGGCACACCAGCCACGUUUUGAUCAAGCACUU
CUGUUACCCCGGACUGAGUAUCAAUAGACUGCUCACGCGGUUGAAGGAGAAAGCGUU
CGUUAUCCGGCCAACUACUUCGAAAAACCUAGUAACACCGUGGAAGUUGCAGAGUGU
UUCGCUCAGCACUACCCCAGUGUAGAUCAGGUCGAUGAGUCACCGCAUUCCCCACGG
GCGACCGUGGCGGUGGCUGCGUUGGCGGCCUGCCCAUGGGGAAACCCAUGGGACGCU
CUAAUACAGACAUGGUGCGAAGAGUCUAUUGAGCUAGUUGGUAGUCCUCCGGCCCCU
GAAUGCGGCUAAUCCUAACUGCGGAGCACACACCCUCAAGCCAGAGGGCAGUGUGUC
GUAACGGGCAACUCUGCAGCGGAACCGACUACUUUGGGUGUCCGUGUUUCAUUUUAU
UCCUAUACUGGCUGCUUAUGGUGACAAUUGAGAGAUCGUUACCAUAUAGCUAUUGGA
UUGGCCAUCCGGUGACUAAUAGAGCUAUUAUAUCCCUUUGUUGGGUUUAUACCACU
UAGCUUGAAAGAGGUUAAAACAUUACAAUUCAUUGUUAAGUUGAAUACAGCAAAauggg
auccgugagcaagggcgaggagcuguucaccgggguggugcccauccuggucgagcuggacggcgacguaaa
cggccacaaguucagcguguccggcgagggcgagggcgaugccaccuacggcaagcugacccugaaguucauc
ugcaccaccggcaagcugcccgugcccuggcccacccucgugaccacccugaccuacggcgugcagugcuuca
gccgcuaccccgaccacaugaagcagcacgacuucuucaaguccgccaugcccgaaggcuacguccaggagcg
caccaucuucuucaaggacgacggcaacuacaagacccgcgccgaggugaaguucgagggcgacacccuggug
aaccgcaucgagcugaagggcaucgacuucaaggaggacggcaacauccuggggcacaagcuggaguacaacu
acaacagccacaacgucuauaucauggccgacaagcagaagaacggcaucaaggugaacuucaagauccgcca
caacaucgaggacggcagcgugcagcucgccgaccacuaccagcagaacacccccaucggcgacggccccgug
cugcugcccgacaaccacuaccugagcacccaguccgcccugagcaaagaccccaacgagaagcgcgaucacau
gguccugcuggaguucgugaccgccgccgggaucacucucggcauggacgagcuguacaagagaucucauau
gcaucucgagugauagAGCAAGUACAUCCACGUUUAAGUucuagaccuucugcggggcuugccuuc
uggccaugcccuucuucucucccuugcaccuguaccucuuggucuuuGAAUAAAGCCUGAGUAGGGG
CUAUUAUGCGUUACCGGCGAGACGCUACGGACUUAAAUAAUUGAGCCUUAAAGAAGAA
AUUCUUUAAGUGGAUGCUCUCAAACUCAGGGAAACCUAAAUCUAGUUAUAGACAAGGCA
AUCCUGAGCCAAGCCGAAGUAGUAAUUAGUAAGACCAGUGGACAAUCGACGGAUAACAG
CAUAUCUAG

3’T302a-5p Circular EGFP +pA (**bold: circularized**)

Group I intron, CVB3 IRES, EGFP, miR-302a-5p target site

GGGAGACCCUCGACCGUCGAUUGUCCACUGGUCAACAAUAGAUGACUUACAACUAAUCG
GAAGGUGCAGAGACUCGACGGGAGCUACCCUAACGUCAAGACGAGGGUAAAGAGAG
UCCAAUUCUCAAAGCCAAUAGGCAGUAGCGAAAGCUGCAAGAGAAUG**AAAAUCCGUUGA**
CCUUAAACGGUCGUGUGGGUUCAAGUCCCUCCACCCCCACGCCGGAAACGCAAUAGC
CGGCGAAUUAAGAGAGAAAAGAAGAGUAAGAAGAAAUAUAAGACACCGGUCGCCACC
UUAAAACAGCCUGUGGGUUGAUCCCACCCACAGGCCCAUUGGGCGCUAGCACUCUGG
UAUCACGGUACCUUUGUGCGCCUGUUUUAUACCCCCUCCCCCAACUGUAACUUAGAAG
UAACACACACCGAUCAACAGUCAGCGUGGCACACCAGCCACGUUUUGAUCAAGCACUU
CUGUUACCCCGGACUGAGUAUCAAUAGACUGCUCACGCGGUUGAAGGAGAAAGCGUU
CGUUAUCCGGCCAACUACUUCGAAAAACCUAGUAACACCGUGGAAGUUGCAGAGUGU
UUCGCUCAGCACUACCCCAGUGUAGAUCAGGUCGAUGAGUCACCGCAUUCCCCACGG
GCGACCGUGGCGGUGGCUGCGUUGGCGGCCUGCCCAUGGGGAAACCCAUGGGACGCU
CUAAUACAGACAUGGUGCGAAGAGUCUAUUGAGCUAGUUGGUAGUCCUCCGGCCCCU
GAAUGCGGCUAAUCCUAACUGCGGAGCACACACCCUCAAGCCAGAGGGCAGUGUGUC
GUAACGGGCAACUCUGCAGCGGAACCGACUACUUUGGGUGUCCGUGUUUCAUUUUAU
UCCUAUACUGGCUGCUUAUGGUGACAAUUGAGAGAUCGUUACCAUAUAGCUAUUGGA
UUGGCCAUCCGGUGACUAAUAGAGCUAUUAUAUCCCUUUGUUGGGUUUAUACCACU
UAGCUUGAAAGAGGUUAAAACAUUACAAUUCAUUGUUAAGUUGAAUACAGCAAAauggg
auccgugagcaagggcgaggagcuguucaccgggguggugcccauccuggucgagcuggacggcgacguaaa
cggccacaaguucagcguguccggcgagggcgagggcgaugccaccuacggcaagcugacccugaaguucauc
ugcaccaccggcaagcugcccgugcccuggcccacccucgugaccacccugaccuacggcgugcagugcuuca
gccgcuaccccgaccacaugaagcagcacgacuucuucaaguccgccaugcccgaaggcuacguccaggagcg
caccaucuucuucaaggacgacggcaacuacaagacccgcgccgaggugaaguucgagggcgacacccuggug
aaccgcaucgagcugaagggcaucgacuucaaggaggacggcaacauccuggggcacaagcuggaguacaacu
acaacagccacaacgucuauaucauggccgacaagcagaagaacggcaucaaggugaacuucaagauccgcca
caacaucgaggacggcagcgugcagcucgccgaccacuaccagcagaacacccccaucggcgacggccccgug
cugcugcccgacaaccacuaccugagcacccaguccgcccugagcaaagaccccaacgagaagcgcgaucacau
gguccugcuggaguucgugaccgccgccgggaucacucucggcauggacgagcuguacaagagaucucauau
gcaucucgagugauagAGCAAGUACAUCCACGUUUAAGUucuagaccuucugcggggcuugccuuc
uggccaugcccuucuucucucccuugcaccuguaccucuuggucuuuGAAUAAAGCCUGAGUAGGAA
AAAAAAAAAAAAAAAAAAAAAAAAAAAAAAAAAAAAAAAAAAAAAAAAAAAAAAAAAA
AAAAAAAAAAAAAAAAAAAAAAAAAAAAAAAAAAAAAAAAAAAAAAAAAAAAAAAAAA
AAGGCUAUUAUGCGUUACCGGCGAGACGCUACGGACUUAAAUAAUUGAGCCUUAAAGA
AGAAAUUCUUUAAGUGGAUGCUCUCAAACUCAGGGAAACCUAAAUCUAGUUAUAGACAA
GGCAAUCCUGAGCCAAGCCGAAGUAGUAAUUAGUAAGACCAGUGGACAAUCGACGGAUA
ACAGCAUAUCUAGACACAGGAAACAGCUAUGACCAUGAUUACGCCAAGCUUGCAUGCCU

GCAGGUCGACUCUAGAGGAUCCCCGGGUACCGAGCUCGAAUU

4x 5'T302a-5p Circular EGFP +pA (**bold: circularized**)

Group I intron, CVB3 IRES, EGFP, miR-302a-5p target site

GGGAGACCCUCGACCGUCGAUUGUCCACUGGUCAACAAUAGAUGACUUACAACUAAUCG
GAAGGUGCAGAGACUCGACGGGAGCUACCCUAACGUCAAGACGAGGGUAAAGAGAGAG
UCCAAUUCUCAAAGCCAAUAGGCAGUAGCGAAAGCUGCAAGAGAAUG**AAAAUCCGUUGA**
CCUUAAACGGUCGUGUGGGUUCAAGUCCCUCCACCCCCACGCCGGAAACGCAAUAGC
CGGCGAAUUAAGAGAGAAAAGAAGAGUAAGAAGAAAUAUAAGACACCGGUCAGCAAG
UACAUCCACGUUUAAGUAGCAAGUACAUCCACGUUUAAGUAGCAAGUACAUCCACGUU
UAAGUAGCAAGUACAUCCACGUUUAAGUGCCACCUUAAAACAGCCUGUGGGUUGAUC
CCACCCACAGGCCCAUUGGGCGCUAGCACUCUGGUAUCACGGUACCUUUGUGCGCCU
GUUUUAUACCCCCUCCCCCAACUGUAACUUAGAAGUAACACACACCGAUCAACAGUCA
GCGUGGCACACCAGCCACGUUUUGAUCAAGCACUUCUGUUACCCCGGACUGAGUAUC
AAUAGACUGCUCACGCGGUUGAAGGAGAAAGCGUUCGUUAUCCGGCCAACUACUUCG
AAAAACCUAGUAACACCGUGGAAGUUGCAGAGUGUUUCGCUCAGCACUACCCCAGUG
UAGAUCAGGUCGAUGAGUCACCGCAUUCCCCACGGGCGACCGUGGCGGUGGCUGCGU
UGGCGGCCUGCCCAUGGGGAAACCCAUGGGACGCUCUAAUACAGACAUGGUGCGAAG
AGUCUAUUGAGCUAGUUGGUAGUCCUCCGGCCCCUGAAUGCGGCUAAUCCUAACUGC
GGAGCACACACCCUCAAGCCAGAGGGCAGUGUGUCGUAACGGGCAACUCUGCAGCGG
AACCGACUACUUUGGGUGUCCGUGUUUCAUUUUAUUCCUAUACUGGCUGCUUAUGGU
GACAAUUGAGAGAUCGUUACCAUAUAGCUAUUGGAUUGGCCAUCCGGUGACUAAUAG
AGCUAUUAUAUAUCCCUUUGUUGGGUUUAUACCACUUAGCUUGAAAGAGGUUAAAACA
UUACAAUUCAUUGUUAAGUUGAAUACAGCAAAaugggauccgugagcaagggcgaggagcuguu
caccgggguggugcccauccuggucgagcuggacggcgacguaaacggccacaaguucagcguguccggcga
gggcgagggcgaugccaccuacggcaagcugacccugaaguucaucugcaccaccggcaagcugcccgugccc
uggcccacccucgugaccacccugaccuacggcgugcagugcuucagccgcuaccccgaccacaugaagcagc
acgacuucuucaaguccgccaugcccgaaggcuacguccaggagcgcaccaucuucuucaaggacgacggcaa
cuacaagacccgcgccgaggugaaguucgagggcgacacccuggugaaccgcaucgagcugaagggcaucgac
uucaaggaggacggcaacauccuggggcacaagcuggaguacaacuacaacagccacaacgucuauaucaugg
ccgacaagcagaagaacggcaucaaggugaacuucaagauccgccacaacaucgaggacggcagcgugcagcu
cgccgaccacuaccagcagaacacccccaucggcgacggccccgugcugcugcccgacaaccacuaccugagca
cccaguccgcccugagcaaagaccccaacgagaagcgcgaucacauggccugcuggaguucgugaccgccgc
cgggaucacucucggcauggacgagcuguacaagagaucucauaugcaucucgagugauagucuagaccuuc
ugcggggcuugccuucuggccaugcccuucuucucucccuugcaccuguaccucuuggucuuuGAAUAAA
GCCUGAGUAGGAAAAAAAAAAAAAAAAAAAAAAAAAAAAAAAAAAAAAAAAAAAAAAAAAAA

AAAAAAAAAAAAAAAAAAAAAAAAAAAAAAAAAAAAAAAAAAAAAAAAAAAAAAAAAA
AAAAAAAAAAAAAAAGGCUAUUAUGCGUUACCGGCGAGACGCUACGGACUUAAAUAAU
UGAGCCUUAAAGAAGAAAUUCUUUAAGUGGAUGCUCUCAAACUCAGGGAAACCUAAAUC
UAGUUAUAGACAAGGCAAUCCUGAGCCAAGCCGAAGUAGUAAUUAGUAAGACCAGUGGA
CAAUCGACGGAUAACAGCAUAUCUAGACACAGGAAACAGCUAUGACCAUGAUUACGCCA
AGCUUGCAUGCCUGCAGGUCGACUCUAGAGGAUCCCCGGGUACCGAGCUCGAAUU

4x 3’T302a-5p Circular EGFP +pA (**bold: circularized**)

Group I intron, CVB3 IRES, EGFP, miR-302a-5p target site

GGGAGACCCUCGACCGUCGAUUGUCCACUGGUCAACAAUAGAUGACUUACAACUAAUCG
GAAGGUGCAGAGACUCGACGGGAGCUACCCUAACGUCAAGACGAGGGUAAAGAGAG
UCCAAUUCUCAAAGCCAAUAGGCAGUAGCGAAAGCUGCAAGAGAAUG**AAAAUCCGUUGA**
CCUUAAACGGUCGUGUGGGUUCAAGUCCCUCCACCCCCACGCCGGAAACGCAAUAGC
CGGCGAAUUAAGAGAGAAAAGAAGAGUAAGAAGAAAUAUAAGACACCGGUCGCCACC
UUAAAACAGCCUGUGGGUUGAUCCCACCCACAGGCCCAUUGGGCGCUAGCACUCUGG
UAUCACGGUACCUUUGUGCGCCUGUUUUAUACCCCCUCCCCCAACUGUAACUUAGAAG
UAACACACACCGAUCAACAGUCAGCGUGGCACACCAGCCACGUUUUGAUCAAGCACUU
CUGUUACCCCGGACUGAGUAUCAAUAGACUGCUCACGCGGUUGAAGGAGAAAGCGUU
CGUUAUCCGGCCAACUACUUCGAAAAACCUAGUAACACCGUGGAAGUUGCAGAGUGU
UUCGCUCAGCACUACCCCAGUGUAGAUCAGGUCGAUGAGUCACCGCAUUCCCCACGG
GCGACCGUGGCGGUGGCUGCGUUGGCGGCCUGCCCAUGGGGAAACCCAUGGGACGCU
CUAAUACAGACAUGGUGCGAAGAGUCUAUUGAGCUAGUUGGUAGUCCUCCGGCCCCU
GAAUGCGGCUAAUCCUAACUGCGGAGCACACACCCUCAAGCCAGAGGGCAGUGUGUC
GUAACGGGCAACUCUGCAGCGGAACCGACUACUUUGGGUGUCCGUGUUUCAUUUUAU
UCCUAUACUGGCUGCUUAUGGUGACAAUUGAGAGAUCGUUACCAUAUAGCUAUUGGA
UUGGCCAUCCGGUGACUAAUAGAGCUAUUAUAUCCCUUUGUUGGGUUUAUACCACU
UAGCUUGAAAGAGGUUAAAACAUUACAAUUCAUUGUUAAGUUGAAUACAGCAAAauggg
auccgugagcaagggcgaggagcuguucaccgggguggugcccauccuggucgagcuggacggcgacguaaa
cggccacaaguucagcguguccggcgagggcgagggcgaugccaccuacggcaagcugacccugaaguucauc
ugcaccaccggcaagcugcccgugcccuggcccacccucgugaccacccugaccuacggcgugcagugcuuca
gccgcuaccccgaccacaugaagcagcacgacuucuucaaguccgccaugcccgaaggcuacguccaggagcg
caccaucuucuucaaggacgacggcaacuacaagacccgcgccgaggugaaguucgagggcgacacccuggug
aaccgcaucgagcugaagggcaucgacuucaaggaggacggcaacauccuggggcacaagcuggaguacaacu
acaacagccacaacgucuauaucauggccgacaagcagaagaacggcaucaaggugaacuucaagauccgcca
caacaucgaggacggcagcgugcagcucgccgaccacuaccagcagaacacccccaucggcgacggccccgug
cugcugcccgacaaccacuaccugagcacccaguccgcccugagcaaagaccccaacgagaagcgcgaucacau

gguccugcuggaguucgugaccgccgccgggaucacucucggcauggacgagcuguacaagagaucucauau
gcaucucgagugauagAGCAAGUACAUCCACGUUUAAGUAGCAAGUACAUCCACGUUUAAG
UAGCAAGUACAUCCACGUUUAAGUAGCAAGUACAUCCACGUUUAAGUucuagaccuucug
cggggcuugccuucuggccaugcccuucuucucucccuugcaccuguaccucuuggucuuuGAAUAAAGC
CUGAGUAGGAAAAAAAAAAAAAAAAAAAAAAAAAAAAAAAAAAAAAAAAAAAAAAAAA
AAAAAAAAAAAAAAAAAAAAAAAAAAAAAAAAAAAAAAAAAAAAAAAAAAAAAAAAAA
AAAAAAAAAAAAAGGCUAUUAUGCGUUACCGGCGAGACGCUACGGACUUAAAUAAUUG
AGCCUUAAAGAAGAAAUUCUUUAAGUGGAUGCUCUCAAACUCAGGGAAACCUAAAUCUA
GUUAUAGACAAGGCAAUCCUGAGCCAAGCCGAAGUAGUAAUUAGUAAGACCAGUGGACA
AUCGACGGAUAACAGCAUAUCUAGACACAGGAAACAGCUAUGACCAUGAUUACGCCAAG
CUUGCAUGCCUGCAGGUCGACUCUAGAGGAUCCCCGGGUACCGAGCUCGAAUU

2x2 5’&3’T302a-5p Circular EGFP +pA (**bold: circularized**)
Group I intron, CVB3 IRES, EGFP, miR-302a-5p target site
GGGAGACCCUCGACCGUCGAUUGUCCACUGGUCAACAAUAGAUGACUUACAACUAAUCG
GAAGGUGCAGAGACUCGACGGGAGCUACCCUAACGUCAAGACGAGGGUAAAGAGAG
UCCAAUUCUCAAAGCCAAUAGGCAGUAGCGAAAGCUGCAAGAGAAUG**AAAAUCCGUUGA**
CCUUAAACGGUCGUGUGGGUUCAAGUCCCUCCACCCCCACGCCGGAAACGCAAUAGC
CGGCGAAUUAAGAGAGAAAAGAAGAGUAAGAAGAAAUAUAAGACACCGGUCAGCAAG
UACAUCCACGUUUAAGUAGCAAGUACAUCCACGUUUAAGUGCCACCUUAAAACAGCCU
GUGGGUUGAUCCCACCCACAGGCCCAUUGGGCGCUAGCACUCUGGUAUCACGGUACC
UUUGUGCGCCUGUUUUAUACCCCCUCCCCCAACUGUAACUUAGAAGUAACACACACCG
AUCAACAGUCAGCGUGGCACACCAGCCACGUUUUGAUCAAGCACUUCUGUUACCCCG
GACUGAGUAUCAAUAGACUGCUCACGCGGUUGAAGGAGAAAGCGUUCGUUAUCCGGC
CAACUACUUCGAAAAACCUAGUAACACCGUGGAAGUUGCAGAGUGUUUCGCUCAGCA
CUACCCCAGUGUAGAUCAGGUCGAUGAGUCACCGCAUUCCCCACGGGCGACCGUGGC
GGUGGCUGCGUUGGCGGCCUGCCCAUGGGGAAACCCAUGGGACGCUCUAAUACAGAC
AUGGUGCGAAGAGUCUAUUGAGCUAGUUGGUAGUCCUCCGGCCCCUGAAUGCGGCUA
AUCCUAACUGCGGAGCACACACCCUCAAGCCAGAGGGCAGUGUGUCGUAACGGGCAA
CUCUGCAGCGGAACCGACUACUUUGGGUGUCCGUGUUUCAUUUUAUUCCUAUACUGG
CUGCUUAUGGUGACAAUUGAGAGAUCGUUACCAUAUAGCUAUUGGAUUGGCCAUCCG
GUGACUAAUAGAGCUAUUAUAUCCCUUUGUUGGGUUUAUACCACUUAGCUUGAAAG
AGGUUAAAACAUUACAAUUCAUUGUUAAGUUGAAUACAGCAAAaugggauccgugagcaag
ggcgaggagcuguucaccgggguggugcccauccuggucgagcuggacggcgacguaaacggccacaaguuc
agcguguccggcgagggcgagggcgaugccaccuacggcaagcugacccugaaguucaucugcaccaccggca
agcugcccgugcccuggcccacccucgugaccacccugaccuacggcgugcagugcuucagccgcuaccccga

ccacaugaagcagcacgacuucuucaaguccgccaugcccgaaggcuacguccaggagcgcaccaucuucuucaaggacgacggcaacuacaagacccgcgccgaggugaaguucgagggcgacacccuggugaaccgcaucgagcugaagggcaucgacuucaaggaggacggcaacauccuggggcacaagcuggaguacaacuacaacagccacaacgucuauaucauggccgacaagcagaagaacggcaucaaggugaacuucaagauccgccacaacaucgaggacggcagcgugcagcucgccgaccacuaccagcagaacacccccaucggcgacggccccgugcugcugcccgacaaccacuaccugagcacccaguccgcccugagcaaagaccccaacgagaagcgcgaucacaugguccugcugagaguucgugaccgccgccgggaucacucucggcauggacgagcuguacaagagaucucauaugcaucucgagugauagAGCAAGUACAUCCACGUUUAAGUAGCAAGUACAUCCACGUUUAAGUucuagaccuucugcggggcuugccuucuggccaugcccuucuucucucccuugcaccuguaccucuuggucuuuGAAUAAAGCCUGAGUAGGAAAAAAAAAAAAAAAAAAAAAAAAAAAAAAAAAAAAAAAAAAAAAAAAAAAAAAAAAAAAAAAAAAAAAAAAAAAAAAAAAAAAAAAAAAAAAAAAAAAAAAAAAAAAAAAAAAAAAAAAAGGCUAUUAUGCGUUACCGGCGAGACGCUACGGACUUAAAUAAUUGAGCCUUAAAGAAGAAAUUCUUUAAGUGGAUGCUCUCAAACUCAGGGAAACCUAAAUCUAGUUAUAGACAAGGCAAUCCUGAGCCAAGCCGAAGUAGUAAUUAGUAAGACCAGUGGACAAUCGACGGAUAACAGCAUAUCUAGACACAGGAAACAGCUAUGACCAUGAUUACGCCAAGCUUGCAUGCCUGCAGGUCGACUCUAGAGGAUCCCCGGGUACCGAGCUCGAAUU

5’T21-5p Circular EGFP ∆pA (**bold: circularized**)

Group I intron, CVB3 IRES, EGFP, miR-21-5p target site

GGGAGACCCUCGACCGUCGAUUGUCCACUGGUCAACAAUAGAUGACUUACAACUAAUCGGAAGGUGCAGAGACUCGACGGGAGCUACCCUAACGUCAAGACGAGGGUAAAGAGAGUCCAAUUCUCAAAGCCAAUAGGCAGUAGCGAAAGCUGCAAGAGAAUGAAAAUCCGUUGACCUUAAACGGUCGUGUGGGUUCAAGUCCCUCCACCCCCACGCCGGAAACGCAAUAGCCGGCGAAUUAAGAGAGAAAAGAAGAGUAAGAAGAAAUAUAAGACACCGGUCUCAACAUCAGUCUGAUAAGCUAGCCACCUUAAAACAGCCUGUGGGUUGAUCCCACCCACAGGCCCAUUGGGCGCUAGCACUCUGGUAUCACGGUACCUUUGUGCGCCUGUUUUAUACCCCCUCCCCCAACUGUAACUUAGAAGUAACACACACCGAUCAACAGUCAGCGUGGCACACCAGCCACGUUUUGAUCAAGCACUUCUGUUACCCCGGACUGAGUAUCAAUAGACUGCUCACGCGGUUGAAGGAGAAAGCGUUCGUUAUCCGGCCAACUACUUCGAAAAACCUAGUAACACCGUGGAAGUUGCAGAGUGUUUCGCUCAGCACUACCCCAGUGUAGAUCAGGUCGAUGAGUCACCGCAUUCCCCACGGGCGACCGUGGCGGUGGCUGCGUUGGCGGCCUGCCCAUGGGGAAACCCAUGGGACGCUCUAAUACAGACAUGGUGCGAAGAGUCUAUUGAGCUAGUUGGUAGUCCUCCGGCCCCUGAAUGCGGCUAAUCCUAACUGCGGAGCACACACCCUCAAGCCAGAGGGCAGUGUGUCGUAACGGGCAACUCUGCAGCGGAACCGACUACUUUGGGUGUCCGUGUUUCAUUUUAUUCCUAUACUGGCUGCUUAUGGUGACAAUUGAGAGAUCGUUACCAUAUAGCUAUUGGAUUGGCCAUCCGGUGACUAAUAGAGCUAUUAUAUAUC

CCUUUGUUGGGUUUAUACCACUUAGCUUGAAAGAGGUUAAAACAUUACAAUUCAUUGU
UAAGUUGAAUACAGCAAAaugggauccgugagcaagggcgaggagcuguucaccgggguggugccca
uccuggucgagcuggacggcgacguaaacggccacaaguucagcguguccggcgagggcgagggcgaugcca
ccuacggcaagcugacccugaaguucaucugcaccaccggcaagcugcccgugcccuggcccacccucgugac
cacccugaccuacggcgugcagugcuucagccgcuaccccgaccacaugaagcagcacgacuucuucaagucc
gccaugcccgaaggcuacguccaggagcgcaccaucuucuucaaggacgacggcaacuacaagacccgcgccg
aggugaaguucgagggcgacacccuggugaaccgcaucgagcugaagggcaucgacuucaaggaggacggca
acauccuggggcacaagcuggaguacaacuacaacagccacaacgucuauaucauggccgacaagcagaagaa
cggcaucaaggugaacuucaagauccgccacaacaucgaggacggcagcgugcagcucgccgaccacuaccag
cagaacacccccaucggcgacggccccgugcugcugcccgacaaccacuaccugagcacccaguccgcccuga
gcaaagaccccaacgagaagcgcgaucacauggucgugcuggaguucgugaccgccgccgggaucacucucgg
cauggacgagcuguacaagagaucucauaugcaucucgagugauagucuagaccuucugcggggcuugccuu
cuggccaugcccuucuucucucccuugcaccuguaccucuuggucuuuGAAUAAAGCCUGAGUAGGG
GCUAUUAUGCGUUACCGGCGAGACGCUACGGACUUAAAUAAUUGAGCCUUAAAGAAGA
AAUUCUUUAAGUGGAUGCUCUCAAACUCAGGGAAACCUAAAUCUAGUUAUAGACAAGGC
AAUCCUGAGCCAAGCCGAAGUAGUAAUUAGUAAGACCAGUGGACAAUCGACGGAUAACA
GCAUAUCUAG

5’T21-5p Circular EGFP +pA (**bold: circularized**)
Group I intron, CVB3 IRES, EGFP, miR-21-5p target site
GGGAGACCCUCGACCGUCGAUUGUCCACUGGUCAACAAUAGAUGACUUACAACUAAUCG
GAAGGUGCAGAGACUCGACGGGAGCUACCCUAACGUCAAGACGAGGGUAAAGAGAG
UCCAAUUCUCAAAGCCAAUAGGCAGUAGCGAAAGCUGCAAGAGAAUG**AAAAUCCGUUGA**
CCUUAAACGGUCGUGUGGGUUCAAGUCCCUCCACCCCCACGCCGGAAACGCAAUAGC
CGGCGAAUUAAGAGAGAAAAGAAGAGUAAGAAGAAAUAUAAGACACCGGUCUCAACA
UCAGUCUGAUAAGCUAGCCACCUUAAAACAGCCUGUGGGUUGAUCCCACCCACAGGC
CCAUUGGGCGCUAGCACUCUGGUAUCACGGUACCUUUGUGCGCCUGUUUUAUACCCC
CUCCCCCAACUGUAACUUAGAAGUAACACACACCGAUCAACAGUCAGCGUGGCACACC
AGCCACGUUUUGAUCAAGCACUUCUGUUACCCCGGACUGAGUAUCAAUAGACUGCUCA
CGCGGUUGAAGGAGAAAGCGUUCGUUAUCCGGCCAACUACUUCGAAAAACCUAGUAA
CACCGUGGAAGUUGCAGAGUGUUUCGCUCAGCACUACCCCAGUGUAGAUCAGGUCGA
UGAGUCACCGCAUUCCCCACGGGCGACCGUGGCGGUGGCUGCGUUGGCGGCCUGCCC
AUGGGGAAACCCAUGGGACGCUCUAAUACAGACAUGGUGCGAAGAGUCUAUUGAGCU
AGUUGGUAGUCCUCCGGCCCCUGAAUGCGGCUAAUCCUAACUGCGGAGCACACACCC
UCAAGCCAGAGGGCAGUGUGUCGUAACGGGCAACUCUGCAGCGGAACCGACUACUUU
GGGUGUCCGUGUUUCAUUUUAUUCCUAUACUGGCUGCUUAUGGUGACAAUUGAGAGA

UCGUUACCAUAUAGCUAUUGGAUUGGCCAUCCGGUGACUAAUAGAGCUAUUAUAUC
CCUUUGUUGGGUUUAUACCACUUAGCUUGAAAGAGGUUAAAACAUUACAAUUCAUUGU
UAAGUUGAAUACAGCAAAaugggauccgugagcaagggcgaggagcuguucaccgggguggugccca
uccuggucgagcuggacggcgacguaaacggccacaaguucagcguguccggcgagggcgagggcgaugcca
ccuacggcaagcugacccugaaguucaucugcaccaccggcaagcugcccgugcccuggcccacccucgugac
cacccugaccuacggcgugcagugcuucagccgcuaccccgaccacaugaagcagcacgacuucuucaagucc
gccaugcccgaaggcuacguccaggagcgcaccaucuucuucaaggacgacggcaacuacaagacccgcgccg
aggugaaguucgagggcgacacccuggugaaccgcaucgagcugaagggcaucgacuucaaggaggacggca
acauccuggggcacaagcuggaguacaacuacaacagccacaacgucuauaucauggccgacaagcagaagaa
cggcaucaaggugaacuucaagauccgccacaacaucgaggacggcagcgugcagcucgccgaccacuaccag
cagaacacccccaucggcgacggccccgugcugcugcccgacaaccacuaccugagcacccaguccgcccuga
gcaaagaccccaacgagaagcgcgaucacaugguccugcuggaguucgugaccgccgccgggaucacucucgg
cauggacgagcuguacaagagaucucauaugcaucucgagugauagucuagaccuucugcggggcuugccuu
cuggccaugcccuucuucucucccuugcaccuguaccucuuggucuuuGAAUAAAGCCUGAGUAGGA
AAAAAAAAAAAAAAAAAAAAAAAAAAAAAAAAAAAAAAAAAAAAAAAAAAAAAAAAAA
AAAAAAAAAAAAAAAAAAAAAAAAAAAAAAAAAAAAAAAAAAAAAAAAAAAAAAAAAA
AAAGGCUAUUAUGCGUUACCGGCGAGACGCUACGGACUUAAAUAAUUGAGCCUUAAAG
AAGAAAUUCUUUAAGUGGAUGCUCUCAAACUCAGGGAAACCUAAAUCUAGUUAUAGACA
AGGCAAUCCUGAGCCAAGCCGAAGUAGUAAUUAGUAAGACCAGUGGACAAUCGACGGAU
AACAGCAUAUCUAGACACAGGAAACAGCUAUGACCAUGAUUACGCCAAGCUUGCAUGCC
UGCAGGUCGACUCUAGAGGAUCCCCGGGUACCGAGCUCGAAUU

3’T21-5p Circular EGFP ΔpA (**bold: circularized**)

Group I intron, CVB3 IRES, EGFP, miR-21-5p target site

GGGAGACCCUCGACCGUCGAUUGUCCACUGGUCAACAAUAGAUGACUUACAACUAAUCG
GAAGGUGCAGAGACUCGACGGGAGCUACCCUAACGUCAAGACGAGGGUAAAGAGAG
UCCAAUUCUCAAAGCCAAUAGGCAGUAGCGAAAGCUGCAAGAGAAUG**AAAAUCCGUUGA**
CCUUAAACGGUCGUGUGGGUUCAAGUCCCUCCACCCCCACGCCGGAAACGCAAUAGC
CGGCGAAUUAAGAGAGAAAAGAAGAGUAAGAAGAAAUAUAAGACACCGGUCGCCACC
UUAAAACAGCCUGUGGGUUGAUCCCACCCACAGGCCCAUUGGGCGCUAGCACUCUGG
UAUCACGGUACCUUUGUGCGCCUGUUUUAUACCCCCUCCCCCAACUGUAACUUAGAAG
UAACACACACCGAUCAACAGUCAGCGUGGCACACCAGCCACGUUUUGAUCAAGCACUU
CUGUUACCCCGGACUGAGUAUCAAUAGACUGCUCACGCGGUUGAAGGAGAAAGCGUU
CGUUAUCCGGCCAACUACUUCGAAAAACCUAGUAACACCGUGGAAGUUGCAGAGUGU
UUCGCUCAGCACUACCCCAGUGUAGAUCAGGUCGAUGAGUCACCGCAUUCCCCACGG
GCGACCGUGGCGGUGGCUGCGUUGGCGGCCUGCCCAUGGGGAAACCCAUGGGACGCU

**CUAAUACAGACAUGGUGCGAAGAGUCUAUUGAGCUAGUUGGUAGUCCUCCGGCCCCU
GAAUGCGGCUAAUCCUAACUGCGGAGCACACACCCUCAAGCCAGAGGGCAGUGUGUC
GUAACGGGCAACUCUGCAGCGGAACCGACUACUUUGGGUGUCCGUGUUUCAUUUUAU
UCCUAUACUGGCUGCUUAUGGUGACAAUUGAGAGAUCGUUACCAUAUAGCUAUUGGA
UUGGCCAUCCGGUGACUAAUAGAGCUAUUAUAUCCCUUUGUUGGGUUUAUACCACU
UAGCUUGAAAGAGGUUAAAACAUUACAAUUCAUUGUUAAGUUGAAUACAGCAAAauggg
auccgugagcaagggcgaggagcuguucaccgggguggugcccauccuggucgagcuggacggcgacguaaa
cggccacaaguucagcguguccggcgagggcgagggcgaugccaccuacggcaagcugacccugaaguucauc
ugcaccaccggcaagcugcccgugcccuggcccacccucgugaccacccugaccuacggcgugcagugcuuca
gccgcuaccccgaccacaugaagcagcacgacuucuucaaguccgccaugcccgaaggcuacguccaggagcg
caccaucuucuucaaggacgacggcaacuacaagacccgcgccgaggugaaguucgagggcgacacccuggug
aaccgcaucgagcugaagggcaucgacuucaaggaggacggcaacauccuggggcacaagcuggaguacaacu
acaacagccacaacgucuauaucauggccgacaagcagaagaacggcaucaaggugaacuucaagauccgcca
caacaucgaggacggcagcgugcagcucgccgaccacuaccagcagaacacccccaucggcgacggccccgug
cugcugcccgacaaccacuaccugagcacccaguccgcccugagcaaagaccccaacgagaagcgcgaucacau
gguccugcuggaguucgugaccgccgccgggaucacucucggcauggacgagcuguacaagagaucucauau
gcaucucgagugauagUCAACAUCAGUCUGAUAAGCUAucuagaccuucugcggggcuugccuucu
ggccaugcccuucuucucucccuugcaccuguaccucuuggucuuuGAAUAAAGCCUGAGUAGGGG
CUAUUAUGCGUUACCGGCGAGACGCUACGGACUU**AAAUAAUUGAGCCUUAAAGAAGAA
AUUCUUUAAGUGGAUGCUCUCAAACUCAGGGAAACCUAAAUCUAGUUAUAGACAAGGCA
AUCCUGAGCCAAGCCGAAGUAGUAAUUAGUAAGACCAGUGGACAAUCGACGGAUAACAG
CAUAUCUAG

3’T21-5p Circular EGFP +pA (**bold: circularized**)

Group I intron, CVB3 IRES, EGFP, miR-21-5p target site

GGGAGACCCUCGACCGUCGAUUGUCCACUGGUCAACAAUAGAUGACUUACAACUAAUCG
GAAGGUGCAGAGACUCGACGGGAGCUACCCUAACGUCAAGACGAGGGUAAAGAGAG
UCCAAUUCUCAAAGCCAAUAGGCAGUAGCGAAAGCUGCAAGAGAAUG**AAAAUCCGUUGA
CCUUAAACGGUCGUGUGGGUUCAAGUCCCUCCACCCCCACGCCGGAAACGCAAUAGC
CGGCGAAUUAAGAGAGAAAAGAAGAGUAAGAAGAAAUAUAAGACACCGGUCGCCACC
UUAAAACAGCCUGUGGGUUGAUCCCACCCACAGGCCCAUUGGGCGCUAGCACUCUGG
UAUCACGGUACCUUUGUGCGCCUGUUUUAUACCCCCUCCCCCAACUGUAACUUAGAAG
UAACACACACCGAUCAACAGUCAGCGUGGCACACCAGCCACGUUUUGAUCAAGCACUU
CUGUUACCCCGGACUGAGUAUCAAUAGACUGCUCACGCGGUUGAAGGAGAAAGCGUU
CGUUAUCCGGCCAACUACUUCGAAAAACCUAGUAACACCGUGGAAGUUGCAGAGUGU
UUCGCUCAGCACUACCCCAGUGUAGAUCAGGUCGAUGAGUCACCGCAUUCCCCACGG**

GCGACCGUGGCGGUGGCUGCGUUGGCGGCCUGCCCAUGGGGAAACCCAUGGGACGCU
CUAAUACAGACAUGGUGCGAAGAGUCUAUUGAGCUAGUUGGUAGUCCUCCGGCCCCU
GAAUGCGGCUAAUCCUAACUGCGGAGCACACACCCUCAAGCCAGAGGGCAGUGUGUC
GUAACGGGCAACUCUGCAGCGGAACCGACUACUUUGGGUGUCCGUGUUUCAUUUUAU
UCCUAUACUGGCUGCUUAUGGUGACAAUUGAGAGAUCGUUACCAUAUAGCUAUUGGA
UUGGCCAUCCGGUGACUAAUAGAGCUAUUAUAUCCCUUUGUUGGGUUUAUACCACU
UAGCUUGAAAGAGGUUAAAACAUUACAAUUCAUUGUUAAGUUGAAUACAGCAAAauggg
auccgugagcaagggcgaggagcuguucaccgggguggugcccauccuggucgagcuggacggcgacguaaa
cggccacaaguucagcguguccggcgagggcgagggcgaugccaccuacggcaagcugacccugaaguucauc
ugcaccaccggcaagcugcccgugcccuggcccacccucgugaccacccugaccuacggcgugcagugcuuca
gccgcuaccccgaccacaugaagcagcacgacuucuucaaguccgccaugcccgaaggcuacguccaggagcg
caccaucuucuucaaggacgacggcaacuacaagacccgcgccgaggugaaguucgagggcgacacccuggug
aaccgcaucgagcugaagggcaucgacuucaaggaggacggcaacauccuggggcacaagcuggaguacaacu
acaacagccacaacgucuauaucauggccgacaagcagaagaacggcaucaaggugaacuucaagauccgcca
caacaucgaggacggcagcgugcagcucgccgaccacuaccagcagaacacccccaucggcgacggccccgug
cugcugcccgacaaccacuaccugagcacccaguccgcccugagcaaagaccccaacgagaagcgcgaucacau
gguccugcuggaguucgugaccgccgccgggaucacucucggcauggacgagcuguacaagagaucucauau
gcaucucgagugauagUCAACAUCAGUCUGAUAAGCUAucuagaccuucugcggggcuugccuucu
ggccaugcccuucuucucucccuugcaccuguaccucuuggucuuuGAAUAAAGCCUGAGUAGGAAA
AAAAAAAAAAAAAAAAAAAAAAAAAAAAAAAAAAAAAAAAAAAAAAAAAAAAAAAAAA
AAAAAAAAAAAAAAAAAAAAAAAAAAAAAAAAAAAAAAAAAAAAAAAAAAAAAAAAAA
AGGCUAUUAUGCGUUACCGGCGAGACGCUACGGACUUAAAUAAUUGAGCCUUAAAGAA
GAAAUUCUUUAAGUGGAUGCUCUCAAACUCAGGGAAACCUAAAUCUAGUUAUAGACAAG
GCAAUCCUGAGCCAAGCCGAAGUAGUAAUUAGUAAGACCAGUGGACAAUCGACGGAUAA
CAGCAUAUCUAGACACAGGAAACAGCUAUGACCAUGAUUACGCCAAGCUUGCAUGCCUG
CAGGUCGACUCUAGAGGAUCCCCGGGUACCGAGCUCGAAUU

5'T339-5p Circular EGFP ΔpA (**bold: circularized**)

Group I intron, CVB3 IRES, EGFP, miR-339-5p target site

GGGAGACCCUCGACCGUCGAUUGUCCACUGGUCAACAAUAGAUGACUUACAACUAAUCG
GAAGGUGCAGAGACUCGACGGGAGCUACCCUAACGUCAAGACGAGGGUAAAGAGAG
UCCAAUUCUCAAAGCCAAUAGGCAGUAGCGAAAGCUGCAAGAGAAUG**AAAAUCCGUUGA**
CCUUAAACGGUCGUGUGGGUUCAAGUCCCUCCACCCCCACGCCGGAAACGCAAUAGC
CGGCGAAUUAAGAGAGAAAAGAAGAGUAAGAAGAAAUAUAAGACACCGGUCcgugagcu
ccuggaggacagggaGCCACCUUAAAACAGCCUGUGGGUUGAUCCCACCCACAGGCCCAUU
GGGCGCUAGCACUCUGGUAUCACGGUACCUUUGUGCGCCUGUUUUAUACCCCUCCC

CCAACUGUAACUUAGAAGUAACACACACCGAUCAACAGUCAGCGUGGCACACCAGCCA
CGUUUUGAUCAAGCACUUCUGUUACCCCGGACUGAGUAUCAAUAGACUGCUCACGCG
GUUGAAGGAGAAAGCGUUCGUUAUCCGGCCAACUACUUCGAAAAACCUAGUAACACC
GUGGAAGUUGCAGAGUGUUUCGCUCAGCACUACCCCAGUGUAGAUCAGGUCGAUGAG
UCACCGCAUUCCCCACGGGCGACCGUGGCGGUGGCUGCGUUGGCGGCCUGCCCAUGG
GGAAACCCAUGGGACGCUCUAAUACAGACAUGGUGCGAAGAGUCUAUUGAGCUAGUU
GGUAGUCCUCCGGCCCCUGAAUGCGGCUAAUCCUAACUGCGGAGCACACACCCUCAA
GCCAGAGGGCAGUGUGUCGUAACGGGCAACUCUGCAGCGGAACCGACUACUUUGGGU
GUCCGUGUUUCAUUUUAUUCCUAUACUGGCUGCUUAUGGUGACAAUUGAGAGAUCGU
UACCAUAUAGCUAUUGGAUUGGCCAUCCGGUGACUAAUAGAGCUAUUAUAUCCCUU
UGUUGGGUUUAUACCACUUAGCUUGAAAGAGGUUAAAACAUUACAAUUCAUUGUUAAG
UUGAAUACAGCAAAaugggauccgugagcaagggcgaggagcuguucaccgggguggugcccauccug
gucgagcuggacggcgacguaaacggccacaaguucagcguguccggcgagggcgagggcgaugccaccuac
ggcaagcugacccugaaguucaucugcaccaccggcaagcugcccgugcccuggcccacccucgugaccaccc
ugaccuacggcgugcagugcuucagccgcuaccccgaccacaugaagcagcacgacuucuucaaguccgccau
gcccgaaggcuacguccaggagcgcaccaucuucuucaaggacgacggcaacuacaagacccgcgccgaggug
aaguucgagggcgacacccuggugaaccgcaucgagcugaagggcaucgacuucaaggaggacggcaacaucc
uggggcacaagcuggaguacaacuacaacagccacaacgucuauaucauggccgacaagcagaagaacggcau
caaggugaacuucaagauccgccacaacaucgaggacggcagcgugcagcucgccgaccacuaccagcagaac
acccccaucggcgacggccccgugcugcugcccgacaaccacuaccugagcacccaguccgcccugagcaaag
accccaacgagaagcgcgaucacaugguccugcuggaguucgugaccgccgccgggaucacucucggcaugga
cgagcuguacaagagaucucauaugcaucucgagugauagucuagaccuucugcggggcuugccuucuggcc
augcccuucuucucucccuugcaccuguaccucuuggucuuuGAAUAAAGCCUGAGUAGGGGCUAU
UAUGCGUUACCGGCGAGACGCUACGGACUUAAAUAAUUGAGCCUUAAAGAAGAAAUUC
UUUAAGUGGAUGCUCUCAAACUCAGGGAAACCUAAAUCUAGUUAUAGACAAGGCAAUCC
UGAGCCAAGCCGAAGUAGUAAUUAGUAAGACCAGUGGACAAUCGACGGAUAACAGCAUA
UCUAG

5’T339-5p Circular EGFP +pA (**bold: circularized**)

Group I intron, CVB3 IRES, EGFP, miR-339-5p target site

GGGAGACCCUCGACCGUCGAUUGUCCACUGGUCAACAAUAGAUGACUUACAACUAAUCG
GAAGGUGCAGAGACUCGACGGGAGCUACCCUAACGUCAAGACGAGGGUAAAGAGAG
UCCAAUUCUCAAAGCCAAUAGGCAGUAGCGAAAGCUGCAAGAGAAUG**AAAAUCCGUUGA**
CCUUAAACGGUCGUGUGGGUUCAAGUCCCUCCACCCCCACGCCGGAAACGCAAUAGC
CGGCGAAUUAAGAGAGAAAAGAAGAGUAAGAAGAAAUAUAAGACACCGGUCcgugagcu
ccuggaggacagggaGCCACCUUAAAACAGCCUGUGGGUUGAUCCCACCCACAGGCCCAUU

GGGCGCUAGCACUCUGGUAUCACGGUACCUUUGUGCGCCUGUUUUAUACCCCCUCCC
CCAACUGUAACUUAGAAGUAACACACACCGAUCAACAGUCAGCGUGGCACACCAGCCA
CGUUUUGAUCAAGCACUUCUGUUACCCCGGACUGAGUAUCAAUAGACUGCUCACGCG
GUUGAAGGAGAAAGCGUUCGUUAUCCGGCCAACUACUUCGAAAAACCUAGUAACACC
GUGGAAGUUGCAGAGUGUUUCGCUCAGCACUACCCCAGUGUAGAUCAGGUCGAUGAG
UCACCGCAUUCCCCACGGGCGACCGUGGCGGUGGCUGCGUUGGCGGCCUGCCCAUGG
GGAAACCCAUGGGACGCUCUAAUACAGACAUGGUGCGAAGAGUCUAUUGAGCUAGUU
GGUAGUCCUCCGGCCCCUGAAUGCGGCUAAUCCUAACUGCGGAGCACACACCCUCAA
GCCAGAGGGCAGUGUGUCGUAACGGGCAACUCUGCAGCGGAACCGACUACUUUGGGU
GUCCGUGUUUCAUUUUAUUCCUAUACUGGCUGCUUAUGGUGACAAUUGAGAGAUCGU
UACCAUAUAGCUAUUGGAUUGGCCAUCCGGUGACUAAUAGAGCUAUUAUAUCCCUU
UGUUGGGUUUAUACCACUUAGCUUGAAAGAGGUUAAAACAUUACAAUUCAUUGUUAAG
UUGAAUACAGCAAAaugggauccgugagcaagggcgaggagcuguucaccgggguggugcccauccug
gucgagcuggacggcgacguaaacggccacaaguucagcguguccggcgagggcgagggcgaugccaccuac
ggcaagcugacccugaaguucaucugcaccaccggcaagcugcccgugcccuggcccacccucgugaccaccc
ugaccuacggcgugcagugcuucagccgcuaccccgaccacaugaagcagcacgacuucuucaaguccgccau
gcccgaaggcuacguccaggagcgcaccaucuucuucaaggacgacggcaacuacaagacccgcgccgaggug
aaguucgagggcgacacccuggugaaccgcaucgagcugaagggcaucgacuucaaggaggacggcaacaucc
uggggcacaagcuggaguacaacuacaacagccacaacgucuauaucauggccgacaagcagaagaacggcau
caaggugaacuucaagauccgccacaacaucgaggacggcagcgugcagcucgccgaccacuaccagcagaac
acccccaucggcgacggccccgugcugcugcccgacaaccacuaccugagcacccaguccgcccugagcaaag
accccaacgagaagcgcgaucacauggccugcuggaguucgugaccgccgccgggaucacucucggcaugga
cgagcuguacaagagaucucauaugcaucucgagugauagucuagaccuucugcggggcuugccuucuggcc
augcccuucuucucucccuugcaccuguaccucuuggucuuuGAAUAAAGCCUGAGUAGGAAAAAA
AAAAAAAAAAAAAAAAAAAAAAAAAAAAAAAAAAAAAAAAAAAAAAAAAAAAAAAAAA
AAAAAAAAAAAAAAAAAAAAAAAAAAAAAAAAAAAAAAAAAAAAAAAAAAAAAAAAGG
CUAUUAUGCGUUACCGGCGAGACGCUACGGACUUAAAUAAUUGAGCCUUAAAGAAGAA
AUUCUUUAAGUGGAUGCUCUCAAACUCAGGGAAACCUAAAUCUAGUUAUAGACAAGGCA
AUCCUGAGCCAAGCCGAAGUAGUAAUUAGUAAGACCAGUGGACAAUCGACGGAUAACAG
CAUAUCUAGACACAGGAAACAGCUAUGACCAUGAUUACGCCAAGCUUGCAUGCCUGCAG
GUCGACUCUAGAGGAUCCCCGGGUACCGAGCUCGAAUU

3'T339-5p Circular EGFP ΔpA (**bold: circularized**)

Group I intron, CVB3 IRES, EGFP, miR-339-5p target site

GGGAGACCCUCGACCGUCGAUUGUCCACUGGUCAACAAUAGAUGACUUACAACUAAUCG
GAAGGUGCAGAGACUCGACGGGAGCUACCCUAACGUCAAGACGAGGGUAAAGAGAG

UCCAAUUCUCAAAGCCAAUAGGCAGUAGCGAAAGCUGCAAGAGAAUG**AAAAUCCGUUGA**
CCUUAAACGGUCGUGUGGGUUCAAGUCCCUCCACCCCCACGCCGGAAACGCAAUAGC
CGGCGAAUUAAGAGAGAAAAGAAGAGUAAGAAGAAAUAUAAGACACCGGUCGCCACC
UUAAAACAGCCUGUGGGUUGAUCCCACCCACAGGCCCAUUGGGCGCUAGCACUCUGG
UAUCACGGUACCUUUGUGCGCCUGUUUUAUACCCCCUCCCCCAACUGUAACUUAGAAG
UAACACACACCGAUCAACAGUCAGCGUGGCACACCAGCCACGUUUUGAUCAAGCACUU
CUGUUACCCCGGACUGAGUAUCAAUAGACUGCUCACGCGGUUGAAGGAGAAAGCGUU
CGUUAUCCGGCCAACUACUUCGAAAAACCUAGUAACACCGUGGAAGUUGCAGAGUGU
UUCGCUCAGCACUACCCCAGUGUAGAUCAGGUCGAUGAGUCACCGCAUUCCCCACGG
GCGACCGUGGCGGUGGCUGCGUUGGCGGCCUGCCCAUGGGGAAACCCAUGGGACGCU
CUAAUACAGACAUGGUGCGAAGAGUCUAUUGAGCUAGUUGGUAGUCCUCCGGCCCCU
GAAUGCGGCUAAUCCUAACUGCGGAGCACACACCCUCAAGCCAGAGGGCAGUGUGUC
GUAACGGGCAACUCUGCAGCGGAACCGACUACUUUGGGUGUCCGUGUUUCAUUUUAU
UCCUAUACUGGCUGCUUAUGGUGACAAUUGAGAGAUCGUUACCAUAUAGCUAUUGGA
UUGGCCAUCCGGUGACUAAUAGAGCUAUUAUAUCCCUUUGUUGGGUUUAUACCACU
UAGCUUGAAAGAGGUUAAAACAUUACAAUUCAUUGUUAAGUUGAAUACAGCAAAauggg
auccgugagcaagggcgaggagcuguucaccgggguggugcccauccuggucgagcuggacggcgacguaaa
cggccacaaguucagcguguccggcgagggcgagggcgaugccaccuacggcaagcugacccugaaguucauc
ugcaccaccggcaagcugcccgugcccuggcccacccucgugaccacccugaccuacggcgugcagugcuuca
gccgcuaccccgaccacaugaagcagcacgacuucuucaaguccgccaugcccgaaggcuacguccaggagcg
caccaucuucuucaaggacgacggcaacuacaagacccgcgccgaggugaaguucgagggcgacacccuggug
aaccgcaucgagcugaagggcaucgacuucaaggaggacggcaacauccuggggcacaagcuggaguacaacu
acaacagccacaacgucuauaucauggccgacaagcagaagaacggcaucaaggugaacuucaagauccgcca
caacaucgaggacggcagcgugcagcucgccgaccacuaccagcagaacacccccaucggcgacggccccgug
cugcugcccgacaaccacuaccugagcacccaguccgcccugagcaaagaccccaacgagaagcgcgaucacau
gguccugcuggaguucgugaccgccgccgggaucacucucggcauggacgagcuguacaagagaucucauau
gcaucucgagugauagcgugagcuccuggaggacagggaucuagaccuucugcggggcuugccuucuggcca
ugcccuucuucucucccuugcaccuguaccucuuggucuuuGAAUAAAGCCUGAGUAGGGGCUAU
UAUGCGUUACCGGCGAGACGCUACGGACUUAAAUAAUUGAGCCUUAAAGAAGAAAUUC
UUUAAGUGGAUGCUCUCAAACUCAGGGAAACCUAAAUCUAGUUAUAGACAAGGCAAUCC
UGAGCCAAGCCGAAGUAGUAAUUAGUAAGACCAGUGGACAAUCGACGGAUAACAGCAUA
UCUAG

3’T339-5p Circular EGFP +pA (**bold: circularized**)

Group I intron, CVB3 IRES, EGFP, miR-339-5p target site

GGGAGACCCUCGACCGUCGAUUGUCCACUGGUCAACAAUAGAUGACUUACAACUAAUCG

GAAGGUGCAGAGACUCGACGGGAGCUACCCUAACGUCAAGACGAGGGUAAAGAGAG
UCCAAUUCUCAAAGCCAAUAGGCAGUAGCGAAAGCUGCAAGAGAAUG**AAAAUCCGUUGA**
CCUUAAACGGUCGUGUGGGUUCAAGUCCCUCCACCCCCACGCCGGAAACGCAAUAGC
CGGCGAAUUAAGAGAGAAAAGAAGAGUAAGAAGAAAUAUAAGACACCGGUCGCCACC
UUAAAACAGCCUGUGGGUUGAUCCCACCCACAGGCCCAUUGGGCGCUAGCACUCUGG
UAUCACGGUACCUUUGUGCGCCUGUUUUAUACCCCCUCCCCCAACUGUAACUUAGAAG
UAACACACACCGAUCAACAGUCAGCGUGGCACACCAGCCACGUUUUGAUCAAGCACUU
CUGUUACCCCGGACUGAGUAUCAAUAGACUGCUCACGCGGUUGAAGGAGAAAGCGUU
CGUUAUCCGGCCAACUACUUCGAAAAACCUAGUAACACCGUGGAAGUUGCAGAGUGU
UUCGCUCAGCACUACCCCAGUGUAGAUCAGGUCGAUGAGUCACCGCAUUCCCCACGG
GCGACCGUGGCGGUGGCUGCGUUGGCGGCCUGCCCAUGGGGAAACCCAUGGGACGCU
CUAAUACAGACAUGGUGCGAAGAGUCUAUUGAGCUAGUUGGUAGUCCUCCGGCCCCU
GAAUGCGGCUAAUCCUAACUGCGGAGCACACACCCUCAAGCCAGAGGGCAGUGUGUC
GUAACGGGCAACUCUGCAGCGGAACCGACUACUUUGGGUGUCCGUGUUUCAUUUUAU
UCCUAUACUGGCUGCUUAUGGUGACAAUUGAGAGAUCGUUACCAUAUAGCUAUUGGA
UUGGCCAUCCGGUGACUAAUAGAGCUAUUAUAUCCCUUUGUUGGGUUUAUACCACU
UAGCUUGAAAGAGGUUAAAACAUUACAAUUCAUUGUUAAGUUGAAUACAGCAAAauggg
auccgugagcaagggcgaggagcuguucaccgggguggugcccauccuggucgagcuggacggcgacguaaa
cggccacaaguucagcguguccggcgagggcgagggcgaugccaccuacggcaagcugacccugaaguucauc
ugcaccaccggcaagcugcccgugcccuggcccacccucgugaccacccugaccuacggcgugcagugcuuca
gccgcuaccccgaccacaugaagcagcacgacuucuucaaguccgccaugcccgaaggcuacguccaggagcg
caccaucuucuucaaggacgacggcaacuacaagacccgcgccgaggugaaguucgagggcgacacccuggug
aaccgcaucgagcugaagggcaucgacuucaaggaggacggcaacauccuggggcacaagcuggaguacaacu
acaacagccacaacgucuauaucauggccgacaagcagaagaacggcaucaaggugaacuucaagauccgcca
caacaucgaggacggcagcgugcagcucgccgaccacuaccagcagaacacccccaucggcgacggccccgug
cugcugcccgacaaccacuaccugagcacccaguccgcccugagcaaagaccccaacgagaagcgcgaucacau
gguccugcuggaguucgugaccgccgccgggaucacucucggcauggacgagcuguacaagagaucucauau
gcaucucgagugauagcgugagcuccuggaggacagggaucuagaccuucugcggggcuugccuucuggcca
ugcccuucuucucucccuugcaccuguaccucuuggucuuuGAAUAAAGCCUGAGUAGGAAAAAAA
AAAAAAAAAAAAAAAAAAAAAAAAAAAAAAAAAAAAAAAAAAAAAAAAAAAAAAAAAA
AAAAAAAAAAAAAAAAAAAAAAAAAAAAAAAAAAAAAAAAAAAAAAAAAAAAAAAGGC
UAUUAUGCGUUACCGGCGAGACGCUACGGACUUAAAUAAUUGAGCCUUAAAGAAGAAA
UUCUUUAAGUGGAUGCUCUCAAACUCAGGGAAACCUAAAUCUAGUUAUAGACAAGGCAA
UCCUGAGCCAAGCCGAAGUAGUAAUUAGUAAGACCAGUGGACAAUCGACGGAUAACAGC
AUAUCUAGACACAGGAAACAGCUAUGACCAUGAUUACGCCAAGCUUGCAUGCCUGCAGG
UCGACUCUAGAGGAUCCCCGGGUACCGAGCUCGAAUU

5’T17-5p Circular EGFP ΔpA (**bold: circularized**)

Group I intron, CVB3 IRES, EGFP, miR-17-5p target site

GGGAGACCCUCGACCGUCGAUUGUCCACUGGUCAACAAUAGAUGACUUACAACUAAUCG
GAAGGUGCAGAGACUCGACGGGAGCUACCCUAACGUCAAGACGAGGGUAAAGAGAGAG
UCCAAUUCUCAAAGCCAAUAGGCAGUAGCGAAAGCUGCAAGAGAAUG**AAAAUCCGUUGA**
CCUUAAACGGUCGUGUGGGUUCAAGUCCCUCCACCCCCACGCCGGAAACGCAAUAGC
CGGCGAAUUAAGAGAGAAAAGAAGAGUAAGAAGAAAUAUAAGACACCGGUCcuaccugc
acuguaagcacuuugGCCACCUUAAAACAGCCUGUGGGUUGAUCCCACCCACAGGCCCAUU
GGGCGCUAGCACUCUGGUAUCACGGUACCUUUGUGCGCCUGUUUUAUACCCCCUCCC
CCAACUGUAACUUAGAAGUAACACACACCGAUCAACAGUCAGCGUGGCACACCAGCCA
CGUUUUGAUCAAGCACUUCUGUUACCCCGGACUGAGUAUCAAUAGACUGCUCACGCG
GUUGAAGGAGAAAGCGUUCGUUAUCCGGCCAACUACUUCGAAAAACCUAGUAACACC
GUGGAAGUUGCAGAGUGUUUCGCUCAGCACUACCCCAGUGUAGAUCAGGUCGAUGAG
UCACCGCAUUCCCCACGGGCGACCGUGGCGGUGGCUGCGUUGGCGGCCUGCCCAUGG
GGAAACCCAUGGGACGCUCUAAUACAGACAUGGUGCGAAGAGUCUAUUGAGCUAGUU
GGUAGUCCUCCGGCCCCUGAAUGCGGCUAAUCCUAACUGCGGAGCACACACCCUCAA
GCCAGAGGGCAGUGUGUCGUAACGGGCAACUCUGCAGCGGAACCGACUACUUUGGGU
GUCCGUGUUUCAUUUUAUUCCUAUACUGGCUGCUUAUGGUGACAAUUGAGAGAUCGU
UACCAUAUAGCUAUUGGAUUGGCCAUCCGGUGACUAAUAGAGCUAUUAUAUCCCUU
UGUUGGGUUUAUACCACUUAGCUUGAAAGAGGUUAAAACAUUACAAUUCAUUGUUAAG
UUGAAUACAGCAAAaugggauccgugagcaagggcgaggagcuguucaccgggguggugcccauccug
gucgagcuggacggcgacguaaacggccacaaguucagcguguccggcgagggcgagggcgaugccaccuac
ggcaagcugacccugaaguucaucugcaccaccggcaagcugcccgugcccuggcccacccucgugaccaccc
ugaccuacggcgugcagugcuucagccgcuaccccgaccacaugaagcagcacgacuucuucaaguccgccau
gcccgaaggcuacguccaggagcgcaccaucuucuucaaggacgacggcaacuacaagacccgcgccgaggug
aaguucgagggcgacacccuggugaaccgcaucgagcugaagggcaucgacuucaaggaggacggcaacaucc
uggggcacaagcuggaguacaacuacaacagccacaacgucuauaucauggccgacaagcagaagaacggcau
caaggugaacuucaagauccgccacaacaucgaggacggcagcgugcagcucgccgaccacuaccagcagaac
acccccaucggcgacggccccgugcugcugcccgacaaccacuaccugagcacccaguccgcccugagcaaag
accccaacgagaagcgcgaucacauggucugcuggaguucgugaccgccgccgggaucacucucggcaugga
cgagcuguacaagagaucucauaugcaucucgagugauagucuagaccuucugcggggcuugccuucuggcc
augcccuucuucucucccuugcaccuguaccucuuggucuuuGAAUAAAGCCUGAGUAGGGGCUAU
UAUGCGUUACCGGCGAGACGCUACGGACUUAAAUAAUUGAGCCUUAAAGAAGAAAUUC
UUUAAGUGGAUGCUCUCAAACUCAGGGAAACCUAAAUCUAGUUAUAGACAAGGCAAUCC
UGAGCCAAGCCGAAGUAGUAAUUAGUAAGACCAGUGGACAAUCGACGGAUAACAGCAUA

UCUAG

5’T17-5p Circular EGFP +pA (**bold: circularized**)

Group I intron, CVB3 IRES, EGFP, miR-17-5p target site

GGGAGACCCUCGACCGUCGAUUGUCCACUGGUCAACAAUAGAUGACUUACAACUAAUCG
GAAGGUGCAGAGACUCGACGGGAGCUACCCUAACGUCAAGACGAGGGUAAAGAGAG
UCCAAUUCUCAAAGCCAAUAGGCAGUAGCGAAAGCUGCAAGAGAAUG**AAAAUCCGUUGA**
CCUUAAACGGUCGUGUGGGUUCAAGUCCCUCCACCCCCACGCCGGAAACGCAAUAGC
CGGCGAAUUAAGAGAGAAAAGAAGAGUAAGAAGAAAUAUAAGACACCGGUCcuaccugc
acuguaagcacuuugGCCACCUUAAAACAGCCUGUGGGUUGAUCCCACCCACAGGCCCAUU
GGGCGCUAGCACUCUGGUAUCACGGUACCUUUGUGCGCCUGUUUUAUACCCCCUCCC
CCAACUGUAACUUAGAAGUAACACACACCGAUCAACAGUCAGCGUGGCACACCAGCCA
CGUUUUGAUCAAGCACUUCUGUUACCCCGGACUGAGUAUCAAUAGACUGCUCACGCG
GUUGAAGGAGAAAGCGUUCGUUAUCCGGCCAACUACUUCGAAAAACCUAGUAACACC
GUGGAAGUUGCAGAGUGUUUCGCUCAGCACUACCCCAGUGUAGAUCAGGUCGAUGAG
UCACCGCAUUCCCCACGGGCGACCGUGGCGGUGGCUGCGUUGGCGGCCUGCCCAUGG
GGAAACCCAUGGGACGCUCUAAUACAGACAUGGUGCGAAGAGUCUAUUGAGCUAGUU
GGUAGUCCUCCGGCCCCUGAAUGCGGCUAAUCCUAACUGCGGAGCACACACCCUCAA
GCCAGAGGGCAGUGUGUCGUAACGGGCAACUCUGCAGCGGAACCGACUACUUUGGGU
GUCCGUGUUUCAUUUUAUUCCUAUACUGGCUGCUUAUGGUGACAAUUGAGAGAUCGU
UACCAUAUAGCUAUUGGAUUGGCCAUCCGGUGACUAAUAGAGCUAUUAUAUCCCUU
UGUUGGGUUUAUACCACUUAGCUUGAAAGAGGUUAAAACAUUACAAUUCAUUGUUAAG
UUGAAUACAGCAAAaugggauccgugagcaagggcgaggagcuguucaccgggguggugcccauccug
gucgagcuggacggcgacguaaacggccacaaguucagcguguccggcgagggcgagggcgaugccaccuac
ggcaagcugacccugaaguucaucugcaccaccggcaagcugcccgugcccuggcccacccucgugaccaccc
ugaccuacggcgugcagugcuucagccgcuaccccgaccacaugaagcagcacgacuucuucaaguccgccau
gcccgaaggcuacguccaggagcgcaccaucuucuucaaggacgacggcaacuacaagacccgcgccgaggug
aaguucgagggcgacacccuggugaaccgcaucgagcugaagggcaucgacuucaaggaggacggcaacaucc
uggggcacaagcuggaguacaacuacaacagccacaacgucuauaucauggccgacaagcagaagaacggcau
caaggugaacuucaagauccgccacaacaucgaggacggcagcgugcagcucgccgaccacuaccagcagaac
acccccaucggcgacggccccgugcugcugcccgacaaccacuaccugagcacccaguccgcccugagcaaag
accccaacgagaagcgcgaucacaugguccugcuggaguucgugaccgccgccgggaucacucucggcaugga
cgagcuguacaagagaucucaugcaucucgagugauagucuagaccuucugcggggcuugccuucuggcc
augcccuucuucucucccuugcaccuguaccucuuggucuuuGAAUAAAGCCUGAGUAGGAAAAAA
AAAAAAAAAAAAAAAAAAAAAAAAAAAAAAAAAAAAAAAAAAAAAAAAAAAAAAAAAA
AAAAAAAAAAAAAAAAAAAAAAAAAAAAAAAAAAAAAAAAAAAAAAAAAAAAAAAAGG

CUAUUAUGCGUUACCGGCGAGACGCUACGGACUUAAAUAAUUGAGCCUUAAAGAAGAA
AUUCUUUAAGUGGAUGCUCUCAAACUCAGGGAAACCUAAAUCUAGUUAUAGACAAGGCA
AUCCUGAGCCAAGCCGAAGUAGUAAUUAGUAAGACCAGUGGACAAUCGACGGAUAACAG
CAUAUCUAGACACAGGAAACAGCUAUGACCAUGAUUACGCCAAGCUUGCAUGCCUGCAG
GUCGACUCUAGAGGAUCCCCGGGUACCGAGCUCGAAUU

3'T17-5p Circular EGFP ΔpA (**bold: circularized**)

Group I intron, CVB3 IRES, EGFP, miR-17-5p target site

GGGAGACCCUCGACCGUCGAUUGUCCACUGGUCAACAAUAGAUGACUUACAACUAAUCG
GAAGGUGCAGAGACUCGACGGGAGCUACCCUAACGUCAAGACGAGGGUAAAGAGAGAG
UCCAAUUCUCAAAGCCAAUAGGCAGUAGCGAAAGCUGCAAGAGAAUG**AAAAUCCGUUGA
CCUUAAACGGUCGUGUGGGUUCAAGUCCCUCCACCCCCACGCCGGAAACGCAAUAGC
CGGCGAAUUAAGAGAGAAAAGAAGAGUAAGAAGAAAUAUAAGACACCGGUCGCCACC
UUAAAACAGCCUGUGGGUUGAUCCCACCCACAGGCCCAUUGGGCGCUAGCACUCUGG
UAUCACGGUACCUUUGUGCGCCUGUUUUAUACCCCCUCCCCCAACUGUAACUUAGAAG
UAACACACACCGAUCAACAGUCAGCGUGGCACACCAGCCACGUUUUGAUCAAGCACUU
CUGUUACCCCGGACUGAGUAUCAAUAGACUGCUCACGCGGUUGAAGGAGAAAGCGUU
CGUUAUCCGGCCAACUACUUCGAAAAACCUAGUAACACCGUGGAAGUUGCAGAGUGU
UUCGCUCAGCACUACCCCAGUGUAGAUCAGGUCGAUGAGUCACCGCAUUCCCCACGG
GCGACCGUGGCGGUGGCUGCGUUGGCGGCCUGCCCAUGGGGAAACCCAUGGGACGCU
CUAAUACAGACAUGGUGCGAAGAGUCUAUUGAGCUAGUUGGUAGUCCUCCGGCCCCU
GAAUGCGGCUAAUCCUAACUGCGGAGCACACACCCUCAAGCCAGAGGGCAGUGUGUC
GUAACGGGCAACUCUGCAGCGGAACCGACUACUUUGGGUGUCCGUGUUUCAUUUUAU
UCCUAUACUGGCUGCUUAUGGUGACAAUUGAGAGAUCGUUACCAUAUAGCUAUUGGA
UUGGCCAUCCGGUGACUAAUAGAGCUAUUAUAUCCCUUUGUUGGGUUUAUACCACU
UAGCUUGAAAGAGGUUAAAACAUUACAAUUCAUUGUUAAGUUGAAUACAGCAAAauggg
auccgugagcaagggcgaggagcuguucaccgggguggugcccauccuggucgagcuggacggcgacguaaa
cggccacaaguucagcguguccggcgagggcgagggcgaugccaccuacggcaagcugacccugaaguucauc
ugcaccaccggcaagcugcccgugcccuggcccacccucgugaccacccugaccuacggcgugcagugcuuca
gccgcuaccccgaccacaugaagcagcacgacuucuucaaguccgccaugcccgaaggcuacguccaggagcg
caccaucuucuucaaggacgacggcaacuacaagacccgcgccgaggugaaguucgagggcgacacccuggug
aaccgcaucgagcugaagggcaucgacuucaaggaggacggcaacauccuggggcacaagcuggaguacaacu
acaacagccacaacgucuauaucauggccgacaagcagaagaacggcaucaaggugaacuucaagauccgcca
caacaucgaggacggcagcgugcagcucgccgaccacuaccagcagaacacccccaucggcgacggccccgug
cugcugcccgacaaccacuaccugagcacccaguccgcccugagcaaagaccccaacgagaagcgcgaucacau
gguccugcuggaguucgugaccgccgccgggaucacucucggcauggacgagcuguacaagagaucucauau**

**gcaucucgagugauagcuaccugcacuguaagcacuuugucuagaccuucugcggggcuugccuucuggcca
ugcccuucuucucucccuugcaccuguaccucuuggucuuuGAAUAAAGCCUGAGUAGGGGCUAU
UAUGCGUUACCGGCGAGACGCUACGGACUU**AAAUAAUUGAGCCUUAAAGAAGAAAUUC
UUUAAGUGGAUGCUCUCAAACUCAGGGAAACCUAAAUCUAGUUAUAGACAAGGCAAUCC
UGAGCCAAGCCGAAGUAGUAAUUAGUAAGACCAGUGGACAAUCGACGGAUAACAGCAUA
UCUAG

3'T17-5p Circular EGFP +pA (**bold: circularized**)

Group I intron, CVB3 IRES, EGFP, miR-17-5p target site

GGGAGACCCUCGACCGUCGAUUGUCCACUGGUCAACAAUAGAUGACUUACAACUAAUCG
GAAGGUGCAGAGACUCGACGGGAGCUACCCUAACGUCAAGACGAGGGUAAAGAGAG
UCCAAUUCUCAAAGCCAAUAGGCAGUAGCGAAAGCUGCAAGAGAAUG**AAAAUCCGUUGA
CCUUAAACGGUCGUGUGGGUUCAAGUCCCUCCACCCCCACGCCGGAAACGCAAUAGC
CGGCGAAUUAAGAGAGAAAAGAAGAGUAAGAAGAAAUAUAAGACACCGGUCGCCACC
UUAAAACAGCCUGUGGGUUGAUCCCACCCACAGGCCCAUUGGGCGCUAGCACUCUGG
UAUCACGGUACCUUUGUGCGCCUGUUUUAUACCCCCUCCCCCAACUGUAACUUAGAAG
UAACACACACCGAUCAACAGUCAGCGUGGCACACCAGCCACGUUUUGAUCAAGCACUU
CUGUUACCCCGGACUGAGUAUCAAUAGACUGCUCACGCGGUUGAAGGAGAAAGCGUU
CGUUAUCCGGCCAACUACUUCGAAAAACCUAGUAACACCGUGGAAGUUGCAGAGUGU
UUCGCUCAGCACUACCCCAGUGUAGAUCAGGUCGAUGAGUCACCGCAUUCCCCACGG
GCGACCGUGGCGGUGGCUGCGUUGGCGGCCUGCCCAUGGGGAAACCCAUGGGACGCU
CUAAUACAGACAUGGUGCGAAGAGUCUAUUGAGCUAGUUGGUAGUCCUCCGGCCCCU
GAAUGCGGCUAAUCCUAACUGCGGAGCACACACCCUCAAGCCAGAGGGCAGUGUGUC
GUAACGGGCAACUCUGCAGCGGAACCGACUACUUUGGGUGUCCGUGUUUCAUUUUAU
UCCUAUACUGGCUGCUUAUGGUGACAAUUGAGAGAUCGUUACCAUAUAGCUAUUGGA
UUGGCCAUCCGGUGACUAAUAGAGCUAUUAUAUCCCUUUGUUGGGUUUAUACCACU
UAGCUUGAAAGAGGUUAAAACAUUACAAUUCAUUGUUAAGUUGAAUACAGCAAAauggg
auccgugagcaagggcgaggagcuguucaccgggguggugcccauccuggucgagcuggacggcgacguaaa
cggccacaaguucagcguguccggcgagggcgagggcgaugccaccuacggcaagcugacccugaaguucauc
ugcaccaccggcaagcugcccgugcccuggcccacccucgugaccacccugaccuacggcgugcagugcuuca
gccgcuaccccgaccacaugaagcagcacgacuucuucaaguccgccaugcccgaaggcuacguccaggagcg
caccaucuucuucaaggacgacggcaacuacaagacccgcgccgaggugaaguucgagggcgacacccuggug
aaccgcaucgagcugaagggcaucgacuucaaggaggacggcaacauccuggggcacaagcuggaguacaacu
acaacagccacaacgucuauaucauggccgacaagcagaagaacggcaucaaggugaacuucaagauccgcca
caacaucgaggacggcagcgugcagcucgccgaccacuaccagcagaacacccccaucggcgacggccccgug
cugcugcccgacaaccacuaccugagcacccaguccgcccugagcaaagaccccaacgagaagcgcgaucacau**

gguccugcuggaguucgugaccgccgccgggaucacucucggcauggacgagcuguacaagagaucucauau
gcaucucgagugauagcuaccugcacuguaagcacuuugucuagaccuucugcggggcuugccuucuggcca
ugcccuucuucucucccuugcaccuguaccucuuggucuuuGAAUAAAGCCUGAGUAGGAAAAAAA
AAAAAAAAAAAAAAAAAAAAAAAAAAAAAAAAAAAAAAAAAAAAAAAAAAAAAAAAAA
AAAAAAAAAAAAAAAAAAAAAAAAAAAAAAAAAAAAAAAAAAAAAAAAAAAAAAAGGC
UAUUAUGCGUUACCGGCGAGACGCUACGGACUUAAAUAAUUGAGCCUUAAAGAAGAAA
UUCUUUAAGUGGAUGCUCUCAAACUCAGGGAAACCUAAAUCUAGUUAUAGACAAGGCAA
UCCUGAGCCAAGCCGAAGUAGUAAUUAGUAAGACCAGUGGACAAUCGACGGAUAACAGC
AUAUCUAGACACAGGAAACAGCUAUGACCAUGAUUACGCCAAGCUUGCAUGCCUGCAGG
UCGACUCUAGAGGAUCCCCGGGUACCGAGCUCGAAUU

5’T206 Linear EGFP

Group I intron, CVB3 IRES, EGFP, miR-206 target site

GGGAAAAUCCGUUGACCUUAAACGGUCGUGUGGGUUCAAGUCCCUCCACCCCCACGCC
GGAAACGCAAUAGCCGGCGAAUUAAGAGAGAAAAGAAGAGUAAGAAGAAAUAUAAGACAC
CGGUCCCACACACUUCCUUACAUUCCAGCCACCUUAAAACAGCCUGUGGGUUGAUCCCA
CCCACAGGCCCAUUGGGCGCUAGCACUCUGGUAUCACGGUACCUUUGUGCGCCUGUUU
UAUACCCCCUCCCCCAACUGUAACUUAGAAGUAACACACACCGAUCAACAGUCAGCGUG
GCACACCAGCCACGUUUUGAUCAAGCACUUCUGUUACCCCGGACUGAGUAUCAAUAGAC
UGCUCACGCGGUUGAAGGAGAAAGCGUUCGUUAUCCGGCCAACUACUUCGAAAAACCUA
GUAACACCGUGGAAGUUGCAGAGUGUUUCGCUCAGCACUACCCCAGUGUAGAUCAGGU
CGAUGAGUCACCGCAUUCCCCACGGGCGACCGUGGCGGUGGCUGCGUUGGCGGCCUG
CCCAUGGGGAAACCCAUGGGACGCUCUAAUACAGACAUGGUGCGAAGAGUCUAUUGAG
CUAGUUGGUAGUCCUCCGGCCCCUGAAUGCGGCUAAUCCUAACUGCGGAGCACACACC
CUCAAGCCAGAGGGCAGUGUGUCGUAACGGGCAACUCUGCAGCGGAACCGACUACUUU
GGGUGUCCGUGUUUCAUUUUAUUCCUAUACUGGCUGCUUAUGGUGACAAUUGAGAGAU
CGUUACCAUAUAGCUAUUGGAUUGGCCAUCCGGUGACUAAUAGAGCUAUUAUAUCCC
UUUGUUGGGUUUAUACCACUUAGCUUGAAAGAGGUUAAAACAUUACAAUUCAUUGUUAA
GUUGAAUACAGCAAAaugggauccgugagcaagggcgaggagcuguucaccgggguggugcccauccuggucg
agcuggacggcgacguaaacggccacaaguucagcguguccggcgagggcgagggcgaugccaccuacggcaagcuga
cccugaaguucaucugcaccaccggcaagcugcccgugcccuggcccacccucgugaccacccugaccuacggcgugca
gugcuucagccgcuaccccgaccacaugaagcagcacgacuucuucaaguccgccaugcccgaaggcuacguccaggag
cgcaccaucuucuucaaggacgacggcaacuacaagacccgcgccgaggugaaguucgagggcgacacccuggugaacc
gcaucgagcugaagggcaucgacuucaaggaggacggcaacauccuggggcacaagcuggaguacaacuacaacagcc
acaacgucuauaucauggccgacaagcagaagaacggcaucaaggugaacuucaagauccgccacaacaucgaggacg
gcagcgugcagcucgccgaccacuaccagcagaacacccccaucggcgacggccccgugcugcugcccgacaaccacuac

cugagcacccaguccgcccugagcaaagaccccaacgagaagcgcgaucacaugguccugcuggaguucgugaccgccg
ccgggaucacucucggcauggacgagcuguacaagagaucucauaugcaucucgagugauagucuagaccuucugcgg
ggcuugccuucuggccaugcccuucuucucucccuugcaccuguaccucuuggucuuuGAAUAAAGCCUGAGUA
GGGGCUAUUAUGCGUUACCGGCGAGACGCUACGGACUUAAAAAAAAAAAAAAAAAAAAA
AAAAAAAAAAAAAAAAAAAAAAAAAAAAAAAAAAAAAAAAAAAAAAAAAAAAAAAAAAAA
AAAAAAAAAAAAAAAAAAAAAAAAAAAAAAAAAAAAA

3'T206 Linear EGFP

Group I intron, CVB3 IRES, EGFP, miR-206 target site

GGGAAAAUCCGUUGACCUUAAACGGUCGUGUGGGUUCAAGUCCCUCCACCCCCACGCC
GGAAACGCAAUAGCCGGCGAAUUAAGAGAGAAAAGAAGAGUAAGAAGAAAUAUAAGACAC
CGGUCGCCACCUUAAAACAGCCUGUGGGUUGAUCCCACCCACAGGCCCAUUGGGCGCU
AGCACUCUGGUAUCACGGUACCUUUGUGCGCCUGUUUUAUACCCCCUCCCCCAACUGU
AACUUAGAAGUAACACACACCGAUCAACAGUCAGCGUGGCACACCAGCCACGUUUUGAU
CAAGCACUUCUGUUACCCCGGACUGAGUAUCAAUAGACUGCUCACGCGGUUGAAGGAG
AAAGCGUUCGUUAUCCGGCCAACUACUUCGAAAAACCUAGUAACACCGUGGAAGUUGCA
GAGUGUUUCGCUCAGCACUACCCCAGUGUAGAUCAGGUCGAUGAGUCACCGCAUUCCC
CACGGGCGACCGUGGCGGUGGCUGCGUUGGCGGCCUGCCCAUGGGGAAACCCAUGGG
ACGCUCUAAUACAGACAUGGUGCGAAGAGUCUAUUGAGCUAGUUGGUAGUCCUCCGGC
CCCUGAAUGCGGCUAAUCCUAACUGCGGAGCACACACCCUCAAGCCAGAGGGCAGUGU
GUCGUAACGGGCAACUCUGCAGCGGAACCGACUACUUUGGGUGUCCGUGUUUCAUUUU
AUUCCUAUACUGGCUGCUUAUGGUGACAAUUGAGAGAUCGUUACCAUAUAGCUAUUGGA
UUGGCCAUCCGGUGACUAAUAGAGCUAUUAUAUCCCUUUGUUGGGUUUAUACCACUU
AGCUUGAAAGAGGUUAAAACAUUACAAUUCAUUGUUAAGUUGAAUACAGCAAAaugggaucc
gugagcaagggcgaggagcuguucaccgggguggugcccauccuggucgagcuggacggcgacguaaacggccacaag
uucagcguguccggcgagggcgagggcgaugccaccuacggcaagcugacccugaaguucaucugcaccaccggcaagc
ugcccgugcccuggcccacccucgugaccacccugaccuacggcgugcagugcuucagccgcuaccccgaccacaugaa
gcagcacgacuucuucaaguccgccaugcccgaaggcuacguccaggagcgcaccaucuucuucaaggacgacggcaac
uacaagacccgcgccgaggugaaguucgagggcgacacccuggugaaccgcaucgagcugaagggcaucgacuucaag
gaggacggcaacauccuggggcacaagcuggaguacaacuacaacagccacaacgucuauaucauggccgacaagcag
aagaacggcaucaaggugaacuucaagauccgccacaacaucgaggacggcagcgugcagcucgccgaccacuaccagc
agaacacccccaucggcgacggccccgugcugcugcccgacaaccacuaccugagcacccaguccgcccugagcaaaga
ccccaacgagaagcgcgaucacaugguccugcuggaguucgugaccgccgccgggaucacucucggcauggacgagcug
uacaagagaucucauaugcaucucgagugauagCCACACACUUCCUUACAUUCCAucuagaccuucugcgg
ggcuugccuucuggccaugcccuucuucucucccuugcaccuguaccucuuggucuuuGAAUAAAGCCUGAGUA
GGGGCUAUUAUGCGUUACCGGCGAGACGCUACGGACUUAAAAAAAAAAAAAAAAAAAAA

AAAAAAAAAAAAAAAAAAAAAAAAAAAAAAAAAAAAAAAAAAAAAAAAAAAAAAAAAAAA
AAAAAAAAAAAAAAAAAAAAAAAAAAAAAAAAA

5’T302a-5p Linear EGFP

Group I intron, CVB3 IRES, EGFP, miR-302a-5p target site

GGGAAAAUCCGUUGACCUUAAACGGUCGUGUGGGUUCAAGUCCCUCCACCCCCACGCC
GGAAACGCAAUAGCCGGCGAAUUAAGAGAGAAAAGAAGAGUAAGAAGAAAUAUAAGACAC
CGGUCAGCAAGUACAUCCACGUUUAAGUGCCACCUUAAAACAGCCUGUGGGUUGAUCCC
ACCCACAGGCCCAUUGGGCGCUAGCACUCUGGUAUCACGGUACCUUUGUGCGCCUGUU
UUAUACCCCCUCCCCCAACUGUAACUUAGAAGUAACACACACCGAUCAACAGUCAGCGU
GGCACACCAGCCACGUUUUGAUCAAGCACUUCUGUUACCCCGGACUGAGUAUCAAUAGA
CUGCUCACGCGGUUGAAGGAGAAAGCGUUCGUUAUCCGGCCAACUACUUCGAAAAACCU
AGUAACACCGUGGAAGUUGCAGAGUGUUUCGCUCAGCACUACCCCAGUGUAGAUCAGG
UCGAUGAGUCACCGCAUUCCCCACGGGCGACCGUGGCGGUGGCUGCGUUGGCGGCCU
GCCCAUGGGGAAACCCAUGGGACGCUCUAAUACAGACAUGGUGCGAAGAGUCUAUUGA
GCUAGUUGGUAGUCCUCCGGCCCCUGAAUGCGGCUAAUCCUAACUGCGGAGCACACAC
CCUCAAGCCAGAGGGCAGUGUGUCGUAACGGGCAACUCUGCAGCGGAACCGACUACUU
UGGGUGUCCGUGUUUCAUUUUAUUCCUAUACUGGCUGCUUAUGGUGACAAUUGAGAGA
UCGUUACCAUAUAGCUAUUGGAUUGGCCAUCCGGUGACUAAUAGAGCUAUUAUAUCC
CUUUGUUGGGUUUAUACCACUUAGCUUGAAAGAGGUUAAAACAUUACAAUUCAUUGUUA
AGUUGAAUACAGCAAAaugggauccgugagcaagggcgaggagcuguucaccgggguggugcccauccugguc
gagcuggacggcgacguaaacggccacaaguucagcguguccggcgagggcgagggcgaugccaccuacggcaagcug
acccugaaguucaucugcaccaccggcaagcugcccgugcccuggcccacccucgugaccacccugaccuacggcgugc
agugcuucagccgcuaccccgaccacaugaagcagcacgacuucuucaaguccgccaugcccgaaggcuacguccagga
gcgcaccaucuucuucaaggacgacggcaacuacaagacccgcgccgaggugaaguucgagggcgacacccuggugaa
ccgcaucgagcugaagggcaucgacuucaaggaggacggcaacauccuggggcacaagcuggaguacaacuacaacag
ccacaacgucuauaucauggccgacaagcagaagaacggcaucaaggugaacuucaagauccgccacaacaucgagga
cggcagcgugcagcucgccgaccacuaccagcagaacacccccaucggcgacggccccgugcugcugcccgacaaccacu
accugagcacccaguccgcccugagcaaagaccccaacgagaagcgcgaucacauggucCugcuggaguucgugaccgc
cgccgggaucacucucggcauggacgagcuguacaagagaucucauaugcaucucgagugauagucuagaccuucugc
ggggcuugccuucuggccaugcccuucuucucucccuugcaccuguaccucuuggucuuuGAAUAAAGCCUGAG
UAGGGGCUAUUAUGCGUUACCGGCGAGACGCUACGGACUUAAAAAAAAAAAAAAAAAAA
AAAAAAAAAAAAAAAAAAAAAAAAAAAAAAAAAAAAAAAAAAAAAAAAAAAAAAAAAAAA
AAAAAAAAAAAAAAAAAAAAAAAAAAAAAAAAAAA

3’T302a-5p Linear EGFP

Group I intron, CVB3 IRES, EGFP, miR-302a-5p target site

GGGAAAAUCCGUUGACCUUAAACGGUCGUGUGGGUUCAAGUCCCUCCACCCCCACGCC
GGAAACGCAAUAGCCGGCGAAUUAAGAGAGAAAAGAAGAGUAAGAAGAAAUAUAAGACAC
CGGUCGCCACCUUAAAACAGCCUGUGGGUUGAUCCCACCCACAGGCCCAUUGGGCGCU
AGCACUCUGGUAUCACGGUACCUUUGUGCGCCUGUUUUAUACCCCCUCCCCCAACUGU
AACUUAGAAGUAACACACACCGAUCAACAGUCAGCGUGGCACACCAGCCACGUUUUGAU
CAAGCACUUCUGUUACCCCGGACUGAGUAUCAAUAGACUGCUCACGCGGUUGAAGGAG
AAAGCGUUCGUUAUCCGGCCAACUACUUCGAAAAACCUAGUAACACCGUGGAAGUUGCA
GAGUGUUUCGCUCAGCACUACCCCAGUGUAGAUCAGGUCGAUGAGUCACCGCAUUCCC
CACGGGCGACCGUGGCGGUGGCUGCGUUGGCGGCCUGCCCAUGGGGAAACCCAUGGG
ACGCUCUAAUACAGACAUGGUGCGAAGAGUCUAUUGAGCUAGUUGGUAGUCCUCCGGC
CCCUGAAUGCGGCUAAUCCUAACUGCGGAGCACACACCCUCAAGCCAGAGGGCAGUGU
GUCGUAACGGGCAACUCUGCAGCGGAACCGACUACUUUGGGUGUCCGUGUUUCAUUUU
AUUCCUAUACUGGCUGCUUAUGGUGACAAUUGAGAGAUCGUUACCAUAUAGCUAUUGGA
UUGGCCAUCCGGUGACUAAUAGAGCUAUUAUAUAUCCCUUUGUUGGGUUUAUACCACUU
AGCUUGAAAGAGGUUAAAACAUUACAAUUCAUUGUUAAGUUGAAUACAGCAAAaugggaucc
gugagcaagggcgaggagcuguucaccgggguggugcccauccuggucgagcuggacggcgacguaaacggccacaag
uucagcguguccggcgagggcgagggcgaugccaccuacggcaagcugacccugaaguucaucugcaccaccggcaagc
ugcccgugcccuggcccacccucgugaccacccugaccuacggcgugcagugcuucagccgcuaccccgaccacaugaa
gcagcacgacuucuucaaguccgccaugcccgaaggcuacguccaggagcgcaccaucuucuucaaggacgacggcaac
uacaagacccgcgccgaggugaaguucgagggcgacacccuggugaaccgcaucgagcugaagggcaucgacuucaag
gaggacggcaacauccuggggcacaagcuggaguacaacuacaacagccacaacgucuauaucauggccgacaagcag
aagaacggcaucaaggugaacuucaagauccgccacaacaucgaggacggcagcgugcagcucgccgaccacuaccagc
agaacacccccaucggcgacggccccgugcugcugcccgacaaccacuaccugagcacccaguccgcccugagcaaaga
ccccaacgagaagcgcgaucacauggucccugcuggaguucgugaccgccgccgggaucacucucggcauggacgagcug
uacaagagaucucauaugcaucucgagugauagAGCAAGUACAUCCACGUUUAAGUucuagaccuucugcg
gggcuugccuucuggccaugcccuucuucucucccuugcaccuguaccucuuggucuuuGAAUAAAGCCUGAGU
AGGGGCUAUUAUGCGUUACCGGCGAGACGCUACGGACUUAAAAAAAAAAAAAAAAAAAA
AAAAAAAAAAAAAAAAAAAAAAAAAAAAAAAAAAAAAAAAAAAAAAAAAAAAAAAAAAAAA
AAAAAAAAAAAAAAAAAAAAAAAAAAAAAAAAAAAA

5’T21-5p Linear EGFP

Group I intron, CVB3 IRES, EGFP, miR-21-5p target site

GGGAAAAUCCGUUGACCUUAAACGGUCGUGUGGGUUCAAGUCCCUCCACCCCCACGCC
GGAAACGCAAUAGCCGGCGAAUUAAGAGAGAAAAGAAGAGUAAGAAGAAAUAUAAGACAC
CGGUCUCAACAUCAGUCUGAUAAGCUAGCCACCUUAAAACAGCCUGUGGGUUGAUCCCA

CCCACAGGCCCAUUGGGCGCUAGCACUCUGGUAUCACGGUACCUUUGUGCGCCUGUUU
UAUACCCCCUCCCCCAACUGUAACUUAGAAGUAACACACACCGAUCAACAGUCAGCGUG
GCACACCAGCCACGUUUUGAUCAAGCACUUCUGUUACCCCGGACUGAGUAUCAAUAGAC
UGCUCACGCGGUUGAAGGAGAAAGCGUUCGUUAUCCGGCCAACUACUUCGAAAAACCUA
GUAACACCGUGGAAGUUGCAGAGUGUUUCGCUCAGCACUACCCCAGUGUAGAUCAGGU
CGAUGAGUCACCGCAUUCCCCACGGGCGACCGUGGCGGUGGCUGCGUUGGCGGCCUG
CCCAUGGGGAAACCCAUGGGACGCUCUAAUACAGACAUGGUGCGAAGAGUCUAUUGAG
CUAGUUGGUAGUCCUCCGGCCCCUGAAUGCGGCUAAUCCUAACUGCGGAGCACACACC
CUCAAGCCAGAGGGCAGUGUGUCGUAACGGGCAACUCUGCAGCGGAACCGACUACUUU
GGGUGUCCGUGUUUCAUUUUAUUCCUAUACUGGCUGCUUAUGGUGACAAUUGAGAGAU
CGUUACCAUAUAGCUAUUGGAUUGGCCAUCCGGUGACUAAUAGAGCUAUUAUAUCCC
UUUGUUGGGUUUAUACCACUUAGCUUGAAAGAGGUUAAAACAUUACAAUUCAUUGUUAA
GUUGAAUACAGCAAAaugggauccgugagcaagggcgaggagcuguucaccgggguggugcccauccuggucg
agcuggacggcgacguaaacggccacaaguucagcguguccggcgagggcgagggcgaugccaccuacggcaagcuga
cccugaaguucaucugcaccaccggcaagcugcccgugcccuggcccacccucgugaccacccugaccuacggcgugca
gugcuucagccgcuaccccgaccacaugaagcagcacgacuucuucaaguccgccaugcccgaaggcuacguccaggag
cgcaccaucuucuucaaggacgacggcaacuacaagacccgcgccgaggugaaguucgagggcgacacccuggugaacc
gcaucgagcugaagggcaucgacuucaaggaggacggcaacauccuggggcacaagcuggaguacaacuacaacagcc
acaacgucuauaucauggccgacaagcagaagaacggcaucaaggugaacuucaagauccgccacaacaucgaggacg
gcagcgugcagcucgccgaccacuaccagcagaacacccccaucggcgacggccccgugcugcugcccgacaaccacuac
cugagcacccaguccgcccugagcaaagaccccaacgagaagcgcgaucacauggccugcuggaguucgugaccgccg
ccgggaucacucucggcauggacgagcuguacaagagaucucauaugcaucucgagugauagucuagaccuucugcgg
ggcuugccuucuggccaugcccuucuucucucccuugcaccuguaccucuuggucuuuGAAUAAAGCCUGAGUA
GGGGCUAUUAUGCGUUACCGGCGAGACGCUACGGACUUAAAAAAAAAAAAAAAAAAAAAA
AAAAAAAAAAAAAAAAAAAAAAAAAAAAAAAAAAAAAAAAAAAAAAAAAAAAAAAAAAAAAAA
AAAAAAAAAAAAAAAAAAAAAAAAAAAAAAAAAAA

3'T21-5p Linear EGFP

Group I intron, CVB3 IRES, EGFP, miR-21-5p target site

GGGAAAAUCCGUUGACCUUAAACGGUCGUGUGGGUUCAAGUCCCUCCACCCCCACGCC
GGAAACGCAAUAGCCGGCGAAUUAAGAGAGAAAAGAAGAGUAAGAAGAAAUAUAAGACAC
CGGUCGCCACCUUAAAACAGCCUGUGGGUUGAUCCCACCCACAGGCCCAUUGGGCGCU
AGCACUCUGGUAUCACGGUACCUUUGUGCGCCUGUUUUAUACCCCCUCCCCCAACUGU
AACUUAGAAGUAACACACACCGAUCAACAGUCAGCGUGGCACACCAGCCACGUUUUGAU
CAAGCACUUCUGUUACCCCGGACUGAGUAUCAAUAGACUGCUCACGCGGUUGAAGGAG
AAAGCGUUCGUUAUCCGGCCAACUACUUCGAAAAACCUAGUAACACCGUGGAAGUUGCA

GAGUGUUUCGCUCAGCACUACCCCAGUGUAGAUCAGGUCGAUGAGUCACCGCAUUCCC
CACGGGCGACCGUGGCGGUGGCUGCGUUGGCGGCCUGCCCAUGGGGAAACCCAUGGG
ACGCUCUAAUACAGACAUGGUGCGAAGAGUCUAUUGAGCUAGUUGGUAGUCCUCCGGC
CCCUGAAUGCGGCUAAUCCUAACUGCGGAGCACACACCCUCAAGCCAGAGGGCAGUGU
GUCGUAACGGGCAACUCUGCAGCGGAACCGACUACUUUGGGUGUCCGUGUUUCAUUUU
AUUCCUAUACUGGCUGCUUAUGGUGACAAUUGAGAGAUCGUUACCAUAUAGCUAUUGGA
UUGGCCAUCCGGUGACUAAUAGAGCUAUUAUAUCCCUUUGUUGGGUUUAUACCACUU
AGCUUGAAAGAGGUUAAAACAUUACAAUUCAUUGUUAAGUUGAAUACAGCAAAaugggaucc
gugagcaagggcgaggagcuguucaccgggguggugcccauccuggucgagcuggacggcgacguaaacggccacaag
uucagcguguccggcgagggcgagggcgaugccaccuacggcaagcugacccugaaguucaucugcaccaccggcaagc
ugcccgugcccuggcccacccucgugaccacccugaccuacggcgugcagugcuucagccgcuaccccgaccacaugaa
gcagcacgacuucuucaaguccgccaugcccgaaggcuacguccaggagcgcaccaucuucuucaaggacgacggcaac
uacaagacccgcgccgaggugaaguucgagggcgacacccuggugaaccgcaucgagcugaagggcaucgacuucaag
gaggacggcaacauccuggggcacaagcuggaguacaacuacaacagccacaacgucuauaucauggccgacaagcag
aagaacggcaucaaggugaacuucaagauccgccacaacaucgaggacggcagcgugcagcucgccgaccacuaccagc
agaacacccccaucggcgacggccccgugcugcugcccgacaaccacuaccugagcacccaguccgcccugagcaaaga
ccccaacgagaagcgcgaucacauggccugcuggaguucgugaccgccgccgggaucacucucggcauggacgagcug
uacaagagaucucauaugcaucucgagugauagUCAACAUCAGUCUGAUAAGCUAucuagaccuucugcgg
ggcuugccuucuggccaugcccuucuucucucccuugcaccuguaccucuuggucuuuGAAUAAAGCCUGAGUA
GGGGCUAUUAUGCGUUACCGGCGAGACGCUACGGACUUAAAAAAAAAAAAAAAAAAAAA
AAAAAAAAAAAAAAAAAAAAAAAAAAAAAAAAAAAAAAAAAAAAAAAAAAAAAAAAAAAAAAAA
AAAAAAAAAAAAAAAAAAAAAAAAAAAAAAAAAAA

5’T339-5p Linear EGFP

Group I intron, CVB3 IRES, EGFP, miR-339-5p target site

GGGAAAAUCCGUUGACCUUAAACGGUCGUGUGGGUUCAAGUCCCUCCACCCCCACGCC
GGAAACGCAAUAGCCGGCGAAUUAAGAGAGAAAAGAAGAGUAAGAAGAAAUAUAAGACAC
CGGUCcgugagcuccuggaggacagggaGCCACCUUAAAACAGCCUGUGGGUUGAUCCCACCCA
CAGGCCCAUUGGGCGCUAGCACUCUGGUAUCACGGUACCUUUGUGCGCCUGUUUUAUA
CCCCCUCCCCCAACUGUAACUUAGAAGUAACACACACCGAUCAACAGUCAGCGUGGCAC
ACCAGCCACGUUUUGAUCAAGCACUUCUGUUACCCCGGACUGAGUAUCAAUAGACUGCU
CACGCGGUUGAAGGAGAAAGCGUUCGUUAUCCGGCCAACUACUUCGAAAAACCUAGUAA
CACCGUGGAAGUUGCAGAGUGUUUCGCUCAGCACUACCCCAGUGUAGAUCAGGUCGAU
GAGUCACCGCAUUCCCCACGGGCGACCGUGGCGGUGGCUGCGUUGGCGGCCUGCCCA
UGGGGAAACCCAUGGGACGCUCUAAUACAGACAUGGUGCGAAGAGUCUAUUGAGCUAG
UUGGUAGUCCUCCGGCCCCUGAAUGCGGCUAAUCCUAACUGCGGAGCACACACCCUCA

AGCCAGAGGGCAGUGUGUCGUAACGGGCAACUCUGCAGCGGAACCGACUACUUUGGGU
GUCCGUGUUUCAUUUUAUUCCUAUACUGGCUGCUUAUGGUGACAAUUGAGAGAUCGUU
ACCAUAUAGCUAUUGGAUUGGCCAUCCGGUGACUAAUAGAGCUAUUAUAUCCCUUUG
UUGGGUUUAUACCACUUAGCUUGAAAGAGGUUAAAACAUUACAAUUCAUUGUUAAGUUG
AAUACAGCAAAaugggauccgugagcaagggcgaggagcuguucaccgggguggugcccauccuggucgagcugg
acggcgacguaaacggccacaaguucagcguguccggcgagggcgagggcgaugccaccuacggcaagcugacccuga
aguucaucugcaccaccggcaagcugcccgugcccuggcccacccucgugaccacccugaccuacggcgugcagugcuu
cagccgcuaccccgaccacaugaagcagcacgacuucuucaaguccgccaugcccgaaggcuacguccaggagcgcacc
aucuucuucaaggacgacggcaacuacaagacccgcgccgaggugaaguucgagggcgacacccuggugaaccgcauc
gagcugaagggcaucgacuucaaggaggacggcaacauccuggggcacaagcuggaguacaacuacaacagccacaac
gucuauaucauggccgacaagcagaagaacggcaucaaggugaacuucaagauccgccacaacaucgaggacggcagc
gugcagcucgccgaccacuaccagcagaacacccccaucggcgacggccccgugcugcugcccgacaaccacuaccuga
gcacccaguccgcccugagcaaagaccccaacgagaagcgcgaucacauggtccugcuggaguucgugaccgccgccgg
gaucacucucggcauggacgagcuguacaagagaucucauaugcaucucgagugauagucuagaccuucugcggggcu
ugccuucuggccaugcccuucuucucucccuugcaccuguaccucuuggucuuuGAAUAAAGCCUGAGUAGG
GGCUAUUAUGCGUUACCGGCGAGACGCUACGGACUUAAAAAAAAAAAAAAAAAAAAAAAA
AAAAAAAAAAAAAAAAAAAAAAAAAAAAAAAAAAAAAAAAAAAAAAAAAAAAAAAAAAAAAAA
AAAAAAAAAAAAAAAAAAAAAAAAAAAAAAAAA

3’T339-5p Linear EGFP

Group I intron, CVB3 IRES, EGFP, miR-339-5p target site

GGGAAAAUCCGUUGACCUUAAACGGUCGUGUGGGUUCAAGUCCCUCCACCCCCACGCC
GGAAACGCAAUAGCCGGCGAAUUAAGAGAGAAAAGAAGAGUAAGAAGAAAUAUAAGACAC
CGGUCGCCACCUUAAAACAGCCUGUGGGUUGAUCCCACCCACAGGCCCAUUGGGCGCU
AGCACUCUGGUAUCACGGUACCUUUGUGCGCCUGUUUUAUACCCCCUCCCCCAACUGU
AACUUAGAAGUAACACACACCGAUCAACAGUCAGCGUGGCACACCAGCCACGUUUUGAU
CAAGCACUUCUGUUACCCCGGACUGAGUAUCAAUAGACUGCUCACGCGGUUGAAGGAG
AAAGCGUUCGUUAUCCGGCCAACUACUUCGAAAAACCUAGUAACACCGUGGAAGUUGCA
GAGUGUUUCGCUCAGCACUACCCCAGUGUAGAUCAGGUCGAUGAGUCACCGCAUUCCC
CACGGGCGACCGUGGCGGUGGCUGCGUUGGCGGCCUGCCCAUGGGGAAACCCAUGGG
ACGCUCUAAUACAGACAUGGUGCGAAGAGUCUAUUGAGCUAGUUGGUAGUCCUCCGGC
CCCUGAAUGCGGCUAAUCCUAACUGCGGAGCACACACCCUCAAGCCAGAGGGCAGUGU
GUCGUAACGGGCAACUCUGCAGCGGAACCGACUACUUUGGGUGUCCGUGUUUCAUUUU
AUUCCUAUACUGGCUGCUUAUGGUGACAAUUGAGAGAUCGUUACCAUAUAGCUAUUGGA
UUGGCCAUCCGGUGACUAAUAGAGCUAUUAUAUCCCUUUGUUGGGUUUAUACCACUU
AGCUUGAAAGAGGUUAAAACAUUACAAUUCAUUGUUAAGUUGAAUACAGCAAAaugggaucc

gugagcaagggcgaggagcuguucaccgggguggugcccauccuggucgagcuggacggcgacguaaacggccacaag
uucagcguguccggcgagggcgagggcgaugccaccuacggcaagcugacccugaaguucaucugcaccaccggcaagc
ugcccgugcccuggcccacccucgugaccacccugaccuacggcgugcagugcuucagccgcuaccccgaccacaugaa
gcagcacgacuucuucaaguccgccaugcccgaaggcuacguccaggagcgcaccaucuucuucaaggacgacggcaac
uacaagacccgcgccgaggugaaguucgagggcgacacccuggugaaccgcaucgagcugaagggcaucgacuucaag
gaggacggcaacauccuggggcacaagcuggaguacaacuacaacagccacaacgucuauaucauggccgacaagcag
aagaacggcaucaaggugaacuucaagauccgccacaacaucgaggacggcagcgugcagcucgccgaccacuaccagc
agaacacccccaucggcgacggccccgugcugcugcccgacaaccacuaccugagcacccaguccgcccugagcaaaga
ccccaacgagaagcgcgaucacauggucccugcuggaguucgugaccgccgccgggaucacucucggcauggacgagcug
uacaagagaucucauaugcaucucgagugauagcgugagcuccuggaggacagggaucuagaccuucugcggggcuug
ccuucuggccaugcccuucuucucucccuugcaccuguaccucuuggucuuuGAAUAAAGCCUGAGUAGGGG
CUAUUAUGCGUUACCGGCGAGACGCUACGGACUUAAAAAAAAAAAAAAAAAAAAAAAAA
AAAAAAAAAAAAAAAAAAAAAAAAAAAAAAAAAAAAAAAAAAAAAAAAAAAAAAAAAAAAAAA
AAAAAAAAAAAAAAAAAAAAAAAAAAAAAA

linear switch (miR-206)

EGFP, miR-206 target site

GGGCGAAUUAAGAGAGAAAAGAAGAGUAAGAAGAAAUAUAAGACACCGGUcCCACACACU
UCCUUACAUUCCAgccaccaugggauccgugagcaagggcgaggagcuguucaccgggguggugcccauccugg
ucgagcuggacggcgacguaaacggccacaaguucagcguguccggcgagggcgagggcgaugccaccuacggcaagc
ugacccugaaguucaucugcaccaccggcaagcugcccgugcccuggcccacccucgugaccacccugaccuacggcgu
gcagugcuucagccgcuaccccgaccacaugaagcagcacgacuucuucaaguccgccaugcccgaaggcuacguccag
gagcgcaccaucuucuucaaggacgacggcaacuacaagacccgcgccgaggugaaguucgagggcgacacccuggug
aaccgcaucgagcugaagggcaucgacuucaaggaggacggcaacauccuggggcacaagcuggaguacaacuacaac
agccacaacgucuauaucauggccgacaagcagaagaacggcaucaaggugaacuucaagauccgccacaacaucgag
gacggcagcgugcagcucgccgaccacuaccagcagaacacccccaucggcgacggccccgugcugcugcccgacaacc
acuaccugagcacccaguccgcccugagcaaagaccccaacgagaagcgcgaucacaugguccugcuggaguucgugac
cgccgccgggaucacucucggcauggacgagcuguacaagagaucucauaugcaucucgagugauagucuagaccuuc
ugcggggcuugccuucuggccaugcccuucuucucucccuugcaccuguaccucuuggucuuuGAAUAAAGCCUG
AGUAGGAAAAAAAAAAAAAAAAAAAAAAAAAAAAAAAAAAAAAAAAAAAAAAAAAAAAAAAA
AAAAAAAAAAAAAAAAAAAAAAAAAAAAAAAAAAAAAAAAAAAAAAAAAAAAAAAAAAAAAAAA

linear switch (miR-302a-5p)

EGFP, miR-302a-5p target site

GGGCGAAUUAAGAGAGAAAAGAAGAGUAAGAAGAAAUAUAAGACACCGGUcAGCAAGUAC
AUCCACGUUUAAGUgccaccaugggauccgugagcaagggcgaggagcuguucaccgggguggugcccauccug

gucgagcuggacggcgacguaaacggccacaaguucagcguguccggcgagggcgagggcgaugccaccuacggcaag
cugacccugaaguucaucugcaccaccggcaagcugcccgugcccuggcccacccucgugaccacccugaccuacggcg
ugcagugcuucagccgcuaccccgaccacaugaagcagcacgacuucuucaaguccgccaugcccgaaggcuacgucca
ggagcgcaccaucuucuucaaggacgacggcaacuacaagacccgcgccgaggugaaguucgagggcgacacccuggu
gaaccgcaucgagcugaagggcaucgacuucaaggaggacggcaacauccuggggcacaagcuggaguacaacuacaa
cagccacaacgucuauaucauggccgacaagcagaagaacggcaucaaggugaacuucaagauccgccacaacaucga
ggacggcagcgugcagcucgccgaccacuaccagcagaacacccccaucggcgacggccccgugcugcugcccgacaac
cacuaccugagcacccaguccgcccugagcaaagaccccaacgagaagcgcgaucacauggucugcuggaguucguga
ccgccgccgggaucacucucggcauggacgagcuguacaagagaucucauaugcaucucgagugauagucuagaccuu
cugcggggcuugccuucuggccaugcccuucuucucucccuugcaccuguaccucuuggucuuuGAAUAAAGCCU
GAGUAGGAAAAAAAAAAAAAAAAAAAAAAAAAAAAAAAAAAAAAAAAAAAAAAAAAAAAAAAAAA
AAAAAAAAAAAAAAAAAAAAAAAAAAAAAAAAAAAAAAAAAAAAAAAAAAAAAAAAAAAAAAAAAA
A

linear switch (miR-21-5p)

EGFP, miR-21-5p target site

GGGCGAAUUAAGAGAGAAAAGAAGAGUAAGAAGAAAUAUAAGACACCGGUcUCAACAUCA
GUCUGAUAAGCUAgccaccaugggauccgugagcaagggcgaggagcuguucaccgggguggugcccauccugg
ucgagcuggacggcgacguaaacggccacaaguucagcguguccggcgagggcgagggcgaugccaccuacggcaagc
ugacccugaaguucaucugcaccaccggcaagcugcccgugcccuggcccacccucgugaccacccugaccuacggcgu
gcagugcuucagccgcuaccccgaccacaugaagcagcacgacuucuucaaguccgccaugcccgaaggcuacguccag
gagcgcaccaucuucuucaaggacgacggcaacuacaagacccgcgccgaggugaaguucgagggcgacacccuggug
aaccgcaucgagcugaagggcaucgacuucaaggaggacggcaacauccuggggcacaagcuggaguacaacuacaac
agccacaacgucuauaucauggccgacaagcagaagaacggcaucaaggugaacuucaagauccgccacaacaucgag
gacggcagcgugcagcucgccgaccacuaccagcagaacacccccaucggcgacggccccgugcugcugcccgacaacc
acuaccugagcacccaguccgcccugagcaaagaccccaacgagaagcgcgaucacauggucugcuggaguucgugac
cgccgccgggaucacucucggcauggacgagcuguacaagagaucucauaugcaucucgagugauagucuagaccuuc
ugcggggcuugccuucuggccaugcccuucuucucucccuugcaccuguaccucuuggucuuuGAAUAAAGCCUG
AGUAGGAAAAAAAAAAAAAAAAAAAAAAAAAAAAAAAAAAAAAAAAAAAAAAAAAAAAAAAAAA
AAAAAAAAAAAAAAAAAAAAAAAAAAAAAAAAAAAAAAAAAAAAAAAAAAAAAAAAAAAAAAAAAA

linear switch (miR-339-5p)

EGFP, miR-339-5p target site

GGGCGAAUUAAGAGAGAAAAGAAGAGUAAGAAGAAAUAUAAGACACCGGUccgugagcuccug
gaggacagggagccaccaugggauccgugagcaagggcgaggagcuguucaccgggguggugcccauccuggucgagc
uggacggcgacguaaacggccacaaguucagcguguccggcgagggcgagggcgaugccaccuacggcaagcugaccc

ugaaguucaucugcaccaccggcaagcugcccgugcccuggcccacccucgugaccacccugaccuacggcgugcagug
cuucagccgcuaccccgaccacaugaagcagcacgacuucuucaaguccgccaugcccgaaggcuacguccaggagcgc
accaucuucuucaaggacgacggcaacuacaagacccgcgccgaggugaaguucgagggcgacacccuggugaaccgc
aucgagcugaagggcaucgacuucaaggaggacggcaacauccuggggcacaagcuggaguacaacuacaacagccac
aacgucuauaucauggccgacaagcagaagaacggcaucaaggugaacuucaagauccgccacaacaucgaggacggc
agcgugcagcucgccgaccacuaccagcagaacacccccaucggcgacggccccgugcugcugcccgacaaccacuaccu
gagcacccaguccgcccugagcaaagaccccaacgagaagcgcgaucacauggccugcuggaguucgugaccgccgcc
gggaucacucucggcauggacgagcuguacaagagaucucauaugcaucucgagugauagucuagaccuucugcgggg
cuugccuucuggccaugcccuucuucucucccuugcaccuguaccucuuggucuuuGAAUAAAGCCUGAGUAG
GAAAAAAAAAAAAAAAAAAAAAAAAAAAAAAAAAAAAAAAAAAAAAAAAAAAAAAAAAA
AAAAAAAAAAAAAAAAAAAAAAAAAAAAAAAAAAAAAAAAAAAAAAAAAAAAAA

MS2CP responsive Circular EGFP variant1 (**bold: circularized**)

Group I intron, CVB3 IRES, EGFP, MS2CP binding motif

GGGAGACCCUCGACCGUCGAUUGUCCACUGGUCAACAAUAGAUGACUUACAACUAAUCG
GAAGGUGCAGAGACUCGACGGGAGCUACCCUAACGUCAAGACGAGGGUAAAGAGAG
UCCAAUUCUCAAAGCCAAUAGGCAGUAGCGAAAGCUGCAAGAGAAUG**AAAAUCCGUUGA**
CCUUAAACGGUCGUGUGGGUUCAAGUCCCUCCACCCCCACGCCGGAAACGCAAUAGC
CGGCGAAUUAAGAGAGAAAAGAAGAGUAAGAAGAAAUAUAAGACACCGGUCGCCACC
UUAAAACAGCCUGUGGGUUGAUCCCACCCACAGGCCCAUUGGGCGCUAGCACUCUGG
UAUCACGGUACCUUUGUGCGCCUGUUUUAUACCCCCUCCCCCAACUGUAACUUAGAAG
UAACACACACCGAUCAACAGUCAGCGUGGCACACCAGCCACGUUUUGAUCAAGCACUU
CUGUUACCCGGGAGCAGGUGAGGAUCACCCAUCUGCCACGAGCGAGGUGAGGAUCAC
CCAUCUCGCUCGUGUUCCCUUAUCCGGCCAACUACUUCGAAAAACCUAGUAACACCGU
GGAAGUUGCAGAGUGUUUCGCUCAGCACUACCCCAGUGUAGAUCAGGUCGAUGAGUC
ACCGCAUUCCCCACGGGCGACCGUGGCGGUGGCUGCGUUGGCGGCCUGCCCAUGGGG
AAACCCAUGGGACGCUCUAAUACAGACAUGGUGCGAAGAGUCUAUUGAGCUAGUUGG
UAGUCCUCCGGCCCCUGAAUGCGGCUAAUCCUAACUGCGGAGCACACACCCUCAAGC
CAGAGGGCAGUGUGUCGUAACGGGCAACUCUGCAGCGGAACCGACUACUUUGGGUGU
CCGUGUUUCAUUUUAUUCCUAUACUGGCUGCUUAUGGUGACAAUUGAGAGAUCGUUA
CCAUAUAGCUAUUGGAUUGGCCAUCCGGUGACUAAUAGAGCUAUUAUAUCCCUUUG
UUGGGUUUAUACCACUUAGCUUGAAAGAGGUUAAAACAUUACAAUUCAUUGUUAAGUU
GAAUACAGCAAAaugggauccgugagcaagggcgaggagcuguucaccgggguggugcccauccugguc
gagcuggacggcgacguaaacggccacaaguucagcguguccggcgagggcgagggcgaugccaccuacggc
aagcugacccugaaguucaucugcaccaccggcaagcugcccgugcccuggcccacccucgugaccacccuga
ccuacggcgugcagugcuucagccgcuaccccgaccacaugaagcagcacgacuucuucaaguccgccaugcc

cgaaggcuacguccaggagcgcaccaucuucuucaaggacgacggcaacuacaagacccgcgccgaggugaag
uucgagggcgacacccuggugaaccgcaucgagcugaagggcaucgacuucaaggaggacggcaacauccugg
ggcacaagcuggaguacaacuacaacagccacaacgucuauaucauggccgacaagcagaagaacggcaucaa
ggugaacuucaagauccgccacaacaucgaggacggcagcgugcagcucgccgaccacuaccagcagaacacc
cccaucggcgacggccccgugcugcugcccgacaaccacuaccugagcacccaguccgcccugagcaaagacc
ccaacgagaagcgcgaucacaugguccugcuggaguucgugaccgccgccgggaucacucucggcauggacga
gcuguacaagagaucucauaugcaucucgagugauagucuagaccuucugcggggcuugccuucuggccaug
cccuucuucucucccuugcaccuguaccucuuggucuuuGAAUAAAGCCUGAGUAGGGGCUAUUA
UGCGUUACCGGCGAGACGCUACGGACUUAAAUAAUUGAGCCUUAAAGAAGAAAUUCUU
UAAGUGGAUGCUCUCAAACUCAGGGAAACCUAAAUCUAGUUAUAGACAAGGCAAUCCUG
AGCCAAGCCGAAGUAGUAAUUAGUAAGACCAGUGGACAAUCGACGGAUAACAGCAUAUC
UAG

MS2CP responsive Circular EGFP variant2 (**bold: circularized**)

Group I intron, CVB3 IRES, EGFP, MS2CP binding motif

GGGAGACCCUCGACCGUCGAUUGUCCACUGGUCAACAAUAGAUGACUUACAACUAAUCG
GAAGGUGCAGAGACUCGACGGGAGCUACCCUAACGUCAAGACGAGGGUAAAGAGAG
UCCAAUUCUCAAAGCCAAUAGGCAGUAGCGAAAGCUGCAAGAGAAUG**AAAAUCCGUUGA**
CCUUAAACGGUCGUGUGGGUUCAAGUCCCUCCACCCCCACGCCGGAAACGCAAUAGC
CGGCGAAUUAAGAGAGAAAAGAAGAGUAAGAAGAAAUAUAAGACACCGGUCGCCACC
UUAAAACAGCCUGUGGGUGGUGAGGAUCACCCAUCCACCCACAGGCCCGGTGAGGAT
CACCCATCGGGCGCTAGCACTCTGGTAGGUGAGGAUCACCCAUCUACCUUUGUGCGCC
UGUUUUAUACCCCCUCCCCCAACUGUAACUUAGAAGUAACACACACCGAUCAACAGUC
AGCGUGGCACACCAGCCACGUUUUGAUCAAGCACUUCUGUUACCCCGGACUGAGUAU
CAAUAGACUGCUCACGCGGUUGAAGGAGAAAGCGUUCGUUAUCCGGCCAACUACUUC
GAAAAACCUAGUAACACCGUGGAAGUUGCAGAGUGUUUCGCUCAGCACUACCCCAGU
GUAGAUCAGGUCGAUGAGUCACCGCAUUCCCCACGGGCGACCGUGGCGGUGGCUGCG
UUGGCGGCCUGCCCAUGGGGAAACCCAUGGGACGCUCUAAUACAGACAUGGUGCGAA
GAGUCUAUUGAGCUAGUUGGUAGUCCUCCGGCCCCUGAAUGCGGCUAAUCCUAACUG
CGGAGCACACACCCUCAAGCCAGAGGGCAGUGUGUCGUAACGGGCAACUCUGCAGCG
GAACCGACUACUUUGGGUGUCCGUGUUUCAUUUUAUUCCUAUACUGGCUGCUUAUGG
UGACAAUUGAGAGAUCGUUACCAUAUAGCUAUUGGAUUGGCCAUCCGGUGACUAAUA
GAGCUAUUAUAUAUCCCUUUGUUGGGUUUAUACCACUUAGCUUGAAAGAGGUUAAAAC
AUUACAAUUCAUUGUUAAGUUGAAUACAGCAAAaugggauccgugagcaagggcgaggagcugu
ucaccgggguggugcccauccuggucgagcuggacggcgacguaaacggccacaaguucagcguguccggcg
agggcgagggcgaugccaccuacggcaagcugacccugaaguucaucugcaccaccggcaagcugcccgugcc

cuggcccacccucgugaccacccugaccuacggcgugcagugcuucagccgcuaccccgaccacaugaagcag cacgacuucuucaaguccgccaugcccgaaggcuacguccaggagcgcaccaucuucuucaaggacgacggca acuacaagacccgcgccgaggugaaguucgagggcgacacccuggugaaccgcaucgagcugaagggcaucga cuucaaggaggacggcaacauccuggggcacaagcuggaguacaacuacaacagccacaacgucuauaucaug gccgacaagcagaagaacggcaucaaggugaacuucaagauccgccacaacaucgaggacggcagcgugcagc ucgccgaccacuaccagcagaacacccccaucggcgacggccccgugcugcugcccgacaaccacuaccugagc acccaguccgcccugagcaaagaccccaacgagaagcgcgaucacauggucgugcuggaguucgugaccgccg ccgggaucacucucggcauggacgagcuguacaagagaucucauaugcaucucgagugauagucuagaccuu cugcggggcuugccuucuggccaugcccuucuucucucccuugcaccuguaccucuuggucuuuGAAUAA AGCCUGAGUAGGGGCUAUUAUGCGUUACCGGCGAGACGCUACGGACUUAAAUAAUUGA GCCUUAAAGAAGAAAUUCUUUAAGUGGAUGCUCUCAAACUCAGGGAAACCUAAAUCUAG UUAUAGACAAGGCAAUCCUGAGCCAAGCCGAAGUAGUAAUUAGUAAGACCAGUGGACAA UCGACGGAUAACAGCAUAUCUAG

MS2CP responsive Circular EGFP variant3 (**bold: circularized**)

Group I intron, CVB3 IRES, EGFP, MS2CP binding motif

GGGAGACCCUCGACCGUCGAUUGUCCACUGGUCAACAAUAGAUGACUUACAACUAAUCG GAAGGUGCAGAGACUCGACGGGAGCUACCCUAACGUCAAGACGAGGGUAAAGAGAGAG UCCAAUUCUCAAAGCCAAUAGGCAGUAGCGAAAGCUGCAAGAGAAUG**AAAAUCCGUUGA CCUUAAACGGUCGUGUGGGUUCAAGUCCCUCCACCCCCACGCCGGAAACGCAAUAGC CGGCGAAUUAAGAGAGAAAAGAAGAGUAAGAAGAAAUAUAAGACACCGGUCGCCACC UUAAAACAGCCUGUGGGUUGAUCCCACCCACAGGCCCAUUGGGCGCUAGCACUCUGG UAUCACGGUACCUUUGUGCGCCUGUUUUAUACCCCCUCCCCCAACUGUAACUUAGAAG UAACACACACCGAUCAACAGUCAGCGUGGCACACCAGCCACGUUUUGAUCAAGCACUU CUGUUACCCCGGACUGAGUAUCAAUAGACUGCUCACGCGGUUGAAGGAGAAAGCGUU CGUUAUCCGGCCAACUACUUCGAAAAACCUAGUAACACCGUGGAAGUUGCAGAGUGU UUCGCUCAGCACUACCCCAGUGUAGAUCAGGUCGAUGAGUCACCGCAUUCCCCACGG GCGACCGUGGCGGUGGCUGCGUUGGCGGCCUGCCCAUGGGGAAACCCAUGGGACGCU CUAAUACAGACAUGGUGCGAAGAGUCUAUUGAGCUAGUUGGUAGUCCUCCGGCCCCU GAAUGCGGCUAAUCCUAACUGCGGAGCACACACCCUCGCAGGUGAGGAUCACCCAUC UGCCACGAGCGAGGUGAGGAUCACCCAUCUCGCUCGUGUGAGGGCAGUGUGUCGUAA CGGGCAACUCUGCAGCGGAACCGACUACUUUGGGUGUCCGUGUUUCAUUUUAUUCCU AUACUGGCUGCUUAUGGUGACAAUUGAGAGAUCGUUACCAUAUAGCUAUUGGAUUGG CCAUCCGGUGACUAAUAGAGCUAUUAUAUAUCCCUUUGUUGGGUUUAUACCACUUAGC UUGAAAGAGGUUAAAACAUUACAAUUCAUUGUUAAGUUGAAUACAGCAAAaugggauccg ugagcaagggcgaggagcuguucaccgggguggugcccauccuggucgagcuggacggcgacguaaacggcc**

acaaguucagcguguccggcgagggcgagggcgaugccaccuacggcaagcugacccugaaguucaucugcac
caccggcaagcugcccgugcccuggcccacccucgugaccacccugaccuacggcgugcagugcuucagccgc
uaccccgaccacaugaagcagcacgacuucuucaaguccgccaugcccgaaggcuacguccaggagcgcacca
ucuucuucaaggacgacggcaacuacaagacccgcgccgaggugaaguucgagggcgacacccuggugaaccg
caucgagcugaagggcaucgacuucaaggaggacggcaacauccuggggcacaagcuggaguacaacuacaac
agccacaacgucuauaucauggccgacaagcagaagaacggcaucaaggugaacuucaagauccgccacaaca
ucgaggacggcagcgugcagcucgccgaccacuaccagcagaacacccccaucggcgacggccccgugcugcu
gcccgacaaccacuaccugagcacccaguccgcccugagcaaagaccccaacgagaagcgcgaucacaugguccc
ugcuggaguucgugaccgccgccgggaucacucucggcauggacgagcuguacaagagaucucauaugcauc
ucgagugauagucuagaccuucugcggggcuugccuucuggccaugcccuucuucucucccuugcaccugua
ccucuuggucuuuGAAUAAAGCCUGAGUAGGGGCUAUUAUGCGUUACCGGCGAGACGCUA
CGGACUUAAAUAAUUGAGCCUUAAAGAAGAAAUUCUUUAAGUGGAUGCUCUCAAACUCA
GGGAAACCUAAAUCUAGUUAUAGACAAGGCAAUCCUGAGCCAAGCCGAAGUAGUAAUUA
GUAAGACCAGUGGACAAUCGACGGAUAACAGCAUAUCUAG

MS2CP responsive Circular EGFP variant4 (**bold: circularized**)
Group I intron, CVB3 IRES, EGFP, MS2CP binding motif
GGGAGACCCUCGACCGUCGAUUGUCCACUGGUCAACAAUAGAUGACUUACAACUAAUCG
GAAGGUGCAGAGACUCGACGGGAGCUACCCUAACGUCAAGACGAGGGUAAAGAGAG
UCCAAUUCUCAAAGCCAAUAGGCAGUAGCGAAAGCUGCAAGAGAAUG**AAAAUCCGUUGA**
CCUUAAACGGUCGUGUGGGUUCAAGUCCCUCCACCCCCACGCCGGAAACGCAAUAGC
CGGCGAAUUAAGAGAGAAAAGAAGAGUAAGAAGAAAUAUAAGACACCGGUCGCCACC
UUAAAACAGCCUGUGGGUUGAUCCCACCCACAGGCCCAUUGGGCGCUAGCACUCUGG
UAUCACGGUACCUUUGUGCGCCUGUUUUAUACCCCCUCCCCCAACUGUAACUUAGAAG
UAACACACACCGAUCAACAGUCAGCGUGGCACACCAGCCACGUUUUGAUCAAGCACUU
CUGUUACCCCGGACUGAGUAUCAAUAGACUGCUCACGCGGUUGAAGGAGAAAGCGUU
CGUUAUCCGGCCAACUACUUCGAAAAACCUAGUAACACCGUGGAAGUUGCAGAGUGU
UUCGCUCAGCACUACCCCAGUGUAGAUCAGGUCGAUGAGUCACCGCAUUCCCCACGG
GCGACCGUGGCGGUGGCUGCGUUGGCGGCCUGCCCAUGGGGAAACCCAUGGGACGCU
CUAAUACAGACAUGGUGCGAAGAGUCUAUUGAGCUAGUUGGUAGUCCUCCGGCCCCU
GAAUGCGGCUAAUCCUAACUGCGGAGCACACACCCUCAAGCCAGAGGGCAGUGUGUC
GUAACGGGCAACUCUGCAGCGGAACCGACUACUUUGGGUGUCCGUGUUUCAUUUUAU
UCCUAUACUGGCUGCUUAUGGUGACAAUUGAGCAGGUGAGGAUCACCCAUCUGCCAC
GAGCGAGGUGAGGAUCACCCAUCUCGCUCGUGUUCGAUCGUUACCAUAUAGCUAUUG
GAUUGGCCAUCCGGUGACUAAUAGAGCUAUUAUAUCCCUUUGUUGGGUUUAUACC
ACUUAGCUUGAAAGAGGUUAAAACAUUACAAUUCAUUGUUAAGUUGAAUACAGCAAAa

ugggauccgugagcaagggcgaggagcuguucaccgggguggugcccauccuggucgagcuggacggcgacg
uaaacggccacaaguucagcguguccggcgagggcgagggcgaugccaccuacggcaagcugacccugaaguu
caucugcaccaccggcaagcugcccgugcccuggcccacccucgugaccacccugaccuacggcgugcagugc
uucagccgcuaccccgaccacaugaagcagcacgacuucuucaaguccgccaugcccgaaggcuacguccagg
agcgcaccaucuucuucaaggacgacggcaacuacaagacccgcgccgaggugaaguucgagggcgacacccu
ggugaaccgcaucgagcugaagggcaucgacuucaaggaggacggcaacauccuggggcacaagcuggaguac
aacuacaacagccacaacgucuauaucauggccgacaagcagaagaacggcaucaaggugaacuucaagaucc
gccacaacaucgaggacggcagcgugcagcucgccgaccacuaccagcagaacacccccaucggcgacggcccc
gugcugcugcccgacaaccacuaccugagcacccaguccgcccugagcaaagaccccaacgagaagcgcgauc
acaugguccugcuggaguucgugaccgccgccgggaucacucucggcauggacgagcuguacaagagaucuc
auaugcaucucgagugauagucuagaccuucugcggggcuugccuucuggccaugcccuucuucucucccuu
gcaccuguaccucuuggucuuuGAAUAAAGCCUGAGUAGGGGCUAUUAUGCGUUACCGGCG
AGACGCUACGGACUUAAAUAAUUGAGCCUUAAAGAAGAAAUUCUUUAAGUGGAUGCUCU
CAAACUCAGGGAAACCUAAAUCUAGUUAUAGACAAGGCAAUCCUGAGCCAAGCCGAAGU
AGUAAUUAGUAAGACCAGUGGACAAUCGACGGAUAACAGCAUAUCUAG

U1A responsive Circular EGFP variant1 (**bold: circularized**)
Group I intron, CVB3 IRES, EGFP, U1A binding motif
GGGAGACCCUCGACCGUCGAUUGUCCACUGGUCAACAAUAGAUGACUUACAACUAAUCG
GAAGGUGCAGAGACUCGACGGGAGCUACCCUAACGUCAAGACGAGGGUAAAGAGAGAG
UCCAAUUCUCAAAGCCAAUAGGCAGUAGCGAAAGCUGCAAGAGAAUG**AAAAUCCGUUGA**
CCUUAAACGGUCGUGUGGGUUCAAGUCCCUCCACCCCCACGCCGGAAACGCAAUAGC
CGGCGAAUUAAGAGAGAAAAGAAGAGUAAGAAGAAAUAUAAGACACCGGUCGCCACC
UUAAAACAGCCUGUGGGUUGAUCCCACCCACAGGCCCAUUGGGCGCUAGCACUCUGG
UAUCACGGUACCUUUGUGCGCCUGUUUUAUACCCCCUCCCCCAACUGUAACUUAGAAG
UAACACACACCGAUCAACAGUCAGCGUGGCACACCAGCCACGUUUUGAUCAAGCACUU
CUGUUACCCGACAGCAUUGUACCCAGAGUCUGUCCCCAGACAUUGCACCUGGCGCUG
UCUUAUCCGGCCAACUACUUCGAAAAACCUAGUAACACCGUGGAAGUUGCAGAGUGU
UUCGCUCAGCACUACCCCAGUGUAGAUCAGGUCGAUGAGUCACCGCAUUCCCCACGG
GCGACCGUGGCGGUGGCUGCGUUGGCGGCCUGCCCAUGGGGAAACCCAUGGGACGCU
CUAAUACAGACAUGGUGCGAAGAGUCUAUUGAGCUAGUUGGUAGUCCUCCGGCCCCU
GAAUGCGGCUAAUCCUAACUGCGGAGCACACACCCUCAAGCCAGAGGGCAGUGUGUC
GUAACGGGCAACUCUGCAGCGGAACCGACUACUUUGGGUGUCCGUGUUUCAUUUUAU
UCCUAUACUGGCUGCUUAUGGUGACAAUUGAGAGAUCGUUACCAUAUAGCUAUUGGA
UUGGCCAUCCGGUGACUAAUAGAGCUAUUAUAUCCCUUUGUUGGGUUUAUACCACU
UAGCUUGAAAGAGGUUAAAACAUUACAAUUCAUUGUUAAGUUGAAUACAGCAAAauggg

auccgugagcaagggcgaggagcuguucaccgggguggugcccauccuggucgagcuggacggcgacguaaa
cggccacaaguucagcguguccggcgagggcgagggcgaugccaccuacggcaagcugacccugaaguucauc
ugcaccaccggcaagcugcccgugcccuggcccacccucgugaccacccugaccuacggcgugcagugcuuca
gccgcuaccccgaccacaugaagcagcacgacuucuucaaguccgccaugcccgaaggcuacguccaggagcg
caccaucuucuucaaggacgacggcaacuacaagacccgcgccgaggugaaguucgagggcgacacccuggug
aaccgcaucgagcugaagggcaucgacuucaaggaggacggcaacauccuggggcacaagcuggaguacaacu
acaacagccacaacgucuauaucauggccgacaagcagaagaacggcaucaaggugaacuucaagauccgcca
caacaucgaggacggcagcgugcagcucgccgaccacuaccagcagaacacccccaucggcgacggccccgug
cugcugcccgacaaccacuaccugagcacccaguccgcccugagcaaagaccccaacgagaagcgcgaucacau
gguccugcuggaguucgugaccgccgccgggaucacucucggcauggacgagcuguacaagagaucucauau
gcaucucgagugauagucuagaccuucugcggggcuugccuucuggccaugcccuucuucucucccuugcac
cuguaccucuuggucuuuGAAUAAAGCCUGAGUAGGGGCUAUUAUGCGUUACCGGCGAGAC
GCUACGGACUUAAAUAAUUGAGCCUUAAAGAAGAAAUUCUUUAAGUGGAUGCUCUCAAA
CUCAGGGAAACCUAAAUCUAGUUAUAGACAAGGCAAUCCUGAGCCAAGCCGAAGUAGUA
AUUAGUAAGACCAGUGGACAAUCGACGGAUAACAGCAUAUCUAG

U1A responsive Circular EGFP variant2 (**bold: circularized**)

Group I intron, CVB3 IRES, EGFP, U1A binding motif

GGGAGACCCUCGACCGUCGAUUGUCCACUGGUCAACAAUAGAUGACUUACAACUAAUCG
GAAGGUGCAGAGACUCGACGGGAGCUACCCUAACGUCAAGACGAGGGUAAAGAGAGAG
UCCAAUUCUCAAAGCCAAUAGGCAGUAGCGAAAGCUGCAAGAGAAUG**AAAAUCCGUUGA**
CCUUAAACGGUCGUGUGGGUUCAAGUCCCUCCACCCCCACGCCGGAAACGCAAUAGC
CGGCGAAUUAAGAGAGAAAAGAAGAGUAAGAAGAAAUAUAAGACACCGGUCGCCACC
UUAAAACAGCCUGUGGGUUGAUCCCACCCACAGGCCCAUUGGGCGCUAGCACUCUGG
UAUCACGGUACCUUUGUGCGCCUGUUUUAUACCCCCUCCCCCAACUGUAACUUAGAAG
UAACACACACCGAUCAACAGUCAGCGUGGCACACCAGCCACGUUUUGAUCAAGCACUU
CUGUUACCCCGGACUGAGUAUCAAUAGACUGCUCACGCGGUUGAAGGAGAAAGCGUU
CGUUAUCCGGCCAACUACUUCGAUUGUACUAGUAACACCGUGGAAGUUGCAGAGUGU
UUCGCUCAGCACUACCCCAGUGUAGAUCAGGUCGAUGAGUCACCGCAUUCCCCACGG
GCGACCGUGGCGGUGGCUGCGUUGGCGGCCUGCCCAUGGGGAAACCCAUGGGACGCU
CUAAUACAGACAUGGUGAUUGCACGUCUAUUGAGCUAGUUGGUAGUCCUCCGGCCCC
UGAAUGCGGCUAAUCCUAACUGCGGAGCACACACCCUCAAGCCAGAGGGCAGUGUGU
CGUAACGGGCAACUCUGCAGCGGAACCGACUACUUUGGGUGUCCGUGUUUCAUUUUA
UUCCUAUACUGGCUGCUUAUGGUGACAAUUGAGAGAUCGUUACCAUAUAGCUAUUGG
AUUGGCCAUCCGGUGACUAAUAGAGCUAUUAUAUCCCUUUGUUGGGUUUAUACCAC
UUAGCUUGAAAGAGGUUAAAACAUUACAAUUCAUUGUUAAGUUGAAUACAGCAAAaug

ggauccgugagcaagggcgaggagcuguucaccgggguggugcccauccuggucgagcuggacggcgacgua
aacggccacaaguucagcguguccggcgagggcgagggcgaugccaccuacggcaagcugacccugaaguuca
ucugcaccaccggcaagcugcccgugcccuggcccacccucgugaccacccugaccuacggcgugcagugcuu
cagccgcuaccccgaccacaugaagcagcacgacuucuucaaguccgccaugcccgaaggcuacguccaggag
cgcaccaucuucuucaaggacgacggcaacuacaagacccgcgccgaggugaaguucgagggcgacacccugg
ugaaccgcaucgagcugaagggcaucgacuucaaggaggacggcaacauccuggggcacaagcuggaguacaa
cuacaacagccacaacgucuauaucauggccgacaagcagaagaacggcaucaaggugaacuucaagauccgc
cacaacaucgaggacggcagcgugcagcucgccgaccacuaccagcagaacacccccaucggcgacggccccg
ugcugcugcccgacaaccacuaccugagcacccaguccgcccugagcaaagaccccaacgagaagcgcgaucac
augguccugcuggaguucgugaccgccgccgggaucacucucggcauggacgagcuguacaagagaucucau
augcaucucgagugauagucuagaccuucugcggggcuugccuucuggccaugcccuucuucucucccuugc
accuguaccucuuggucuuuGAAUAAAGCCUGAGUAGGGGCUAUUAUGCGUUACCGGCGAG
ACGCUACGGACUUAAAUAAUUGAGCCUUAAAGAAGAAAUUCUUUAAGUGGAUGCUCUCA
AACUCAGGGAAACCUAAAUCUAGUUAUAGACAAGGCAAUCCUGAGCCAAGCCGAAGUAG
UAAUUAGUAAGACCAGUGGACAAUCGACGGAUAACAGCAUAUCUAG

U1A responsive Circular EGFP variant3 (**bold: circularized**)

Group I intron, CVB3 IRES, EGFP, U1A binding motif

GGGAGACCCUCGACCGUCGAUUGUCCACUGGUCAACAAUAGAUGACUUACAACUAAUCG
GAAGGUGCAGAGACUCGACGGGAGCUACCCUAACGUCAAGACGAGGGUAAAGAGAG
UCCAAUUCUCAAAGCCAAUAGGCAGUAGCGAAAGCUGCAAGAGAAUG**AAAAUCCGUUGA**
CCUUAAACGGUCGUGUGGGUUCAAGUCCCUCCACCCCCACGCCGGAAACGCAAUAGC
CGGCGAAUUAAGAGAGAAAAGAAGAGUAAGAAGAAAUAUAAGACACCGGUCGCCACC
UUAAAACAGCCUGUGGGUUGAUCCCACCCACAGGCCCAUUGGGCGCUAGCACUCUGG
UAUCACGGUACCUUUGUGCGCCUGUUUUAUACCCCCUCCCCCAACUGUAACUUAGAAG
UAACACACACCGAUCAACAGUCAGCGUGGCACACCAGCCACGUUUUGAUCAAGCACUU
CUGUUACCCCGGACUGAGUAUCAAUAGACUGCUCACGCGGUUGAAGGAGAAAGCGUU
CGUUAUCCGGCCAACUACUUCGAAAAACCUAGUAACACCGUGGAAGUUGCAGAGUGU
UUCGCUCAGCACUACCCCAGUGUAGAUCAGGUCGAUGAGUCACCGCAUUCCCCACGG
GCGACCGUGGCGGUGGCUGCGUUGGCGGCCUGCCCAUGGGGAAACCCAUGGGACGCU
CUAAUACAGACAUGGUGCGAAGAGUCUAUUGAGCUAGUUGGUAGUCCUCCGGCCCCU
GAAUGCGGCUAAUCCUAACUGCGGAGCACACACCCUCACAGCAUUGUACCCAGAGUC
UGUCCCCAGACAUUGCACCUGGCGCUGUGAGGGCAGUGUGUCGUAACGGGCAACUCU
GCAGCGGAACCGACUACUUUGGGUGUCCGUGUUUCAUUUUAUUCCUAUACUGGCUGC
UUAUGGUGACAAUUGAGAGAUCGUUACCAUAUAGCUAUUGGAUUGGCCAUCCGGUGA
CUAAUAGAGCUAUUAUAUAUCCCUUUGUUGGGUUUAUACCACUUAGCUUGAAGAGGU

UAAAACAUUACAAUUCAUUGUUAAGUUGAAUACAGCAAAaugggauccgugagcaagggcgag
gagcuguucaccgggguggugcccauccuggucgagcuggacggcgacguaaacggccacaaguucagcgug
uccggcgagggcgagggcgaugccaccuacggcaagcugacccugaaguucaucugcaccaccggcaagcugc
ccgugcccuggcccacccucgugaccacccugaccuacggcgugcagugcuucagccgcuaccccgaccacau
gaagcagcacgacuucuucaaguccgccaugcccgaaggcuacguccaggagcgcaccaucuucuucaaggac
gacggcaacuacaagacccgcgccgaggugaaguucgagggcgacacccuggugaaccgcaucgagcugaagg
gcaucgacuucaaggaggacggcaacauccuggggcacaagcuggaguacaacuacaacagccacaacgucua
uaucauggccgacaagcagaagaacggcaucaaggugaacuucaagauccgccacaacaucgaggacggcagc
gugcagcucgccgaccacuaccagcagaacacccccaucggcgacggccccgugcugcugcccgacaaccacu
accugagcacccaguccgcccugagcaaagaccccaacgagaagcgcgaucacaugguccugcuggaguucgu
gaccgccgccgggaucacucucggcauggacgagcuguacaagagaucucauaugcaucucgagugauaguc
uagaccuucugcggggcuugccuucuggccaugcccuucuucucucccuugcaccuguaccucuuggucuuu
GAAUAAAGCCUGAGUAGGGGCUAUUAUGCGUUACCGGCGAGACGCUACGGACUUAAA
UAAUUGAGCCUUAAAGAAGAAAUUCUUUAAGUGGAUGCUCUCAAACUCAGGGAAACCUA
AAUCUAGUUAUAGACAAGGCAAUCCUGAGCCAAGCCGAAGUAGUAAUUAGUAAGACCAG
UGGACAAUCGACGGAUAACAGCAUAUCUAG

U1A responsive Circular EGFP variant4 (**bold: circularized**)

Group I intron, CVB3 IRES, EGFP, U1A binding motif

GGGAGACCCUCGACCGUCGAUUGUCCACUGGUCAACAAUAGAUGACUUACAACUAAUCG
GAAGGUGCAGAGACUCGACGGGAGCUACCCUAACGUCAAGACGAGGGUAAAGAGAG
UCCAAUUCUCAAAGCCAAUAGGCAGUAGCGAAAGCUGCAAGAGAAUG**AAAAUCCGUUGA**
CCUUAAACGGUCGUGUGGGUUCAAGUCCCUCCACCCCCACGCCGGAAACGCAAUAGC
CGGCGAAUUAAGAGAGAAAAGAAGAGUAAGAAGAAAUAUAAGACACCGGUCGCCACC
UUAAAACAGCCUGUGGGUUGAUCCCACCCACAGGCCCAUUGGGCGCUAGCACUCUGG
UAUCACGGUACCUUUGUGCGCCUGUUUUAUACCCCCUCCCCCAACUGUAACUUAGAAG
UAACACACACCGAUCAACAGUCAGCGUGGCACACCAGCCACGUUUUGAUCAAGCACUU
CUGUUACCCCGGACUGAGUAUCAAUAGACUGCUCACGCGGUUGAAGGAGAAAGCGUU
CGUUAUCCGGCCAACUACUUCGAAAAACCUAGUAACACCGUGGAAGUUGCAGAGUGU
UUCGCUCAGCACUACCCCAGUGUAGAUCAGGUCGAUGAGUCACCGCAUUCCCCACGG
GCGACCGUGGCGGUGGCUGCGUUGGCGGCCUGCCCAUGGGGAAACCCAUGGGACGCU
CUAAUACAGACAUGGUGCGAAGAGUCUAUUGAGCUAGUUGGUAGUCCUCCGGCCCCU
GAAUGCGGCUAAUCCUAACUGCGGAGCACACACCCUCAAGCCAGAGGGCAGUGUGUC
GUAACGGGCAACUCUGCAGCGGAACCGACUACUUUGGGUGUCCGUGUUUCAUUUUAU
UCCUAUACUGGCUGCUUAUGGUGACAAUUGAGAGAUCGUUACCAUAUAGCUAUUGGA
UUGGCGACAGCAUUGUACCCAGAGUCUGUCCCCAGACAUUGCACCUGGCGCUGUCCA

UCCGGUGACUAAUAGAGCUAUUAUAUCCCUUUGUUGGGUUUAUACCACUUAGCUUG
AAAGAGGUUAAAACAUUACAAUUCAUUGUUAAGUUGAAUACAGCAAAaugggauccguga
gcaagggcgaggagcuguucaccgggguggugcccauccuggucgagcuggacggcgacguaaacggccaca
aguucagcguguccggcgagggcgagggcgaugccaccuacggcaagcugacccugaaguucaucugcaccac
cggcaagcugcccgugcccuggcccacccucgugaccacccugaccuacggcgugcagugcuucagccgcuac
cccgaccacaugaagcagcacgacuucuucaaguccgccaugcccgaaggcuacguccaggagcgcaccaucu
ucuucaaggacgacggcaacuacaagacccgcgccgaggugaaguucgagggcgacacccuggugaaccgcau
cgagcugaagggcaucgacuucaaggaggacggcaacauccuggggcacaagcuggaguacaacuacaacagc
cacaacgucuauaucauggccgacaagcagaagaacggcaucaaggugaacuucaagauccgccacaacaucg
aggacggcagcgugcagcucgccgaccacuaccagcagaacacccccaucggcgacggccccgugcugcugcc
cgacaaccacuaccugagcacccaguccgcccugagcaaagaccccaacgagaagcgcgaucacaugguccugc
uggaguucgugaccgccgccgggaucacucucggcauggacgagcuguacaagagaucucauaugcaucucg
agugauagucuagaccuucugcggggcuugccuucuggccaugcccuucuucucucccuugcaccuguaccu
cuuggucuuuGAAUAAAGCCUGAGUAGGGGCUAUUAUGCGUUACCGGCGAGACGCUACG
GACUUAAAUAAUUGAGCCUUAAAGAAGAAAUUCUUUAAGUGGAUGCUCUCAAACUCAGG
GAAACCUAAAUCUAGUUAUAGACAAGGCAAUCCUGAGCCAAGCCGAAGUAGUAAUUAGU
AAGACCAGUGGACAAUCGACGGAUAACAGCAUAUCUAG

MS2CP responsive A-cap Linear EGFP variant4

Group I intron, CVB3 IRES, EGFP, MS2CP binding motif

GGGAAAAUCCGUUGACCUUAAACGGUCGUGUGGGUUCAAGUCCCUCCACCCCCACGCC
GGAAACGCAAUAGCCGGCGAAUUAAGAGAGAAAAGAAGAGUAAGAAGAAAUAUAAGACAC
CGGUCGCCACCUUAAAACAGCCUGUGGGUUGAUCCCACCCACAGGCCCAUUGGGCGCU
AGCACUCUGGUAUCACGGUACCUUUGUGCGCCUGUUUUAUACCCCCUCCCCCAACUGU
AACUUAGAAGUAACACACACCGAUCAACAGUCAGCGUGGCACACCAGCCACGUUUUGAU
CAAGCACUUCUGUUACCCCGGACUGAGUAUCAAUAGACUGCUCACGCGGUUGAAGGAG
AAAGCGUUCGUUAUCCGGCCAACUACUUCGAAAAACCUAGUAACACCGUGGAAGUUGCA
GAGUGUUUCGCUCAGCACUACCCCAGUGUAGAUCAGGUCGAUGAGUCACCGCAUUCCC
CACGGGCGACCGUGGCGGUGGCUGCGUUGGCGGCCUGCCCAUGGGGAAACCCAUGGG
ACGCUCUAAUACAGACAUGGUGCGAAGAGUCUAUUGAGCUAGUUGGUAGUCCUCCGGC
CCCUGAAUGCGGCUAAUCCUAACUGCGGAGCACACACCCUCAAGCCAGAGGGCAGUGU
GUCGUAACGGGCAACUCUGCAGCGGAACCGACUACUUUGGGUGUCCGUGUUUCAUUUU
AUUCCUAUACUGGCUGCUUAUGGUGACAAUUGAGCAGGUGAGGAUCACCCAUCUGCCA
CGAGCGAGGUGAGGAUCACCCAUCUCGCUCGUGUUCGAUCGUUACCAUAUAGCUAUUG
GAUUGGCCAUCCGGUGACUAAUAGAGCUAUUAUAUCCCUUUGUUGGGUUUAUACCAC
UUAGCUUGAAAGAGGUUAAAACAUUACAAUUCAUUGUUAAGUUGAAUACAGCAAAauggga

uccgugagcaagggcgaggagcuguucaccgggguggugcccauccuggucgagcuggacggcgacguaaacggccac
aaguucagcguguccggcgagggcgagggcgaugccaccuacggcaagcugacccugaaguucaucugcaccaccggc
aagcugcccgugcccuggcccacccucgugaccacccugaccuacggcgugcagugcuucagccgcuaccccgaccacau
gaagcagcacgacuucuucaaguccgccaugcccgaaggcuacguccaggagcgcaccaucuucuucaaggacgacggc
aacuacaagacccgcgccgaggugaaguucgagggcgacacccuggugaaccgcaucgagcugaagggcaucgacuuc
aaggaggacggcaacauccuggggcacaagcuggaguacaacuacaacagccacaacgucuauaucauggccgacaag
cagaagaacggcaucaaggugaacuucaagauccgccacaacaucgaggacggcagcgugcagcucgccgaccacuacc
agcagaacacccccaucggcgacggccccgugcugcugcccgacaaccacuaccugagcacccaguccgcccugagcaa
agaccccaacgagaagcgcgaucacaugguccugcuggaguucgugaccgccgccgggaucacucucggcauggacga
gcuguacaagagaucucauaugcaucucgagugauagucuagaccuucugcggggcuugccuucuggccaugcccuuc
uucucucccuugcaccuguaccucuuggucuuuGAAUAAAGCCUGAGUAGGGGCUAUUAUGCGUUAC
CGGCGAGACGCUACGGACUUAAAAAAAAAAAAAAAAAAAAAAAAAAAAAAAAAAAAAAAAAAAAA
AAAAAAAAAAAAAAAAAAAAAAAAAAAAAAAAAAAAAAAAAAAAAAAAAAAAAAAAAAAAAAA
AAAAAAAAAAAAAAAA

MS2CP responsive A-cap Linear EGFP variant5

Group I intron, CVB3 IRES, EGFP, MS2CP binding motif

GGGAAAAUCCGUUGACCUUAAACGGUCGUGUGGGUUCAAGUCCCUCCACCCCCACGCC
GGAAACGCAAUAGCCGGCGAAUUAAGAGAGAAAAGAAGAGUAAGAAGAAAUAUAAGACAC
CGGUCGCCACCUUAAAACAGCCUGUGGGUUGAUCCCACCCACAGGCCCAUUGGGCGCU
AGCACUCUGGUAUCACGGUACCUUUGUGCGCCUGUUUUAUACCCCCUCCCCCAACUGU
AACUUAGAAGUAACACACACCGAUCAACAGUCAGCGUGGCACACCAGCCACGUUUUGAU
CAAGCACUUCUGUUACCCCGGACUGAGUAUCAAUAGACUGCUCACGCGGUUGAAGGAG
AAAGCGUUCGUUAUCCGGCCAACUACUUCGAAAAACCUAGUAACACCGUGGAAGUUGCA
GAGUGUUUCGCUCAGCACUACCCCAGUGUAGAUCAGGUCGAUGAGUCACCGCAUUCCC
CACGGGCGACCGUGGCGGUGGCUGCGUUGGCGGCCUGCCCAUGGGGAAACCCAUGGG
ACGCUCUAAUACAGACAUGGUGCGAAGAGUCUAUUGAGCUAGUUGGUAGUCCUCCGGC
CCCUGAAUGCGGCUAAUCCUAACUGCGGAGCACACACCCUCAAGCCAGAGGGCAGUGU
GUCGUAACGGGCAACUCUGCAGCGGAACCGACUACUUUGGGUGUCCGUGUUUCAUUUU
AUUCCUAUACUGGCUGCUUAUGGUGACAAUUGAGAGAUCGUUACCAUAUAGCUAUUGGA
UUGGCCAUCCGGUGACUAAUAGAGCUAUUAUAUAGAGCAGGUGAGGAUCACCCAUCUG
CCACGAGCGAGGUGAGGAUCACCCAUCUCGCUCGUGUUCUCCCUUUGUUGGGUUUAUA
CCACUUAGCUUGAAAGAGGUUAAAACAUUACAAUUCAUUGUUAAGUUGAAUACAGCAAAa
ugggauccgugagcaagggcgaggagcuguucaccgggguggugcccauccuggucgagcuggacggcgacguaaacg
gccacaaguucagcguguccggcgagggcgagggcgaugccaccuacggcaagcugacccugaaguucaucugcaccac
cggcaagcugcccgugcccuggcccacccucgugaccacccugaccuacggcgugcagugcuucagccgcuaccccgacc

acaugaagcagcacgacuucuucaaguccgccaugcccgaaggcuacguccaggagcgcaccaucuucuucaaggacga
cggcaacuacaagacccgcgccgaggugaaguucgagggcgacacccuggugaaccgcaucgagcugaagggcaucga
cuucaaggaggacggcaacauccuggggcacaagcuggaguacaacuacaacagccacaacgucuauaucauggccga
caagcagaagaacggcaucaaggugaacuucaagauccgccacaacaucgaggacggcagcgugcagcucgccgacca
cuaccagcagaacacccccaucggcgacggccccgugcugcugcccgacaaccacuaccugagcacccaguccgcccuga
gcaaagaccccaacgagaagcgcgaucacaugguccugcuggaguucgugaccgccgccgggaucacucucggcaugg
acgagcuguacaagagaucucauaugcaucucgagugauagucuagaccuucugcggggcuugccuucuggccaugcc
cuucuucucucccuugcaccuguaccucuuggucuuuGAAUAAAGCCUGAGUAGGGGCUAUUAUGCGU
UACCGGCGAGACGCUACGGACUUAAAAAAAAAAAAAAAAAAAAAAAAAAAAAAAAAAAAAA
AAAAAAAAAAAAAAAAAAAAAAAAAAAAAAAAAAAAAAAAAAAAAAAAAAAAAAAAAAAAAAA
AAAAAAAAAAAAAAAAAAA

MS2CP responsive A-cap Linear EGFP variant6

Group I intron, CVB3 IRES, EGFP, MS2CP binding motif

GGGAAAAUCCGUUGACCUUAAACGGUCGUGUGGGUUCAAGUCCCUCCACCCCCACGCC
GGAAACGCAAUAGCCGGCGAAUUAAGAGAGAAAAGAAGAGUAAGAAGAAAUAUAAGACAC
CGGUCGCCACCUUAAAACAGCCUGUGGGUUGAUCCCACCCACAGGCCCAUUGGGCGCU
AGCACUCUGGUAUCACGGUACCUUUGUGCGCCUGUUUUAUACCCCCUCCCCCAACUGU
AACUUAGAAGUAACACACACCGAUCAACAGUCAGCGUGGCACACCAGCCACGUUUUGAU
CAAGCACUUCUGUUACCCCGGACUGAGUAUCAAUAGACUGCUCACGCGGUUGAAGGAG
AAAGCGUUCGUUAUCCGGCCAACUACUUCGAAAAACCUAGUAACACCGUGGAAGUUGCA
GAGUGUUUCGCUCAGCACUACCCCAGUGUAGAUCAGGUCGAUGAGUCACCGCAUUCCC
CACGGGCGACCGUGGCGGUGGCUGCGUUGGCGGCCUGCCCAUGGGGAAACCCAUGGG
ACGCUCUAAUACAGACAUGGUGCGAAGAGUCUAUUGAGCUAGUUGGUAGUCCUCCGGC
CCCUGAAUGCGGCUAAUCCUAACUGCGGAGCACACACCCUCAAGCCAGAGGGCAGUGU
GUCGUAACGGGCAACUCUGCAGCGGAACCGACUACUUUGGGUGUCCGUGUUUCAUUUU
AUUCCUAUACUGGCUGCUUAUGGUGACAAUUGAGAGAUCGUUACCAUAUAGCUAUUGGA
UUGGCCAUCCGGUGACUAAUAGAGCUAUUAUAUCCCUUUGUUGGGUUUAUACCACUU
AGCUUGAAAGAGGUUAAAACAUUACAAUUCAUUGUUAAGUUGAAUACAGCAAAGAGCAG
GUGAGGAUCACCCAUCUGCCACGAGCGAGGUGAGGAUCACCCAUCUCGCUCGUGUUCa
ugggauccgugagcaagggcgaggagcuguucaccgggguggugcccauccuggucgagcuggacggcgacguaaacg
gccacaaguucagcguguccggcgagggcgagggcgaugccaccuacggcaagcugacccugaaguucaucugcaccac
cggcaagcugcccgugcccuggcccacccucgugaccacccugaccuacggcgugcagugcuucagccgcuaccccgacc
acaugaagcagcacgacuucuucaaguccgccaugcccgaaggcuacguccaggagcgcaccaucuucuucaaggacga
cggcaacuacaagacccgcgccgaggugaaguucgagggcgacacccuggugaaccgcaucgagcugaagggcaucga
cuucaaggaggacggcaacauccuggggcacaagcuggaguacaacuacaacagccacaacgucuauaucauggccga

caagcagaagaacggcaucaaggugaacuucaagauccgccacaacaucgaggacggcagcgugcagcucgccgacca
cuaccagcagaacacccccaucggcgacggccccgugcugcugcccgacaaccacuaccugagcacccaguccgcccuga
gcaaagaccccaacgagaagcgcgaucacauggucc ugcuggaguucgugaccgccgccgggaucacucucggcaugg
acgagcuguacaagagaucucauaugcaucucgagugauagucuagaccuucugcggggcuugccuucuggccaugcc
cuucuucucucccuugcaccuguaccucuuggucuuuGAAUAAAGCCUGAGUAGGGCUAUUAUGCGU
UACCGGCGAGACGCUACGGACUUAAAAAAAAAAAAAAAAAAAAAAAAAAAAAAAAAAAAAA
AAAAAAAAAAAAAAAAAAAAAAAAAAAAAAAAAAAAAAAAAAAAAAAAAAAAAAAAAAAAAAA
AAAAAAAAAAAAAAAAAAA

U1A responsive A-cap Linear EGFP variant4

Group I intron, CVB3 IRES, EGFP, U1A binding motif

GGGAAAAUCCGUUGACCUUAAACGGUCGUGUGGGUUCAAGUCCCUCCACCCCCACGCC
GGAAACGCAAUAGCCGGCGAAUUAAGAGAGAAAAGAAGAGUAAGAAGAAAUAUAAGACAC
CGGUCGCCACCUUAAAACAGCCUGUGGGUUGAUCCCACCCACAGGCCCAUUGGGCGCU
AGCACUCUGGUAUCACGGUACCUUUGUGCGCCUGUUUUAUACCCCCUCCCCCAACUGU
AACUUAGAAGUAACACACACCGAUCAACAGUCAGCGUGGCACACCAGCCACGUUUUGAU
CAAGCACUUCUGUUACCCCGGACUGAGUAUCAAUAGACUGCUCACGCGGUUGAAGGAG
AAAGCGUUCGUUAUCCGGCCAACUACUUCGAAAAACCUAGUAACACCGUGGAAGUUGCA
GAGUGUUUCGCUCAGCACUACCCCAGUGUAGAUCAGGUCGAUGAGUCACCGCAUUCCC
CACGGGCGACCGUGGCGGUGGCUGCGUUGGCGGCCUGCCCAUGGGGAAACCCAUGGG
ACGCUCUAAUACAGACAUGGUGCGAAGAGUCUAUUGAGCUAGUUGGUAGUCCUCCGGC
CCCUGAAUGCGGCUAAUCCUAACUGCGGAGCACACACCCUCAAGCCAGAGGGCAGUGU
GUCGUAACGGGCAACUCUGCAGCGGAACCGACUACUUUGGGUGUCCGUGUUUCAUUUU
AUUCCUAUACUGGCUGCUUAUGGUGACAAUUGAGAGAUCGUUACCAUAUAGCUAUUGGA
UUGGCGACAGCAUUGUACCCAGAGUCUGUCCCCAGACAUUGCACCUGGCGCUGUCCAU
CCGGUGACUAAUAGAGCUAUUAUAUCCCUUUGUUGGGUUUAUACCACUUAGCUUGAA
AGAGGUUAAAACAUUACAAUUCAUUGUUAAGUUGAAUACAGCAAAaugggauccgugagcaagg
gcgaggagcuguucaccgggguggugcccauccuggucgagcuggacggcgacguaaacggccacaaguucagcgugu
ccggcgagggcgagggcgaugccaccuacggcaagcugacccugaaguucaucugcaccaccggcaagcugcccgugcc
cuggcccacccucgugaccacccugaccuacggcgugcagugcuucagccgcuaccccgaccacaugaagcagcacgac
uucuucaaguccgccaugcccgaaggcuacguccaggagcgcaccaucuucuucaaggacgacggcaacuacaagaccc
gcgccgaggugaaguucgagggcgacacccuggugaaccgcaucgagcugaagggcaucgacuucaaggaggacggca
acauccuggggcacaagcuggaguacaacuacaacagccacaacgucuauaucauggccgacaagcagaagaacggca
ucaaggugaacuucaagauccgccacaacaucgaggacggcagcgugcagcucgccgaccacuaccagcagaacacccc
caucggcgacggccccgugcugcugcccgacaaccacuaccugagcacccaguccgcccugagcaaagaccccaacgag
aagcgcgaucacaugguccugcuggaguucgugaccgccgccgggaucacucucggcauggacgagcuguacaagaga

ucucauaugcaucucgagugauagucuagaccuucugcggggcuugccuucuggccaugcccuucuucucucccuugca
ccuguaccucuuggucuuuGAAUAAAGCCUGAGUAGGGGCUAUUAUGCGUUACCGGCGAGACG
CUACGGACUUAAAAAAAAAAAAAAAAAAAAAAAAAAAAAAAAAAAAAAAAAAAAAAAAAA
AAAAAAAAAAAAAAAAAAAAAAAAAAAAAAAAAAAAAAAAAAAAAAAAAAAAAAAAAAAAAAAA
AAAAA

U1A responsive A-cap Linear EGFP variant5

Group I intron, CVB3 IRES, EGFP, U1A binding motif

GGGAAAAUCCGUUGACCUUAAACGGUCGUGUGGGUUCAAGUCCCUCCACCCCCACGCC
GGAAACGCAAUAGCCGGCGAAUUAAGAGAGAAAAGAAGAGUAAGAAGAAAUAUAAGACAC
CGGUCGCCACCUUAAAACAGCCUGUGGGUUGAUCCCACCCACAGGCCCAUUGGGCGCU
AGCACUCUGGUAUCACGGUACCUUUGUGCGCCUGUUUUAUACCCCCUCCCCCAACUGU
AACUUAGAAGUAACACACACCGAUCAACAGUCAGCGUGGCACACCAGCCACGUUUUGAU
CAAGCACUUCUGUUACCCCGGACUGAGUAUCAAUAGACUGCUCACGCGGUUGAAGGAG
AAAGCGUUCGUUAUCCGGCCAACUACUUCGAAAAACCUAGUAACACCGUGGAAGUUGCA
GAGUGUUUCGCUCAGCACUACCCCAGUGUAGAUCAGGUCGAUGAGUCACCGCAUUCCC
CACGGGCGACCGUGGCGGUGGCUGCGUUGGCGGCCUGCCCAUGGGGAAACCCAUGGG
ACGCUCUAAUACAGACAUGGUGCGAAGAGUCUAUUGAGCUAGUUGGUAGUCCUCCGGC
CCCUGAAUGCGGCUAAUCCUAACUGCGGAGCACACACCCUCAAGCCAGAGGGCAGUGU
GUCGUAACGGGCAACUCUGCAGCGGAACCGACUACUUUGGGUGUCCGUGUUUCAUUUU
AUUCCUAUACUGGCUGCUUAUGGUGACAAUUGAGAGAUCGUUACCAUAUAGCUAUUGGA
UUGGCCAUCCGGUGACUAAUAGAGCUAUUAUAUAGACAGCAUUGUACCCAGAGUCUGUC
CCCAGACAUUGCACCUGGCGCUGUCUCCCUUUGUUGGGUUUAUACCACUUAGCUUGAA
AGAGGUUAAAACAUUACAAUUCAUUGUUAAGUUGAAUACAGCAAAaugggauccgugagcaagg
gcgaggagcuguucaccgggguggugcccauccuggucgagcuggacggcgacguaaacggccacaaguucagcgugu
ccggcgagggcgagggcgaugccaccuacggcaagcugacccugaaguucaucugcaccaccggcaagcugcccgugcc
cuggcccacccucgugaccacccugaccuacggcgugcagugcuucagccgcuaccccgaccacaugaagcagcacgac
uucuucaaguccgccaugcccgaaggcuacguccaggagcgcaccaucuucuucaaggacgacggcaacuacaagaccc
gcgccgaggugaaguucgagggcgacacccuggugaaccgcaucgagcugaagggcaucgacuucaaggaggacggca
acauccuggggcacaagcuggaguacaacuacaacagccacaacgucuauaucauggccgacaagcagaagaacggca
ucaaggugaacuucaagauccgccacaacaucgaggacggcagcgugcagcucgccgaccacuaccagcagaacacccc
caucggcgacggccccgugcugcugcccgacaaccacuaccugagcacccaguccgcccugagcaaagaccccaacgag
aagcgcgaucacaugguccugcuggaguucgugaccgccgccgggaucacucucggcauggacgagcuguacaagaga
ucucauaugcaucucgagugauagucuagaccuucugcggggcuugccuucuggccaugcccuucuucucucccuugca
ccuguaccucuuggucuuuGAAUAAAGCCUGAGUAGGGGCUAUUAUGCGUUACCGGCGAGACG
CUACGGACUUAAAAAAAAAAAAAAAAAAAAAAAAAAAAAAAAAAAAAAAAAAAAAAAAAA

AAAAAAAAAAAAAAAAAAAAAAAAAAAAAAAAAAAAAAAAAAAAAAAAAAAAAAAAAAAAAAAA
AAAAA

U1A responsive A-cap Linear EGFP variant6

Group I intron, CVB3 IRES, EGFP, U1A binding motif

GGGAAAAUCCGUUGACCUUAAACGGUCGUGUGGGUUCAAGUCCCUCCACCCCCACGCC
GGAAACGCAAUAGCCGGCGAAUUAAGAGAGAAAAGAAGAGUAAGAAGAAAUAUAAGACAC
CGGUCGCCACC
UUAAAACAGCCUGUGGGUUGAUCCCACCCACAGGCCCAUUGGGCGCUAGCACUCUGGU
AUCACGGUACCUUUGUGCGCCUGUUUUAUACCCCCUCCCCCAACUGUAACUUAGAAGUA
ACACACACCGAUCAACAGUCAGCGUGGCACACCAGCCACGUUUUGAUCAAGCACUUCUG
UUACCCCGGACUGAGUAUCAAUAGACUGCUCACGCGGUUGAAGGAGAAAGCGUUCGUU
AUCCGGCCAACUACUUCGAAAAACCUAGUAACACCGUGGAAGUUGCAGAGUGUUUCGCU
CAGCACUACCCCAGUGUAGAUCAGGUCGAUGAGUCACCGCAUUCCCCACGGGCGACCG
UGGCGGUGGCUGCGUUGGCGGCCUGCCCAUGGGGAAACCCAUGGGACGCUCUAAUACA
GACAUGGUGCGAAGAGUCUAUUGAGCUAGUUGGUAGUCCUCCGGCCCCUGAAUGCGGC
UAAUCCUAACUGCGGAGCACACACCCUCAAGCCAGAGGGCAGUGUGUCGUAACGGGCA
ACUCUGCAGCGGAACCGACUACUUUGGGUGUCCGUGUUUCAUUUUAUUCCUAUACUGG
CUGCUUAUGGUGACAAUUGAGAGAUCGUUACCAUAUAGCUAUUGGAUUGGCCAUCCGG
UGACUAAUAGAGCUAUUAUAUCCCUUUGUUGGGUUUAUACCACUUAGCUUGAAAGAG
GUUAAAACAUUACAAUUCAUUGUUAAGUUGAAUACAGCAAAGACAGCAUUGUACCCAGA
GUCUGUCCCCAGACAUUGCACCUGGCGCUGUCaugggauccgugagcaagggcgaggagcuguucac
cgggguggugcccauccuggucgagcuggacggcgacguaaacggccacaaguucagcguguccggcgagggcgaggg
cgaugccaccuacggcaagcugacccugaaguucaucugcaccaccggcaagcugcccgugcccuggcccacccucgug
accacccugaccuacggcgugcagugcuucagccgcuaccccgaccacaugaagcagcacgacuucuucaaguccgcca
ugcccgaaggcuacguccaggagcgcaccaucuucuucaaggacgacggcaacuacaagacccgcgccgaggugaagu
ucgagggcgacacccuggugaaccgcaucgagcugaagggcaucgacuucaaggaggacggcaacauccuggggcaca
agcuggaguacaacuacaacagccacaacgucuauaucauggccgacaagcagaagaacggcaucaaggugaacuuca
agauccgccacaacaucgaggacggcagcgugcagcucgccgaccacuaccagcagaacacccccaucggcgacggccc
cgugcugcugcccgacaaccacuaccugagcacccaguccgcccugagcaaagaccccaacgagaagcgcgaucacaug
guccugcuggaguucgugaccgccgccgggaucacucucggcauggacgagcuguacaagagaucucauaugcaucuc
gagugauagucuagaccuucugcggggcuugccuucuggccaugcccuucuucucucccuugcaccuguaccucuuggu
cuuuGAAUAAAGCCUGAGUAGGGGCUAUUAUGCGUUACCGGCGAGACGCUACGGACUUA
AAAAAAAAAAAAAAAAAAAAAAAAAAAAAAAAAAAAAAAAAAAAAAAAAAAAAAAAAAAAAAAA
AAAAAAAAAAAAAAAAAAAAAAAAAAAAAAAAAAAAAAAAAAAAAAAAAAAAAAAAA

MS2CP responsive Circular EGFP +pA variant4 (**bold: circularized**)

Group I intron, CVB3 IRES, EGFP, MS2CP binding motif

GGGAGACCCUCGACCGUCGAUUGUCCACUGGUCAACAAUAGAUGACUUACAACUAAUCG
GAAGGUGCAGAGACUCGACGGGAGCUACCCUAACGUCAAGACGAGGGUAAAGAGAG
UCCAAUUCUCAAAGCCAAUAGGCAGUAGCGAAAGCUGCAAGAGAAUG**AAAAUCCGUUGA**
CCUUAAACGGUCGUGUGGGUUCAAGUCCCUCCACCCCCACGCCGGAAACGCAAUAGC
CGGCGAAUUAAGAGAGAAAAGAAGAGUAAGAAGAAAUAUAAGACACCGGUCGCCACC
UUAAAACAGCCUGUGGGUUGAUCCCACCCACAGGCCCAUUGGGCGCUAGCACUCUGG
UAUCACGGUACCUUUGUGCGCCUGUUUUAUACCCCCUCCCCCAACUGUAACUUAGAAG
UAACACACACCGAUCAACAGUCAGCGUGGCACACCAGCCACGUUUUGAUCAAGCACUU
CUGUUACCCCGGACUGAGUAUCAAUAGACUGCUCACGCGGUUGAAGGAGAAAGCGUU
CGUUAUCCGGCCAACUACUUCGAAAAACCUAGUAACACCGUGGAAGUUGCAGAGUGU
UUCGCUCAGCACUACCCCAGUGUAGAUCAGGUCGAUGAGUCACCGCAUUCCCCACGG
GCGACCGUGGCGGUGGCUGCGUUGGCGGCCUGCCCAUGGGGAAACCCAUGGGACGCU
CUAAUACAGACAUGGUGCGAAGAGUCUAUUGAGCUAGUUGGUAGUCCUCCGGCCCCU
GAAUGCGGCUAAUCCUAACUGCGGAGCACACACCCUCAAGCCAGAGGGCAGUGUGUC
GUAACGGGCAACUCUGCAGCGGAACCGACUACUUUGGGUGUCCGUGUUUCAUUUUAU
UCCUAUACUGGCUGCUUAUGGUGACAAUUGAGCAGGUGAGGAUCACCCAUCUGCCAC
GAGCGAGGUGAGGAUCACCCAUCUCGCUCGUGUUCGAUCGUUACCAUAUAGCUAUUG
GAUUGGCCAUCCGGUGACUAAUAGAGCUAUUAUAUCCCUUUGUUGGGUUUAUACC
ACUUAGCUUGAAAGAGGUUAAAACAUUACAAUUCAUUGUUAAGUUGAAUACAGCAAAa
ugggauccgugagcaagggcgaggagcuguucaccgggguggugcccauccuggucgagcuggacggcgacg
uaaacggccacaaguucagcguguccggcgagggcgagggcgaugccaccuacggcaagcugacccugaaguu
caucugcaccaccggcaagcugcccgugcccuggcccacccucgugaccacccugaccuacggcgugcagugc
uucagccgcuaccccgaccacaugaagcagcacgacuucuucaaguccgccaugcccgaaggcuacguccagg
agcgcaccaucuucuucaaggacgacggcaacuacaagacccgcgccgaggugaaguucgagggcgacacccu
ggugaaccgcaucgagcugaagggcaucgacuucaaggaggacggcaacauccuggggcacaagcuggaguac
aacuacaacagccacaacgucuauaucauggccgacaagcagaagaacggcaucaaggugaacuucaagaucc
gccacaacaucgaggacggcagcgugcagcucgccgaccacuaccagcagaacacccccaucggcgacggcccc
gugcugcugcccgacaaccacuaccugagcacccaguccgcccugagcaaagaccccaacgagaagcgcgauc
acauggucugcuggaguucgugaccgccgccgggaucacucucggcauggacgagcuguacaagagaucuc
auaugcaucucgagugauagucuagaccuucugcggggcuugccuucuggccaugcccuucuucucucccuu
gcaccuguaccucuuggucuuuGAAUAAAGCCUGAGUAGGAAAAAAAAAAAAAAAAAAAAAA
AAAAAAAAAAAAAAAAAAAAAAAAAAAAAAAAAAAAAAAAAAAAAAAAAAAAAAAAAAA
AAAAAAAAAAAAAAAAAAAAAAAAAAAAAAAAAAAAAAAAGGCUAUUAUGCGUUACCG
GCGAGACGCUACGGACUUAAAUAAUUGAGCCUUAAAGAAGAAAUUCUUUAAGUGGAUGC

UCUCAAACUCAGGGAAACCUAAAUCUAGUUAUAGACAAGGCAAUCCUGAGCCAAGCCGA
AGUAGUAAUUAGUAAGACCAGUGGACAAUCGACGGAUAACAGCAUAUCUAGACACAGGA
AACAGCUAUGACCAUGAUUACGCCAAGCUUGCAUGCCUGCAGGUCGACUCUAGAGGAUC
CCCGGGUACCGAGCUCGAAUU

U1A responsive Circular EGFP variant5 (**bold: circularized**)

Group I intron, CVB3 IRES, EGFP, U1A binding motif

GGGAGACCCUCGACCGUCGAUUGUCCACUGGUCAACAAUAGAUGACUUACAACUAAUCG
GAAGGUGCAGAGACUCGACGGGAGCUACCCUAACGUCAAGACGAGGGUAAAGAGAGAG
UCCAAUUCUCAAAGCCAAUAGGCAGUAGCGAAAGCUGCAAGAGAAUG**AAAAUCCGUUGA
CCUUAAACGGUCGUGUGGGUUCAAGUCCCUCCACCCCCACGCCGGAAACGCAAUAGC
CGGCGAAUUAAGAGAGAAAAGAAGAGUAAGAAGAAAUAUAAGACACCGGUCGCCACC
UUAAAACAGCCUGUGGGUUGAUCCCACCCACAGGCCCAUUGGGCGCUAGCACUCUGG
UAUCACGGUACCUUUGUGCGCCUGUUUUAUACCCCCUCCCCCAACUGUAACUUAGAAG
UAACACACACCGAUCAACAGUCAGCGUGGCACACCAGCCACGUUUUGAUCAAGCACUU
CUGUUACCCCGGACUGAGUAUCAAUAGACUGCUCACGCGGUUGAAGGAGAAAGCGUU
CGUUAUCCGGCCAACUACUUCGAAAAACCUAGUAACACCGUGGAAGUUGCAGAGUGU
UUCGCUCAGCACUACCCCAGUGUAGAUCAGGUCGAUGAGUCACCGCAUUCCCCACGG
GCGACCGUGGCGGUGGCUGCGUUGGCGGCCUGCCCAUGGGGAAACCCAUGGGACGCU
CUAAUACAGACAUGGUGCGAAGAGUCUAUUGAGCUAGUUGGUAGUCCUCCGGCCCCU
GAAUGCGGCUAAUCCUAACUGCGGAGCACACACCCUCAAGCCAGAGGGCAGUGUGUC
GUAACGGGCAACUCUGCAGCGGAACCGACUACUUUGGGUGUCCGUGUUUCAUUUUAU
UCCUAUACUGGCUGCUUAUGGUGACAAUUGAGAGAUCGUUACCAUAUAGCUAUUGGA
UUGGCCAUCCGGUGACUAAUAGAGCUAUUAUAUAGACAGCAUUGUACCCAGAGUCUG
UCCCCAGACAUUGCACCUGGCGCUGUCUCCCUUUGUUGGGUUUAUACCACUUAGCUU
GAAAGAGGUUAAAACAUUACAAUUCAUUGUUAAGUUGAAUACAGCAAAaugggauccgug
agcaagggcgaggagcuguucaccgggguggugcccauccuggucgagcuggacggcgacguaaacggccac
aaguucagcguguccggcgagggcgagggcgaugccaccuacggcaagcugacccugaaguucaucugcacca
ccggcaagcugcccgugcccuggcccacccucgugaccacccugaccuacggcgugcagugcuucagccgcua
ccccgaccacaugaagcagcacgacuucuucaaguccgccaugcccgaaggcuacguccaggagcgcaccauc
uucuucaaggacgacggcaacuacaagacccgcgccgaggugaaguucgagggcgacacccuggugaaccgca
ucgagcugaagggcaucgacuucaaggaggacggcaacauccuggggcacaagcuggaguacaacuacaacag
ccacaacgucuauaucauggccgacaagcagaagaacggcaucaaggugaacuucaagauccgccacaacauc
gaggacggcagcgugcagcucgccgaccacuaccagcagaacacccccaucggcgacggccccgugcugcugc
ccgacaaccacuaccugagcacccaguccgcccugagcaaagaccccaacgagaagcgcgaucacauggu ccug
cuggaguucgugaccgccgccgggaucacucucggcauggacgagcuguacaagagaucucauaugcaucuc**

gagugauagucuagaccuucugcggggcuugccuucuggccaugcccuucuucucucccuugcaccuguacc
ucuuggucuuuGAAUAAAGCCUGAGUAGGGGCUAUUAUGCGUUACCGGCGAGACGCUAC
GGACUUAAAUAAUUGAGCCUUAAAGAAGAAAUUCUUUAAGUGGAUGCUCUCAAACUCAG
GGAAACCUAAAUCUAGUUAUAGACAAGGCAAUCCUGAGCCAAGCCGAAGUAGUAAUUAG
UAAGACCAGUGGACAAUCGACGGAUAACAGCAUAUCUAGACACAGGAAACAGCUAUGAC
CAUGAUUACGCCAAGCUUGCAUGCCUGCAGGUCGACUCUAGAGGAUCCCCGGGUACCG
AGCUCGAAUU

U1A responsive Circular EGFP +pA variant5 (**bold: circularized**)
Group I intron, CVB3 IRES, EGFP, U1A binding motif
GGGAGACCCUCGACCGUCGAUUGUCCACUGGUCAACAAUAGAUGACUUACAACUAAUCG
GAAGGUGCAGAGACUCGACGGGAGCUACCCUAACGUCAAGACGAGGGUAAAGAGAGAG
UCCAAUUCUCAAAGCCAAUAGGCAGUAGCGAAAGCUGCAAGAGAAUG**AAAAUCCGUUGA**
CCUUAAACGGUCGUGUGGGUUCAAGUCCCUCCACCCCCACGCCGGAAACGCAAUAGC
CGGCGAAUUAAGAGAGAAAAGAAGAGUAAGAAGAAAUAUAAGACACCGGUCGCCACC
UUAAAACAGCCUGUGGGUUGAUCCCACCCACAGGCCCAUUGGGCGCUAGCACUCUGG
UAUCACGGUACCUUUGUGCGCCUGUUUUAUACCCCCUCCCCCAACUGUAACUUAGAAG
UAACACACACCGAUCAACAGUCAGCGUGGCACACCAGCCACGUUUUGAUCAAGCACUU
CUGUUACCCCGGACUGAGUAUCAAUAGACUGCUCACGCGGUUGAAGGAGAAAGCGUU
CGUUAUCCGGCCAACUACUUCGAAAAACCUAGUAACACCGUGGAAGUUGCAGAGUGU
UUCGCUCAGCACUACCCCAGUGUAGAUCAGGUCGAUGAGUCACCGCAUUCCCCACGG
GCGACCGUGGCGGUGGCUGCGUUGGCGGCCUGCCCAUGGGGAAACCCAUGGGACGCU
CUAAUACAGACAUGGUGCGAAGAGUCUAUUGAGCUAGUUGGUAGUCCUCCGGCCCCU
GAAUGCGGCUAAUCCUAACUGCGGAGCACACACCCUCAAGCCAGAGGGCAGUGUGUC
GUAACGGGCAACUCUGCAGCGGAACCGACUACUUUGGGUGUCCGUGUUUCAUUUUAU
UCCUAUACUGGCUGCUUAUGGUGACAAUUGAGAGAUCGUUACCAUAUAGCUAUUGGA
UUGGCCAUCCGGUGACUAAUAGAGCUAUUAUAUAGACAGCAUUGUACCCAGAGUCUG
UCCCCAGACAUUGCACCUGGCGCUGUCUCCCUUUGUUGGGUUUAUACCACUUAGCUU
GAAAGAGGUUAAAACAUUACAAUUCAUUGUUAAGUUGAAUACAGCAAAaugggauccgug
agcaagggcgaggagcuguucaccgggguggugcccauccuggucgagcuggacggcgacguaaacggccac
aaguucagcguguccggcgagggcgagggcgaugccaccuacggcaagcugacccugaaguucaucugcacca
ccggcaagcugcccgugcccuggcccacccucgugaccacccugaccuacggcgugcagugcuucagccgcua
ccccgaccacaugaagcagcacgacuucuucaaguccgccaugcccgaaggcuacguccaggagcgcaccauc
uucuucaaggacgacggcaacuacaagacccgcgccgaggugaaguucgagggcgacacccuggugaaccgca
ucgagcugaagggcaucgacuucaaggaggacggcaacauccuggggcacaagcuggaguacaacuacaacag
ccacaacgucuauaucauggccgacaagcagaagaacggcaucaaggugaacuucaagauccgccacaacauc

gaggacggcagcgugcagcucgccgaccacuaccagcagaacacccccaucggcgacggccccgugcugcugc ccgacaaccacuaccugagcacccaguccgcccugagcaaagaccccaacgagaagcgcgaucacauggucug cuggaguucgugaccgccgccgggaucacucucggcauggacgagcuguacaagagaucucauaugcaucuc gagugauagucuagaccuucugcggggcuugccuucuggccaugcccuucuucucucccuugcaccuguacc ucuuggucuuuGAAUAAAGCCUGAGUAGGAAAAAAAAAAAAAAAAAAAAAAAAAAAAAAA AAAAAAAAAAAAAAAAAAAAAAAAAAAAAAAAAAAAAAAAAAAAAAAAAAAAAAAAAA AAAAAAAAAAAAAAAAAAAAAAAAAAAAAAAGGCUAUUAUGCGUUACCGGCGAGACGC UACGGACUUAAAUAAUUGAGCCUUAAAGAAGAAAUUCUUUAAGUGGAUGCUCUCAAACU CAGGGAAACCUAAAUCUAGUUAUAGACAAGGCAAUCCUGAGCCAAGCCGAAGUAGUAAU UAGUAAGACCAGUGGACAAUCGACGGAUAACAGCAUAUCUAG

Cap-MS2CP

MS2CP

GGGCGAAUUAAGAGAGAAAAGAAGAGUAAGAAGAAAUAUAAGACACCGGUcgccaccaugGC UUCUAACUUUACUCAGUUCGUUCUCGUCGACAAUGGCGGAACUGGCGACGUGACUGUC GCCCCAAGCAACUUCGCUAACGGGGUCGCUGAAUGGAUCAGCUCUAACUCGCGAUCAC AGGCUUACAAAGUAACCUGUAGCGUUCGUCAGAGCUCUGCGCAGAAUCGCAAAUACACC AUCAAAGUCGAGGUGCCUAAAGGCGCAUGGAGGUCUUACUUAAAUAUGGAACUAACCAU UCCAAUUUUCGCCACGAAUUCCGACUGCGAGCUUAUUGUUAAGGCAAUGCAAGGUCUCC UAAAAGAUGGAAACCCGAUUCCCUCGGCCAUCGCGGCCAACUCCGGCAUCUACUGAucua gaccuucugcggggcuugccuucuggccaugcccuucuucucucccuugcaccuguaccucuuggucuuuGAAUAAA GCCUGAGUAGGAAAAAAAAAAAAAAAAAAAAAAAAAAAAAAAAAAAAAAAAAAAAAAAAAAA AAAAAAAAAAAAAAAAAAAAAAAAAAAAAAAAAAAAAAAAAAAAAAAAAAAAAAAAAAAAAAA AAAAAA

Cap-U1A

U1A

GGGCGAAUUAAGAGAGAAAAGAAGAGUAAGAAGAAAUAUAAGACACCGGUcgccaccAUGG cgGCAGUUCCCGAGACCCGCCCUAACCACACUAUUUAUAUCAACAACCUCAAUGAGAAGA UCAAGAAGGAUGAGCUAAAAAAGUCCCUGUACGCCAUCUUCUCCCAGUUUGGCCAGAUC CUGGAUAUCCUGGUAUCACGGAGCCUGAAGAUGAGGGGCCAGGCCUUUGUCAUCUUCA AGGAGGUCAGCAGCGCCACCAACGCCCUGCGCUCCAUGCAGGGUUUCCCUUUCUAUGA CAAACCUAUGCGUAUCCAGUAUGCCAAGACCGACUCAGAUAUCAUUGCCAAGAUGAAAG GCACCUUCGUGGAGCGGGACCGCAAGCGGGAGAAGAGGAAGCCCAAGAGCCAGGAGAC CCCGGCCACCAAGAAGGCUGUGCAAGGCGGGGGAGCCACCCCCGUGGUGGGGGCUGU CCAGGGGCCUGUCCCGGGCAUGCCGCCGAUGACUCAGGCGCCCCGCAUUAUGCACCAC

AUGCCGGGCCAGCCGCCCUACAUGCCGCCCCCUGGUAUGAUCCCCCGCCAGGCCUUG
CACCUGGCCAGAUCCCACCAGGGGCCAUGCCCCCGCAGCAGCUUAUGCCAGGACAGAU
GCCCCCUGCCCAGCCUCUUUCUGAGAAUCCACCGAAUCACAUCUUGUUCCUCACCAACC
UGCCAGAGGAGACCAACGAGCUCAUGCUGUCCAUGCUUUUCAAUCAGUUCCCUGGCUU
CAAGGAGGUCCGUCUGGUACCCGGGCGGCAUGACAUCGCCUUCGUGGAGUUUGACAAU
GAGGUACAGGCAGGGGCAGCUCGCGAUGCCCUGCAGGGCUUUAAGAUCACGCAGAACA
ACGCCAUGAAGAUCUCCUUUGCCAAGAAGUAGucuagaccuucugcggggcuugccuucuggccaugc
ccuucuucucucccuugcaccuguaccucuuggucuuuGAAUAAAGCCUGAGUAGGAAAAAAAAAAAAA
AAAAAAAAAAAAAAAAAAAAAAAAAAAAAAAAAAAAAAAAAAAAAAAAAAAAAAAAAAAAAAAAAAAAAA
AAAAAAAAAAAAAAAAAAAAAAAAAAAAAAAAAAAAAAAAAAAAAAAAA

3’T302a-5p Circular MS2CP +pA (**bold: circularized**)

Group I intron, CVB3 IRES, miR-302a-5p target site, MS2CP

GGGAGACCCUCGACCGUCGAUUGUCCACUGGUCAACAAUAGAUGACUUACAACUAAUCG
GAAGGUGCAGAGACUCGACGGGAGCUACCCUAACGUCAAGACGAGGGUAAAGAGAG
UCCAAUUCUCAAAGCCAAUAGGCAGUAGCGAAAGCUGCAAGAGAAUG**AAAAUCCGUUGA**
CCUUAAACGGUCGUGUGGGUUCAAGUCCCUCCACCCCCACGCCGGAAACGCAAUAGC
CGGCGAAUUAAGAGAGAAAAGAAGAGUAAGAAGAAAUAUAAGACACCGGUCGCCACC
UUAAAACAGCCUGUGGGUUGAUCCCACCCACAGGCCCAUUGGGCGCUAGCACUCUGG
UAUCACGGUACCUUUGUGCGCCUGUUUUAUACCCCCUCCCCCAACUGUAACUUAGAAG
UAACACACACCGAUCAACAGUCAGCGUGGCACACCAGCCACGUUUUGAUCAAGCACUU
CUGUUACCCCGGACUGAGUAUCAAUAGACUGCUCACGCGGUUGAAGGAGAAAGCGUU
CGUUAUCCGGCCAACUACUUCGAAAAACCUAGUAACACCGUGGAAGUUGCAGAGUGU
UUCGCUCAGCACUACCCCAGUGUAGAUCAGGUCGAUGAGUCACCGCAUUCCCCACGG
GCGACCGUGGCGGUGGCUGCGUUGGCGGCCUGCCCAUGGGGAAACCCAUGGGACGCU
CUAAUACAGACAUGGUGCGAAGAGUCUAUUGAGCUAGUUGGUAGUCCUCCGGCCCCU
GAAUGCGGCUAAUCCUAACUGCGGAGCACACACCCUCAAGCCAGAGGGCAGUGUGUC
GUAACGGGCAACUCUGCAGCGGAACCGACUACUUUGGGUGUCCGUGUUUCAUUUUAU
UCCUAUACUGGCUGCUUAUGGUGACAAUUGAGAGAUCGUUACCAUAUAGCUAUUGGA
UUGGCCAUCCGGUGACUAAUAGAGCUAUUAUAUCCCUUUGUUGGGUUUAUACCACU
UAGCUUGAAAGAGGUUAAAACAUUACAAUUCAUUGUUAAGUUGAAUACAGCAAAaugG
CUUCUAACUUUACUCAGUUCGUUCUCGUCGACAAUGGCGGAACUGGCGACGUGACUG
UCGCCCCAAGCAACUUCGCUAACGGGGUCGCUGAAUGGAUCAGCUCUAACUCGCGAU
CACAGGCUUACAAAGUAACCUGUAGCGUUCGUCAGAGCUCUGCGCAGAAUCGCAAAU
ACACCAUCAAAGUCGAGGUGCCUAAAGGCGCAUGGAGGUCUUACUUAAAUAUGGAAC

UAACCAUUCCAAUUUUCGCCACGAAUUCCGACUGCGAGCUUAUUGUUAAGGCAAUGCA
AGGUCUCCUAAAAGAUGGAAACCCGAUUCCCUCGGCCAUCGCGGCCAACUCCGGCAU
CUACUGAAGCAAGUACAUCCACGUUUAAGUucuagaccuucugcggggcuugccuucuggccau
gcccuucuucucucccuugcaccuguaccucuuggucuuuGAAUAAAGCCUGAGUAGGAAAAAAA
AAAAAAAAAAAAAAAAAAAAAAAAAAAAAAAAAAAAAAAAAAAAAAAAAAAAAAAAAA
AAAAAAAAAAAAAAAAAAAAAAAAAAAAAAAAAAAAAAAAAAAAAAAAAAAAAAAGGC
UAUUAUGCGUUACCGGCGAGACGCUACGGACUUAAAUAAUUGAGCCUUAAAGAAGAAA
UUCUUUAAGUGGAUGCUCUCAAACUCAGGGAAACCUAAAUCUAGUUAUAGACAAGGCAA
UCCUGAGCCAAGCCGAAGUAGUAAUUAGUAAGACCAGUGGACAAUCGACGGAUAACAGC
AUAUCUAGACACAGGAAACAGCUAUGACCAUGAUUACGCCAAGCUUGCAUGCCUGCAGG
UCGACUCUAGAGGAUC

3’T302a-5p Circular U1A +pA (**bold: circularized**)

Group I intron, CVB3 IRES, miR-302a-5p target site, U1A

GGGAGACCCUCGACCGUCGAUUGUCCACUGGUCAACAAUAGAUGACUUACAACUAAUCG
GAAGGUGCAGAGACUCGACGGGAGCUACCCUAACGUCAAGACGAGGGUAAAGAGAG
UCCAAUUCUCAAAGCCAAUAGGCAGUAGCGAAAGCUGCAAGAGAAUG**AAAAUCCGUUGA**
CCUUAAACGGUCGUGUGGGUUCAAGUCCCUCCACCCCCACGCCGGAAACGCAAUAGC
CGGCGAAUUAAGAGAGAAAAGAAGAGUAAGAAGAAAUAUAAGACACCGGUCGCCACC
UUAAAACAGCCUGUGGGUUGAUCCCACCCACAGGCCCAUUGGGCGCUAGCACUCUGG
UAUCACGGUACCUUUGUGCGCCUGUUUUAUACCCCCUCCCCCAACUGUAACUUAGAAG
UAACACACACCGAUCAACAGUCAGCGUGGCACACCAGCCACGUUUUGAUCAAGCACUU
CUGUUACCCCGGACUGAGUAUCAAUAGACUGCUCACGCGGUUGAAGGAGAAAGCGUU
CGUUAUCCGGCCAACUACUUCGAAAAACCUAGUAACACCGUGGAAGUUGCAGAGUGU
UUCGCUCAGCACUACCCCAGUGUAGAUCAGGUCGAUGAGUCACCGCAUUCCCCACGG
GCGACCGUGGCGGUGGCUGCGUUGGCGGCCUGCCCAUGGGGAAACCCAUGGGACGCU
CUAAUACAGACAUGGUGCGAAGAGUCUAUUGAGCUAGUUGGUAGUCCUCCGGCCCCU
GAAUGCGGCUAAUCCUAACUGCGGAGCACACACCCUCAAGCCAGAGGGCAGUGUGUC
GUAACGGGCAACUCUGCAGCGGAACCGACUACUUUGGGUGUCCGUGUUUCAUUUUAU
UCCUAUACUGGCUGCUUAUGGUGACAAUUGAGAGAUCGUUACCAUAUAGCUAUUGGA
UUGGCCAUCCGGUGACUAAUAGAGCUAUUAUAUCCCUUUGUUGGGUUUAUACCACU
UAGCUUGAAAGAGGUUAAAACAUUACAAUUCAUUGUUAAGUUGAAUACAGCAAAAUGG
cgGCAGUUCCCGAGACCCGCCCUAACCACACUAUUUAUAUCAACAACCUCAAUGAGAA
GAUCAAGAAGGAUGAGCUAAAAAAGUCCCUGUACGCCAUCUUCUCCCAGUUUGGCCA
GAUCCUGGAUAUCCUGGUAUCACGGAGCCUGAAGAUGAGGGGCCAGGCCUUUGUCAU
CUUCAAGGAGGUCAGCAGCGCCACCAACGCCCUGCGCUCCAUGCAGGGUUUCCCUUU

CUAUGACAAACCUAUGCGUAUCCAGUAUGCCAAGACCGACUCAGAUAUCAUUGCCAAG AUGAAAGGCACCUUCGUGGAGCGGGACCGCAAGCGGGAGAAGAGGAAGCCCAAGAGC CAGGAGACCCCGGCCACCAAGAAGGCUGUGCAAGGCGGGGGAGCCACCCCCGUGGUG GGGGCUGUCCAGGGGCCUGUCCCGGGCAUGCCGCCGAUGACUCAGGCGCCCCGCAUU AUGCACCACAUGCCGGGCCAGCCGCCCUACAUGCCGCCCCCUGGUAUGAUCCCCCCG CCAGGCCUUGCACCUGGCCAGAUCCCACCAGGGGCCAUGCCCCCGCAGCAGCUUAUG CCAGGACAGAUGCCCCCUGCCCAGCCUCUUUCUGAGAAUCCACCGAAUCACAUCUUGU UCCUCACCAACCUGCCAGAGGAGACCAACGAGCUCAUGCUGUCCAUGCUUUUCAAUCA GUUCCCUGGCUUCAAGGAGGUCCGUCUGGUACCCGGGCGGCAUGACAUCGCCUUCGU GGAGUUUGACAAUGAGGUACAGGCAGGGGCAGCUCGCGAUGCCCUGCAGGGCUUUAA GAUCACGCAGAACAACGCCAUGAAGAUCUCCUUUGCCAAGAAGUAGAGCAAGUACAU CCACGUUUAAGUucuagaccuucugcggggcuugccuucuggccaugcccuucuucucucccuugcacc uguaccucuuggucuuuGAAUAAAGCCUGAGUAGGAAAAAAAAAAAAAAAAAAAAAAAAAAAA AAAAAAAAAAAAAAAAAAAAAAAAAAAAAAAAAAAAAAAAAAAAAAAAAAAAAAAAAAAA AAAAAAAAAAAAAAAAAAAAAAAAAAAAAAAAAAAAAGGCUAUUAUGCGUUACCGGCGA GACGCUACGGACUUAAAUAAUUGAGCCUUAAAGAAGAAAUUCUUUAAGUGGAUGCUCUC AAACUCAGGGAAACCUAAAUCUAGUUAUAGACAAGGCAAUCCUGAGCCAAGCCGAAGUA GUAAUUAGUAAGACCAGUGGACAAUCGACGGAUAACAGCAUAUCUAGACACAGGAAACA GCUAUGACCAUGAUUACGCCAAGCUUGCAUGCCUGCAGGUCGACUCUAGAGGAUC

EGFP linear switch (MS2CP)

EGFP, MS2CP binding motif

GGUCAGAUCCGCUAGCGGAUCCGGGAGCAGGUGAGGAUCACCCAUCUGCCACGAGCGA GGUGAGGAUCACCCAUCUCGCUCGUGUUCCCACCGGUCgccaccaugggauccgugagcaagggc gaggagcuguucaccgggguggugcccauccuggucgagcuggacggcgacguaaacggccacaaguucagcguguccggcgagggcgagggcgaugccaccuacggcaagcugacccugaaguucaucugcaccaccggcaagcugcccgugcccu ggcccacccucgugaccacccugaccuacggcgugcagugcuucagccgcuaccccgaccacaugaagcagcacgacuu cuucaaguccgccaugcccgaaggcuacguccaggagcgcaccaucuucuucaaggacgacggcaacuacaagacccgc gccgaggugaaguucgagggcgacacccuggugaaccgcaucgagcugaagggcaucgacuucaaggaggacggcaac auccuggggcacaagcuggaguacaacuacaacagccacaacgucuauaucauggccgacaagcagaagaacggcauc aaggugaacuucaagauccgccacaacaucgaggacggcagcgugcagcucgccgaccacuaccagcagaacacccccaucggcgacggccccgugcugcugcccgacaaccacuaccugagcacccaguccgcccugagcaaagaccccaacgagaa gcgcgaucacaugguccugcuggaguucgugaccgccgccgggaucacucucggcauggacgagcuguacaagagauc ucauaugcaucucgagugauagucuagaccuucugcggggcuugccuucuggccaugcccuucuucucucccuugcacc uguaccucuuggucuuuGAAUAAAGCCUGAGUAGGAAAAAAAAAAAAAAAAAAAAAAAAAAAAA AAAAAAAAAAAAAAAAAAAAAAAAAAAAAAAAAAAAAAAAAAAAAAAAAAAAAAAAAAAAA

AAAAAAAAAAAAAAAAAAAAAAAAAAA

EGFP linear switch (U1A)

EGFP, U1A binding motif

GACAGCAUUGUACCCAGAGUCUGUCCCCAGACAUUGCACCUGGCGCUGUCCGCAGAUC
GAGAAGAAGGCGAAUUAAGAGAGAAAAGAAGAGUAAGAAGAAAUAUAAGACACCGGUgcc
accaugggauccgugagcaagggcgaggagcuguucaccgggguggugcccauccuggucgagcuggacggcgacgua
aacggccacaaguucagcguguccggcgagggcgagggcgaugccaccuacggcaagcugacccugaaguucaucugc
accaccggcaagcugcccgugcccuggcccacccucgugaccacccugaccuacggcgugcagugcuucagccgcuaccc
cgaccacaugaagcagcacgacuucuucaaguccgccaugcccgaaggcuacguccaggagcgcaccaucuucuucaag
gacgacggcaacuacaagacccgcgccgaggugaaguucgagggcgacacccuggugaaccgcaucgagcugaagggc
aucgacuucaaggaggacggcaacauccuggggcacaagcuggaguacaacuacaacagccacaacgucuauaucaug
gccgacaagcagaagaacggcaucaaggugaacuucaagauccgccacaacaucgaggacggcagcgugcagcucgcc
gaccacuaccagcagaacacccccaucggcgacggccccgugcugcugcccgacaaccacuaccugagcacccaguccgc
ccugagcaaagaccccaacgagaagcgcgaucacaugguccugcuggaguucgugaccgccgccgggaucacucucggc
auggacgagcuguacaagagaucucauaugcaucucgagugauagucuagaccuucugcggggcuugccuucuggcca
ugcccuucuucucucccuugcaccuguaccucuuggucuuuGAAUAAAGCCUGAGUAGGAAAAAAAAAAA
AAAAAAAAAAAAAAAAAAAAAAAAAAAAAAAAAAAAAAAAAAAAAAAAAAAAAAAAAAAAAAA
AAAAAAAAAAAAAAAAAAAAAAAAAAAAAAAAAAAAAAAAAAAAAA

MS2CP responsive Circular MetLuc2 +pA variant4 (**bold: circularized**)

Group I intron, CVB3 IRES, MetLuc2, MS2CP binding motif

GGGAGACCCUCGACCGUCGAUUGUCCACUGGUCAACAAUAGAUGACUUACAACUAAUCG
GAAGGUGCAGAGACUCGACGGGAGCUACCCUAACGUCAAGACGAGGGUAAAGAGAGAG
UCCAAUUCUCAAAGCCAAUAGGCAGUAGCGAAAGCUGCAAGAGAAUG**AAAAUCCGUUGA
CCUUAAACGGUCGUGUGGGUUCAAGUCCCUCCACCCCCACGCCGGAAACGCAAUAGC
CGGCGAAUUAAGAGAGAAAAGAAGAGUAAGAAGAAAUAUAAGACACCGGUCGCCACC
UUAAAACAGCCUGUGGGUUGAUCCCACCCACAGGCCCAUUGGGCGCUAGCACUCUGG
UAUCACGGUACCUUUGUGCGCCUGUUUUAUACCCCCUCCCCCAACUGUAACUUAGAAG
UAACACACACCGAUCAACAGUCAGCGUGGCACACCAGCCACGUUUUGAUCAAGCACUU
CUGUUACCCCGGACUGAGUAUCAAUAGACUGCUCACGCGGUUGAAGGAGAAAGCGUU
CGUUAUCCGGCCAACUACUUCGAAAAACCUAGUAACACCGUGGAAGUUGCAGAGUGU
UUCGCUCAGCACUACCCCAGUGUAGAUCAGGUCGAUGAGUCACCGCAUUCCCCACGG
GCGACCGUGGCGGUGGCUGCGUUGGCGGCCUGCCCAUGGGGAAACCCAUGGGACGCU
CUAAUACAGACAUGGUGCGAAGAGUCUAUUGAGCUAGUUGGUAGUCCUCCGGCCCCU
GAAUGCGGCUAAUCCUAACUGCGGAGCACACACCCUCAAGCCAGAGGGCAGUGUGUC**

GUAACGGGCAACUCUGCAGCGGAACCGACUACUUUGGGUGUCCGUGUUUCAUUUUAU
UCCUAUACUGGCUGCUUAUGGUGACAAUUGAGCAGGUGAGGAUCACCCAUCUGCCAC
GAGCGAGGUGAGGAUCACCCAUCUCGCUCGUGUUCGAUCGUUACCAUAUAGCUAUUG
GAUUGGCCAUCCGGUGACUAAUAGAGCUAUUAUAUCCCUUUGUUGGGUUUAUACC
ACUUAGCUUGAAAGAGGUUAAAACAUUACAAUUCAUUGUUAAGUUGAAUACAGCAAAa
uggacaucaagguggguucacccugguguucagcgcccuggugcaggccaagagcaccgaguucgacccca
acaucgacaucgugggccuggaaggcaaguucggcaucaccaaccuggaaaccgaccuguucaccaucuggga
gaccauggaagugaugaucaaggccgacaucgccgacaccgaccgggccagcaacuucguggccaccgagacc
gacgccaaccggggcaagaugcccggcaagaagcugccccuggccgucaucauggaaauggaagccaacgccu
ucaaggccggcugcacccggggcugccugaucugccugagcaagaucaagugcaccgccaagaugaaggugua
cauccccggcaggugccacgacuacggcggcgacaagaaaaccggccaggccggcaucgugggcgccaucgug
gacauccccgagaucagcggcuucaaagaaauggcccccauggaacaguucaucgcccagguggacagaugcg
ccagcugcaccaccggcugccugaagggccuggccaacgugaagugcagcgagcugcugaagaaguggcugcc
cgaccgcugcgccagcuucgccgacaagauccagaaagaggugcacaacaucaagggcauggccggcgacagg
ugaucuagaccuucugcggggcuugccuucuggccaugcccuucuucucucccuugcaccuguaccucuugg
ucuuuGAAUAAAGCCUGAGUAGGAAAAAAAAAAAAAAAAAAAAAAAAAAAAAAAAAAAA
AAAAAAAAAAAAAAAAAAAAAAAAAAAAAAAAAAAAAAAAAAAAAAAAAAAAAAAAAA
AAAAAAAAAAAAAAAAAAAAAAAAAAGGCUAUUAUGCGUUACCGGCGAGACGCUACGG
ACUUAAAUAAUUGAGCCUUAAAGAAGAAAUUCUUUAAGUGGAUGCUCUCAAACUCAGGG
AAACCUAAAUCUAGUUAUAGACAAGGCAAUCCUGAGCCAAGCCGAAGUAGUAAUUAGUA
AGACCAGUGGACAAUCGACGGAUAACAGCAUAUCUAGACACAGGAAACAGCUAUGACCA
UGAUUACGCCAAGCUUGCAUGCCUGCAGGUCGACUCUAGAGGAUCCCCGGGUACCGAG
CUCGAAUU

U1A responsive Circular MetLuc2 +pA variant5 (**bold: circularized**)

Group I intron, CVB3 IRES, MetLuc2, U1A binding motif

GGGAGACCCUCGACCGUCGAUUGUCCACUGGUCAACAAUAGAUGACUUACAACUAAUCG
GAAGGUGCAGAGACUCGACGGGAGCUACCCUAACGUCAAGACGAGGGUAAAGAGAGAG
UCCAAUUCUCAAAGCCAAUAGGCAGUAGCGAAAGCUGCAAGAGAAUG**AAAAUCCGUUGA**
CCUUAAACGGUCGUGUGGGUUCAAGUCCCUCCACCCCCACGCCGGAAACGCAAUAGC
CGGCGAAUUAAGAGAGAAAAGAAGAGUAAGAAGAAAUAUAAGACACCGGUCGCCACC
UUAAAACAGCCUGUGGGUUGAUCCCACCCACAGGCCCAUUGGGCGCUAGCACUCUGG
UAUCACGGUACCUUUGUGCGCCUGUUUUAUACCCCCUCCCCCAACUGUAACUUAGAAG
UAACACACACCGAUCAACAGUCAGCGUGGCACACCAGCCACGUUUUGAUCAAGCACUU
CUGUUACCCCGGACUGAGUAUCAAUAGACUGCUCACGCGGUUGAAGGAGAAAGCGUU
CGUUAUCCGGCCAACUACUUCGAAAAACCUAGUAACACCGUGGAAGUUGCAGAGUGU

UUCGCUCAGCACUACCCCAGUGUAGAUCAGGUCGAUGAGUCACCGCAUUCCCCACGG
GCGACCGUGGCGGUGGCUGCGUUGGCGGCCUGCCCAUGGGGAAACCCAUGGGACGCU
CUAAUACAGACAUGGUGCGAAGAGUCUAUUGAGCUAGUUGGUAGUCCUCCGGCCCCU
GAAUGCGGCUAAUCCUAACUGCGGAGCACACACCCUCAAGCCAGAGGGCAGUGUGUC
GUAACGGGCAACUCUGCAGCGGAACCGACUACUUUGGGUGUCCGUGUUUCAUUUUAU
UCCUAUACUGGCUGCUUAUGGUGACAAUUGAGAGAUCGUUACCAUAUAGCUAUUGGA
UUGGCCAUCCGGUGACUAAUAGAGCUAUUAUAUAGACAGCAUUGUACCCAGAGUCUG
UCCCCAGACAUUGCACCUGGCGCUGUCUCCCUUUGUUGGGUUUAUACCACUUAGCUU
GAAAGAGGUUAAAACAUUACAAUUCAUUGUUAAGUUGAAUACAGCAAAauggacaucaag
guggguguucacccugguguucagcgcccuggugcaggccaagagcaccgaguucgaccccaacaucgacaucg
ugggccuggaaggcaaguucggcaucaccaaccuggaaaccgaccuguucaccaucugggagaccauggaagu
gaugaucaaggccgacaucgccgacaccgaccgggccagcaacuucguggccaccgagaccgacgccaaccgg
ggcaagaugcccggcaagaagcugccccuggccgucaucauggaaauggaagccaacgccuucaaggccggcu
gcacccggggcugccugaucugccugagcaagaucaagugcaccgccaagaugaagguguacaucccccggcag
gugccacgacuacggcggcgacaagaaaaccggccaggccggcaucgugggcgccaucguggacauccccgag
aucagcggcuucaaagaaauggcccccauggaacaguucaucgcccagguggacagaugcgccagcugcacca
ccggcugccugaagggccuggccaacgugaagugcagcgagcugcugaagaaguggcugcccgaccgcugcg
ccagcuucgccgacaagauccagaaagaggugcacaacaucaagggcauggccggcgacaggugaucuagacc
uucugcggggcuugccuucuggccaugcccuucuucucucccuugcaccuguaccucuuggucuuuGAAUA
AAGCCUGAGUAGGAAAAAAAAAAAAAAAAAAAAAAAAAAAAAAAAAAAAAAAAAAAAAAAAA
AAAAAAAAAAAAAAAAAAAAAAAAAAAAAAAAAAAAAAAAAAAAAAAAAAAAAAAAAAAAAA
AAAAAAAAAAAAAAAAAGGCUAUUAUGCGUUACCGGCGAGACGCUACGGACUUAAAUA
AUUGAGCCUUAAAGAAGAAAUUCUUUAAGUGGAUGCUCUCAAACUCAGGGAAACCUAAA
UCUAGUUAUAGACAAGGCAAUCCUGAGCCAAGCCGAAGUAGUAAUUAGUAAGACCAGUG
GACAAUCGACGGAUAACAGCAUAUCUAGACACAGGAAACAGCUAUGACCAUGAUUACGC
CAAGCUUGCAUGCCUGCAGGUCGACUCUAGAGGAUCCCCGGGUACCGAGCUCGAAUU

3'T21-5p Circular MS2CP +pA (**bold: circularized**)

Group I intron, CVB3 IRES, miR-21-5p target site, MS2CP

GGGAGACCCUCGACCGUCGAUUGUCCACUGGUCAACAAUAGAUGACUUACAACUAAUCG
GAAGGUGCAGAGACUCGACGGGAGCUACCCUAACGUCAAGACGAGGGUAAAGAGAG
UCCAAUUCUCAAAGCCAAUAGGCAGUAGCGAAAGCUGCAAGAGAAUG**AAAAUCCGUUGA**
CCUUAAACGGUCGUGUGGGUUCAAGUCCCUCCACCCCCACGCCGGAAACGCAAUAGC
CGGCGAAUUAAGAGAGAAAAGAAGAGUAAGAAGAAAUAUAAGACACCGGUCGCCACC
UUAAAACAGCCUGUGGGUUGAUCCCACCCACAGGCCCAUUGGGCGCUAGCACUCUGG
UAUCACGGUACCUUUGUGCGCCUGUUUUAUACCCCCUCCCCCAACUGUAACUUAGAAG

UAACACACACCGAUCAACAGUCAGCGUGGCACACCAGCCACGUUUGAUCAAGCACUU
CUGUUACCCCGGACUGAGUAUCAAUAGACUGCUCACGCGGUUGAAGGAGAAAGCGUU
CGUUAUCCGGCCAACUACUUCGAAAAACCUAGUAACACCGUGGAAGUUGCAGAGUGU
UUCGCUCAGCACUACCCCAGUGUAGAUCAGGUCGAUGAGUCACCGCAUUCCCCACGG
GCGACCGUGGCGGUGGCUGCGUUGGCGGCCUGCCCAUGGGGAAACCCAUGGGACGCU
CUAAUACAGACAUGGUGCGAAGAGUCUAUUGAGCUAGUUGGUAGUCCUCCGGCCCCU
GAAUGCGGCUAAUCCUAACUGCGGAGCACACACCCUCAAGCCAGAGGGCAGUGUGUC
GUAACGGGCAACUCUGCAGCGGAACCGACUACUUUGGGUGUCCGUGUUUCAUUUUAU
UCCUAUACUGGCUGCUUAUGGUGACAAUUGAGAGAUCGUUACCAUAUAGCUAUUGGA
UUGGCCAUCCGGUGACUAAUAGAGCUAUUAUAUCCCUUUGUUGGGUUUAUACCACU
UAGCUUGAAAGAGGUUAAAACAUUACAAUUCAUUGUUAAGUUGAAUACAGCAAAaugG
CUUCUAACUUUACUCAGUUCGUUCUCGUCGACAAUGGCGGAACUGGCGACGUGACUG
UCGCCCCAAGCAACUUCGCUAACGGGGUCGCUGAAUGGAUCAGCUCUAACUCGCGAU
CACAGGCUUACAAAGUAACCUGUAGCGUUCGUCAGAGCUCUGCGCAGAAUCGCAAAU
ACACCAUCAAAGUCGAGGUGCCUAAAGGCGCAUGGAGGUCUUACUUAAAUAUGGAAC
UAACCAUUCCAAUUUUCGCCACGAAUUCCGACUGCGAGCUUAUUGUUAAGGCAAUGCA
AGGUCUCCUAAAAGAUGGAAACCCGAUUCCCUCGGCCAUCGCGGCCAACUCCGGCAU
CUACUGAUCAACAUCAGUCUGAUAAGCUAucuagaccuucugcggggcuugccuucuggccaugc
ccuucuucucucccuugcaccuguaccucuuggucuuuGAAUAAAGCCUGAGUAGGAAAAAAAAA
AAAAAAAAAAAAAAAAAAAAAAAAAAAAAAAAAAAAAAAAAAAAAAAAAAAAAAAAAA
AAAAAAAAAAAAAAAAAAAAAAAAAAAAAAAAAAAAAAAAAAAAAAAAAAAAAGGCUA
UUAUGCGUUACCGGCGAGACGCUACGGACUUAAAUAAUUGAGCCUUAAAGAAGAAAUU
CUUUAAGUGGAUGCUCUCAAACUCAGGGAAACCUAAAUCUAGUUAUAGACAAGGCAAUC
CUGAGCCAAGCCGAAGUAGUAAUUAGUAAGACCAGUGGACAAUCGACGGAUAACAGCAU
AUCUAGACACAGGAAACAGCUAUGACCAUGAUUACGCCAAGCUUGCAUGCCUGCAGGUC
GACUCUAGAGGAUC

3’T21-5p Circular U1A +pA (**bold: circularized**)

Group I intron, CVB3 IRES, miR-21-5p target site, U1A

GGGAGACCCUCGACCGUCGAUUGUCCACUGGUCAACAAUAGAUGACUUACAACUAAUCG
GAAGGUGCAGAGACUCGACGGGAGCUACCCUAACGUCAAGACGAGGGUAAAGAGAG
UCCAAUUCUCAAAGCCAAUAGGCAGUAGCGAAAGCUGCAAGAGAAUG**AAAAUCCGUUGA**
CCUUAAACGGUCGUGUGGGUUCAAGUCCCUCCACCCCCACGCCGGAAACGCAAUAGC
CGGCGAAUUAAGAGAGAAAAGAAGAGUAAGAAGAAAUAUAAGACACCGGUCGCCACC
UUAAAACAGCCUGUGGGUUGAUCCCACCCACAGGCCCAUUGGGCGCUAGCACUCUGG
UAUCACGGUACCUUUGUGCGCCUGUUUUAUACCCCCUCCCCCAACUGUAACUUAGAAG

UAACACACACCGAUCAACAGUCAGCGUGGCACACCAGCCACGUUUUGAUCAAGCACUU
CUGUUACCCCGGACUGAGUAUCAAUAGACUGCUCACGCGGUUGAAGGAGAAAGCGUU
CGUUAUCCGGCCAACUACUUCGAAAAACCUAGUAACACCGUGGAAGUUGCAGAGUGU
UUCGCUCAGCACUACCCCAGUGUAGAUCAGGUCGAUGAGUCACCGCAUUCCCCACGG
GCGACCGUGGCGGUGGCUGCGUUGGCGGCCUGCCCAUGGGGAAACCCAUGGGACGCU
CUAAUACAGACAUGGUGCGAAGAGUCUAUUGAGCUAGUUGGUAGUCCUCCGGCCCCU
GAAUGCGGCUAAUCCUAACUGCGGAGCACACACCCUCAAGCCAGAGGGCAGUGUGUC
GUAACGGGCAACUCUGCAGCGGAACCGACUACUUUGGGUGUCCGUGUUUCAUUUUAU
UCCUAUACUGGCUGCUUAUGGUGACAAUUGAGAGAUCGUUACCAUAUAGCUAUUGGA
UUGGCCAUCCGGUGACUAAUAGAGCUAUUAUAUCCCUUUGUUGGGUUUAUACCACU
UAGCUUGAAAGAGGUUAAAACAUUACAAUUCAUUGUUAAGUUGAAUACAGCAAAAUGG
cgGCAGUUCCCGAGACCCGCCCUAACCACACUAUUUAUAUCAACAACCUCAAUGAGAA
GAUCAAGAAGGAUGAGCUAAAAAAGUCCCUGUACGCCAUCUUCUCCCAGUUUGGCCA
GAUCCUGGAUAUCCUGGUAUCACGGAGCCUGAAGAUGAGGGGCCAGGCCUUUGUCAU
CUUCAAGGAGGUCAGCAGCGCCACCAACGCCCUGCGCUCCAUGCAGGGUUUCCCUUU
CUAUGACAAACCUAUGCGUAUCCAGUAUGCCAAGACCGACUCAGAUAUCAUUGCCAAG
AUGAAAGGCACCUUCGUGGAGCGGGACCGCAAGCGGGAGAAGAGGAAGCCCAAGAGC
CAGGAGACCCCGGCCACCAAGAAGGCUGUGCAAGGCGGGGGAGCCACCCCCGUGGUG
GGGGCUGUCCAGGGGCCUGUCCCGGGCAUGCCGCCGAUGACUCAGGCGCCCCGCAUU
AUGCACCACAUGCCGGGCCAGCCGCCCUACAUGCCGCCCCCUGGUAUGAUCCCCCCG
CCAGGCCUUGCACCUGGCCAGAUCCCACCAGGGGCCAUGCCCCCGCAGCAGCUUAUG
CCAGGACAGAUGCCCCCUGCCCAGCCUCUUUCUGAGAAUCCACCGAAUCACAUCUUGU
UCCUCACCAACCUGCCAGAGGAGACCAACGAGCUCAUGCUGUCCAUGCUUUUCAAUCA
GUUCCCUGGCUUCAAGGAGGUCCGUCUGGUACCCGGGCGGCAUGACAUCGCCUUCGU
GGAGUUUGACAAUGAGGUACAGGCAGGGGCAGCUCGCGAUGCCCUGCAGGGCUUUAA
GAUCACGCAGAACAACGCCAUGAAGAUCUCCUUUGCCAAGAAGUAGUCAACAUCAGUC
UGAUAAGCUAucuagaccuucugcggggcuugccuucuggccaugcccuucuucucucccuugcaccugu
accucuuggucuuuGAAUAAAGCCUGAGUAGGAAAAAAAAAAAAAAAAAAAAAAAAAAA
AAAAAAAAAAAAAAAAAAAAAAAAAAAAAAAAAAAAAAAAAAAAAAAAAAAAAAAA
AAAAAAAAAAAAAAAAAAAAAAAAAAAAAAAAAAGGCUAUUAUGCGUUACCGGCGAGA
CGCUACGGACUUAAAUAAUUGAGCCUUAAAGAAGAAAUUCUUUAAGUGGAUGCUCUCAA
ACUCAGGGAAACCUAAAUCUAGUUAUAGACAAGGCAAUCCUGAGCCAAGCCGAAGUAGU
AAUUAGUAAGACCAGUGGACAAUCGACGGAUAACAGCAUAUCUAGACACAGGAAACAGC
UAUGACCAUGAUUACGCCAAGCUUGCAUGCCUGCAGGUCGACUCUAGAGGAUC

5’T302a-5p MS2CP

MS2CP, miR-302a-5p target site

GGGCGAAUUAAGAGAGAAAAGAAGAGUAAGAAGAAAUAUAAGACACCGGUcAGCAAGUAC
AUCCACGUUUAAGUgccaccaugGCUUCUAACUUUACUCAGUUCGUUCUCGUCGACAAUGG
CGGAACUGGCGACGUGACUGUCGCCCCAAGCAACUUCGCUAACGGGGUCGCUGAAUGG
AUCAGCUCUAACUCGCGAUCACAGGCUUACAAAGUAACCUGUAGCGUUCGUCAGAGCUC
UGCGCAGAAUCGCAAAUACACCAUCAAAGUCGAGGUGCCUAAAGGCGCAUGGAGGUCUU
ACUUAAAUAUGGAACUAACCAUUCCAAUUUUCGCCACGAAUUCCGACUGCGAGCUUAUU
GUUAAGGCAAUGCAAGGUCUCCUAAAAGAUGGAAACCCGAUUCCCUCGGCCAUCGCGG
CCAACUCCGGCAUCUACUGAucuagaccuucugcggggcuugccuucuggccaugcccuucuucucucccuu
gcaccuguaccucuuggucuuuGAAUAAAGCCUGAGUAGGAAAAAAAAAAAAAAAAAAAAAAAAAA
AAAAAAAAAAAAAAAAAAAAAAAAAAAAAAAAAAAAAAAAAAAAAAAAAAAAAAAAAAAAAAA
AAAAAAAAAAAAAAAAAAAAAAAAAAAAAAA

5’T302a-5p U1A

U1A, miR-302a-5p target site

GGGCGAAUUAAGAGAGAAAAGAAGAGUAAGAAGAAAUAUAAGACACCGGUcAGCAAGUAC
AUCCACGUUUAAGUgccaccAUGGcgGCAGUUCCCGAGACCCGCCCUAACCACACUAUUUA
UAUCAACAACCUCAAUGAGAAGAUCAAGAAGGAUGAGCUAAAAAGUCCCUGUACGCCA
UCUUCUCCCAGUUUGGCCAGAUCCUGGAUAUCCUGGUAUCACGGAGCCUGAAGAUGAG
GGGCCAGGCCUUUGUCAUCUUCAAGGAGGUCAGCAGCGCCACCAACGCCCUGCGCUCC
AUGCAGGGUUUCCCUUUCUAUGACAAACCUAUGCGUAUCCAGUAUGCCAAGACCGACUC
AGAUAUCAUUGCCAAGAUGAAAGGCACCUUCGUGGAGCGGGACCGCAAGCGGGAGAAG
AGGAAGCCCAAGAGCCAGGAGACCCCGGCCACCAAGAAGGCUGUGCAAGGCGGGGGAG
CCACCCCCGUGGUGGGGGCUGUCCAGGGGCCUGUCCCGGGCAUGCCGCCGAUGACUC
AGGCGCCCCGCAUUAUGCACCACAUGCCGGGCCAGCCGCCCUACAUGCCGCCCCCUGG
UAUGAUCCCCCCGCCAGGCCUUGCACCUGGCCAGAUCCCACCAGGGGCCAUGCCCCCG
CAGCAGCUUAUGCCAGGACAGAUGCCCCCUGCCCAGCCUCUUUCUGAGAAUCCACCGAA
UCACAUCUUGUUCCUCACCAACCUGCCAGAGGAGACCAACGAGCUCAUGCUGUCCAUGC
UUUUCAAUCAGUUCCCUGGCUUCAAGGAGGUCCGUCUGGUACCCGGGCGGCAUGACAU
CGCCUUCGUGGAGUUUGACAAUGAGGUACAGGCAGGGGCAGCUCGCGAUGCCCUGCAG
GGCUUUAAGAUCACGCAGAACAACGCCAUGAAGAUCUCCUUUGCCAAGAAGUAGucuagac
cuucugcggggcuugccuucuggccaugcccuucuucucucccuugcaccuguaccucuuggucuuuGAAUAAAGC
CUGAGUAGGAAAAAAAAAAAAAAAAAAAAAAAAAAAAAAAAAAAAAAAAAAAAAAAAAAAAA
AAAAAAAAAAAAAAAAAAAAAAAAAAAAAAAAAAAAAAAAAAAAAAAAAAAAAAAAAAAAAAA
AAAA

5’T21-5p MS2CP

MS2CP, miR-21-5p target site

GGGCGAAUUAAGAGAGAAAAGAAGAGUAAGAAGAAAUAUAAGACACCGGUcUCAACAUCA
GUCUGAUAAGCUAgccaccaugGCUUCUAACUUUACUCAGUUCGUUCUCGUCGACAAUGGC
GGAACUGGCGACGUGACUGUCGCCCCAAGCAACUUCGCUAACGGGGUCGCUGAAUGGA
UCAGCUCUAACUCGCGAUCACAGGCUUACAAAGUAACCUGUAGCGUUCGUCAGAGCUCU
GCGCAGAAUCGCAAAUACACCAUCAAAGUCGAGGUGCCUAAAGGCGCAUGGAGGUCUUA
CUUAAAUAUGGAACUAACCAUUCCAAUUUUCGCCACGAAUUCCGACUGCGAGCUUAUUG
UUAAGGCAAUGCAAGGUCUCCUAAAAGAUGGAAACCCGAUUCCCUCGGCCAUCGCGGCC
AACUCCGGCAUCUACUGAucuagaccuucugcggggcuugccuucuggccaugcccuucuucucucccuugca
ccuguaccucuuggucuuuGAAUAAAGCCUGAGUAGGAAAAAAAAAAAAAAAAAAAAAAAAAAAAA
AAAAAAAAAAAAAAAAAAAAAAAAAAAAAAAAAAAAAAAAAAAAAAAAAAAAAAAAAAAAAAAA
AAAAAAAAAAAAAAAAAAAAAAAAAAAAA

5’T21-5p U1A

U1A, miR-21-5p target site

GGGCGAAUUAAGAGAGAAAAGAAGAGUAAGAAGAAAUAUAAGACACCGGUcUCAACAUCA
GUCUGAUAAGCUAgccaccAUGGcgGCAGUUCCCGAGACCCGCCCUAACCACACUAUUUAU
AUCAACAACCUCAAUGAGAAGAUCAAGAAGGAUGAGCUAAAAAAGUCCCUGUACGCCAU
CUUCUCCCAGUUUGGCCAGAUCCUGGAUAUCCUGGUAUCACGGAGCCUGAAGAUGAGG
GGCCAGGCCUUUGUCAUCUUCAAGGAGGUCAGCAGCGCCACCAACGCCCUGCGCUCCA
UGCAGGGUUUCCCUUUCUAUGACAAACCUAUGCGUAUCCAGUAUGCCAAGACCGACUCA
GAUAUCAUUGCCAAGAUGAAAGGCACCUUCGUGGAGCGGGACCGCAAGCGGGAGAAGA
GGAAGCCCAAGAGCCAGGAGACCCCGGCCACCAAGAAGGCUGUGCAAGGCGGGGGAGC
CACCCCCGUGGUGGGGGCUGUCCAGGGGCCUGUCCCGGGCAUGCCGCCGAUGACUCA
GGCGCCCCGCAUUAUGCACCACAUGCCGGGCCAGCCGCCCUACAUGCCGCCCCCUGGU
AUGAUCCCCCCGCCAGGCCUUGCACCUGGCCAGAUCCCACCAGGGGCCAUGCCCCCGC
AGCAGCUUAUGCCAGGACAGAUGCCCCCUGCCCAGCCUCUUUCUGAGAAUCCACCGAAU
CACAUCUUGUUCCUCACCAACCUGCCAGAGGAGACCAACGAGCUCAUGCUGUCCAUGCU
UUUCAAUCAGUUCCCUGGCUUCAAGGAGGUCCGUCUGGUACCCGGGCGGCAUGACAUC
GCCUUCGUGGAGUUUGACAAUGAGGUACAGGCAGGGGCAGCUCGCGAUGCCCUGCAGG
GCUUUAAGAUCACGCAGAACAACGCCAUGAAGAUCUCCUUUGCCAAGAAGUAGucuagacc
uucugcggggcuugccuucuggccaugcccuucuucucucccuugcaccuguaccucuuggucuuuGAAUAAAGCC
UGAGUAGGAAAAAAAAAAAAAAAAAAAAAAAAAAAAAAAAAAAAAAAAAAAAAAAAAAAAA
AAAAAAAAAAAAAAAAAAAAAAAAAAAAAAAAAAAAAAAAAAAAAAAAAAAAAAAAAAAAAAAA
AAA

MetLuc2 linear switch (MS2CP)

MetLuc2, MS2CP binding motif

GGUCAGAUCCGCUAGCGGAUCCGGGAGCAGGUGAGGAUCACCCAUCUGCCACGAGCGAGGUGAGGAUCACCCAUCUCGCUCGUGUUCCCACCGGUCgccaccauggacaucaagguggguguucacccugguguucagcgcccuggugcaggccaagagcaccgaguucgaccccaacaucgacaucgugggccuggaaggcaaguucggcaucaccaaccuggaaaccgaccuguucaccaucugggagaccauggaagugaugaucaaggccgacaucgccgacaccgaccgggccagcaacuucguggccaccgagaccgacgccaaccggggcaagaugcccggcaagaagcugccccuggccgucaucauggaaauggaagccaacgccuucaaggccggcugcacccggggcugccugaucugccugagcaagaucaagugcaccgccaagaugaagguguacauccccggcaggugccacgacuacggcggcgacaagaaaaccggccaggccggcaucgugggcgccaucguggacauccccgagaucagcggcuucaaagaaauggcccccauggaacaguucaucgcccagguggacagaugcgccagcugcaccaccggcugccugaagggccuggccaacgugaagugcagcgagcugcugaagaaguggcugcccgaccgcugcgccagcuucgccgacaagauccagaaagaggugcacaacaucaagggcauggccggcgacaggugaucuagaccuucugcggggcuugccuucuggccaugcccuucuucucucccuugcaccuguaccucuuggucuuuGAAUAAAGCCUGAGUAGGAAAAAAAAAAAAAAAAAAAAAAAAAAAAAAAAAAAAAAAAAAAAAAAAAAAAAAAAAAAAAAAAAAAAAAAAAAAAAAAAAAAAAAAAAAAAAAAAAAAAAAAAAAAAAAAAAAAAAAAA

MetLuc2 linear switch (U1A)

MetLuc2, U1A binding motif

GACAGCAUUGUACCCAGAGUCUGUCCCCAGACAUUGCACCUGGCGCUGUCCGCAGAUCGAGAAGAAGGCGAAUUAAGAGAGAAAAGAAGAGUAAGAAGAAAUAUAAGACACCGGUgccaccauggacaucaagguggguguucacccugguguucagcgcccuggugcaggccaagagcaccgaguucgaccccaacaucgacaucgugggccuggaaggcaaguucggcaucaccaaccuggaaaccgaccuguucaccaucugggagaccauggaagugaugaucaaggccgacaucgccgacaccgaccgggccagcaacuucguggccaccgagaccgacgccaaccggggcaagaugcccggcaagaagcugccccuggccgucaucauggaaauggaagccaacgccuucaaggccggcugcacccggggcugccugaucugccugagcaagaucaagugcaccgccaagaugaagguguacauccccggcaggugccacgacuacggcggcgacaagaaaaccggccaggccggcaucgugggcgccaucguggacauccccgagaucagcggcuucaaagaaauggcccccauggaacaguucaucgcccagguggacagaugcgccagcugcaccaccggcugccugaagggccuggccaacgugaagugcagcgagcugcugaagaaguggcugcccgaccgcugcgccagcuucgccgacaagauccagaaagaggugcacaacaucaagggcauggccggcgacaggugaucuagaccuucugcggggcuugccuucuggccaugcccuucuucucucccuugcaccuguaccucuuggucuuuGAAUAAAGCCUGAGUAGGAAAAAAAAAAAAAAAAAAAAAAAAAAAAAAAAAAAAAAAAAAAAAAAAAAAAAAAAAAAAAAAAAAAAAAAAAAAAAAAAAAAAAAAAAAAAAAAAAAAAAAAAAAAAAAAAAAAAAAAAAAAA

Supplementary References

S1. Gale,M., Li,Y., Cao,J., Liu,Z. Z., Holmbeck,M.A., Zhang,M., Lang,S.M., Wu,L., Carmo,M.D., Gupta,S. *et al.* (2020) Acquired resistance to HER2-targeted therapies creates vulnerability to ATP synthase inhibition. *Cancer Res.* **80**, 524–535.

S2. Warren,L., Manos,P.D., Ahfeldt,T., Loh,Y.H., Li,H., Lau,F., Ebina,W., Mandal,P. K., Smith,Z.D., Meissner,A. et al. (2010) Highly efficient reprogramming to pluripotency and directed differentiation of human cells with synthetic modified mRNA. *Cell Stem Cell.* **7**, 618–630.

S3. Chen,Y.G., Kim,M.V., Chen,X., Batista,P.J., Aoyama,S., Wilusz,J.E., Iwasaki,A. and Chang,H.Y. (2017) Sensing Self and Foreign Circular RNAs by Intron Identity. *Mol. Cell.* **67**, 228-238.e5.

S4. Liu,C.X., GuoS.K., Nan,F., Xu,Y.F., Yang,L. and Chen,L.L. (2022) RNA circles with minimized immunogenicity as potent PKR inhibitors. *Mol. Cell.* **82**, 420-434.e6.

S5. Sato,K., Hamada,M., Asai,K. and Mituyama,T. (2009) CentroidFold: A web server for RNA secondary structure prediction. *Nucleic Acids Res.* **37**, 277–280.

S6. Kawasaki,S., Fujita,Y., Nagaike,T., Tomita,K. and Saito,H. (2017) Synthetic mRNA devices that detect endogenous proteins and distinguish mammalian cells. *Nucleic Acids Res.* **45**, e117.
